# Neural correlates of depression-related smartphone language use in adolescents

**DOI:** 10.1038/s44277-024-00009-6

**Published:** 2024-07-09

**Authors:** Elizabeth A. McNeilly, Giana I. Teresi, Saché Coury, Zia Bajwa, Lauren E. Kahn, Ryann Crowley, Nicholas B. Allen, Tiffany C. Ho

**Affiliations:** 1 Department of Psychology, University of Oregon, Eugene, OR USA; 2 Department of Psychology, University of Pittsburgh, Pittsburgh, PA USA; 3 Department of Psychology, University of California, Los Angeles, CA USA; 4 Department of Psychology, University of California, Berkeley, Berkeley, CA USA

**Keywords:** Emotion, Human behaviour, Social behaviour

## Abstract

Magnetic resonance imaging has provided pathophysiological insights into adolescent depression but is a relatively inaccessible technology. Generating scalable indicators of depression that are informed by neuroscience is therefore critical for providing solutions that allow us to detect and treat this devastating disorder. In this preregistered study, we investigated whether passively acquired smartphone-based language usage represents such an indicator of depression and explored whether the neural correlates of depression mediate or moderate this association. Forty adolescents (ages 14–18 years) with (*n* = 26) and without (*n* = 14) depression completed clinical assessments and a resting-state fMRI scan, prior to downloading a passive mobile sensing app to their smartphones. Linguistic features derived from over 1.2 million words (319,364 messages) across all smartphone apps were used to examine word usage patterns. Independent components analysis followed by dual regression was used to derive intrinsic networks commonly associated with depression: central executive network (CEN), default mode network (DMN), and salience network (SN). Depression was associated with more negative emotion word usage and fewer future-focus word usage on a daily basis (all *ps* < 0.05). Higher depressive symptoms and brain networks DMN and CEN were associated with greater first-person pronoun usage (all *p*s < 0.04). Accounting for CEN connectivity amplified the positive association between depressive symptoms and first-person pronoun usage. Lower SN–CEN connectivity moderated the association between depression and negative emotion word usage. Depression in adolescents is associated with naturalistic language usage during smartphone activities and may represent neurocognitive biases that are candidate treatment targets for interventions.

## Lay summary

We analyzed keyboard usage on smartphones and brain imaging data from 40 adolescents with and without depression. We found that those experiencing depression were more likely to use words related to self-focus and negative emotions but less likely to use future-focused words. Brain activity in regions involved in depression were also related to these language patterns. Our results indicate that the type of smartphone language adolescents use day-to-day may potentially reflect neurobiological risk for depression.

## Neural correlates of depression-related smartphone language use in adolescents

Video

## Introduction

Adolescence is a developmental period characterized by significant changes in functional brain development [1] and heightened risk for depression [2]. While advances in noninvasive neuroimaging, such as magnetic resonance imaging (MRI), have provided important insight into the neural correlates of adolescent depression, such technologies are expensive and not widely accessible. Generating scalable indicators of depression that are informed by neuroscience is therefore critical for providing solutions that allow us to detect and treat this devastating disorder.

Prior research in this area has suggested that particular linguistic features detected in naturalistic language reflect depression [3]. Specifically, the use of first-person pronouns, which may reflect cognitive biases such as self-focused attention [4] and reduced psychological distance [5], and negative emotion words, which may reflect biases in processing affective stimuli, have been found to be associated with depressive symptoms in adults [3, 6, 7]. Recent evidence indicates that such linguistic features can be reliably assayed from *digital* language and are associated with depression-related outcomes, such as suicide risk, dysphoria, and low mood [8, 9]. Smartphones, which are used almost ubiquitously among adolescents [10], offer a window into naturalistic language usage in this population, and, thus, provide an opportunity to observe ecologically-valid linguistic markers of depression in adolescents. For instance, recent research from our team demonstrated that in a community sample of early adolescent girls, smartphone-derived linguistic features associated with self-focus (i.e., greater usage of first-person pronouns) and reduced temporal distance (i.e., reduced usage of future-focus words) were expressed more on days characterized by lower mood (relative to an individual’s average mood) [9]. Yet it remains unknown whether these patterns exist in adolescents with depression.

Further, certain cognitive patterns of depression, including self-focused attention [4], rumination [11], and a negative bias [12] may manifest in everyday language usage and have been found to be associated with functional brain networks that are implicated in depression [13, 14]. Specifically, depression among adolescents has been found to be associated with alterations in intrinsic brain networks including the Central Executive Network (CEN) [15], which is involved in goal-directed processing and executive functioning [16]; the Default Mode Network (DMN) [17], which is involved in depression-relevant processes such as self-referential processing and rumination [14, 18]; and the Salience Network (SN) [19], which is involved in salience detection and adaptively shifting between internally and externally focused mental states [20].

Despite the ubiquity of smartphones in the lives of youth today, empirical research on the associations between specific smartphone use behaviors, mental health, and brain function is lacking. Specifically, we do not know whether the neural correlates of depression-related naturalistic language usage share the same neural substrates as depression or whether these brain patterns mediate or moderate associations between naturalistic language usage and depression. Although recent research suggests that structural brain development is differentially associated with levels of adolescent social media use and mental well-being [21], no prior studies have examined these processes in relation to brain *function* and, critically, in a sample of adolescents with Major Depressive Disorder (MDD) and demographically-matched healthy controls (CTL). Investigating linguistic features of smartphone activity as they relate to individual differences in neural correlates of depression will more clearly identify potential mechanisms and will bring us closer to identifying scalable biomarkers of depression.

In the present study, we acquired in vivo smartphone-based language among adolescents (ages 14-18 years) with and without MDD using keyboard data acquired through the Effortless Assessment Research System (EARS) [22]. Within- and between-network connectivity of the CEN, DMN, and SN were identified for each participant from an 8-minute resting-state fMRI scan. Our study objectives were to identify which linguistic features from keyboard data are associated with depression in adolescence (Aim 1), whether within- and between-network connectivity of the CEN, DMN, and SN relate to the same linguistic usage patterns associated with depression in our sample (Aim 2) and whether these neural patterns account for (Aim 3), or strengthen (Aim 4), the association between naturalistic language usage and depression in our sample. We hypothesized that depression would have a positive association with first-person pronouns, negative emotion words, present-focus words, and future-focus words and a negative association with positive emotion words, past-focus words, and word count (Aim 1). Based on extant literature highlighting the key role of the DMN in depression [17, 23], we hypothesized that higher DMN within-network connectivity and greater positive coupling between DMN with CEN and SN will be related to the linguistic features we examined in the same manner as depression (Aim 2). We did not make *a priori* hypotheses for Aims 3 and 4 as they are exploratory, given the lack of prior research in this domain. By elucidating whether there are naturalistic linguistic features that are associated with specific neural patterns commonly linked with depression, we will be paving the way for identifying potentially scalable digital biomarkers of depression in adolescence.

## Methods

### Participants

The present study was drawn from a larger 18-month longitudinal investigation [24]. The analytic sample consisted of 40 (26 MDD, 14 CTL) adolescents (ages 14–18 years; 65% female). See Table 1 for sociodemographic characteristics of the sample. Participants were recruited from the San Francisco Bay Area in California through posted flyers, online advertisements, and an internal referral program. Beginning in January 2020 (~2 years after study recruitment), study participants were invited to participate in a substudy that involved downloading the Effortless Assessment Research System (EARS) software application (or “app”). Participants and their parent/legal guardian(s) completed written assent and informed consent, respectively, and were financially compensated for their participation. The institutional review boards of Stanford University, the University of California, San Francisco, and the University of California, Los Angeles approved this study.

**Table 1:** Sociodemographic characteristics and descriptive statistics of key variables.

Variable	MDD (*n* = 26)	CTL (*n* = 14)
Age at Scan	M = 16.25 years (SD = 1.31)	M = 16.02 years (SD = 0.92)
Sex at Birth		
Male	9 (35%)	5 (36%)
Female	17 (65%)	9 (64%)
Gender		
Male	8 (31%)	5 (36%)
Female	13 (50%)	9 (64%)
Other	5 (19%)	0 (0%)
Ethnicity		
Hispanic or Latino	5 (19%)	0 (0%)
Not Hispanic or Latino	21 (81%)	14 (100%)
Race		
American Indian or Alaska Native	1 (4%)	0 (0%)
Asian	4 (15%)	7 (50%)
Black or African American	1 (4%)	0 (0%)
Native Hawaiian or Other Pacific Islander	0 (0%)	0 (0%)
White	13 (50%)	5 (36%)
Multiracial	6 (23%)	1 (7%)
Other	1 (4%)	1 (7%)
Highest Parental Education		
Without HS diploma	0 (0%)	0 (0%)
HS graduate without college education	0 (0%)	0 (0%)
Some college education	5 (19%)	0 (0%)
Degree from 4-year college or more	21 (81%)	14 (100%)
Psychotropic Medication	12 (46%)	0 (0%)
RADS-2 Total Score	M = 81.27 (SD = 14.01)	M = 49.43 (SD = 10.4)
Age of First Major Depressive Episode	M = 12.27 years (SD = 2.4)	NA
EARS App # Days	M = 67.73 (4 – 326)	M = 35.0 (1 – 116)
Time to EARS Start (days)	M = 295 (0 – 779)	M = 327.14 (0 – 563)
Linguistic Features (proportion of daily words)		
First-Person Pronouns	M = 8.32 (SD = 1.86)	M = 7.37 (SD = 1.43)
Positive Emotion Words	M = 4.51 (SD = 1.26)	M = 4.48 (SD = 1.34)
Negative Emotion Words	M = 2.53 (SD = 0.51)	M = 1.51 (SD = 0.56)
Past-focus Words	M = 3.04 (SD = 0.66)	M = 2.98 (SD = 0.92)
Present-focus Words	M = 11.20 (SD = 1.23)	M = 11.53 (SD = 1.53)
Future-focus Words	M = 1.43 (SD = 0.54)	M = 1.74 (SD = 0.57)
Daily Word Count	M = 530.84 (SD = 597.43)	M = 221.15 (SD = 98.39)
Within-Network Connectivity		
DMN	M = 1.50 (SD = 0.32)	M = 1.63 (SD = 0.51)
SN	M = 2.50 (SD = 0.43)	M = 2.70 (SD = 0.53)
Right CEN	M = 1.99 (SD = 0.43)	M = 2.32 (SD = 0.45)
Left CEN	M = 1.78 (SD = 0.31)	M = 2.11 (SD = 0.25)
Between-Network Connectivity		
DMN – SN	M = 0.25 (SD = 0.30)	M = 0.11 (SD = 0.36)
DMN – right CEN	M = 0.22 (SD = 0.32)	M = 0.49 (SD = 0.30)
DMN – left CEN	M = 0.34 (SD = 0.30)	M = 0.27 (SD = 0.27)
SN – right CEN	M = 0.31 (SD = 0.28)	M = 0.44 (SD = 0.20)
SN – left CEN	M = 0.33 (SD = 0.25)	M = 0.48 (SD = 0.27)
Framewise Displacement Value (Motion)	M = 0.08 (SD = 0.02)	M = 0.11 (SD = 0.04)

*CEN* Central executive network, *CTL* Controls, *DMN* Default mode network, *MDD* Major depressive disorder, *SN* Salience network.

### Procedures

This study was preregistered on the Open Science Framework (https://osf.io/u2k6h). Deviations from the preregistration are explained in Supplement 1.

#### Data collection

As part of a pilot study funded by a supplemental award to the parent study (K01MH117442), participants were invited to download the EARS app on their smartphone (iPhone or Android). These data were encrypted, stored on a secure server in the cloud, and subsequently downloaded and decrypted by the research team. Further details on the engineering, encryption, and secure storage of the data can be found in Lind et al. [22].

While the EARS portion of the study was added to the study protocol in January 2020, all participants, regardless of study completion status, were invited to download the app in March 2020 due to the COVID-19 pandemic; thus, participants chose to participate at different points of progression throughout the study. As such, we conducted sensitivity analyses on the time between the baseline fMRI visit and the EARS data collection, and time-related COVID-19 nuisance covariates (see Supplement 1 for results).

### Measures

#### Depression diagnosis

Assignment to the MDD group was based exclusively on meeting diagnostic criteria for a depressive disorder (Major Depressive Disorder, Dysthymia, or Depressive Disorder Not Otherwise Specified) according to clinical interviews [24]. Group assignment was a binary variable based on the presence (MDD group) or absence (CTL group) of a depression diagnosis. Depression diagnoses were determined using a combination of the Kiddie Schedule for Affective Disorders and Schizophrenia Interview – Present and Lifetime Version (K-SADS-PL) [25] and the Children’s Depression Rating Scale-Revised [26]. The KSADS-PL is a semi-structured clinical interview of child and parent designed to yield reliable and valid diagnoses of psychiatric disorders. The CDRS-R is a widely used clinician-rated scale for assessing depressive symptom severity through an integration of child- and parent-report. The senior author reviewed and finalized all KSADS-PL codes and CDRS-R scores with study assessors. See Supplement 1 for further details on inclusion/exclusion criteria by group (MDD, CTL).

#### Depressive symptoms

Self-reported depressive symptoms were measured with the Reynolds Adolescent Depression Scale (RADS-2) [27], a 30-item survey that has been validated in adolescents through age 20. The total score (item sum) of the RADS-2 was used in all analyses involving symptom severity. Depressive symptoms were based on self-report responses to RADS-2 assessed across all participants (MDD and CTL group).

#### Text data

The EARS app passively collected all keystrokes on the participant’s smartphone via a keylogger, along with the app in which the message was entered. Text data were processed by the Linguistic Inquiry and Word Count (LIWC) 2015 software [28], which identifies specific linguistic categories as the proportion of total words that each category represents within a given body of text. LIWC has been used in studies of online language as advances in technology have made that type of data more available, including linguistic features from text messages [9], Facebook [29], Reddit [30], and Twitter [31]. Linguistic features were the primary outcome of interest and were analyzed at the daily level as proportions of total words entered in a day (i.e., a score of 5 indicated 5% of total words in a given day). This approach to processing text data has been used previously by our group [9]. Further details regarding the proportion calculation are provided in Supplement 1.

#### Resting-state functional connectivity

Following previous work by our group [32, 33], resting-state fMRI data was preprocessed using field-consensus procedures for mitigating the effects of motion; participants were excluded from analyses if more than 20% of volumes exceeded a mean framewise displacement (FD) of 0.25 mm. Five participants met these thresholds and were excluded (mean FD of final analytic sample = 0.9 ± 0.03 mm). All subsequent data was then submitted to group-level independent component analysis (ICA) using FSL MELODIC. ICA is a data-driven multivariate signal-processing method used to characterize spatiotemporal properties of timeseries data derived from functional MRI that defines a spatial network of voxels based on their temporal correlations. We visually examined each network component generated from the group ICA and extracted individual-level metrics of network coherence from the left and right CEN, DMN, and SN (see Fig. S2 in Supplement 1 for network visualization).

### Statistical analysis

To disaggregate within- and between-person variance of linguistic features, multilevel modeling (MLM) using random intercepts and slopes was employed. When significant main effects on linguistic features were observed for Aims 1 and 2, exploratory analyses were conducted to investigate Aims 3 and 4. For Aim 3, we conducted three separate models to test the extent to which resting-state functional connectivity (rsFC) networks accounted for variance in the association between depression and linguistic features: 1) the effect of depression on the linguistic features identified in Aim 1, 2) the effect of depression on rsFC networks identified in Aim 2, and 3) the effect of these rsFC networks on the identified linguistic features, while controlling for depression. We used the *RMediation* package in R to estimate the indirect effect that rsFC networks had on the association between depression and linguistic features. For Aim 4, we constructed interaction models to test whether rsFC networks identified in Aim 2 moderate the association between depression and linguistic features identified in Aim 1. See Supplement 2 for the primary analysis code and output.

Sensitivity analyses were conducted on all models to test whether the model fit improved by adding age, sex, and gender, along with nuisance covariates related to timing of assessments and COVID-19. In all models involving rsFC, we tested whether the results held when mean FD values were included as a covariate. See Supplement 1 for sensitivity analysis methods.

Hierarchical model comparisons using likelihood ratio tests (LRTs) were used to determine the best fit model when comparing models of main effects with models containing covariates as sensitivity analyses. Criteria for the best-fitting model were having the lowest Bayesian information criterion (BIC) and passing the likelihood-ratio test (*p* < 0.05) when compared to the simpler model.

Effect sizes are reported as the standardized coefficient estimate, along with the 95% confidence interval, from the best fit model. All models were estimated in the most recent version of R [34] using the *lme4* package [35] for multilevel model estimation and the *psych* package for descriptive statistics [36]. Outliers were examined using the *influence.ME* package in R [37]. To correct for family-wise error, we used false discovery rate (FDR) estimation for Aims 1 and 2 (as Aims 3 and 4 were exploratory). For Aim 1, we corrected for two types of depression assessments—depression diagnosis and depressive symptoms—when estimating the effect on linguistic features. For Aim 2, we corrected for the number of network connectivity values (4) hypothesized involving the DMN among patients with MDD and CTL. Unadjusted and adjusted *p*-values are presented in Tables 2 and 3.

**Table 2:** Fixed effects from linear mixed effects models for Aim 1 estimating the effect of A) depression (group) and B) depressive symptoms (continuous) on smartphone-based linguistic features. Results from the best fit models based on likelihood ratio tests are reported.

Outcome	β	95% CI	*t*	*p*	*p adj*.	Model
Depression (Group)						
First-person pronouns	0.25	[−0.05, 0.56]	1.616	0.115	0.115	1a
Positive emotion words	−0.02	[−0.36, 0.32]	−0.112	0.912	0.912	1b
Negative emotion words	**0.58**	**[0.34, 0.82]**	**4.771**	**<0.001**	**<0.001**	1c
Past-focus words	0.08	[−0.17, 0.33]	0.660	0.514	0.564	1d
Present-focus words	−0.08	[−0.34, 0.19]	−0.579	0.566	0.768	1e
Future-focus words^a^	**−0.29**	**[−0.53, −0.05]**	**−2.411**	**0.023**	**0.043**	1f.2^a^
Daily Word Count	0.38	[−0.03, 0.78]	1.833	0.074	0.075	1g
Depressive Symptoms						
First-person pronouns	**0.19**	**[0.06, 0.31]**	**2.929**	**0.006**	**0.012**	S1a
Positive emotion words	0.03	[−0.12, 0.18]	0.425	0.673	0.912	S1b
Negative emotion words	**0.23**	**[0.12, 0.34]**	**4.186**	**<0.001**	**<0.001**	S1c
Past-focus words	0.03	[−0.08, 0.14]	0.583	0.564	0.564	S1d
Present-focus words	−0.02	[−0.13, 0.10]	−0.297	0.768	0.768	S1e
Future-focus words^a^	**−0.11**	**[−0.22, −0.01]**	**−2.126**	**0.043**	**0.043**	S1f.2^a^
Daily Word Count	0.17	[−0.01, 0.35]	1.829	0.075	0.075	S1g

Bold values indicate statistical significance *p* < 0.05.

^a^Best fit model covaried for age. See Supplement 3.

All models included only a random intercept for participant. Model number corresponds to the model output in Supplement 2, except for 1 f.2 and S1f.2 in Supplement 3. β = Standardized coefficient. Unadjusted p-values (*p)* and FDR-corrected adjusted *p* values (*p adj*.) for multiple comparisons with 2 depression-related measures.

**Table 3:** Significant fixed effects from linear mixed effects models for Aim 2.

Predictor – Outcome	β	95% CI	*t*	*p*	*p adj*.	Model
DMN – First-person pronouns	**0.12**	**[0.01, 0.23]**	**2.152**	**0.038**	0.152	S2a.1
Left CEN – First-person pronouns	**0.19**	**[0.04, 0.34]**	**2.464**	**0.019**	—	S2a.2
SN – Negative emotion words	**0.11**	**[0.01, 0.20]**	**5.114**	**0.032**	—	2b.4
DMN – Future-focus words^a^	**0.11**	**[0.02, 0.21]**	**2.291**	**0.029**	0.105	2c.1b^a^

Bold values indicate statistical significance *p* < 0.05.

^a^Best fit model covaried for age. See Supplement 3.

We report results from the best fit models based on likelihood ratio tests. All models included a random intercept for participants and covaried for depression. Model number corresponds to the model output in Supplement 2, except for 2c.1b in Supplement 3. β = Standardized coefficient. *p =* unadjusted p-value. *p adj*. = FDR-corrected adjusted p-values for multiple comparisons with 4 DMN-related networks.

## Results

### Descriptive statistics

Descriptive statistics for the sample, group assignment (MDD or CTL), and key variables are displayed in Table 1. For a detailed summary of results from Aims 1 and 2, see Tables 2 and 3, respectively. Sensitivity analysis results are presented in Supplement 1 and the accompanying code and output are presented in Supplement 3. Our results did not change as a result of including age, sex, or mean FD values. We note below if model fit was improved by adding these covariates.

### Aim 1: What are the features of smartphone-based language that are associated with depression?

#### First-person pronouns

**Fig. 1:**
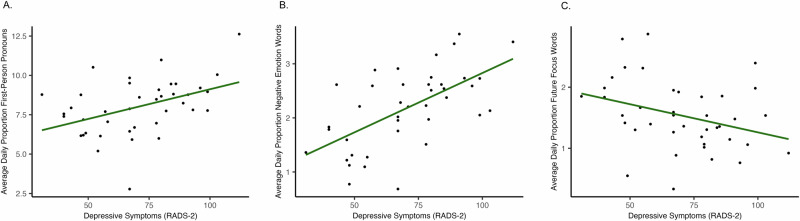
Associations between depressive symptoms (RADS-2 total scores) and the average daily proportion of linguistic features relative to total words (e.g., 5 = 5% of total words per day). The solid line represents the overall predicted regression. **A** Higher depressive symptoms were associated with a higher average daily proportion of first-person pronouns. **B** Higher depressive symptoms were associated with a higher average daily proportion of negative emotion words. **C** Higher depressive symptoms were associated with a higher average daily proportion of future-focus words.

Depressive symptoms were positively associated with first-person pronoun use (Fig. 1A; β = 0.19, *p* = 0.006). However, we did not observe an association between MDD and first-person pronouns (β = 0.25, p = 0.115). One multivariate outlier was identified in the association between depressive symptoms and first-person pronouns. The positive association remained significant when the model was tested without the outlier. See Table 2.

#### Negative emotion words

Depressive symptoms were positively associated with negative emotion words (Fig. 1B; β = 0.23, *p* < 0.001). Furthermore, compared to the CTL group, the MDD group entered a higher daily proportion of negative emotion words into their smartphones (*β* = 0.58, *p* < 0.001).

#### Future-focus words

Depressive symptoms had a negative association with future-focus words (β = −0.11, *p* = 0.043). Hierarchical model comparisons revealed that age, but not sex, improved model fit when investigating the association with depressive symptoms, χ^2^(1, *N* = 40) = 4.21, *p* = 0.04. Similarly, those with MDD used a lower proportion of future-focus words (Fig. 1C; β = −0.29, *p* = 0.023). Adding age, but not sex, to the model improved model fit, χ^2^(1, *N* = 40) = 5.91, *p* = 0.015.

#### Other linguistic features

We observed no associations between depression and positive emotion words, past-focus words, present-focus words, or daily word count.

### Aim 2: what are the intrinsic network connectivity patterns associated with the linguistic features identified in Aim 1?

#### First-person pronouns

DMN within-network connectivity showed a positive association with first-person pronoun use, when controlling for depressive symptoms (β = 0.12, *p* = 0.038). Left CEN within-network connectivity had a positive association with first-person pronouns, when controlling for depressive symptoms (β = 0.19, *p* = 0.019). See Table 3. We did not observe any significant associations with between-network connectivity and first-person pronoun use (Supplement 2).

#### Negative emotion words

SN within-network connectivity had a positive association with negative emotion words, while controlling for MDD diagnosis (β = 0.11, *p* = 0.032). Similarly, SN within-network connectivity had a positive association with negative emotion words, while controlling for depressive symptoms (β = 0.14, *p* = 0.011). We observed no associations with between-network connectivity and negative emotion words (Supplement 2).

#### Future-focus words

DMN within-network connectivity had a positive association with future-focus words, while controlling for MDD diagnosis (β = 0.11, *p* = 0.029). Hierarchical model comparisons revealed that adding age, but not sex, to the model improved model fit, χ^2^(1, *N* = 40) = 4.31, *p* = 0.038. Alternatively, when controlling for depressive symptoms, we did not observe an association between DMN within-network connectivity and future-focus words (β = 0.10, *p* = 0.061). We observed no associations with between-network connectivity and future-focus words (Supplement 2 Aim 2).

### Exploratory Aim 3: if Aims 1 and 2 are significant, are intrinsic network connectivity patterns associated with the shared variance between depression and linguistic features?

**Fig. 2:**
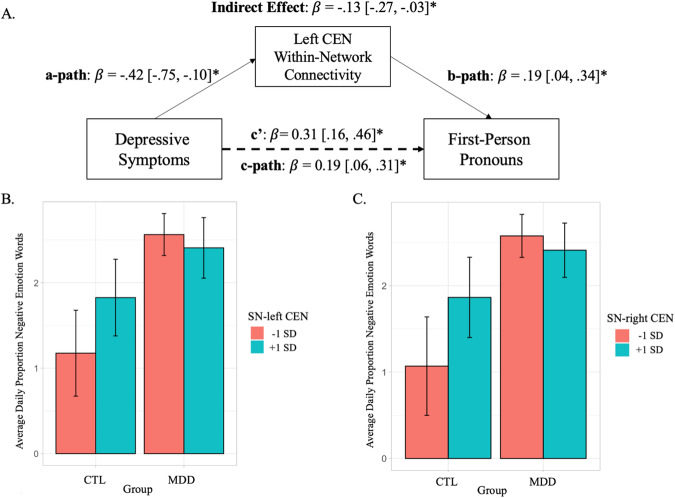
Mediation and moderation effects of neural correlates on depression-related language use. **A** Path diagram depicting indirect effect (suppression) of left CEN connectivity on the association between depressive symptoms and daily proportion of first-person pronouns. C’ represents the direct effect, controlling for left CEN connectivity. *β* = Standardized beta estimate. 95% confidence intervals presented in brackets. Asterisk (*) indicates p < 0.05. **B**, **C** Bar charts depicting the moderation effect from higher (+1 SD) and lower −1 SD) SN-CEN between-network connectivity on the association between group (control or MDD) and daily proportion of negative emotion words (e.g., 2 = 2% of total words). Error bars represent the 95% confidence interval.

Left CEN within-network connectivity had an indirect effect on the positive association between depressive symptoms and first-person pronouns (Fig. 2A; β = −0.13, 95% CI [−0.27, −0.03]). Adolescents with higher depressive symptoms used relatively more first-person pronouns on a daily basis, and the magnitude of this association was strengthened when accounting for greater left CEN within-network connectivity (Supplement 2, model 3a). This association remained when age, sex, and mean FD values were included in the model (Supplement 3, model 3a.2). We also tested for, but did not observe, an indirect effect of DMN within-network connectivity on the association between depression with first-person pronouns. All other models we tested (i.e., with negative emotion words and future-focus words as outcome measures) did not generate significant indirect effects. See Supplement 2 for more details.

### Exploratory Aim 4: is the association between depression and linguistic features moderated by intrinsic network connectivity patterns?

#### Negative emotion words

SN and CEN between-network connectivity moderated the association between MDD and negative emotion words. This association was observed in both the left CEN (Fig. 2B; β = −0.26, *p* = 0.045) and the right CEN (Fig. 2C; β = −0.26, *p* = 0.035). See Supplement 2, models 4b.3 and 4b.4, respectively. Lower SN-CEN between network connectivity was associated with using more negative emotion words for adolescents with depression, when compared to the CTL group. No other intrinsic network connectivity patterns moderated the association between depression and linguistic word usage (i.e., first-person pronouns and future-focus words). See Supplement 2 for more details. These associations remained when age, sex, and mean FD values were included in the model (Supplement 3, models 4b.3–4b.4).

## Discussion

This preregistered study is the first to examine the extent to which depression and neural correlates of depression are associated with naturalistic smartphone language patterns (ascertained passively through smartphone keyboard data), and whether neural correlates of depression are associated with or influence (i.e., moderate) this association in adolescents. Certain neurobiological features of depression may be represented by behavioral differences in smartphone-based language use, addressing an existing gap in clinical translation within the field [38]. As such, this study is a proof-of-concept that smartphones, and digital phenotyping methods more broadly, hold translational value in the field of adolescent neuropsychiatry.

As hypothesized in Aim 1, depressive symptoms were associated with higher daily use of first-person pronouns. It appears that first-person pronouns is a linguistic feature that tracks dimensionally with subclinical depressive symptoms, or changes in daily mood [9], as opposed to a dichotomous diagnostic threshold. As expected, depression (symptoms and diagnosis) was associated with higher daily use of negative emotion words. There is a substantial body of evidence considering negative emotion words as a cognitive bias underlying the maintenance of depression [6, 12, 39]. We extend this important literature by demonstrating that these links between depression and both first-person pronouns and negative emotion words are observed in passively acquired smartphone keyboard data.

Contrary to our hypotheses for Aim 1, adolescents with depression typed fewer future-focus words into their smartphones. While the direction of this effect is opposite from what was expected [9, 40], this finding aligns with recent work indicating that the emotion regulation technique of psychological distancing is expressed via linguistic distance (i.e., future-focus words) [5] and may be difficult for adolescents with depression. Additionally, lower use of future-focus words by adolescents with depression could relate to feelings of hopelessness (whereas in our previous study comprised of a community sample [9], it may be that the positive association between future-focus words and depressive symptoms represents worry or rumination). Nevertheless, further research is needed to explore the underlying psychological process related to future-focus words and whether there may be moderators that influence the magnitude of this effect. Indeed, advancements in natural language processing using machine learning offer opportunities for future work to explore the underlying facets of the temporal dynamics of psychological distancing in relation to mental health [41].

Previous work in our group did not find an association between depressive symptoms and first-person pronouns or negative emotion words, but rather, only with future-focus word usage [9]. There were several differences between these two studies, however. First, the present study had a case-control design (whereas the prior study recruited a convenience sample), suggesting that perhaps these associations are observable when there is more range and variability in clinical severity. Second, the present study utilized a larger corpus of keyboard data—limited not only to social communication apps—which is important for clarifying that these signals reflect general tendencies across contexts. Nevertheless, it will be critical in future investigations to further probe app sources and determine if there are certain digital contexts in which these patterns are (or are not) present [42].

We found preliminary evidence that certain patterns of intrinsic connectivity commonly implicated in depression (CEN, DMN, and SN) [43] explained unique variance in word usage in a manner similar to depression, including a positive association between DMN connectivity and use of first-person pronouns. The DMN has long been considered to underpin self-referential processing [14]; this finding provides additional face validity that elevated use of first-person pronouns in a digital context reflects excessive self-referential processing. We also found that adolescents with greater left CEN connectivity used a higher proportion of first-person pronouns. While there is no clear precedent for this finding, one possibility is that the CEN, as defined in this study, overlaps in functioning with the DMN. The evidence supporting this view comes from using more precise mapping of intrinsic networks that fractionate the CEN into subsystems [44]. In other words, the internal organization of the CEN appears to be strongly associated with the DMN. Regardless, given the exploratory nature of these analyses, it is important to replicate this finding before it is strongly interpreted.

Interestingly, greater connectivity of the SN, when controlling for depression diagnosis, was associated with higher use of negative emotion words. This finding is consistent with extant research linking greater connectivity of the SN and rumination in adolescents [18] and with problematic smartphone use [45], suggesting that perhaps these maladaptive outcomes share a common neurobiological mechanism. Greater use of negative emotion words may represent an underlying neurocognitive process that contributes to the maintenance of depression through SN connectivity.

Finally, our finding that greater DMN connectivity is associated with using a higher proportion of future-focus words aligns with prior research that the DMN is implicated in future-oriented thought [46]. While the directionality of the associations is the opposite of what we found when relating depression with future-focus words, this result suggests that DMN connectivity explains unique variance in future-focus words that is likely due to the DMN supporting a broad range of adaptive and maladaptive future-focused cognition.

We explored whether network connectivity within and between CEN, DMN, and SN had an indirect effect on the association between depression and linguistic features and found a suppression effect from the left CEN, such that accounting for greater left CEN connectivity reveals a larger effect of depressive symptoms on first-person pronouns (as opposed to smaller as with a statistical mediation effect). Given the lack of precedence in the literature, we intend for this exploratory analysis to contribute to future hypothesis generation.

When testing whether CEN, DMN, and SN network connectivity moderated the association between depression and linguistic features, we found that the difference in use of negative emotion words between the MDD and CTL groups was strongest for those with lower SN-CEN connectivity. Adolescents with depression have shown resting-state *hypo*connectivity between and among the CEN, DMN, and SN [15]. Aberrant connectivity between the SN and CEN in particular has been conceptualized as a difficulty with regulating emotionally salient stimuli, a process likely to be more challenging for individuals with MDD, or particular subgroups within MDD [19]. This finding highlights the role of SN–CEN connectivity in the neurocognitive biases of depression and is meant to guide novel hypotheses in this area.

The present study had both methodological strengths and limitations. Our study included keyboard data from *any* smartphone app, which highlights the generalizability of depression-related linguistic patterns and extends prior investigations limited to language from social communication apps only [9, 47]. While 90.2% of the language in this study was derived from social media and text messaging (see Fig. S3), we also analyzed language from all other apps including web browsing and entertainment (e.g., YouTube), which captures what adolescents are consuming as well as communicating. While there was variability in the amount of time between the baseline assessment and collection of the EARS data, our results were largely robust when covarying by this factor. Our multimethod data provided a unique opportunity to test the convergent validity of depression-related linguistic features with the neuroscience of adolescent depression [38].

Nevertheless, future prospective studies are needed to unpack whether depression *causes* language biases in typical smartphone app usage or vice versa. Additionally, the present study’s sample size was relatively small. Given the nascent stage of this area of research, it will be necessary to replicate these findings in larger clinical samples before firm conclusions can be drawn. Further, even though the resting-state fMRI scan was sufficient for extracting group-derived levels of intrinsic networks, it will be important to consider using longer sequences that are adequate for identifying networks at the level of a single individual; such an approach is necessary for precisely mapping individual differences in linguistic patterns with neural functioning. Advances in sequence development that could generate such data with relatively shorter scan times (e.g., multi-echo sequences) [48], or dense-sampling techniques, provide an exciting opportunity to link granular brain network patterns with scalable indicators of depression, such as smartphone-derived language usage [49].

In conclusion, this study provides novel evidence that adolescent depression is associated with passively monitored language usage from a smartphone and that these indicators of depression relate to alterations in the CEN, DMN, and SN in promising ways that inform biomarker development. Depression-related linguistic patterns, such as first-person pronouns and negative emotion words, represent key psychological mechanisms (i.e., self-focused attention, negative cognitive bias) that can be targeted in treatment and have the potential to serve as indicators of treatment efficacy for adolescent depression [50]. Future work is needed to test the utility of smartphone-based language as scalable biomarkers representing neurocognitive targets for depression in adolescents.

## Supplementary information

Supplement 1

Supplement 2

Supplement 3

## Data Availability

Data reported in this study—depression diagnosis and symptoms, linguistic features, and resting-state functional connectivity—can be accessed through the project’s registration on the Open Science Framework: https://osf.io/b764q/files/osfstorage.
