# Supplementary material for: Neural correlates of depression-related smartphone language use in adolescents
Source: NPP Digit Psychiatry Neurosci. 2024 Jul 9;2:11. doi: 10.1038/s44277-024-00009-6 (PMC12592214; doi:10.1038/s44277-024-00009-6)
Supplement: Supplementary file 1 — Supplement 1 [file 44277_2024_9_MOESM1_ESM.docx]

**Supplement 1**

**Supplemental Methods**

**Inclusion/Exclusion Criteria**

The sample size was determined by 1) whether the participant met the inclusion criteria for the overall parent study, described below, 2) the number of participants who enrolled in EARS, and 3) recruitment for the parent study was completed in December 2021.

***Depression diagnosis***

All participants and a parent or legal guardian were interviewed by a trained study staff member and all K-SADS-PL diagnostic codes and CDRS-R scores were reviewed by the PI and staff members, weighing responses from the adolescent and their caregiver according to clinical judgment. Participants who were coded as subthreshold according to their responses on the K-SADS-PL depression module had to have a CDRS-R *t*-score of at least 55 to be included in the study as a depressed participant. All psychiatrically healthy controls (CTL) were required to have a CDRS-R *t*-score below 54 and no history of any Axis I disorder according to the K-SADS-PL or a first-degree relative with diagnosed or suspected mood or psychotic disorders. **Figure S1** depicts the inclusion and exclusion criteria for the parent study.

**
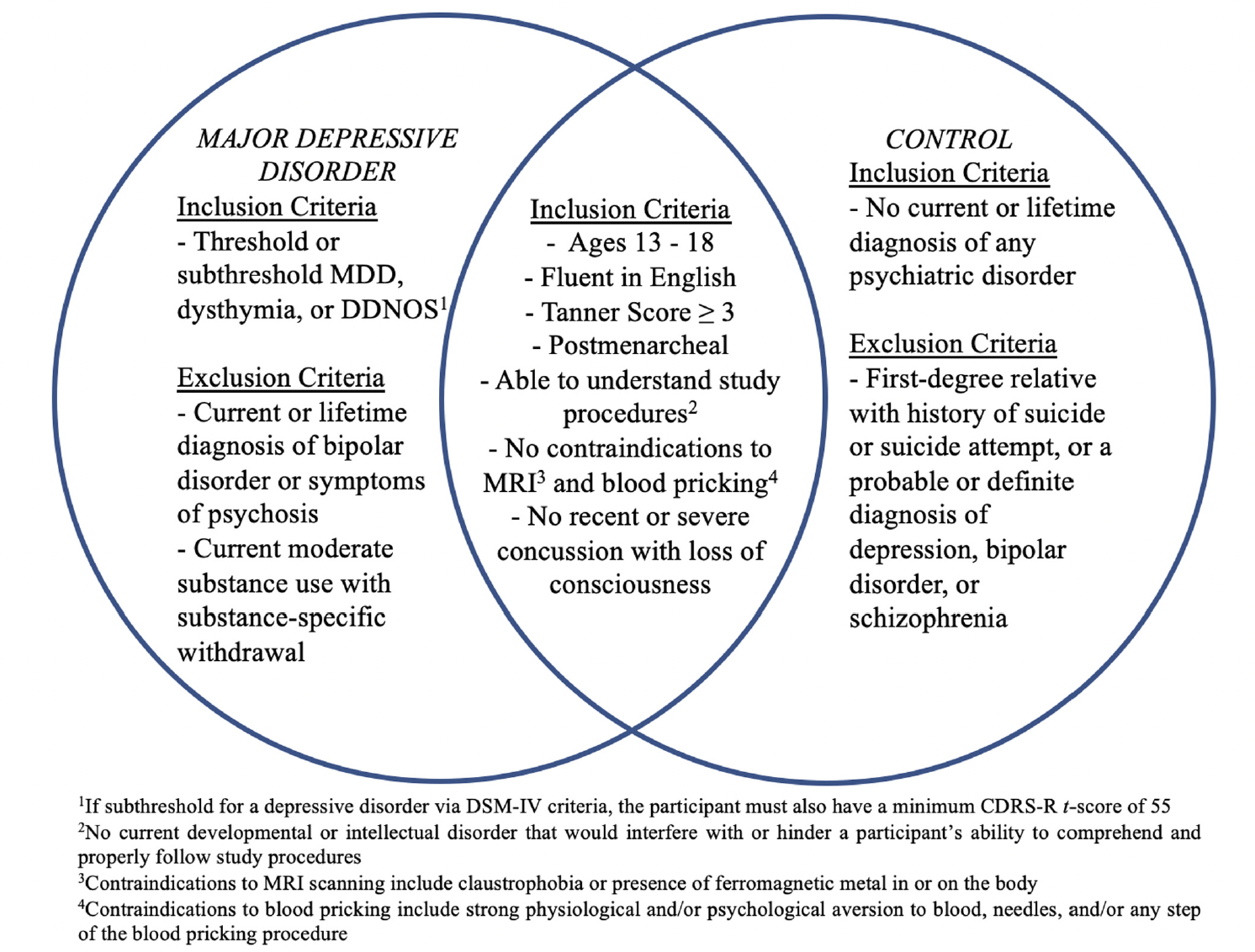
**

**Figure S1.** Figure from the parent study’s protocol [23].

**Calculation of Linguistic Features**

To arrive at the daily linguistic feature proportions, we first analyzed each message using LIWC, which calculated the proportion of total words in a message from a given linguistic category. For instance, a score of 10 for a linguistic category, such as first-person pronouns, indicated that 10% of the words in that message were first-person pronouns. Using both the proportion and the total number of words in a given message, we converted proportions to absolute word counts by message. Last, daily proportions of linguistic categories were a ratio of the total number of words within a category to the total words that day for an individual. Thus, each linguistic category was independent of the number of words entered on a given day, which controlled in part for individual differences in quantity of word usage. Total daily words were analyzed as a separate linguistic category of interest. Of the 2,706 daily observations, we applied a minimum threshold of 30 words for a given daily observation to be included, which resulted in 2,251 daily observations, or 83.19% of the original sample. The minimum of 30 words per day was identified by plotting the bivariate distributions of daily word count and the daily proportion of the linguistic feature and then estimating the quantity of daily words at which the daily proportion appeared to cease systematically producing outliers.

**Intrinsic Brain Networks**


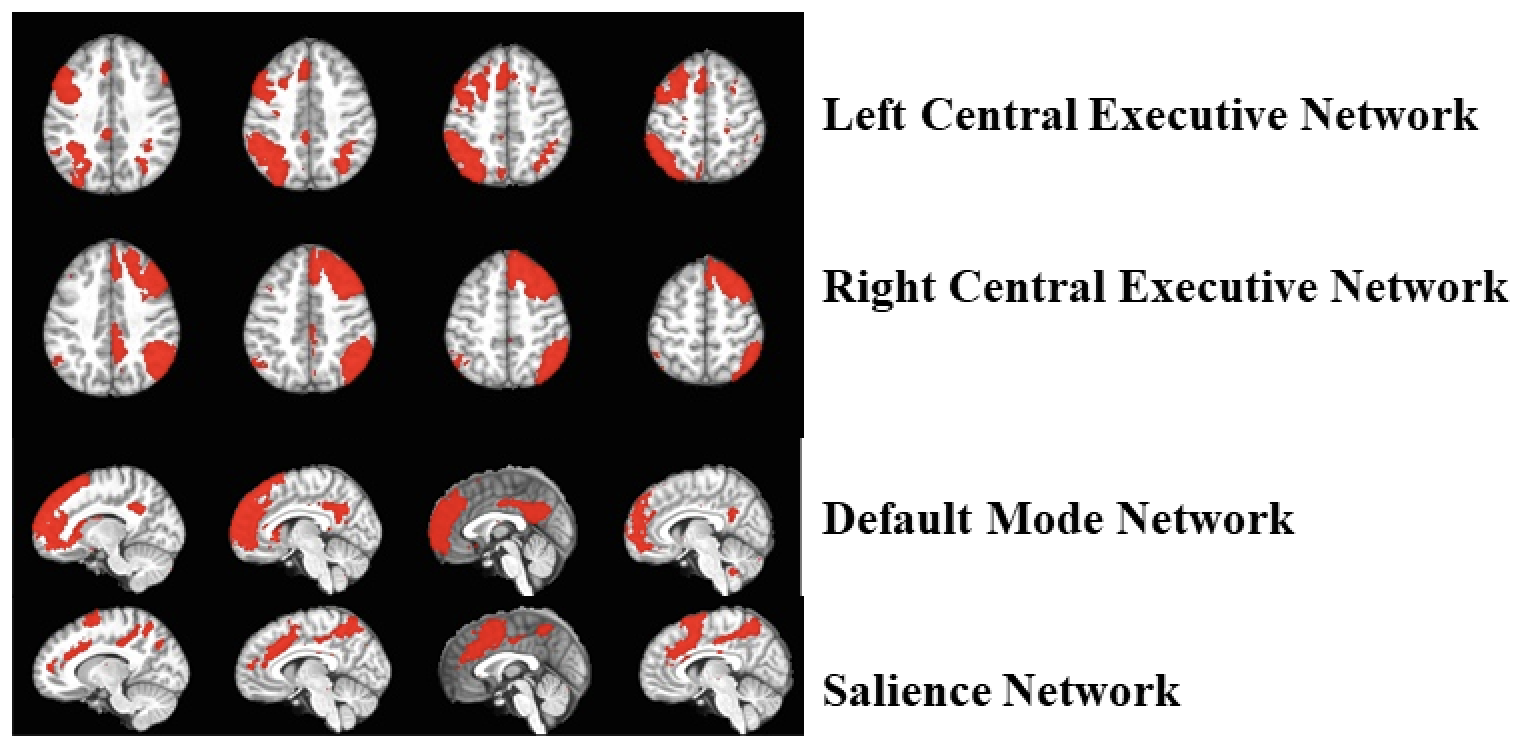


**Figure S2.** Visualization of intrinsic brain networks derived from group-based independent components analysis (see Methods in the main text for more details). Images in axial views (left and right central executive networks) are shown, from left to right, progressively more superior. Images in sagittal views (default mode and salience networks) are shown, from left to right, laterally to medially.

**Deviations from Preregistration**

***Social Communication***

Prior to viewing the data, we preregistered analyses that only included keyboard data from social communication apps, such as social media, text messaging, and email. This decision was based on prior research from our group [9]; however, we subsequently were informed of a technical change issued by Apple’s iOS 16 software upgrade wherein the function that allowed keystrokes to be labeled with the app in the foreground was discontinued with iOS 16 and will not be available in the future. Therefore, we wanted to use a methodology that will be useful in future studies. In addition, when we accessed the data, it was evident that the vast majority of apps from which the corpus of linguistic features were derived were from social communication apps (see **Figure S3**). As such, using all of the keyboard data enabled us to both increase the study’s power and examine our hypotheses at a more generalizable level for future studies. Further, given that we only conducted between-person analyses, we did not require a minimum of 3 days of data, as was stipulated in the original preregistration, which includes a description of within-person analyses that we plan to conduct in the future (see next section)..


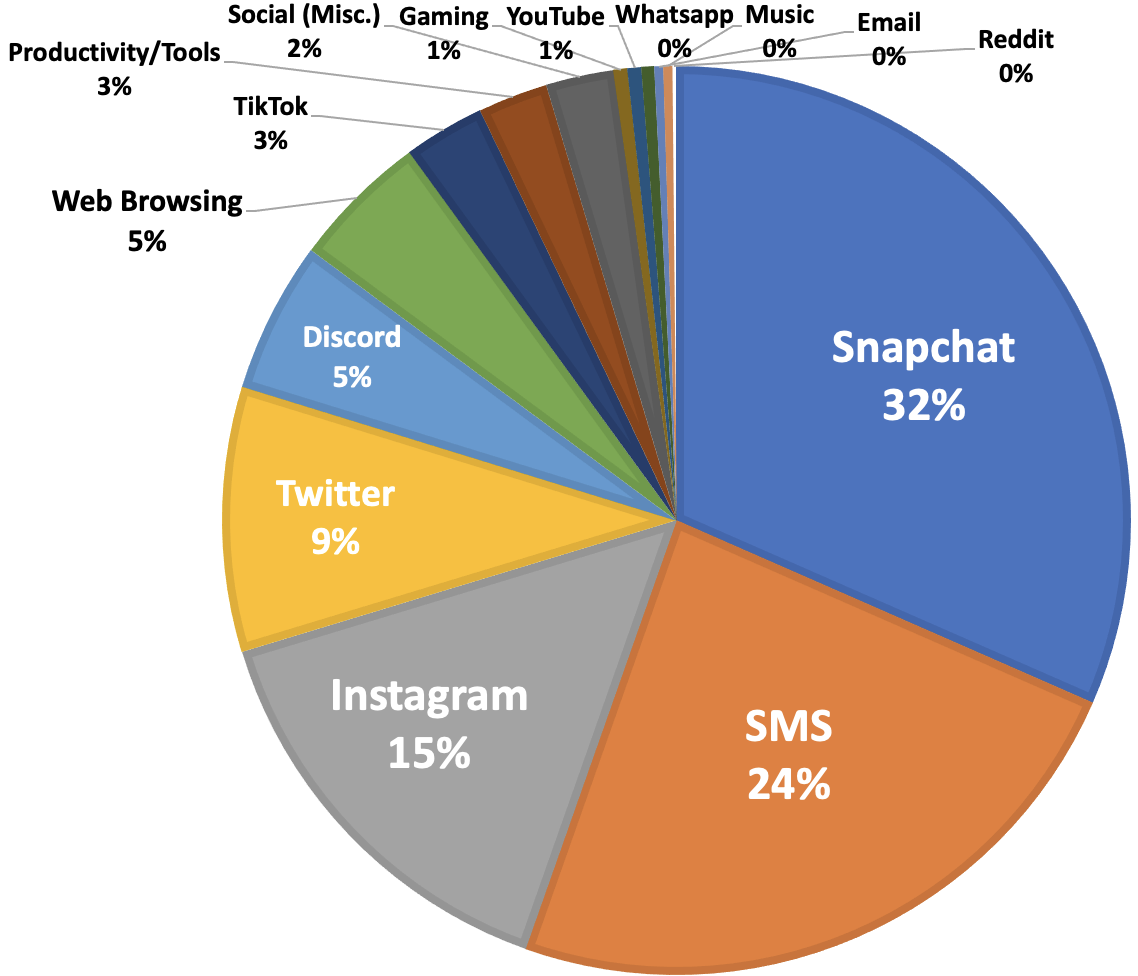


**Figure S3.** Pie chart depicting app-based percent of total words (99% of the data). Remaining 1% of data consisted of apps with <0% of total words each.

***Sensitivity Analysis of Pubertal Development***

We had preregistered that we would test whether our results would be robust to including pubertal development (as measured by the Tanner Stage Line Drawings). However, there was a limited range of pubertal development in the sample, as only adolescents with a Tanner Stage of ≥ 3 were included (see inclusion criteria, **Fig S1**). For this reason, we did not explore pubertal development as a covariate.

***Within-person Analysis of Daily Affect and Mood***

We had preregistered within-person analyses of the associations between ecological momentary assessments of daily affect and daily mood with linguistic features of smartphone use. We are planning to conduct these analyses in the future. Changes in daily affect and mood are likely correlated with depression diagnosis and symptoms, yet within-person changes in these outcomes involve a different analysis approach. For consistency within the methods, we focused the present study on depression diagnosis and symptoms at the between-person level.

**Supplemental Results**

**Sensitivity Analyses**

We conducted sensitivity analyses testing additional covariates that theoretically could impact our results. The results are summarized below. The accompanying statistics and analysis code are presented within Supplement 3 according to the model numbers noted below.

***Age and Sex***

Sensitivity analyses including age and sex as covariates were conducted on all significant models to test whether our results were robust to any age- or sex-related association with our outcomes of interest. Our results did not change when including age or sex in any model. Age improved the model fit of three associations: 1) depression and future focus words, 2) depressive symptoms and future focus words, and 3) depression and DMN within-network connectivity and future focus words (see Supplement 3, models 1f.2, S1f.2, and 2c.1b, respectively). Sex neither improved model fits nor was significantly associated with any depression-related linguistic features.

***Gender***

Given the gender diversity in our sample, we also tested whether our findings were robust to including gender identity in the model. The association between depression and future focus words (model 1f.1) became marginal (*p* = .057) when gender was included in the model; however, gender was not associated with future focus words (*p* = .66). All other results remained when gender was in the model.

***Number of Daily Messages***

The present study’s outcomes of interest are daily proportions of linguistic features relative to the total words each participant enters into their keyboard. While this controls for between-person variability in quantity of daily word use, it is nonetheless possible that these associations covary with the number of daily messages sent. As such, we tested the number of daily messages as a covariate. All findings were robust to including the number of daily messages (Supplement 3). For one model in particular – the association between depressive symptoms and first-person pronouns (model S1a.6) – including the number of daily messages improved the model fit, χ^2^(1, *N* = 40) = 10.67, *p* < .01. Number of daily messages was associated with a higher daily proportion of first-person pronouns (S1a.6; β = .09, 95% CI [.03, .14]) above and beyond the effect of depressive symptoms (S1a.6; β = .17, 95% CI [.05, .29]).

***COVID-19-related Covariates***

The present study spanned data collection that began before the COVID-19 pandemic and continued through the local stay-at-home order in the Bay Area (in effect on March 16, 2020). As such, we tested the robustness of our findings when two COVID-19-related covariates were tested: 1) a dichotomous variable indicating whether the participant entered the study after the COVID-19 pandemic began, 2) a continuous variable of the days between the local stay-at-home order (March 16, 2020) and the date when the EARS keyboard data (i.e., linguistic features) collection began. When controlling for whether the data was collected during COVID, the associations between depression (model 1f.5) and depressive symptoms (S1f.5) and future focus words became marginal (*p*=.10 and *p*=.08, respectively). Similarly, these associations (models 1f.8, S1f.8) became marginal when the days since the stay-at-home order went into effect (*p*=.10 and *p*=.08, respectively). Nonetheless, the associations between depression and first-person pronouns and negative emotions words were robust to these COVID-related covariates.

***Days between resting-state fMRI and EARS data collection***

Participants varied in the time between their resting-state fMRI scan and when they downloaded the EARS app, as the addition of the EARS app began after the larger longitudinal study was in progress. As such, we wanted to test whether our results were robust to this variability in timing. Only one association became marginal (*p* = .057) – the association between DMN within-network connectivity and future focus words (model 2c.1e). All other results remained significant when taking into account the difference in time between the resting-state fMRI and the EARS download.

**Depression Medication Status**

Participants within the MDD group varied as to whether they were taking a prescription medication to treat their depression. As such, we conducted a sensitivity analysis to test whether the inclusion of depression medication status as a dichotomous variable (0=No; 1=Yes) affected our results. All of our results remained when including depression medication status. In one case, medication status had a positive association with future-focus words in the model including Group, but not depressive symptoms (p=.03; see model 1f.9). Including medication status passed the likelihood ratio test when comparing the models χ^2^(1, *N* = 40) = 5.31, *p=* .02, but did not have a lower BIC. The direction and significance of the main effect of MDD on future-focus words did not change when including depression medication status.
