# Supplementary material for: Neural correlates of depression-related smartphone language use in adolescents
Source: NPP Digit Psychiatry Neurosci. 2024 Jul 9;2:11. doi: 10.1038/s44277-024-00009-6 (PMC12592214; doi:10.1038/s44277-024-00009-6)
Supplement: Supplementary file 2 — Supplement 2 [file 44277_2024_9_MOESM2_ESM.docx]

Supplement 2: Primary Results

Neural Correlates of Depression-Related Smartphone Language Use in Adolescents

Table of Contents

[1. AIM 1: What are the linguistic features of smartphone use that are associated with depression? 4](#_Toc164094215)

[1a. MDD - First-Person Pronouns 4](#_Toc164094216)

[S1a. Dep Sx - First-person pronouns 5](#_Toc164094217)

[1b. MDD - Positive Emotion Words 6](#_Toc164094218)

[S1b. Dep Sx - Positive Emotion Words 7](#_Toc164094219)

[1c. MDD - Negative Emotion Words 8](#_Toc164094220)

[S1c. Dep Sx - Negative Emotion Words 9](#_Toc164094221)

[1d. MDD - Past Focus Words 9](#_Toc164094222)

[S1d. Dep Sx - Past Focus Words 10](#_Toc164094223)

[1e. MDD - Present Focus Words 11](#_Toc164094224)

[S1e. Dep Sx - Present Focus Words 12](#_Toc164094225)

[1f. MDD - Future Focus Words 13](#_Toc164094226)

[S1f. Dep Sx - Future Focus Words 14](#_Toc164094227)

[1g. MDD - Word Count 15](#_Toc164094228)

[S1g. Dep Sx - Word Count 16](#_Toc164094229)

[Aim 1 Adjusted P-values 17](#_Toc164094230)

[2. AIM 2 - Which intrinsic connectivity networks are associated with depression-related linguistic features of smartphone use? 18](#_Toc164094231)

[S2a. First-person pronouns (controlling for depressive symptoms) 18](#_Toc164094232)

[S2a.1 Within-Network aDMN 18](#_Toc164094233)

[S2a.2 Within-Network leftCEN_Z 19](#_Toc164094234)

[S2a.3 Within-Network rightCEN_Z 20](#_Toc164094235)

[S2a.4 Within-Network SN_CO_Z 21](#_Toc164094236)

[S2a.5 Between-Network admn_lcen 22](#_Toc164094237)

[S2a.6 Between-Network admn_rcen 23](#_Toc164094238)

[S2a.7 Between-Network admn_sn 24](#_Toc164094239)

[S2a.8 Between-Network sn_lcen 25](#_Toc164094240)

[S2a.9 Between-Network sn_rcen 26](#_Toc164094241)

[2b. Negative Emotion Words (controlling for Group) 27](#_Toc164094242)

[2b.1 Within-Network aDMN 27](#_Toc164094243)

[2b.2 Within-Network leftCEN_Z 28](#_Toc164094244)

[2b.3 Within-Network rightCEN_Z 29](#_Toc164094245)

[2b.4 Within-Network SN_CO_Z 30](#_Toc164094246)

[2b.5 Between-Network admn_lcen 31](#_Toc164094247)

[2b.6 Between-Network admn_rcen 32](#_Toc164094248)

[2b.7 Between-Network admn_sn 33](#_Toc164094249)

[2b.8 Between-Network sn_lcen 34](#_Toc164094250)

[2b.9 Between-Network sn_rcen 35](#_Toc164094251)

[S2b. Negative Emotion Words (controlling for depressive symptoms) 36](#_Toc164094252)

[S2b.1 Within-Network aDMN 36](#_Toc164094253)

[S2b.2 Within-Network leftCEN_Z 37](#_Toc164094254)

[S2b.3 Within-Network rightCEN_Z 38](#_Toc164094255)

[S2b.4 Within-Network SN_CO_Z 39](#_Toc164094256)

[S2b.5 Between-Network admn_lcen 40](#_Toc164094257)

[S2b.6 Between-Network admn_rcen 41](#_Toc164094258)

[S2b.7 Between-Network admn_sn 42](#_Toc164094259)

[S2b.8 Between-Network sn_lcen 43](#_Toc164094260)

[S2b.9 Between-Network sn_rcen 44](#_Toc164094261)

[2c. Future focus words (controlling for Group) 45](#_Toc164094262)

[2c.1 Within-Network aDMN 45](#_Toc164094263)

[2c.2 Within-Network leftCEN_Z 46](#_Toc164094264)

[2c.3 Within-Network rightCEN_Z 47](#_Toc164094265)

[2c.4 Within-Network SN_CO_Z 48](#_Toc164094266)

[2c.5 Between-Network admn_lcen 49](#_Toc164094267)

[2c.6 Between-Network admn_rcen 50](#_Toc164094268)

[2c.7 Between-Network admn_sn 51](#_Toc164094269)

[2c.8 Between-Network sn_lcen 52](#_Toc164094270)

[2c.9 Between-Network sn_rcen 53](#_Toc164094271)

[S2c. Future Focus Words (controlling for depressive symptoms) 54](#_Toc164094272)

[S2c.1 Within-Network aDMN 54](#_Toc164094273)

[S2c.2 Within-Network leftCEN_Z 55](#_Toc164094274)

[S2c.3 Within-Network rightCEN_Z 56](#_Toc164094275)

[S2c.4 Within-Network SN_CO_Z 57](#_Toc164094276)

[S2c.5 Between-Network admn_lcen 58](#_Toc164094277)

[S2c.6 Between-Network admn_rcen 59](#_Toc164094278)

[S2c.7 Between-Network admn_sn 60](#_Toc164094279)

[S2c.8 Between-Network sn_lcen 61](#_Toc164094280)

[S2c.9 Between-Network sn_rcen 62](#_Toc164094281)

[Aim 2 Adjusted P-values 63](#_Toc164094282)

[3. AIM 3: Mediation: Does intrinsic network connectivity mediate the association between depression and depression-related linguistic features of smartphone use? 63](#_Toc164094283)

[3a. Dep Sx - leftCEN - First-person Pronouns 63](#_Toc164094284)

[3b. Dep Sx - aDMN - First-person Pronouns 68](#_Toc164094285)

[3c. MDD - SN - Negative Emotion Words 72](#_Toc164094286)

[3d. Dep Sx - SN - Negative Emotion Words 76](#_Toc164094287)

[3e. MDD - aDMN - Future-focus Words 80](#_Toc164094288)

[3f. Dep Sx - aDMN - Future-focus Words 84](#_Toc164094289)

[4. AIM 4 - Does intrinsic connectivity moderate the association between depression and depression-related linguistic features of smartphone use? 88](#_Toc164094290)

[S4a. Dep Sx - First-person pronouns 88](#_Toc164094291)

[S4a.1 Within-Network aDMN 88](#_Toc164094292)

[S4a.2 Within-Network leftCEN_Z 89](#_Toc164094293)

[S4a.3 Between-Network admn_lcen 90](#_Toc164094294)

[S4a.4 Between-Network admn_rcen 91](#_Toc164094295)

[S4a.5 Between-Network admn_sn 92](#_Toc164094296)

[S4a.6 Between-Network sn_lcen 93](#_Toc164094297)

[4b. MDD - Negative Emotion Words 95](#_Toc164094298)

[4b.1 Within-Network SN_CO_Z 95](#_Toc164094299)

[4b.2 Between-Network admn_sn 96](#_Toc164094300)

[4b.3 Between-Network sn_lcen 97](#_Toc164094301)

[4b.4 Between-Network sn_rcen 98](#_Toc164094302)

[S4b. Dep Sx - Negative Emotion Words 99](#_Toc164094303)

[S4b.1 Within-Network SN_CO_Z 99](#_Toc164094304)

[S4b.2 Between-Network admn_sn 100](#_Toc164094305)

[S4b.3 Between-Network sn_lcen 101](#_Toc164094306)

[S4b.4 Between-Network sn_rcen 102](#_Toc164094307)

[4c. MDD - Future focus words 103](#_Toc164094308)

[4c.1 Within-Network aDMN 103](#_Toc164094309)

[4c.2 Between-Network admn_lcen 104](#_Toc164094310)

[4c.3 Between-Network admn_rcen 105](#_Toc164094311)

[4c.4 Between-Network admn_sn 106](#_Toc164094312)

[S4c. Dep Sx - Future focus words 107](#_Toc164094313)

[S4c.1 Within-Network aDMN 107](#_Toc164094314)

[S4c.2 Between-Network admn_lcen 108](#_Toc164094315)

[S4c.3 Between-Network admn_rcen 109](#_Toc164094316)

[S4c.4 Between-Network admn_sn 110](#_Toc164094317)

# 1. AIM 1: What are the linguistic features of smartphone use that are associated with depression?

## 1a. MDD - First-Person Pronouns

#First-person pronouns (daily proportion of total words)
 model1a <- lmer(formula = i_day_prop ~
 Group
 + (1 |id_participant),
 data = df_aim1_all_language,
 na.action=na.exclude)
summary(model1a)

## Linear mixed model fit by REML. t-tests use Satterthwaite's method [
## lmerModLmerTest]
## Formula: i_day_prop ~ Group + (1 | id_participant)
## Data: df_aim1_all_language
##
## REML criterion at convergence: 11971.8
##
## Scaled residuals:
## Min 1Q Median 3Q Max
## -3.1316 -0.5786 -0.0171 0.5044 7.4673
##
## Random effects:
## Groups Name Variance Std.Dev.
## id_participant (Intercept) 2.25 1.500
## Residual 11.54 3.397
## Number of obs: 2251, groups: id_participant, 40
##
## Fixed effects:
## Estimate Std. Error df t value Pr(>|t|)
## (Intercept) 7.4851 0.4812 36.2583 15.556 <2e-16 ***
## GroupMDD 0.9357 0.5791 34.9761 1.616 0.115
## ---
## Signif. codes: 0 '***' 0.001 '**' 0.01 '*' 0.05 '.' 0.1 ' ' 1
##
## Correlation of Fixed Effects:
## (Intr)
## GroupMDD -0.831

standardize_parameters(model1a)

## # Standardization method: refit
##
## Parameter | Std. Coef. | 95% CI
## ----------------------------------------
## (Intercept) | -0.19 | [-0.44, 0.07]
## GroupMDD | 0.25 | [-0.05, 0.56]

### S1a. Dep Sx - First-person pronouns

#First-person pronouns (daily proportion of total words)
 model.S1a <- lmer(formula = i_day_prop ~
 RADS_total
 + (1 |id_participant),
 data = df_aim1_all_language,
 na.action=na.exclude)
summary(model.S1a)

## Linear mixed model fit by REML. t-tests use Satterthwaite's method [
## lmerModLmerTest]
## Formula: i_day_prop ~ RADS_total + (1 | id_participant)
## Data: df_aim1_all_language
##
## REML criterion at convergence: 11974.2
##
## Scaled residuals:
## Min 1Q Median 3Q Max
## -3.1166 -0.5743 -0.0162 0.5058 7.4826
##
## Random effects:
## Groups Name Variance Std.Dev.
## id_participant (Intercept) 1.889 1.374
## Residual 11.538 3.397
## Number of obs: 2251, groups: id_participant, 40
##
## Fixed effects:
## Estimate Std. Error df t value Pr(>|t|)
## (Intercept) 5.50537 0.93401 32.77152 5.894 1.35e-06 ***
## RADS_total 0.03680 0.01256 32.15868 2.929 0.00621 **
## ---
## Signif. codes: 0 '***' 0.001 '**' 0.01 '*' 0.05 '.' 0.1 ' ' 1
##
## Correlation of Fixed Effects:
## (Intr)
## RADS_total -0.964

standardize_parameters(model.S1a)

## # Standardization method: refit
##
## Parameter | Std. Coef. | 95% CI
## ----------------------------------------
## (Intercept) | 0.01 | [-0.12, 0.14]
## RADS_total | 0.19 | [ 0.06, 0.31]

## 1b. MDD - Positive Emotion Words

#Positive Emotion Words (daily proportion of total words)
model1b <- lmer(formula = posemo_day_prop ~
 Group
 + (1 |id_participant),
 data = df_aim1_all_language,
 na.action=na.exclude)
summary(model1b)

## Linear mixed model fit by REML. t-tests use Satterthwaite's method [
## lmerModLmerTest]
## Formula: posemo_day_prop ~ Group + (1 | id_participant)
## Data: df_aim1_all_language
##
## REML criterion at convergence: 10141.2
##
## Scaled residuals:
## Min 1Q Median 3Q Max
## -2.8983 -0.5469 -0.0867 0.4166 8.8687
##
## Random effects:
## Groups Name Variance Std.Dev.
## id_participant (Intercept) 1.464 1.210
## Residual 5.086 2.255
## Number of obs: 2251, groups: id_participant, 40
##
## Fixed effects:
## Estimate Std. Error df t value Pr(>|t|)
## (Intercept) 4.64284 0.37513 41.55894 12.377 1.59e-15 ***
## GroupMDD -0.05063 0.45288 39.97841 -0.112 0.912
## ---
## Signif. codes: 0 '***' 0.001 '**' 0.01 '*' 0.05 '.' 0.1 ' ' 1
##
## Correlation of Fixed Effects:
## (Intr)
## GroupMDD -0.828

standardize_parameters(model1b)

## # Standardization method: refit
##
## Parameter | Std. Coef. | 95% CI
## ----------------------------------------
## (Intercept) | 0.07 | [-0.21, 0.35]
## GroupMDD | -0.02 | [-0.36, 0.32]

### S1b. Dep Sx - Positive Emotion Words

#Positive Emotion Words (daily proportion of total words)
model.S1b <- lmer(formula = posemo_day_prop ~
 RADS_total
 + (1 |id_participant),
 data = df_aim1_all_language,
 na.action=na.exclude)
summary(model.S1b)

## Linear mixed model fit by REML. t-tests use Satterthwaite's method [
## lmerModLmerTest]
## Formula: posemo_day_prop ~ RADS_total + (1 | id_participant)
## Data: df_aim1_all_language
##
## REML criterion at convergence: 10148.6
##
## Scaled residuals:
## Min 1Q Median 3Q Max
## -2.8995 -0.5458 -0.0874 0.4149 8.8640
##
## Random effects:
## Groups Name Variance Std.Dev.
## id_participant (Intercept) 1.460 1.208
## Residual 5.086 2.255
## Number of obs: 2251, groups: id_participant, 40
##
## Fixed effects:
## Estimate Std. Error df t value Pr(>|t|)
## (Intercept) 4.285451 0.788075 38.417850 5.438 3.26e-06 ***
## RADS_total 0.004517 0.010629 37.482755 0.425 0.673
## ---
## Signif. codes: 0 '***' 0.001 '**' 0.01 '*' 0.05 '.' 0.1 ' ' 1
##
## Correlation of Fixed Effects:
## (Intr)
## RADS_total -0.964

standardize_parameters(model.S1b)

## # Standardization method: refit
##
## Parameter | Std. Coef. | 95% CI
## ----------------------------------------
## (Intercept) | 0.06 | [-0.09, 0.22]
## RADS_total | 0.03 | [-0.12, 0.18]

## 1c. MDD - Negative Emotion Words

#Negative Emotion Words (daily proportion of total words)
 model1c <- lmer(formula = negemo_day_prop ~
 Group
 + (1 |id_participant),
 data = df_aim1_all_language,
 na.action=na.exclude)
summary(model1c)

## Linear mixed model fit by REML. t-tests use Satterthwaite's method [
## lmerModLmerTest]
## Formula: negemo_day_prop ~ Group + (1 | id_participant)
## Data: df_aim1_all_language
##
## REML criterion at convergence: 8201.2
##
## Scaled residuals:
## Min 1Q Median 3Q Max
## -2.1937 -0.5804 -0.0889 0.3796 9.9977
##
## Random effects:
## Groups Name Variance Std.Dev.
## id_participant (Intercept) 0.2125 0.4609
## Residual 2.1765 1.4753
## Number of obs: 2251, groups: id_participant, 40
##
## Fixed effects:
## Estimate Std. Error df t value Pr(>|t|)
## (Intercept) 1.5991 0.1594 37.8636 10.031 3.26e-12 ***
## GroupMDD 0.9111 0.1910 36.5820 4.771 2.93e-05 ***
## ---
## Signif. codes: 0 '***' 0.001 '**' 0.01 '*' 0.05 '.' 0.1 ' ' 1
##
## Correlation of Fixed Effects:
## (Intr)
## GroupMDD -0.835

standardize_parameters(model1c)

## # Standardization method: refit
##
## Parameter | Std. Coef. | 95% CI
## -----------------------------------------
## (Intercept) | -0.39 | [-0.59, -0.20]
## GroupMDD | 0.58 | [ 0.34, 0.82]

### S1c. Dep Sx - Negative Emotion Words

#Negative Emotion Words (daily proportion of total words)
 model.S1c <- lmer(formula = negemo_day_prop ~
 RADS_total
 + (1 |id_participant),
 data = df_aim1_all_language,
 na.action=na.exclude)
summary(model.S1c)

## Linear mixed model fit by REML. t-tests use Satterthwaite's method [
## lmerModLmerTest]
## Formula: negemo_day_prop ~ RADS_total + (1 | id_participant)
## Data: df_aim1_all_language
##
## REML criterion at convergence: 8212.6
##
## Scaled residuals:
## Min 1Q Median 3Q Max
## -2.2606 -0.5817 -0.0889 0.3822 9.9958
##
## Random effects:
## Groups Name Variance Std.Dev.
## id_participant (Intercept) 0.2343 0.484
## Residual 2.1778 1.476
## Number of obs: 2251, groups: id_participant, 40
##
## Fixed effects:
## Estimate Std. Error df t value Pr(>|t|)
## (Intercept) 0.853749 0.342013 33.425330 2.496 0.017653 *
## RADS_total 0.019230 0.004594 32.953465 4.186 0.000198 ***
## ---
## Signif. codes: 0 '***' 0.001 '**' 0.01 '*' 0.05 '.' 0.1 ' ' 1
##
## Correlation of Fixed Effects:
## (Intr)
## RADS_total -0.964

standardize_parameters(model.S1c)

## # Standardization method: refit
##
## Parameter | Std. Coef. | 95% CI
## ----------------------------------------
## (Intercept) | 0.03 | [-0.08, 0.15]
## RADS_total | 0.23 | [ 0.12, 0.34]

## 1d. MDD - Past Focus Words

#Past focus words (daily proportion of total words)
 model1d <- lmer(formula = focuspast_day_prop ~
 Group
 + (1 |id_participant),
 data = df_aim1_all_language,
 na.action=na.exclude)
summary(model1d)

## Linear mixed model fit by REML. t-tests use Satterthwaite's method [
## lmerModLmerTest]
## Formula: focuspast_day_prop ~ Group + (1 | id_participant)
## Data: df_aim1_all_language
##
## REML criterion at convergence: 8909.2
##
## Scaled residuals:
## Min 1Q Median 3Q Max
## -2.1862 -0.6134 -0.0773 0.4884 6.7088
##
## Random effects:
## Groups Name Variance Std.Dev.
## id_participant (Intercept) 0.3141 0.5605
## Residual 2.9791 1.7260
## Number of obs: 2251, groups: id_participant, 40
##
## Fixed effects:
## Estimate Std. Error df t value Pr(>|t|)
## (Intercept) 2.8797 0.1920 33.1836 15.00 2.42e-16 ***
## GroupMDD 0.1520 0.2301 32.0672 0.66 0.514
## ---
## Signif. codes: 0 '***' 0.001 '**' 0.01 '*' 0.05 '.' 0.1 ' ' 1
##
## Correlation of Fixed Effects:
## (Intr)
## GroupMDD -0.834

standardize_parameters(model1d)

## # Standardization method: refit
##
## Parameter | Std. Coef. | 95% CI
## ----------------------------------------
## (Intercept) | -0.08 | [-0.29, 0.13]
## GroupMDD | 0.08 | [-0.17, 0.33]

### S1d. Dep Sx - Past Focus Words

#Past focus words (daily proportion of total words)
 model.S1d <- lmer(formula = focuspast_day_prop ~
 RADS_total
 + (1 |id_participant),
 data = df_aim1_all_language,
 na.action=na.exclude)
summary(model.S1d)

## Linear mixed model fit by REML. t-tests use Satterthwaite's method [
## lmerModLmerTest]
## Formula: focuspast_day_prop ~ RADS_total + (1 | id_participant)
## Data: df_aim1_all_language
##
## REML criterion at convergence: 8916.9
##
## Scaled residuals:
## Min 1Q Median 3Q Max
## -2.1860 -0.6126 -0.0770 0.4897 6.7097
##
## Random effects:
## Groups Name Variance Std.Dev.
## id_participant (Intercept) 0.3186 0.5645
## Residual 2.9788 1.7259
## Number of obs: 2251, groups: id_participant, 40
##
## Fixed effects:
## Estimate Std. Error df t value Pr(>|t|)
## (Intercept) 2.761354 0.399080 30.334305 6.919 1.04e-07 ***
## RADS_total 0.003126 0.005360 29.907187 0.583 0.564
## ---
## Signif. codes: 0 '***' 0.001 '**' 0.01 '*' 0.05 '.' 0.1 ' ' 1
##
## Correlation of Fixed Effects:
## (Intr)
## RADS_total -0.964

standardize_parameters(model.S1d)

## # Standardization method: refit
##
## Parameter | Std. Coef. | 95% CI
## ----------------------------------------
## (Intercept) | -0.01 | [-0.13, 0.10]
## RADS_total | 0.03 | [-0.08, 0.14]

## 1e. MDD - Present Focus Words

#Present focus words (daily proportion of total words)
 model1e <- lmer(formula = focuspresent_day_prop ~
 Group
 + (1 |id_participant),
 data = df_aim1_all_language,
 na.action=na.exclude)
summary(model1e)

## Linear mixed model fit by REML. t-tests use Satterthwaite's method [
## lmerModLmerTest]
## Formula: focuspresent_day_prop ~ Group + (1 | id_participant)
## Data: df_aim1_all_language
##
## REML criterion at convergence: 11628.3
##
## Scaled residuals:
## Min 1Q Median 3Q Max
## -4.1510 -0.5102 0.0207 0.5153 5.4574
##
## Random effects:
## Groups Name Variance Std.Dev.
## id_participant (Intercept) 1.263 1.124
## Residual 9.958 3.156
## Number of obs: 2251, groups: id_participant, 40
##
## Fixed effects:
## Estimate Std. Error df t value Pr(>|t|)
## (Intercept) 11.4516 0.3768 37.3638 30.395 <2e-16 ***
## GroupMDD -0.2619 0.4521 36.1130 -0.579 0.566
## ---
## Signif. codes: 0 '***' 0.001 '**' 0.01 '*' 0.05 '.' 0.1 ' ' 1
##
## Correlation of Fixed Effects:
## (Intr)
## GroupMDD -0.833

standardize_parameters(model1e)

## # Standardization method: refit
##
## Parameter | Std. Coef. | 95% CI
## ----------------------------------------
## (Intercept) | 0.14 | [-0.08, 0.36]
## GroupMDD | -0.08 | [-0.34, 0.19]

### S1e. Dep Sx - Present Focus Words

#Present focus words (daily proportion of total words)
 model.S1e <- lmer(formula = focuspresent_day_prop ~
 RADS_total
 + (1 |id_participant),
 data = df_aim1_all_language,
 na.action=na.exclude)
summary(model.S1e)

## Linear mixed model fit by REML. t-tests use Satterthwaite's method [
## lmerModLmerTest]
## Formula: focuspresent_day_prop ~ RADS_total + (1 | id_participant)
## Data: df_aim1_all_language
##
## REML criterion at convergence: 11636.1
##
## Scaled residuals:
## Min 1Q Median 3Q Max
## -4.1548 -0.5108 0.0197 0.5151 5.4574
##
## Random effects:
## Groups Name Variance Std.Dev.
## id_participant (Intercept) 1.283 1.133
## Residual 9.958 3.156
## Number of obs: 2251, groups: id_participant, 40
##
## Fixed effects:
## Estimate Std. Error df t value Pr(>|t|)
## (Intercept) 11.495082 0.786181 34.474294 14.621 2.41e-16 ***
## RADS_total -0.003143 0.010566 33.923298 -0.297 0.768
## ---
## Signif. codes: 0 '***' 0.001 '**' 0.01 '*' 0.05 '.' 0.1 ' ' 1
##
## Correlation of Fixed Effects:
## (Intr)
## RADS_total -0.964

standardize_parameters(model.S1e)

## # Standardization method: refit
##
## Parameter | Std. Coef. | 95% CI
## ----------------------------------------
## (Intercept) | 0.08 | [-0.04, 0.21]
## RADS_total | -0.02 | [-0.13, 0.10]

## 1f. MDD - Future Focus Words

#Future focus words (daily proportion of total words)
 model1f <- lmer(formula = focusfuture_day_prop ~
 Group
 + (1 |id_participant),
 data = df_aim1_all_language,
 na.action=na.exclude)
summary(model1f)

## Linear mixed model fit by REML. t-tests use Satterthwaite's method [
## lmerModLmerTest]
## Formula: focusfuture_day_prop ~ Group + (1 | id_participant)
## Data: df_aim1_all_language
##
## REML criterion at convergence: 6913.1
##
## Scaled residuals:
## Min 1Q Median 3Q Max
## -1.9359 -0.6197 -0.0888 0.4275 8.0243
##
## Random effects:
## Groups Name Variance Std.Dev.
## id_participant (Intercept) 0.1233 0.3512
## Residual 1.2271 1.1077
## Number of obs: 2251, groups: id_participant, 40
##
## Fixed effects:
## Estimate Std. Error df t value Pr(>|t|)
## (Intercept) 1.7058 0.1210 28.4992 14.096 2.24e-14 ***
## GroupMDD -0.3166 0.1450 27.5340 -2.183 0.0377 *
## ---
## Signif. codes: 0 '***' 0.001 '**' 0.01 '*' 0.05 '.' 0.1 ' ' 1
##
## Correlation of Fixed Effects:
## (Intr)
## GroupMDD -0.835

standardize_parameters(model1f)

## # Standardization method: refit
##
## Parameter | Std. Coef. | 95% CI
## -----------------------------------------
## (Intercept) | 0.26 | [ 0.05, 0.46]
## GroupMDD | -0.27 | [-0.52, -0.03]

### S1f. Dep Sx - Future Focus Words

#Future focus words (daily proportion of total words)
 model.S1f <- lmer(formula = focusfuture_day_prop ~
 RADS_total
 + (1 |id_participant),
 data = df_aim1_all_language,
 na.action=na.exclude)
summary(model.S1f)

## Linear mixed model fit by REML. t-tests use Satterthwaite's method [
## lmerModLmerTest]
## Formula: focusfuture_day_prop ~ RADS_total + (1 | id_participant)
## Data: df_aim1_all_language
##
## REML criterion at convergence: 6920.1
##
## Scaled residuals:
## Min 1Q Median 3Q Max
## -1.9146 -0.6200 -0.0865 0.4261 8.0308
##
## Random effects:
## Groups Name Variance Std.Dev.
## id_participant (Intercept) 0.1243 0.3526
## Residual 1.2267 1.1076
## Number of obs: 2251, groups: id_participant, 40
##
## Fixed effects:
## Estimate Std. Error df t value Pr(>|t|)
## (Intercept) 2.041092 0.250683 26.441813 8.142 1.12e-08 ***
## RADS_total -0.007745 0.003367 26.082358 -2.300 0.0297 *
## ---
## Signif. codes: 0 '***' 0.001 '**' 0.01 '*' 0.05 '.' 0.1 ' ' 1
##
## Correlation of Fixed Effects:
## (Intr)
## RADS_total -0.964

standardize_parameters(model.S1f)

## # Standardization method: refit
##
## Parameter | Std. Coef. | 95% CI
## -----------------------------------------
## (Intercept) | 0.06 | [-0.06, 0.17]
## RADS_total | -0.13 | [-0.23, -0.02]

## 1g. MDD - Word Count

#Word count (daily)
 model1g <- lmer(formula = WC_day ~
 Group
 + (1 |id_participant),
 data = df_aim1_all_language,
 na.action=na.exclude)
summary(model1g)

## Linear mixed model fit by REML. t-tests use Satterthwaite's method [
## lmerModLmerTest]
## Formula: WC_day ~ Group + (1 | id_participant)
## Data: df_aim1_all_language
##
## REML criterion at convergence: 35391.3
##
## Scaled residuals:
## Min 1Q Median 3Q Max
## -3.9940 -0.2851 -0.0918 0.1453 11.1707
##
## Random effects:
## Groups Name Variance Std.Dev.
## id_participant (Intercept) 243747 493.7
## Residual 377718 614.6
## Number of obs: 2251, groups: id_participant, 40
##
## Fixed effects:
## Estimate Std. Error df t value Pr(>|t|)
## (Intercept) 220.80 144.77 43.98 1.525 0.1344
## GroupMDD 322.65 176.03 42.19 1.833 0.0739 .
## ---
## Signif. codes: 0 '***' 0.001 '**' 0.01 '*' 0.05 '.' 0.1 ' ' 1
##
## Correlation of Fixed Effects:
## (Intr)
## GroupMDD -0.822

standardize_parameters(model1g)

## # Standardization method: refit
##
## Parameter | Std. Coef. | 95% CI
## -----------------------------------------
## (Intercept) | -0.39 | [-0.72, -0.05]
## GroupMDD | 0.38 | [-0.03, 0.78]

### S1g. Dep Sx - Word Count

#Word count (daily)
 model.S1g <- lmer(formula = WC_day ~
 RADS_total
 + (1 |id_participant),
 data = df_aim1_all_language,
 na.action=na.exclude)
summary(model.S1g)

## Linear mixed model fit by REML. t-tests use Satterthwaite's method [
## lmerModLmerTest]
## Formula: WC_day ~ RADS_total + (1 | id_participant)
## Data: df_aim1_all_language
##
## REML criterion at convergence: 35398.8
##
## Scaled residuals:
## Min 1Q Median 3Q Max
## -3.9968 -0.2855 -0.0921 0.1486 11.1682
##
## Random effects:
## Groups Name Variance Std.Dev.
## id_participant (Intercept) 244447 494.4
## Residual 377702 614.6
## Number of obs: 2251, groups: id_participant, 40
##
## Fixed effects:
## Estimate Std. Error df t value Pr(>|t|)
## (Intercept) -105.208 308.755 41.271 -0.341 0.7350
## RADS_total 7.654 4.185 40.159 1.829 0.0748 .
## ---
## Signif. codes: 0 '***' 0.001 '**' 0.01 '*' 0.05 '.' 0.1 ' ' 1
##
## Correlation of Fixed Effects:
## (Intr)
## RADS_total -0.964

standardize_parameters(model.S1g)

## # Standardization method: refit
##
## Parameter | Std. Coef. | 95% CI
## ----------------------------------------
## (Intercept) | -0.11 | [-0.30, 0.08]
## RADS_total | 0.17 | [-0.01, 0.35]

## Aim 1 Adjusted P-values

#Correct for multiple comparisons between depression (MDD) and depressive symptoms

#First-person pronouns
fpp_pvalues <- c(.115, .006)
p.adjust(fpp_pvalues,method="fdr")

## [1] 0.115 0.012

#Positive Emotion Words
pos_pvalues <- c(.912, .673)
p.adjust(pos_pvalues,method="fdr")

## [1] 0.912 0.912

#Negative Emotion Words
neg_pvalues <- c(.0000293, 0.000198)
p.adjust(neg_pvalues,method="fdr")

## [1] 5.86e-05 1.98e-04

#Past Focus Words
past_pvalues <- c(.514, .564)
p.adjust(past_pvalues,method="fdr")

## [1] 0.564 0.564

#Present Focus Words
present_pvalues <- c(.566, .768)
p.adjust(present_pvalues,method="fdr")

## [1] 0.768 0.768

#Future Focus Words
future_pvalues <- c(.023, .043)
p.adjust(future_pvalues,method="fdr")

## [1] 0.043 0.043

#Daily Word Count
word_count_pvalues <- c(0.0739, 0.0748)
p.adjust(word_count_pvalues,method="fdr")

## [1] 0.0748 0.0748

# 2. AIM 2 - Which intrinsic connectivity networks are associated with depression-related linguistic features of smartphone use?

## S2a. First-person pronouns (controlling for depressive symptoms)

### S2a.1 Within-Network aDMN

modelS2a.1 <- lmer(formula = i_day_prop ~
 aDMN_Z + RADS_total
 + (1 |id_participant) ,
 data = df_aim1_all_language,
 na.action=na.exclude)
summary(modelS2a.1)

## Linear mixed model fit by REML. t-tests use Satterthwaite's method [
## lmerModLmerTest]
## Formula: i_day_prop ~ aDMN_Z + RADS_total + (1 | id_participant)
## Data: df_aim1_all_language
##
## REML criterion at convergence: 10984.5
##
## Scaled residuals:
## Min 1Q Median 3Q Max
## -3.1295 -0.5621 -0.0136 0.5006 7.5236
##
## Random effects:
## Groups Name Variance Std.Dev.
## id_participant (Intercept) 1.817 1.348
## Residual 11.416 3.379
## Number of obs: 2070, groups: id_participant, 34
##
## Fixed effects:
## Estimate Std. Error df t value Pr(>|t|)
## (Intercept) 2.10012 1.70117 34.41917 1.235 0.22537
## aDMN_Z 1.69478 0.78764 36.97581 2.152 0.03802 *
## RADS_total 0.04799 0.01353 28.89800 3.547 0.00135 **
## ---
## Signif. codes: 0 '***' 0.001 '**' 0.01 '*' 0.05 '.' 0.1 ' ' 1
##
## Correlation of Fixed Effects:
## (Intr) aDMN_Z
## aDMN_Z -0.808
## RADS_total -0.712 0.190

standardize_parameters(modelS2a.1)

## # Standardization method: refit
##
## Parameter | Std. Coef. | 95% CI
## ----------------------------------------
## (Intercept) | 0.01 | [-0.12, 0.15]
## aDMN_Z | 0.12 | [ 0.01, 0.23]
## RADS_total | 0.24 | [ 0.11, 0.38]

### S2a.2 Within-Network leftCEN_Z

modelS2a.2 <- lmer(formula = i_day_prop ~
 leftCEN_Z + RADS_total
 + (1 |id_participant) ,
 data = df_aim1_all_language,
 na.action=na.exclude)
summary(modelS2a.2)

## Linear mixed model fit by REML. t-tests use Satterthwaite's method [
## lmerModLmerTest]
## Formula: i_day_prop ~ leftCEN_Z + RADS_total + (1 | id_participant)
## Data: df_aim1_all_language
##
## REML criterion at convergence: 10982.9
##
## Scaled residuals:
## Min 1Q Median 3Q Max
## -3.0881 -0.5685 -0.0138 0.5009 7.5146
##
## Random effects:
## Groups Name Variance Std.Dev.
## id_participant (Intercept) 1.731 1.316
## Residual 11.415 3.379
## Number of obs: 2070, groups: id_participant, 34
##
## Fixed effects:
## Estimate Std. Error df t value Pr(>|t|)
## (Intercept) -0.57178 2.49014 37.69776 -0.230 0.819631
## leftCEN_Z 2.23640 0.90764 35.05626 2.464 0.018789 *
## RADS_total 0.06149 0.01515 33.46429 4.059 0.000279 ***
## ---
## Signif. codes: 0 '***' 0.001 '**' 0.01 '*' 0.05 '.' 0.1 ' ' 1
##
## Correlation of Fixed Effects:
## (Intr) lCEN_Z
## leftCEN_Z -0.919
## RADS_total -0.797 0.512

standardize_parameters(modelS2a.2)

## # Standardization method: refit
##
## Parameter | Std. Coef. | 95% CI
## ----------------------------------------
## (Intercept) | 0.01 | [-0.13, 0.15]
## leftCEN_Z | 0.19 | [ 0.04, 0.34]
## RADS_total | 0.31 | [ 0.16, 0.46]

### S2a.3 Within-Network rightCEN_Z

modelS2a.3 <- lmer(formula = i_day_prop ~
 rightCEN_Z + RADS_total
 + (1 |id_participant) ,
 data = df_aim1_all_language,
 na.action=na.exclude)
summary(modelS2a.3)

## Linear mixed model fit by REML. t-tests use Satterthwaite's method [
## lmerModLmerTest]
## Formula: i_day_prop ~ rightCEN_Z + RADS_total + (1 | id_participant)
## Data: df_aim1_all_language
##
## REML criterion at convergence: 10986.4
##
## Scaled residuals:
## Min 1Q Median 3Q Max
## -3.1520 -0.5687 -0.0126 0.5035 7.5249
##
## Random effects:
## Groups Name Variance Std.Dev.
## id_participant (Intercept) 1.808 1.345
## Residual 11.424 3.380
## Number of obs: 2070, groups: id_participant, 34
##
## Fixed effects:
## Estimate Std. Error df t value Pr(>|t|)
## (Intercept) 8.13223 1.99510 26.52896 4.076 0.000372 ***
## rightCEN_Z -1.14253 0.64191 26.51564 -1.780 0.086558 .
## RADS_total 0.03305 0.01427 25.70156 2.316 0.028796 *
## ---
## Signif. codes: 0 '***' 0.001 '**' 0.01 '*' 0.05 '.' 0.1 ' ' 1
##
## Correlation of Fixed Effects:
## (Intr) rCEN_Z
## rightCEN_Z -0.865
## RADS_total -0.770 0.370

standardize_parameters(modelS2a.3)

## # Standardization method: refit
##
## Parameter | Std. Coef. | 95% CI
## ----------------------------------------
## (Intercept) | -0.02 | [-0.16, 0.13]
## rightCEN_Z | -0.17 | [-0.36, 0.02]
## RADS_total | 0.17 | [ 0.03, 0.31]

### S2a.4 Within-Network SN_CO_Z

modelS2a.4 <- lmer(formula = i_day_prop ~
 SN_CO_Z + RADS_total
 + (1 |id_participant) ,
 data = df_aim1_all_language,
 na.action=na.exclude)
summary(modelS2a.4)

## Linear mixed model fit by REML. t-tests use Satterthwaite's method [
## lmerModLmerTest]
## Formula: i_day_prop ~ SN_CO_Z + RADS_total + (1 | id_participant)
## Data: df_aim1_all_language
##
## REML criterion at convergence: 10989.2
##
## Scaled residuals:
## Min 1Q Median 3Q Max
## -3.1387 -0.5670 -0.0158 0.5018 7.5217
##
## Random effects:
## Groups Name Variance Std.Dev.
## id_participant (Intercept) 2.084 1.443
## Residual 11.421 3.380
## Number of obs: 2070, groups: id_participant, 34
##
## Fixed effects:
## Estimate Std. Error df t value Pr(>|t|)
## (Intercept) 4.37056 2.26862 31.22637 1.927 0.06318 .
## SN_CO_Z 0.22314 0.67356 31.77715 0.331 0.74260
## RADS_total 0.04403 0.01466 27.96335 3.004 0.00557 **
## ---
## Signif. codes: 0 '***' 0.001 '**' 0.01 '*' 0.05 '.' 0.1 ' ' 1
##
## Correlation of Fixed Effects:
## (Intr) SN_CO_
## SN_CO_Z -0.884
## RADS_total -0.678 0.276

standardize_parameters(modelS2a.4)

## # Standardization method: refit
##
## Parameter | Std. Coef. | 95% CI
## ----------------------------------------
## (Intercept) | 7.25e-03 | [-0.14, 0.15]
## SN_CO_Z | 0.02 | [-0.10, 0.14]
## RADS_total | 0.22 | [ 0.08, 0.37]

### S2a.5 Between-Network admn_lcen

modelS2a.5 <- lmer(formula = i_day_prop ~
 admn_lcen + RADS_total
 + (1 |id_participant) ,
 data = df_aim1_all_language,
 na.action=na.exclude)
summary(modelS2a.5)

## Linear mixed model fit by REML. t-tests use Satterthwaite's method [
## lmerModLmerTest]
## Formula: i_day_prop ~ admn_lcen + RADS_total + (1 | id_participant)
## Data: df_aim1_all_language
##
## REML criterion at convergence: 10879.6
##
## Scaled residuals:
## Min 1Q Median 3Q Max
## -3.1659 -0.5682 -0.0119 0.5035 7.5631
##
## Random effects:
## Groups Name Variance Std.Dev.
## id_participant (Intercept) 2.127 1.459
## Residual 11.290 3.360
## Number of obs: 2054, groups: id_participant, 32
##
## Fixed effects:
## Estimate Std. Error df t value Pr(>|t|)
## (Intercept) 5.14784 1.08962 26.10727 4.724 6.89e-05 ***
## admn_lcen -0.02129 1.04517 27.36623 -0.020 0.98389
## RADS_total 0.04184 0.01463 25.44083 2.861 0.00833 **
## ---
## Signif. codes: 0 '***' 0.001 '**' 0.01 '*' 0.05 '.' 0.1 ' ' 1
##
## Correlation of Fixed Effects:
## (Intr) admn_l
## admn_lcen -0.115
## RADS_total -0.912 -0.213

standardize_parameters(modelS2a.5)

## # Standardization method: refit
##
## Parameter | Std. Coef. | 95% CI
## ----------------------------------------
## (Intercept) | 0.02 | [-0.14, 0.17]
## admn_lcen | -1.55e-03 | [-0.15, 0.15]
## RADS_total | 0.21 | [ 0.07, 0.36]

### S2a.6 Between-Network admn_rcen

modelS2a.6 <- lmer(formula = i_day_prop ~
 admn_rcen + RADS_total
 + (1 |id_participant) ,
 data = df_aim1_all_language,
 na.action=na.exclude)
summary(modelS2a.6)

## Linear mixed model fit by REML. t-tests use Satterthwaite's method [
## lmerModLmerTest]
## Formula: i_day_prop ~ admn_rcen + RADS_total + (1 | id_participant)
## Data: df_aim1_all_language
##
## REML criterion at convergence: 10021.4
##
## Scaled residuals:
## Min 1Q Median 3Q Max
## -3.3758 -0.5728 -0.0031 0.5271 4.6344
##
## Random effects:
## Groups Name Variance Std.Dev.
## id_participant (Intercept) 1.976 1.406
## Residual 9.943 3.153
## Number of obs: 1938, groups: id_participant, 31
##
## Fixed effects:
## Estimate Std. Error df t value Pr(>|t|)
## (Intercept) 4.32356 1.18501 25.97909 3.649 0.00116 **
## admn_rcen -0.41168 0.92929 22.66706 -0.443 0.66196
## RADS_total 0.05483 0.01555 24.82566 3.526 0.00167 **
## ---
## Signif. codes: 0 '***' 0.001 '**' 0.01 '*' 0.05 '.' 0.1 ' ' 1
##
## Correlation of Fixed Effects:
## (Intr) admn_r
## admn_rcen -0.215
## RADS_total -0.863 -0.252

standardize_parameters(modelS2a.6)

## # Standardization method: refit
##
## Parameter | Std. Coef. | 95% CI
## ----------------------------------------
## (Intercept) | 0.04 | [-0.12, 0.20]
## admn_rcen | -0.04 | [-0.21, 0.13]
## RADS_total | 0.25 | [ 0.11, 0.39]

### S2a.7 Between-Network admn_sn

modelS2a.7 <- lmer(formula = i_day_prop ~
 admn_sn + RADS_total
 + (1 |id_participant) ,
 data = df_aim1_all_language,
 na.action=na.exclude)
summary(modelS2a.7)

## Linear mixed model fit by REML. t-tests use Satterthwaite's method [
## lmerModLmerTest]
## Formula: i_day_prop ~ admn_sn + RADS_total + (1 | id_participant)
## Data: df_aim1_all_language
##
## REML criterion at convergence: 10021.7
##
## Scaled residuals:
## Min 1Q Median 3Q Max
## -3.3805 -0.5734 -0.0032 0.5266 4.6345
##
## Random effects:
## Groups Name Variance Std.Dev.
## id_participant (Intercept) 1.982 1.408
## Residual 9.944 3.153
## Number of obs: 1938, groups: id_participant, 31
##
## Fixed effects:
## Estimate Std. Error df t value Pr(>|t|)
## (Intercept) 4.20925 1.15882 25.76322 3.632 0.00122 **
## admn_sn -0.17255 0.89274 23.58796 -0.193 0.84840
## RADS_total 0.05362 0.01531 25.14071 3.502 0.00175 **
## ---
## Signif. codes: 0 '***' 0.001 '**' 0.01 '*' 0.05 '.' 0.1 ' ' 1
##
## Correlation of Fixed Effects:
## (Intr) admn_s
## admn_sn 0.006
## RADS_total -0.956 -0.177

standardize_parameters(modelS2a.7)

## # Standardization method: refit
##
## Parameter | Std. Coef. | 95% CI
## ----------------------------------------
## (Intercept) | 0.04 | [-0.11, 0.20]
## admn_sn | -0.01 | [-0.16, 0.13]
## RADS_total | 0.24 | [ 0.11, 0.38]

### S2a.8 Between-Network sn_lcen

modelS2a.8 <- lmer(formula = i_day_prop ~
 sn_lcen + RADS_total
 + (1 |id_participant) ,
 data = df_aim1_all_language,
 na.action=na.exclude)
summary(modelS2a.8)

## Linear mixed model fit by REML. t-tests use Satterthwaite's method [
## lmerModLmerTest]
## Formula: i_day_prop ~ sn_lcen + RADS_total + (1 | id_participant)
## Data: df_aim1_all_language
##
## REML criterion at convergence: 10987.7
##
## Scaled residuals:
## Min 1Q Median 3Q Max
## -3.1253 -0.5682 -0.0114 0.5024 7.5203
##
## Random effects:
## Groups Name Variance Std.Dev.
## id_participant (Intercept) 2.032 1.425
## Residual 11.422 3.380
## Number of obs: 2070, groups: id_participant, 34
##
## Fixed effects:
## Estimate Std. Error df t value Pr(>|t|)
## (Intercept) 4.56827 1.21190 27.17765 3.769 0.000805 ***
## sn_lcen 0.87781 1.12589 27.75721 0.780 0.442193
## RADS_total 0.04459 0.01416 26.72064 3.149 0.004003 **
## ---
## Signif. codes: 0 '***' 0.001 '**' 0.01 '*' 0.05 '.' 0.1 ' ' 1
##
## Correlation of Fixed Effects:
## (Intr) sn_lcn
## sn_lcen -0.498
## RADS_total -0.912 0.176

standardize_parameters(modelS2a.8)

## # Standardization method: refit
##
## Parameter | Std. Coef. | 95% CI
## ----------------------------------------
## (Intercept) | 0.02 | [-0.13, 0.18]
## sn_lcen | 0.05 | [-0.08, 0.18]
## RADS_total | 0.23 | [ 0.09, 0.37]

### S2a.9 Between-Network sn_rcen

modelS2a.9 <- lmer(formula = i_day_prop ~
 sn_rcen + RADS_total
 + (1 |id_participant) ,
 data = df_aim1_all_language,
 na.action=na.exclude)
summary(modelS2a.9)

## Linear mixed model fit by REML. t-tests use Satterthwaite's method [
## lmerModLmerTest]
## Formula: i_day_prop ~ sn_rcen + RADS_total + (1 | id_participant)
## Data: df_aim1_all_language
##
## REML criterion at convergence: 10988.5
##
## Scaled residuals:
## Min 1Q Median 3Q Max
## -3.1448 -0.5667 -0.0151 0.5012 7.5216
##
## Random effects:
## Groups Name Variance Std.Dev.
## id_participant (Intercept) 2.098 1.448
## Residual 11.421 3.379
## Number of obs: 2070, groups: id_participant, 34
##
## Fixed effects:
## Estimate Std. Error df t value Pr(>|t|)
## (Intercept) 5.05306 1.13384 27.36062 4.457 0.000128 ***
## sn_rcen -0.05326 1.05639 26.79597 -0.050 0.960164
## RADS_total 0.04269 0.01413 27.14184 3.021 0.005440 **
## ---
## Signif. codes: 0 '***' 0.001 '**' 0.01 '*' 0.05 '.' 0.1 ' ' 1
##
## Correlation of Fixed Effects:
## (Intr) sn_rcn
## sn_rcen -0.342
## RADS_total -0.911 0.011

standardize_parameters(modelS2a.9)

## # Standardization method: refit
##
## Parameter | Std. Coef. | 95% CI
## ----------------------------------------
## (Intercept) | 8.31e-03 | [-0.14, 0.16]
## sn_rcen | -3.29e-03 | [-0.13, 0.12]
## RADS_total | 0.22 | [ 0.08, 0.36]

## 2b. Negative Emotion Words (controlling for Group)

### 2b.1 Within-Network aDMN

model2b.1 <- lmer(formula = negemo_day_prop ~
 aDMN_Z + Group +
 (1 |id_participant),
 data = df_aim1_all_language,
 na.action=na.exclude)
summary(model2b.1)

## Linear mixed model fit by REML. t-tests use Satterthwaite's method [
## lmerModLmerTest]
## Formula: negemo_day_prop ~ aDMN_Z + Group + (1 | id_participant)
## Data: df_aim1_all_language
##
## REML criterion at convergence: 7492
##
## Scaled residuals:
## Min 1Q Median 3Q Max
## -2.2415 -0.5755 -0.0861 0.3718 10.1195
##
## Random effects:
## Groups Name Variance Std.Dev.
## id_participant (Intercept) 0.2512 0.5012
## Residual 2.1220 1.4567
## Number of obs: 2070, groups: id_participant, 34
##
## Fixed effects:
## Estimate Std. Error df t value Pr(>|t|)
## (Intercept) 1.2590 0.5046 35.8043 2.495 0.017351 *
## aDMN_Z 0.1775 0.3003 37.1073 0.591 0.558032
## GroupMDD 0.9968 0.2228 30.1431 4.474 0.000101 ***
## ---
## Signif. codes: 0 '***' 0.001 '**' 0.01 '*' 0.05 '.' 0.1 ' ' 1
##
## Correlation of Fixed Effects:
## (Intr) aDMN_Z
## aDMN_Z -0.928
## GroupMDD -0.407 0.100

standardize_parameters(model2b.1)

## # Standardization method: refit
##
## Parameter | Std. Coef. | 95% CI
## -----------------------------------------
## (Intercept) | -0.45 | [-0.69, -0.21]
## aDMN_Z | 0.03 | [-0.07, 0.13]
## GroupMDD | 0.64 | [ 0.36, 0.92]

### 2b.2 Within-Network leftCEN_Z

model2b.2 <- lmer(formula = negemo_day_prop ~
 leftCEN_Z + Group +
 (1 |id_participant),
 data = df_aim1_all_language,
 na.action=na.exclude)
summary(model2b.2)

## Linear mixed model fit by REML. t-tests use Satterthwaite's method [
## lmerModLmerTest]
## Formula: negemo_day_prop ~ leftCEN_Z + Group + (1 | id_participant)
## Data: df_aim1_all_language
##
## REML criterion at convergence: 7490
##
## Scaled residuals:
## Min 1Q Median 3Q Max
## -2.2517 -0.5782 -0.0820 0.3731 10.1247
##
## Random effects:
## Groups Name Variance Std.Dev.
## id_participant (Intercept) 0.2447 0.4947
## Residual 2.1210 1.4564
## Number of obs: 2070, groups: id_participant, 34
##
## Fixed effects:
## Estimate Std. Error df t value Pr(>|t|)
## (Intercept) 0.4468 0.8074 37.1537 0.553 0.583
## leftCEN_Z 0.4981 0.3591 35.3001 1.387 0.174
## GroupMDD 1.1816 0.2621 35.4708 4.508 6.86e-05 ***
## ---
## Signif. codes: 0 '***' 0.001 '**' 0.01 '*' 0.05 '.' 0.1 ' ' 1
##
## Correlation of Fixed Effects:
## (Intr) lCEN_Z
## leftCEN_Z -0.973
## GroupMDD -0.696 0.547

standardize_parameters(model2b.2)

## # Standardization method: refit
##
## Parameter | Std. Coef. | 95% CI
## -----------------------------------------
## (Intercept) | -0.54 | [-0.81, -0.27]
## leftCEN_Z | 0.10 | [-0.04, 0.25]
## GroupMDD | 0.76 | [ 0.43, 1.09]

### 2b.3 Within-Network rightCEN_Z

model2b.3 <- lmer(formula = negemo_day_prop ~
 rightCEN_Z + Group
 +
 (1 |id_participant),
 data = df_aim1_all_language,
 na.action=na.exclude)
summary(model2b.3)

## Linear mixed model fit by REML. t-tests use Satterthwaite's method [
## lmerModLmerTest]
## Formula: negemo_day_prop ~ rightCEN_Z + Group + (1 | id_participant)
## Data: df_aim1_all_language
##
## REML criterion at convergence: 7490.9
##
## Scaled residuals:
## Min 1Q Median 3Q Max
## -2.2415 -0.5761 -0.0857 0.3763 10.1089
##
## Random effects:
## Groups Name Variance Std.Dev.
## id_participant (Intercept) 0.230 0.4796
## Residual 2.122 1.4568
## Number of obs: 2070, groups: id_participant, 34
##
## Fixed effects:
## Estimate Std. Error df t value Pr(>|t|)
## (Intercept) 2.2848 0.5579 26.3196 4.095 0.000358 ***
## rightCEN_Z -0.3249 0.2297 26.3235 -1.414 0.169013
## GroupMDD 0.8871 0.2239 28.0896 3.963 0.000462 ***
## ---
## Signif. codes: 0 '***' 0.001 '**' 0.01 '*' 0.05 '.' 0.1 ' ' 1
##
## Correlation of Fixed Effects:
## (Intr) rCEN_Z
## rightCEN_Z -0.946
## GroupMDD -0.541 0.293

standardize_parameters(model2b.3)

## # Standardization method: refit
##
## Parameter | Std. Coef. | 95% CI
## -----------------------------------------
## (Intercept) | -0.41 | [-0.64, -0.18]
## rightCEN_Z | -0.12 | [-0.28, 0.04]
## GroupMDD | 0.57 | [ 0.29, 0.85]

### 2b.4 Within-Network SN_CO_Z

model2b.4 <- lmer(formula = negemo_day_prop ~
 SN_CO_Z + Group +
 (1 |id_participant),
 data = df_aim1_all_language,
 na.action=na.exclude)
summary(model2b.4)

## Linear mixed model fit by REML. t-tests use Satterthwaite's method [
## lmerModLmerTest]
## Formula: negemo_day_prop ~ SN_CO_Z + Group + (1 | id_participant)
## Data: df_aim1_all_language
##
## REML criterion at convergence: 7488.2
##
## Scaled residuals:
## Min 1Q Median 3Q Max
## -2.2065 -0.5776 -0.0863 0.3760 10.1242
##
## Random effects:
## Groups Name Variance Std.Dev.
## id_participant (Intercept) 0.2013 0.4486
## Residual 2.1227 1.4570
## Number of obs: 2070, groups: id_participant, 34
##
## Fixed effects:
## Estimate Std. Error df t value Pr(>|t|)
## (Intercept) 0.2343 0.6074 31.4548 0.386 0.7023
## SN_CO_Z 0.4938 0.2198 31.9641 2.247 0.0317 *
## GroupMDD 1.0546 0.2062 28.6064 5.114 1.92e-05 ***
## ---
## Signif. codes: 0 '***' 0.001 '**' 0.01 '*' 0.05 '.' 0.1 ' ' 1
##
## Correlation of Fixed Effects:
## (Intr) SN_CO_
## SN_CO_Z -0.959
## GroupMDD -0.404 0.174

standardize_parameters(model2b.4)

## # Standardization method: refit
##
## Parameter | Std. Coef. | 95% CI
## -----------------------------------------
## (Intercept) | -0.49 | [-0.71, -0.26]
## SN_CO_Z | 0.11 | [ 0.01, 0.20]
## GroupMDD | 0.68 | [ 0.42, 0.94]

### 2b.5 Between-Network admn_lcen

model2b.5 <- lmer(formula = negemo_day_prop ~
 admn_lcen + Group +
 (1 |id_participant),
 data = df_aim1_all_language,
 na.action=na.exclude)
summary(model2b.5)

## Linear mixed model fit by REML. t-tests use Satterthwaite's method [
## lmerModLmerTest]
## Formula: negemo_day_prop ~ admn_lcen + Group + (1 | id_participant)
## Data: df_aim1_all_language
##
## REML criterion at convergence: 7418.5
##
## Scaled residuals:
## Min 1Q Median 3Q Max
## -2.2552 -0.5700 -0.0809 0.3792 10.1570
##
## Random effects:
## Groups Name Variance Std.Dev.
## id_participant (Intercept) 0.2402 0.4901
## Residual 2.1080 1.4519
## Number of obs: 2054, groups: id_participant, 32
##
## Fixed effects:
## Estimate Std. Error df t value Pr(>|t|)
## (Intercept) 1.5721 0.2275 30.3806 6.909 1.06e-07 ***
## admn_lcen 0.1905 0.3592 29.1571 0.530 0.59986
## GroupMDD 0.8808 0.2266 27.6215 3.886 0.00058 ***
## ---
## Signif. codes: 0 '***' 0.001 '**' 0.01 '*' 0.05 '.' 0.1 ' ' 1
##
## Correlation of Fixed Effects:
## (Intr) admn_l
## admn_lcen -0.516
## GroupMDD -0.720 -0.033

standardize_parameters(model2b.5)

## # Standardization method: refit
##
## Parameter | Std. Coef. | 95% CI
## -----------------------------------------
## (Intercept) | -0.38 | [-0.63, -0.13]
## admn_lcen | 0.03 | [-0.09, 0.15]
## GroupMDD | 0.57 | [ 0.28, 0.86]

### 2b.6 Between-Network admn_rcen

model2b.6 <- lmer(formula = negemo_day_prop ~
 admn_rcen + Group +
 (1 |id_participant),
 data = df_aim1_all_language,
 na.action=na.exclude)
summary(model2b.6)

## Linear mixed model fit by REML. t-tests use Satterthwaite's method [
## lmerModLmerTest]
## Formula: negemo_day_prop ~ admn_rcen + Group + (1 | id_participant)
## Data: df_aim1_all_language
##
## REML criterion at convergence: 7012.2
##
## Scaled residuals:
## Min 1Q Median 3Q Max
## -2.2638 -0.5557 -0.0791 0.3680 10.1230
##
## Random effects:
## Groups Name Variance Std.Dev.
## id_participant (Intercept) 0.2411 0.491
## Residual 2.1212 1.456
## Number of obs: 1938, groups: id_participant, 31
##
## Fixed effects:
## Estimate Std. Error df t value Pr(>|t|)
## (Intercept) 1.5282 0.2724 27.5281 5.611 5.55e-06 ***
## admn_rcen 0.2924 0.3273 24.1283 0.894 0.3804
## GroupMDD 0.8128 0.2426 26.9987 3.350 0.0024 **
## ---
## Signif. codes: 0 '***' 0.001 '**' 0.01 '*' 0.05 '.' 0.1 ' ' 1
##
## Correlation of Fixed Effects:
## (Intr) admn_r
## admn_rcen -0.630
## GroupMDD -0.605 -0.114

standardize_parameters(model2b.6)

## # Standardization method: refit
##
## Parameter | Std. Coef. | 95% CI
## -----------------------------------------
## (Intercept) | -0.38 | [-0.65, -0.10]
## admn_rcen | 0.06 | [-0.07, 0.20]
## GroupMDD | 0.53 | [ 0.22, 0.83]

### 2b.7 Between-Network admn_sn

model2b.7 <- lmer(formula = negemo_day_prop ~
 admn_sn + Group +
 (1 |id_participant),
 data = df_aim1_all_language,
 na.action=na.exclude)
summary(model2b.7)

## Linear mixed model fit by REML. t-tests use Satterthwaite's method [
## lmerModLmerTest]
## Formula: negemo_day_prop ~ admn_sn + Group + (1 | id_participant)
## Data: df_aim1_all_language
##
## REML criterion at convergence: 7012.5
##
## Scaled residuals:
## Min 1Q Median 3Q Max
## -2.2496 -0.5572 -0.0820 0.3701 10.1267
##
## Random effects:
## Groups Name Variance Std.Dev.
## id_participant (Intercept) 0.2444 0.4944
## Residual 2.1211 1.4564
## Number of obs: 1938, groups: id_participant, 31
##
## Fixed effects:
## Estimate Std. Error df t value Pr(>|t|)
## (Intercept) 1.6521 0.2164 28.0731 7.636 2.52e-08 ***
## admn_sn 0.2388 0.3250 25.1746 0.735 0.46930
## GroupMDD 0.8078 0.2458 26.8779 3.286 0.00283 **
## ---
## Signif. codes: 0 '***' 0.001 '**' 0.01 '*' 0.05 '.' 0.1 ' ' 1
##
## Correlation of Fixed Effects:
## (Intr) admn_s
## admn_sn -0.184
## GroupMDD -0.820 -0.167

standardize_parameters(model2b.7)

## # Standardization method: refit
##
## Parameter | Std. Coef. | 95% CI
## -----------------------------------------
## (Intercept) | -0.38 | [-0.65, -0.11]
## admn_sn | 0.04 | [-0.07, 0.16]
## GroupMDD | 0.52 | [ 0.21, 0.84]

### 2b.8 Between-Network sn_lcen

model2b.8 <- lmer(formula = negemo_day_prop ~
 sn_lcen + Group +
 (1 |id_participant),
 data = df_aim1_all_language,
 na.action=na.exclude)
summary(model2b.8)

## Linear mixed model fit by REML. t-tests use Satterthwaite's method [
## lmerModLmerTest]
## Formula: negemo_day_prop ~ sn_lcen + Group + (1 | id_participant)
## Data: df_aim1_all_language
##
## REML criterion at convergence: 7491.6
##
## Scaled residuals:
## Min 1Q Median 3Q Max
## -2.2447 -0.5751 -0.0861 0.3761 10.1200
##
## Random effects:
## Groups Name Variance Std.Dev.
## id_participant (Intercept) 0.2503 0.5003
## Residual 2.1223 1.4568
## Number of obs: 2070, groups: id_participant, 34
##
## Fixed effects:
## Estimate Std. Error df t value Pr(>|t|)
## (Intercept) 1.4680 0.2682 31.1164 5.473 5.47e-06 ***
## sn_lcen 0.1465 0.4130 29.2869 0.355 0.725395
## GroupMDD 1.0011 0.2268 29.6118 4.413 0.000124 ***
## ---
## Signif. codes: 0 '***' 0.001 '**' 0.01 '*' 0.05 '.' 0.1 ' ' 1
##
## Correlation of Fixed Effects:
## (Intr) sn_lcn
## sn_lcen -0.714
## GroupMDD -0.735 0.218

standardize_parameters(model2b.8)

## # Standardization method: refit
##
## Parameter | Std. Coef. | 95% CI
## -----------------------------------------
## (Intercept) | -0.44 | [-0.68, -0.21]
## sn_lcen | 0.02 | [-0.09, 0.14]
## GroupMDD | 0.64 | [ 0.36, 0.93]

### 2b.9 Between-Network sn_rcen

model2b.9 <- lmer(formula = negemo_day_prop ~
 sn_rcen + Group +
 (1 |id_participant),
 data = df_aim1_all_language,
 na.action=na.exclude)
summary(model2b.9)

## Linear mixed model fit by REML. t-tests use Satterthwaite's method [
## lmerModLmerTest]
## Formula: negemo_day_prop ~ sn_rcen + Group + (1 | id_participant)
## Data: df_aim1_all_language
##
## REML criterion at convergence: 7491.8
##
## Scaled residuals:
## Min 1Q Median 3Q Max
## -2.2401 -0.5764 -0.0881 0.3756 10.1177
##
## Random effects:
## Groups Name Variance Std.Dev.
## id_participant (Intercept) 0.2518 0.5018
## Residual 2.1223 1.4568
## Number of obs: 2070, groups: id_participant, 34
##
## Fixed effects:
## Estimate Std. Error df t value Pr(>|t|)
## (Intercept) 1.531666 0.252835 29.377249 6.058 1.29e-06 ***
## sn_rcen 0.009399 0.385075 27.584263 0.024 0.980703
## GroupMDD 0.984833 0.225956 29.186027 4.359 0.000148 ***
## ---
## Signif. codes: 0 '***' 0.001 '**' 0.01 '*' 0.05 '.' 0.1 ' ' 1
##
## Correlation of Fixed Effects:
## (Intr) sn_rcn
## sn_rcen -0.668
## GroupMDD -0.746 0.188

standardize_parameters(model2b.9)

## # Standardization method: refit
##
## Parameter | Std. Coef. | 95% CI
## -----------------------------------------
## (Intercept) | -0.44 | [-0.68, -0.21]
## sn_rcen | 1.38e-03 | [-0.11, 0.11]
## GroupMDD | 0.63 | [ 0.35, 0.92]

## S2b. Negative Emotion Words (controlling for depressive symptoms)

### S2b.1 Within-Network aDMN

modelS2b.1 <- lmer(formula = negemo_day_prop ~
 aDMN_Z + RADS_total
 + (1 |id_participant) ,
 data = df_aim1_all_language,
 na.action=na.exclude)
summary(modelS2b.1)

## Linear mixed model fit by REML. t-tests use Satterthwaite's method [
## lmerModLmerTest]
## Formula: negemo_day_prop ~ aDMN_Z + RADS_total + (1 | id_participant)
## Data: df_aim1_all_language
##
## REML criterion at convergence: 7503.1
##
## Scaled residuals:
## Min 1Q Median 3Q Max
## -2.3043 -0.5796 -0.0838 0.3767 10.1184
##
## Random effects:
## Groups Name Variance Std.Dev.
## id_participant (Intercept) 0.279 0.5282
## Residual 2.123 1.4571
## Number of obs: 2070, groups: id_participant, 34
##
## Fixed effects:
## Estimate Std. Error df t value Pr(>|t|)
## (Intercept) 0.276200 0.681995 34.462908 0.405 0.687990
## aDMN_Z 0.291986 0.316542 36.782345 0.922 0.362318
## RADS_total 0.020952 0.005386 28.321177 3.890 0.000556 ***
## ---
## Signif. codes: 0 '***' 0.001 '**' 0.01 '*' 0.05 '.' 0.1 ' ' 1
##
## Correlation of Fixed Effects:
## (Intr) aDMN_Z
## aDMN_Z -0.811
## RADS_total -0.711 0.195

standardize_parameters(modelS2b.1)

## # Standardization method: refit
##
## Parameter | Std. Coef. | 95% CI
## ----------------------------------------
## (Intercept) | 0.03 | [-0.10, 0.16]
## aDMN_Z | 0.05 | [-0.06, 0.16]
## RADS_total | 0.25 | [ 0.13, 0.38]

### S2b.2 Within-Network leftCEN_Z

modelS2b.2 <- lmer(formula = negemo_day_prop ~
 leftCEN_Z + RADS_total
 + (1 |id_participant) ,
 data = df_aim1_all_language,
 na.action=na.exclude)
summary(modelS2b.2)

## Linear mixed model fit by REML. t-tests use Satterthwaite's method [
## lmerModLmerTest]
## Formula: negemo_day_prop ~ leftCEN_Z + RADS_total + (1 | id_participant)
## Data: df_aim1_all_language
##
## REML criterion at convergence: 7502.8
##
## Scaled residuals:
## Min 1Q Median 3Q Max
## -2.3150 -0.5789 -0.0813 0.3779 10.1194
##
## Random effects:
## Groups Name Variance Std.Dev.
## id_participant (Intercept) 0.2805 0.5296
## Residual 2.1230 1.4571
## Number of obs: 2070, groups: id_participant, 34
##
## Fixed effects:
## Estimate Std. Error df t value Pr(>|t|)
## (Intercept) -0.076702 1.021203 37.368488 -0.075 0.940528
## leftCEN_Z 0.341166 0.371527 34.736832 0.918 0.364805
## RADS_total 0.022938 0.006193 32.944612 3.704 0.000774 ***
## ---
## Signif. codes: 0 '***' 0.001 '**' 0.01 '*' 0.05 '.' 0.1 ' ' 1
##
## Correlation of Fixed Effects:
## (Intr) lCEN_Z
## leftCEN_Z -0.920
## RADS_total -0.800 0.519

standardize_parameters(modelS2b.2)

## # Standardization method: refit
##
## Parameter | Std. Coef. | 95% CI
## ----------------------------------------
## (Intercept) | 0.03 | [-0.10, 0.16]
## leftCEN_Z | 0.07 | [-0.08, 0.22]
## RADS_total | 0.28 | [ 0.13, 0.42]

### S2b.3 Within-Network rightCEN_Z

modelS2b.3 <- lmer(formula = negemo_day_prop ~
 rightCEN_Z + RADS_total
 + (1 |id_participant) ,
 data = df_aim1_all_language,
 na.action=na.exclude)
summary(modelS2b.3)

## Linear mixed model fit by REML. t-tests use Satterthwaite's method [
## lmerModLmerTest]
## Formula: negemo_day_prop ~ rightCEN_Z + RADS_total + (1 | id_participant)
## Data: df_aim1_all_language
##
## REML criterion at convergence: 7502.9
##
## Scaled residuals:
## Min 1Q Median 3Q Max
## -2.2977 -0.5789 -0.0843 0.3769 10.1085
##
## Random effects:
## Groups Name Variance Std.Dev.
## id_participant (Intercept) 0.271 0.5206
## Residual 2.123 1.4571
## Number of obs: 2070, groups: id_participant, 34
##
## Fixed effects:
## Estimate Std. Error df t value Pr(>|t|)
## (Intercept) 1.60701 0.78669 27.55497 2.043 0.05075 .
## rightCEN_Z -0.30422 0.25307 27.34529 -1.202 0.23962
## RADS_total 0.01746 0.00562 27.04390 3.106 0.00442 **
## ---
## Signif. codes: 0 '***' 0.001 '**' 0.01 '*' 0.05 '.' 0.1 ' ' 1
##
## Correlation of Fixed Effects:
## (Intr) rCEN_Z
## rightCEN_Z -0.866
## RADS_total -0.770 0.371

standardize_parameters(modelS2b.3)

## # Standardization method: refit
##
## Parameter | Std. Coef. | 95% CI
## ----------------------------------------
## (Intercept) | 0.01 | [-0.12, 0.15]
## rightCEN_Z | -0.11 | [-0.29, 0.07]
## RADS_total | 0.21 | [ 0.08, 0.34]

### S2b.4 Within-Network SN_CO_Z

modelS2b.4 <- lmer(formula = negemo_day_prop ~
 SN_CO_Z + RADS_total
 + (1 |id_participant) ,
 data = df_aim1_all_language,
 na.action=na.exclude)
summary(modelS2b.4)

## Linear mixed model fit by REML. t-tests use Satterthwaite's method [
## lmerModLmerTest]
## Formula: negemo_day_prop ~ SN_CO_Z + RADS_total + (1 | id_participant)
## Data: df_aim1_all_language
##
## REML criterion at convergence: 7498.1
##
## Scaled residuals:
## Min 1Q Median 3Q Max
## -2.2851 -0.5794 -0.0829 0.3822 10.1241
##
## Random effects:
## Groups Name Variance Std.Dev.
## id_participant (Intercept) 0.2041 0.4518
## Residual 2.1248 1.4577
## Number of obs: 2070, groups: id_participant, 34
##
## Fixed effects:
## Estimate Std. Error df t value Pr(>|t|)
## (Intercept) -1.039218 0.763732 29.846689 -1.361 0.1838
## SN_CO_Z 0.618096 0.227345 30.658083 2.719 0.0107 *
## RADS_total 0.023574 0.004856 25.786385 4.855 5.03e-05 ***
## ---
## Signif. codes: 0 '***' 0.001 '**' 0.01 '*' 0.05 '.' 0.1 ' ' 1
##
## Correlation of Fixed Effects:
## (Intr) SN_CO_
## SN_CO_Z -0.888
## RADS_total -0.682 0.290

standardize_parameters(modelS2b.4)

## # Standardization method: refit
##
## Parameter | Std. Coef. | 95% CI
## ----------------------------------------
## (Intercept) | 0.02 | [-0.09, 0.14]
## SN_CO_Z | 0.14 | [ 0.04, 0.23]
## RADS_total | 0.28 | [ 0.17, 0.40]

### S2b.5 Between-Network admn_lcen

modelS2b.5 <- lmer(formula = negemo_day_prop ~
 admn_lcen + RADS_total
 + (1 |id_participant) ,
 data = df_aim1_all_language,
 na.action=na.exclude)
summary(modelS2b.5)

## Linear mixed model fit by REML. t-tests use Satterthwaite's method [
## lmerModLmerTest]
## Formula: negemo_day_prop ~ admn_lcen + RADS_total + (1 | id_participant)
## Data: df_aim1_all_language
##
## REML criterion at convergence: 7427
##
## Scaled residuals:
## Min 1Q Median 3Q Max
## -2.3011 -0.5757 -0.0830 0.3807 10.1535
##
## Random effects:
## Groups Name Variance Std.Dev.
## id_participant (Intercept) 0.2468 0.4968
## Residual 2.1083 1.4520
## Number of obs: 2054, groups: id_participant, 32
##
## Fixed effects:
## Estimate Std. Error df t value Pr(>|t|)
## (Intercept) 0.903332 0.384919 27.354310 2.347 0.026419 *
## admn_lcen -0.051556 0.371141 28.935883 -0.139 0.890481
## RADS_total 0.019206 0.005155 26.811265 3.726 0.000917 ***
## ---
## Signif. codes: 0 '***' 0.001 '**' 0.01 '*' 0.05 '.' 0.1 ' ' 1
##
## Correlation of Fixed Effects:
## (Intr) admn_l
## admn_lcen -0.125
## RADS_total -0.910 -0.209

standardize_parameters(modelS2b.5)

## # Standardization method: refit
##
## Parameter | Std. Coef. | 95% CI
## ----------------------------------------
## (Intercept) | 0.05 | [-0.08, 0.18]
## admn_lcen | -8.91e-03 | [-0.13, 0.12]
## RADS_total | 0.23 | [ 0.11, 0.36]

### S2b.6 Between-Network admn_rcen

modelS2b.9 <- lmer(formula = negemo_day_prop ~
 admn_rcen + RADS_total
 + (1 |id_participant) ,
 data = df_aim1_all_language,
 na.action=na.exclude)
summary(modelS2b.9)

## Linear mixed model fit by REML. t-tests use Satterthwaite's method [
## lmerModLmerTest]
## Formula: negemo_day_prop ~ admn_rcen + RADS_total + (1 | id_participant)
## Data: df_aim1_all_language
##
## REML criterion at convergence: 7021.1
##
## Scaled residuals:
## Min 1Q Median 3Q Max
## -2.3075 -0.5612 -0.0819 0.3714 10.1231
##
## Random effects:
## Groups Name Variance Std.Dev.
## id_participant (Intercept) 0.2558 0.5058
## Residual 2.1214 1.4565
## Number of obs: 1938, groups: id_participant, 31
##
## Fixed effects:
## Estimate Std. Error df t value Pr(>|t|)
## (Intercept) 0.901572 0.445154 28.010812 2.025 0.05246 .
## admn_rcen 0.165889 0.343623 23.857967 0.483 0.63366
## RADS_total 0.017752 0.005812 26.540586 3.055 0.00508 **
## ---
## Signif. codes: 0 '***' 0.001 '**' 0.01 '*' 0.05 '.' 0.1 ' ' 1
##
## Correlation of Fixed Effects:
## (Intr) admn_r
## admn_rcen -0.219
## RADS_total -0.866 -0.241

standardize_parameters(modelS2b.9)

## # Standardization method: refit
##
## Parameter | Std. Coef. | 95% CI
## ----------------------------------------
## (Intercept) | 0.05 | [-0.08, 0.19]
## admn_rcen | 0.04 | [-0.11, 0.18]
## RADS_total | 0.18 | [ 0.07, 0.30]

### S2b.7 Between-Network admn_sn

modelS2b.7 <- lmer(formula = negemo_day_prop ~
 admn_sn + RADS_total
 + (1 |id_participant) ,
 data = df_aim1_all_language,
 na.action=na.exclude)
summary(modelS2b.7)

## Linear mixed model fit by REML. t-tests use Satterthwaite's method [
## lmerModLmerTest]
## Formula: negemo_day_prop ~ admn_sn + RADS_total + (1 | id_participant)
## Data: df_aim1_all_language
##
## REML criterion at convergence: 7020.9
##
## Scaled residuals:
## Min 1Q Median 3Q Max
## -2.3043 -0.5597 -0.0817 0.3723 10.1281
##
## Random effects:
## Groups Name Variance Std.Dev.
## id_participant (Intercept) 0.253 0.5029
## Residual 2.121 1.4565
## Number of obs: 1938, groups: id_participant, 31
##
## Fixed effects:
## Estimate Std. Error df t value Pr(>|t|)
## (Intercept) 0.949180 0.432332 27.752462 2.195 0.03666 *
## admn_sn 0.244123 0.329898 25.437491 0.740 0.46608
## RADS_total 0.017705 0.005697 27.085395 3.108 0.00439 **
## ---
## Signif. codes: 0 '***' 0.001 '**' 0.01 '*' 0.05 '.' 0.1 ' ' 1
##
## Correlation of Fixed Effects:
## (Intr) admn_s
## admn_sn 0.000
## RADS_total -0.957 -0.170

standardize_parameters(modelS2b.7)

## # Standardization method: refit
##
## Parameter | Std. Coef. | 95% CI
## ----------------------------------------
## (Intercept) | 0.05 | [-0.09, 0.18]
## admn_sn | 0.05 | [-0.07, 0.17]
## RADS_total | 0.18 | [ 0.07, 0.30]

### S2b.8 Between-Network sn_lcen

modelS2b.8 <- lmer(formula = negemo_day_prop ~
 sn_lcen + RADS_total
 + (1 |id_participant) ,
 data = df_aim1_all_language,
 na.action=na.exclude)
summary(modelS2b.8)

## Linear mixed model fit by REML. t-tests use Satterthwaite's method [
## lmerModLmerTest]
## Formula: negemo_day_prop ~ sn_lcen + RADS_total + (1 | id_participant)
## Data: df_aim1_all_language
##
## REML criterion at convergence: 7503.3
##
## Scaled residuals:
## Min 1Q Median 3Q Max
## -2.3003 -0.5782 -0.0832 0.3776 10.1158
##
## Random effects:
## Groups Name Variance Std.Dev.
## id_participant (Intercept) 0.2858 0.5346
## Residual 2.1234 1.4572
## Number of obs: 2070, groups: id_participant, 34
##
## Fixed effects:
## Estimate Std. Error df t value Pr(>|t|)
## (Intercept) 0.772278 0.464328 27.281950 1.663 0.10772
## sn_lcen 0.024101 0.431954 27.972155 0.056 0.95590
## RADS_total 0.020055 0.005419 26.785771 3.701 0.00098 ***
## ---
## Signif. codes: 0 '***' 0.001 '**' 0.01 '*' 0.05 '.' 0.1 ' ' 1
##
## Correlation of Fixed Effects:
## (Intr) sn_lcn
## sn_lcen -0.498
## RADS_total -0.911 0.173

standardize_parameters(modelS2b.8)

## # Standardization method: refit
##
## Parameter | Std. Coef. | 95% CI
## ----------------------------------------
## (Intercept) | 0.03 | [-0.11, 0.17]
## sn_lcen | 3.41e-03 | [-0.12, 0.12]
## RADS_total | 0.24 | [ 0.11, 0.37]

### S2b.9 Between-Network sn_rcen

modelS2b.9 <- lmer(formula = negemo_day_prop ~
 sn_rcen + RADS_total
 + (1 |id_participant) ,
 data = df_aim1_all_language,
 na.action=na.exclude)
summary(modelS2b.9)

## Linear mixed model fit by REML. t-tests use Satterthwaite's method [
## lmerModLmerTest]
## Formula: negemo_day_prop ~ sn_rcen + RADS_total + (1 | id_participant)
## Data: df_aim1_all_language
##
## REML criterion at convergence: 7502.9
##
## Scaled residuals:
## Min 1Q Median 3Q Max
## -2.2920 -0.5793 -0.0835 0.3752 10.1109
##
## Random effects:
## Groups Name Variance Std.Dev.
## id_participant (Intercept) 0.2804 0.5295
## Residual 2.1233 1.4572
## Number of obs: 2070, groups: id_participant, 34
##
## Fixed effects:
## Estimate Std. Error df t value Pr(>|t|)
## (Intercept) 0.895746 0.424939 26.933040 2.108 0.044483 *
## sn_rcen -0.298489 0.395510 26.733235 -0.755 0.457029
## RADS_total 0.019955 0.005294 26.811543 3.769 0.000819 ***
## ---
## Signif. codes: 0 '***' 0.001 '**' 0.01 '*' 0.05 '.' 0.1 ' ' 1
##
## Correlation of Fixed Effects:
## (Intr) sn_rcn
## sn_rcen -0.341
## RADS_total -0.910 0.008

standardize_parameters(modelS2b.9)

## # Standardization method: refit
##
## Parameter | Std. Coef. | 95% CI
## ----------------------------------------
## (Intercept) | 0.02 | [-0.11, 0.16]
## sn_rcen | -0.04 | [-0.16, 0.07]
## RADS_total | 0.24 | [ 0.12, 0.37]

## 2c. Future focus words (controlling for Group)

### 2c.1 Within-Network aDMN

model2c.1 <- lmer(formula = focusfuture_day_prop ~
 aDMN_Z + Group +
 (1 |id_participant),
 data = df_aim1_all_language,
 na.action=na.exclude)
summary(model2c.1)

## Linear mixed model fit by REML. t-tests use Satterthwaite's method [
## lmerModLmerTest]
## Formula: focusfuture_day_prop ~ aDMN_Z + Group + (1 | id_participant)
## Data: df_aim1_all_language
##
## REML criterion at convergence: 6305.9
##
## Scaled residuals:
## Min 1Q Median 3Q Max
## -1.9513 -0.6115 -0.0903 0.4230 8.0910
##
## Random effects:
## Groups Name Variance Std.Dev.
## id_participant (Intercept) 0.1282 0.358
## Residual 1.1968 1.094
## Number of obs: 2070, groups: id_participant, 34
##
## Fixed effects:
## Estimate Std. Error df t value Pr(>|t|)
## (Intercept) 1.0226 0.3654 30.5717 2.798 0.00881 **
## aDMN_Z 0.4610 0.2176 31.5882 2.119 0.04205 *
## GroupMDD -0.3010 0.1608 25.4826 -1.872 0.07275 .
## ---
## Signif. codes: 0 '***' 0.001 '**' 0.01 '*' 0.05 '.' 0.1 ' ' 1
##
## Correlation of Fixed Effects:
## (Intr) aDMN_Z
## aDMN_Z -0.928
## GroupMDD -0.407 0.100

standardize_parameters(model2c.1)

## # Standardization method: refit
##
## Parameter | Std. Coef. | 95% CI
## ----------------------------------------
## (Intercept) | 0.27 | [ 0.04, 0.51]
## aDMN_Z | 0.11 | [ 0.01, 0.21]
## GroupMDD | -0.26 | [-0.54, 0.01]

### 2c.2 Within-Network leftCEN_Z

model2c.2 <- lmer(formula = focusfuture_day_prop ~
 leftCEN_Z + Group +
 (1 |id_participant),
 data = df_aim1_all_language,
 na.action=na.exclude)
summary(model2c.2)

## Linear mixed model fit by REML. t-tests use Satterthwaite's method [
## lmerModLmerTest]
## Formula: focusfuture_day_prop ~ leftCEN_Z + Group + (1 | id_participant)
## Data: df_aim1_all_language
##
## REML criterion at convergence: 6309.4
##
## Scaled residuals:
## Min 1Q Median 3Q Max
## -1.9763 -0.6099 -0.0892 0.4251 8.1168
##
## Random effects:
## Groups Name Variance Std.Dev.
## id_participant (Intercept) 0.137 0.3701
## Residual 1.198 1.0946
## Number of obs: 2070, groups: id_participant, 34
##
## Fixed effects:
## Estimate Std. Error df t value Pr(>|t|)
## (Intercept) 1.2788 0.6047 28.3934 2.115 0.0434 *
## leftCEN_Z 0.2119 0.2690 26.9690 0.788 0.4376
## GroupMDD -0.2505 0.1963 27.0938 -1.276 0.2128
## ---
## Signif. codes: 0 '***' 0.001 '**' 0.01 '*' 0.05 '.' 0.1 ' ' 1
##
## Correlation of Fixed Effects:
## (Intr) lCEN_Z
## leftCEN_Z -0.973
## GroupMDD -0.696 0.548

standardize_parameters(model2c.2)

## # Standardization method: refit
##
## Parameter | Std. Coef. | 95% CI
## ----------------------------------------
## (Intercept) | 0.24 | [-0.04, 0.51]
## leftCEN_Z | 0.06 | [-0.09, 0.21]
## GroupMDD | -0.22 | [-0.56, 0.12]

### 2c.3 Within-Network rightCEN_Z

model2c.3 <- lmer(formula = focusfuture_day_prop ~
 rightCEN_Z + Group +
 (1 |id_participant),
 data = df_aim1_all_language,
 na.action=na.exclude)
summary(model2c.3)

## Linear mixed model fit by REML. t-tests use Satterthwaite's method [
## lmerModLmerTest]
## Formula: focusfuture_day_prop ~ rightCEN_Z + Group + (1 | id_participant)
## Data: df_aim1_all_language
##
## REML criterion at convergence: 6310.6
##
## Scaled residuals:
## Min 1Q Median 3Q Max
## -1.9841 -0.6090 -0.0877 0.4251 8.1221
##
## Random effects:
## Groups Name Variance Std.Dev.
## id_participant (Intercept) 0.1382 0.3717
## Residual 1.1983 1.0947
## Number of obs: 2070, groups: id_participant, 34
##
## Fixed effects:
## Estimate Std. Error df t value Pr(>|t|)
## (Intercept) 1.9386 0.4302 21.1757 4.506 0.00019 ***
## rightCEN_Z -0.0853 0.1771 21.2235 -0.482 0.63503
## GroupMDD -0.3597 0.1725 22.4758 -2.085 0.04862 *
## ---
## Signif. codes: 0 '***' 0.001 '**' 0.01 '*' 0.05 '.' 0.1 ' ' 1
##
## Correlation of Fixed Effects:
## (Intr) rCEN_Z
## rightCEN_Z -0.946
## GroupMDD -0.542 0.294

standardize_parameters(model2c.3)

## # Standardization method: refit
##
## Parameter | Std. Coef. | 95% CI
## -----------------------------------------
## (Intercept) | 0.30 | [ 0.06, 0.55]
## rightCEN_Z | -0.04 | [-0.21, 0.13]
## GroupMDD | -0.31 | [-0.61, -0.02]

### 2c.4 Within-Network SN_CO_Z

model2c.4 <- lmer(formula = focusfuture_day_prop ~
 SN_CO_Z + Group +
 (1 |id_participant),
 data = df_aim1_all_language,
 na.action=na.exclude)
summary(model2c.4)

## Linear mixed model fit by REML. t-tests use Satterthwaite's method [
## lmerModLmerTest]
## Formula: focusfuture_day_prop ~ SN_CO_Z + Group + (1 | id_participant)
## Data: df_aim1_all_language
##
## REML criterion at convergence: 6310.8
##
## Scaled residuals:
## Min 1Q Median 3Q Max
## -1.9867 -0.6090 -0.0897 0.4257 8.1182
##
## Random effects:
## Groups Name Variance Std.Dev.
## id_participant (Intercept) 0.1384 0.3721
## Residual 1.1984 1.0947
## Number of obs: 2070, groups: id_participant, 34
##
## Fixed effects:
## Estimate Std. Error df t value Pr(>|t|)
## (Intercept) 1.82010 0.49162 25.28286 3.702 0.00105 **
## SN_CO_Z -0.02921 0.17777 25.72946 -0.164 0.87075
## GroupMDD -0.33993 0.16747 23.35782 -2.030 0.05392 .
## ---
## Signif. codes: 0 '***' 0.001 '**' 0.01 '*' 0.05 '.' 0.1 ' ' 1
##
## Correlation of Fixed Effects:
## (Intr) SN_CO_
## SN_CO_Z -0.959
## GroupMDD -0.402 0.172

standardize_parameters(model2c.4)

## # Standardization method: refit
##
## Parameter | Std. Coef. | 95% CI
## -----------------------------------------
## (Intercept) | 0.30 | [ 0.05, 0.54]
## SN_CO_Z | -8.71e-03 | [-0.11, 0.10]
## GroupMDD | -0.30 | [-0.58, -0.01]

### 2c.5 Between-Network admn_lcen

model2c.5 <- lmer(formula = focusfuture_day_prop ~
 admn_lcen + Group +
 (1 |id_participant),
 data = df_aim1_all_language,
 na.action=na.exclude)
summary(model2c.5)

## Linear mixed model fit by REML. t-tests use Satterthwaite's method [
## lmerModLmerTest]
## Formula: focusfuture_day_prop ~ admn_lcen + Group + (1 | id_participant)
## Data: df_aim1_all_language
##
## REML criterion at convergence: 6221
##
## Scaled residuals:
## Min 1Q Median 3Q Max
## -2.0268 -0.6086 -0.0899 0.4251 8.1950
##
## Random effects:
## Groups Name Variance Std.Dev.
## id_participant (Intercept) 0.1372 0.3703
## Residual 1.1754 1.0842
## Number of obs: 2054, groups: id_participant, 32
##
## Fixed effects:
## Estimate Std. Error df t value Pr(>|t|)
## (Intercept) 1.8812 0.1715 24.2849 10.970 6.79e-11 ***
## admn_lcen -0.3065 0.2708 23.2932 -1.132 0.2692
## GroupMDD -0.3671 0.1709 22.0667 -2.148 0.0429 *
## ---
## Signif. codes: 0 '***' 0.001 '**' 0.01 '*' 0.05 '.' 0.1 ' ' 1
##
## Correlation of Fixed Effects:
## (Intr) admn_l
## admn_lcen -0.515
## GroupMDD -0.720 -0.034

standardize_parameters(model2c.5)

## # Standardization method: refit
##
## Parameter | Std. Coef. | 95% CI
## -----------------------------------------
## (Intercept) | 0.31 | [ 0.05, 0.57]
## admn_lcen | -0.07 | [-0.20, 0.05]
## GroupMDD | -0.32 | [-0.62, -0.03]

### 2c.6 Between-Network admn_rcen

model2c.6 <- lmer(formula = focusfuture_day_prop ~
 admn_rcen + Group +
 (1 |id_participant),
 data = df_aim1_all_language,
 na.action=na.exclude)
summary(model2c.6)

## Linear mixed model fit by REML. t-tests use Satterthwaite's method [
## lmerModLmerTest]
## Formula: focusfuture_day_prop ~ admn_rcen + Group + (1 | id_participant)
## Data: df_aim1_all_language
##
## REML criterion at convergence: 5777.3
##
## Scaled residuals:
## Min 1Q Median 3Q Max
## -2.1139 -0.6173 -0.0875 0.4178 8.3969
##
## Random effects:
## Groups Name Variance Std.Dev.
## id_participant (Intercept) 0.1303 0.361
## Residual 1.1202 1.058
## Number of obs: 1938, groups: id_participant, 31
##
## Fixed effects:
## Estimate Std. Error df t value Pr(>|t|)
## (Intercept) 2.0293 0.1998 21.8778 10.157 9.63e-10 ***
## admn_rcen -0.4964 0.2402 19.1838 -2.066 0.0526 .
## GroupMDD -0.3195 0.1780 21.4623 -1.795 0.0867 .
## ---
## Signif. codes: 0 '***' 0.001 '**' 0.01 '*' 0.05 '.' 0.1 ' ' 1
##
## Correlation of Fixed Effects:
## (Intr) admn_r
## admn_rcen -0.630
## GroupMDD -0.605 -0.115

standardize_parameters(model2c.6)

## # Standardization method: refit
##
## Parameter | Std. Coef. | 95% CI
## -----------------------------------------
## (Intercept) | 0.31 | [ 0.03, 0.58]
## admn_rcen | -0.15 | [-0.29, -0.01]
## GroupMDD | -0.29 | [-0.60, 0.03]

### 2c.7 Between-Network admn_sn

model2c.7 <- lmer(formula = focusfuture_day_prop ~
 admn_sn + Group +
 (1 |id_participant),
 data = df_aim1_all_language,
 na.action=na.exclude)
summary(model2c.7)

## Linear mixed model fit by REML. t-tests use Satterthwaite's method [
## lmerModLmerTest]
## Formula: focusfuture_day_prop ~ admn_sn + Group + (1 | id_participant)
## Data: df_aim1_all_language
##
## REML criterion at convergence: 5780.9
##
## Scaled residuals:
## Min 1Q Median 3Q Max
## -2.0898 -0.6133 -0.0895 0.4090 8.3981
##
## Random effects:
## Groups Name Variance Std.Dev.
## id_participant (Intercept) 0.1529 0.391
## Residual 1.1203 1.058
## Number of obs: 1938, groups: id_participant, 31
##
## Fixed effects:
## Estimate Std. Error df t value Pr(>|t|)
## (Intercept) 1.7947 0.1683 22.4970 10.663 2.88e-10 ***
## admn_sn -0.1798 0.2538 20.1442 -0.708 0.4868
## GroupMDD -0.3401 0.1915 21.5929 -1.776 0.0899 .
## ---
## Signif. codes: 0 '***' 0.001 '**' 0.01 '*' 0.05 '.' 0.1 ' ' 1
##
## Correlation of Fixed Effects:
## (Intr) admn_s
## admn_sn -0.181
## GroupMDD -0.819 -0.171

standardize_parameters(model2c.7)

## # Standardization method: refit
##
## Parameter | Std. Coef. | 95% CI
## ----------------------------------------
## (Intercept) | 0.34 | [ 0.04, 0.63]
## admn_sn | -0.05 | [-0.17, 0.08]
## GroupMDD | -0.31 | [-0.65, 0.03]

### 2c.8 Between-Network sn_lcen

model2c.8 <- lmer(formula = focusfuture_day_prop ~
 sn_lcen + Group +
 (1 |id_participant),
 data = df_aim1_all_language,
 na.action=na.exclude)
summary(model2c.8)

## Linear mixed model fit by REML. t-tests use Satterthwaite's method [
## lmerModLmerTest]
## Formula: focusfuture_day_prop ~ sn_lcen + Group + (1 | id_participant)
## Data: df_aim1_all_language
##
## REML criterion at convergence: 6309.7
##
## Scaled residuals:
## Min 1Q Median 3Q Max
## -1.9844 -0.6086 -0.0889 0.4262 8.1185
##
## Random effects:
## Groups Name Variance Std.Dev.
## id_participant (Intercept) 0.1397 0.3737
## Residual 1.1982 1.0946
## Number of obs: 2070, groups: id_participant, 34
##
## Fixed effects:
## Estimate Std. Error df t value Pr(>|t|)
## (Intercept) 1.76975 0.20064 24.46205 8.821 4.56e-09 ***
## sn_lcen -0.05809 0.30884 23.01255 -0.188 0.8524
## GroupMDD -0.34216 0.16965 23.26394 -2.017 0.0554 .
## ---
## Signif. codes: 0 '***' 0.001 '**' 0.01 '*' 0.05 '.' 0.1 ' ' 1
##
## Correlation of Fixed Effects:
## (Intr) sn_lcn
## sn_lcen -0.714
## GroupMDD -0.735 0.218

standardize_parameters(model2c.8)

## # Standardization method: refit
##
## Parameter | Std. Coef. | 95% CI
## -----------------------------------------
## (Intercept) | 0.29 | [ 0.05, 0.54]
## sn_lcen | -0.01 | [-0.13, 0.11]
## GroupMDD | -0.30 | [-0.59, -0.01]

### 2c.9 Between-Network sn_rcen

model2c.9 <- lmer(formula = focusfuture_day_prop ~
 sn_rcen + Group +
 (1 |id_participant),
 data = df_aim1_all_language,
 na.action=na.exclude)
summary(model2c.9)

## Linear mixed model fit by REML. t-tests use Satterthwaite's method [
## lmerModLmerTest]
## Formula: focusfuture_day_prop ~ sn_rcen + Group + (1 | id_participant)
## Data: df_aim1_all_language
##
## REML criterion at convergence: 6309.4
##
## Scaled residuals:
## Min 1Q Median 3Q Max
## -1.9898 -0.6091 -0.0869 0.4272 8.1179
##
## Random effects:
## Groups Name Variance Std.Dev.
## id_participant (Intercept) 0.1401 0.3743
## Residual 1.1979 1.0945
## Number of obs: 2070, groups: id_participant, 34
##
## Fixed effects:
## Estimate Std. Error df t value Pr(>|t|)
## (Intercept) 1.8323 0.1889 23.5839 9.702 1.05e-09 ***
## sn_rcen -0.2040 0.2876 22.1444 -0.709 0.4855
## GroupMDD -0.3578 0.1688 23.4239 -2.120 0.0448 *
## ---
## Signif. codes: 0 '***' 0.001 '**' 0.01 '*' 0.05 '.' 0.1 ' ' 1
##
## Correlation of Fixed Effects:
## (Intr) sn_rcn
## sn_rcen -0.668
## GroupMDD -0.746 0.188

standardize_parameters(model2c.9)

## # Standardization method: refit
##
## Parameter | Std. Coef. | 95% CI
## -----------------------------------------
## (Intercept) | 0.30 | [ 0.06, 0.54]
## sn_rcen | -0.04 | [-0.15, 0.07]
## GroupMDD | -0.31 | [-0.60, -0.02]

## S2c. Future Focus Words (controlling for depressive symptoms)

### S2c.1 Within-Network aDMN

modelS2c.1 <- lmer(formula = focusfuture_day_prop ~
 aDMN_Z + RADS_total
 + (1 |id_participant) ,
 data = df_aim1_all_language,
 na.action=na.exclude)
summary(modelS2c.1)

## Linear mixed model fit by REML. t-tests use Satterthwaite's method [
## lmerModLmerTest]
## Formula: focusfuture_day_prop ~ aDMN_Z + RADS_total + (1 | id_participant)
## Data: df_aim1_all_language
##
## REML criterion at convergence: 6314.5
##
## Scaled residuals:
## Min 1Q Median 3Q Max
## -1.9431 -0.6124 -0.0927 0.4208 8.1051
##
## Random effects:
## Groups Name Variance Std.Dev.
## id_participant (Intercept) 0.1356 0.3682
## Residual 1.1966 1.0939
## Number of obs: 2070, groups: id_participant, 34
##
## Fixed effects:
## Estimate Std. Error df t value Pr(>|t|)
## (Intercept) 1.270076 0.484709 30.836242 2.620 0.0135 *
## aDMN_Z 0.438115 0.225353 32.740619 1.944 0.0605 .
## RADS_total -0.005884 0.003806 24.901582 -1.546 0.1347
## ---
## Signif. codes: 0 '***' 0.001 '**' 0.01 '*' 0.05 '.' 0.1 ' ' 1
##
## Correlation of Fixed Effects:
## (Intr) aDMN_Z
## aDMN_Z -0.814
## RADS_total -0.711 0.199

standardize_parameters(modelS2c.1)

## # Standardization method: refit
##
## Parameter | Std. Coef. | 95% CI
## ----------------------------------------
## (Intercept) | 0.08 | [-0.05, 0.20]
## aDMN_Z | 0.10 | [ 0.00, 0.20]
## RADS_total | -0.10 | [-0.22, 0.03]

### S2c.2 Within-Network leftCEN_Z

modelS2c.2 <- lmer(formula = focusfuture_day_prop ~
 leftCEN_Z + RADS_total
 + (1 |id_participant) ,
 data = df_aim1_all_language,
 na.action=na.exclude)
summary(modelS2c.2)

## Linear mixed model fit by REML. t-tests use Satterthwaite's method [
## lmerModLmerTest]
## Formula: focusfuture_day_prop ~ leftCEN_Z + RADS_total + (1 | id_participant)
## Data: df_aim1_all_language
##
## REML criterion at convergence: 6317.1
##
## Scaled residuals:
## Min 1Q Median 3Q Max
## -1.9602 -0.6151 -0.0903 0.4252 8.1227
##
## Random effects:
## Groups Name Variance Std.Dev.
## id_participant (Intercept) 0.1397 0.3738
## Residual 1.1980 1.0945
## Number of obs: 2070, groups: id_participant, 34
##
## Fixed effects:
## Estimate Std. Error df t value Pr(>|t|)
## (Intercept) 1.449090 0.733785 31.041720 1.975 0.0572 .
## leftCEN_Z 0.232210 0.266518 28.842415 0.871 0.3908
## RADS_total -0.005334 0.004437 27.206590 -1.202 0.2396
## ---
## Signif. codes: 0 '***' 0.001 '**' 0.01 '*' 0.05 '.' 0.1 ' ' 1
##
## Correlation of Fixed Effects:
## (Intr) lCEN_Z
## leftCEN_Z -0.921
## RADS_total -0.803 0.525

standardize_parameters(modelS2c.2)

## # Standardization method: refit
##
## Parameter | Std. Coef. | 95% CI
## ----------------------------------------
## (Intercept) | 0.07 | [-0.06, 0.20]
## leftCEN_Z | 0.06 | [-0.08, 0.21]
## RADS_total | -0.09 | [-0.23, 0.06]

### S2c.3 Within-Network rightCEN_Z

modelS2c.3 <- lmer(formula = focusfuture_day_prop ~
 rightCEN_Z + RADS_total
 + (1 |id_participant) ,
 data = df_aim1_all_language,
 na.action=na.exclude)
summary(modelS2c.3)

## Linear mixed model fit by REML. t-tests use Satterthwaite's method [
## lmerModLmerTest]
## Formula: focusfuture_day_prop ~ rightCEN_Z + RADS_total + (1 | id_participant)
## Data: df_aim1_all_language
##
## REML criterion at convergence: 6318.3
##
## Scaled residuals:
## Min 1Q Median 3Q Max
## -1.9601 -0.6104 -0.0877 0.4217 8.1305
##
## Random effects:
## Groups Name Variance Std.Dev.
## id_participant (Intercept) 0.1403 0.3746
## Residual 1.1981 1.0946
## Number of obs: 2070, groups: id_participant, 34
##
## Fixed effects:
## Estimate Std. Error df t value Pr(>|t|)
## (Intercept) 2.349651 0.570433 22.251387 4.119 0.000442 ***
## rightCEN_Z -0.115657 0.183471 22.009311 -0.630 0.534935
## RADS_total -0.008317 0.004074 21.970474 -2.041 0.053395 .
## ---
## Signif. codes: 0 '***' 0.001 '**' 0.01 '*' 0.05 '.' 0.1 ' ' 1
##
## Correlation of Fixed Effects:
## (Intr) rCEN_Z
## rightCEN_Z -0.866
## RADS_total -0.770 0.371

standardize_parameters(modelS2c.3)

## # Standardization method: refit
##
## Parameter | Std. Coef. | 95% CI
## -----------------------------------------
## (Intercept) | 0.06 | [-0.07, 0.20]
## rightCEN_Z | -0.06 | [-0.23, 0.12]
## RADS_total | -0.14 | [-0.27, -0.01]

### S2c.4 Within-Network SN_CO_Z

modelS2c.4 <- lmer(formula = focusfuture_day_prop ~
 SN_CO_Z + RADS_total
 + (1 |id_participant) ,
 data = df_aim1_all_language,
 na.action=na.exclude)
summary(modelS2c.4)

## Linear mixed model fit by REML. t-tests use Satterthwaite's method [
## lmerModLmerTest]
## Formula: focusfuture_day_prop ~ SN_CO_Z + RADS_total + (1 | id_participant)
## Data: df_aim1_all_language
##
## REML criterion at convergence: 6318.5
##
## Scaled residuals:
## Min 1Q Median 3Q Max
## -1.9698 -0.6110 -0.0869 0.4240 8.1232
##
## Random effects:
## Groups Name Variance Std.Dev.
## id_participant (Intercept) 0.141 0.3755
## Residual 1.198 1.0946
## Number of obs: 2070, groups: id_participant, 34
##
## Fixed effects:
## Estimate Std. Error df t value Pr(>|t|)
## (Intercept) 2.249095 0.618643 25.831442 3.636 0.00121 **
## SN_CO_Z -0.070635 0.183985 26.425085 -0.384 0.70411
## RADS_total -0.007798 0.003955 22.610672 -1.972 0.06099 .
## ---
## Signif. codes: 0 '***' 0.001 '**' 0.01 '*' 0.05 '.' 0.1 ' ' 1
##
## Correlation of Fixed Effects:
## (Intr) SN_CO_
## SN_CO_Z -0.887
## RADS_total -0.681 0.285

standardize_parameters(modelS2c.4)

## # Standardization method: refit
##
## Parameter | Std. Coef. | 95% CI
## -----------------------------------------
## (Intercept) | 0.07 | [-0.05, 0.20]
## SN_CO_Z | -0.02 | [-0.13, 0.09]
## RADS_total | -0.13 | [-0.26, 0.00]

### S2c.5 Between-Network admn_lcen

modelS2c.5 <- lmer(formula = focusfuture_day_prop ~
 admn_lcen + RADS_total
 + (1 |id_participant) ,
 data = df_aim1_all_language,
 na.action=na.exclude)
summary(modelS2c.5)

## Linear mixed model fit by REML. t-tests use Satterthwaite's method [
## lmerModLmerTest]
## Formula: focusfuture_day_prop ~ admn_lcen + RADS_total + (1 | id_participant)
## Data: df_aim1_all_language
##
## REML criterion at convergence: 6230.2
##
## Scaled residuals:
## Min 1Q Median 3Q Max
## -1.9971 -0.6133 -0.0888 0.4210 8.2099
##
## Random effects:
## Groups Name Variance Std.Dev.
## id_participant (Intercept) 0.1487 0.3857
## Residual 1.1753 1.0841
## Number of obs: 2054, groups: id_participant, 32
##
## Fixed effects:
## Estimate Std. Error df t value Pr(>|t|)
## (Intercept) 2.070359 0.296835 21.874792 6.975 5.49e-07 ***
## admn_lcen -0.232453 0.285956 23.120869 -0.813 0.425
## RADS_total -0.006638 0.003976 21.421603 -1.669 0.110
## ---
## Signif. codes: 0 '***' 0.001 '**' 0.01 '*' 0.05 '.' 0.1 ' ' 1
##
## Correlation of Fixed Effects:
## (Intr) admn_l
## admn_lcen -0.123
## RADS_total -0.910 -0.210

standardize_parameters(modelS2c.5)

## # Standardization method: refit
##
## Parameter | Std. Coef. | 95% CI
## ----------------------------------------
## (Intercept) | 0.07 | [-0.07, 0.20]
## admn_lcen | -0.05 | [-0.19, 0.08]
## RADS_total | -0.11 | [-0.24, 0.02]

### S2c.6 Between-Network admn_rcen

modelS2c.6 <- lmer(formula = focusfuture_day_prop ~
 admn_rcen + RADS_total
 + (1 |id_participant) ,
 data = df_aim1_all_language,
 na.action=na.exclude)
summary(modelS2c.6)

## Linear mixed model fit by REML. t-tests use Satterthwaite's method [
## lmerModLmerTest]
## Formula: focusfuture_day_prop ~ admn_rcen + RADS_total + (1 | id_participant)
## Data: df_aim1_all_language
##
## REML criterion at convergence: 5786.5
##
## Scaled residuals:
## Min 1Q Median 3Q Max
## -2.0856 -0.6218 -0.0908 0.4121 8.4129
##
## Random effects:
## Groups Name Variance Std.Dev.
## id_participant (Intercept) 0.1428 0.378
## Residual 1.1200 1.058
## Number of obs: 1938, groups: id_participant, 31
##
## Fixed effects:
## Estimate Std. Error df t value Pr(>|t|)
## (Intercept) 2.161205 0.330829 22.402697 6.533 1.31e-06 ***
## admn_rcen -0.478789 0.255849 19.138462 -1.871 0.0767 .
## RADS_total -0.005173 0.004322 21.253210 -1.197 0.2445
## ---
## Signif. codes: 0 '***' 0.001 '**' 0.01 '*' 0.05 '.' 0.1 ' ' 1
##
## Correlation of Fixed Effects:
## (Intr) admn_r
## admn_rcen -0.219
## RADS_total -0.866 -0.243

standardize_parameters(modelS2c.6)

## # Standardization method: refit
##
## Parameter | Std. Coef. | 95% CI
## ----------------------------------------
## (Intercept) | 0.08 | [-0.06, 0.21]
## admn_rcen | -0.14 | [-0.29, 0.01]
## RADS_total | -0.07 | [-0.20, 0.05]

### S2c.7 Between-Network admn_sn

modelS2c.7 <- lmer(formula = focusfuture_day_prop ~
 admn_sn + RADS_total
 + (1 |id_participant) ,
 data = df_aim1_all_language,
 na.action=na.exclude)
summary(modelS2c.7)

## Linear mixed model fit by REML. t-tests use Satterthwaite's method [
## lmerModLmerTest]
## Formula: focusfuture_day_prop ~ admn_sn + RADS_total + (1 | id_participant)
## Data: df_aim1_all_language
##
## REML criterion at convergence: 5789.4
##
## Scaled residuals:
## Min 1Q Median 3Q Max
## -2.0696 -0.6158 -0.0887 0.4105 8.4089
##
## Random effects:
## Groups Name Variance Std.Dev.
## id_participant (Intercept) 0.1614 0.4018
## Residual 1.1201 1.0584
## Number of obs: 1938, groups: id_participant, 31
##
## Fixed effects:
## Estimate Std. Error df t value Pr(>|t|)
## (Intercept) 2.030424 0.339416 22.252881 5.982 4.85e-06 ***
## admn_sn -0.194270 0.259891 20.388363 -0.748 0.463
## RADS_total -0.006599 0.004477 21.722800 -1.474 0.155
## ---
## Signif. codes: 0 '***' 0.001 '**' 0.01 '*' 0.05 '.' 0.1 ' ' 1
##
## Correlation of Fixed Effects:
## (Intr) admn_s
## admn_sn 0.002
## RADS_total -0.957 -0.173

standardize_parameters(modelS2c.7)

## # Standardization method: refit
##
## Parameter | Std. Coef. | 95% CI
## ----------------------------------------
## (Intercept) | 0.09 | [-0.05, 0.24]
## admn_sn | -0.05 | [-0.18, 0.08]
## RADS_total | -0.10 | [-0.22, 0.03]

### S2c.8 Between-Network sn_lcen

modelS2c.8 <- lmer(formula = focusfuture_day_prop ~
 sn_lcen + RADS_total
 + (1 |id_participant) ,
 data = df_aim1_all_language,
 na.action=na.exclude)
summary(modelS2c.8)

## Linear mixed model fit by REML. t-tests use Satterthwaite's method [
## lmerModLmerTest]
## Formula: focusfuture_day_prop ~ sn_lcen + RADS_total + (1 | id_participant)
## Data: df_aim1_all_language
##
## REML criterion at convergence: 6317.6
##
## Scaled residuals:
## Min 1Q Median 3Q Max
## -1.9619 -0.6111 -0.0860 0.4237 8.1278
##
## Random effects:
## Groups Name Variance Std.Dev.
## id_participant (Intercept) 0.1433 0.3786
## Residual 1.1981 1.0946
## Number of obs: 2070, groups: id_participant, 34
##
## Fixed effects:
## Estimate Std. Error df t value Pr(>|t|)
## (Intercept) 2.053007 0.332146 22.183869 6.181 3.09e-06 ***
## sn_lcen -0.026023 0.309183 22.794451 -0.084 0.9337
## RADS_total -0.007426 0.003874 21.761862 -1.917 0.0685 .
## ---
## Signif. codes: 0 '***' 0.001 '**' 0.01 '*' 0.05 '.' 0.1 ' ' 1
##
## Correlation of Fixed Effects:
## (Intr) sn_lcn
## sn_lcen -0.498
## RADS_total -0.910 0.172

standardize_parameters(modelS2c.8)

## # Standardization method: refit
##
## Parameter | Std. Coef. | 95% CI
## ----------------------------------------
## (Intercept) | 0.07 | [-0.06, 0.21]
## sn_lcen | -5.02e-03 | [-0.12, 0.11]
## RADS_total | -0.12 | [-0.25, 0.00]

### S2c.9 Between-Network sn_rcen

modelS2c.9 <- lmer(formula = focusfuture_day_prop ~
 sn_rcen + RADS_total
 + (1 |id_participant) ,
 data = df_aim1_all_language,
 na.action=na.exclude)
summary(modelS2c.9)

## Linear mixed model fit by REML. t-tests use Satterthwaite's method [
## lmerModLmerTest]
## Formula: focusfuture_day_prop ~ sn_rcen + RADS_total + (1 | id_participant)
## Data: df_aim1_all_language
##
## REML criterion at convergence: 6317.7
##
## Scaled residuals:
## Min 1Q Median 3Q Max
## -1.9638 -0.6112 -0.0857 0.4242 8.1284
##
## Random effects:
## Groups Name Variance Std.Dev.
## id_participant (Intercept) 0.1444 0.3799
## Residual 1.1979 1.0945
## Number of obs: 2070, groups: id_participant, 34
##
## Fixed effects:
## Estimate Std. Error df t value Pr(>|t|)
## (Intercept) 2.074470 0.307277 22.163579 6.751 8.41e-07 ***
## sn_rcen -0.095921 0.285968 22.094906 -0.335 0.7405
## RADS_total -0.007381 0.003828 22.085371 -1.928 0.0668 .
## ---
## Signif. codes: 0 '***' 0.001 '**' 0.01 '*' 0.05 '.' 0.1 ' ' 1
##
## Correlation of Fixed Effects:
## (Intr) sn_rcn
## sn_rcen -0.341
## RADS_total -0.910 0.007

standardize_parameters(modelS2c.9)

## # Standardization method: refit
##
## Parameter | Std. Coef. | 95% CI
## ----------------------------------------
## (Intercept) | 0.07 | [-0.06, 0.20]
## sn_rcen | -0.02 | [-0.13, 0.09]
## RADS_total | -0.12 | [-0.24, 0.00]

## Aim 2 Adjusted P-values

#Correct for multiple comparisons of 4 hypothesized DMN networks

#First-person pronouns and DMN network connectivity (controlling for depressive symptoms)
fpp_network_pvalues <- c(0.03802, 0.98389, 0.66196, 0.84840)
p.adjust(fpp_network_pvalues,method="fdr")

## [1] 0.15208 0.98389 0.98389 0.98389

#Future focus words and network connectivity (controlling for MDD. Best fit DMN model includes age.)
future_network_pvalues_1 <- c(0.029, 0.2692, 0.0526, 0.4868)
p.adjust(future_network_pvalues_1,method="fdr")

## [1] 0.1052000 0.3589333 0.1052000 0.4868000

# 3. AIM 3: Mediation: Does intrinsic network connectivity mediate the association between depression and depression-related linguistic features of smartphone use?

## 3a. Dep Sx - leftCEN - First-person Pronouns

#install.packages("RMediation")
library(RMediation)

## Loading required package: lavaan

## This is lavaan 0.6-16
## lavaan is FREE software! Please report any bugs.

##
## Attaching package: 'lavaan'

## The following object is masked from 'package:psych':
##
## cor2cov

## Loading required package: e1071

## Loading required package: OpenMx

## OpenMx may run faster if it is compiled to take advantage of multiple cores.

##
## Attaching package: 'OpenMx'

## The following objects are masked from 'package:Matrix':
##
## %&%, expm

## The following object is masked from 'package:psych':
##
## tr

fit1 <- lmer(scale(i_day_prop) ~ 1 + scale(RADS_total) + scale(Age.at.V2) + Sex + (1|id_participant),
 data = df_aim1_all_language,
 na.action=na.exclude)

fit2<- lm(scale(leftCEN_Z) ~ scale(RADS_total) + scale(Age.at.V2) + Sex, df_px_means, na.action=na.exclude)

fit3 <- lmer(scale(i_day_prop) ~ 1 + scale(RADS_total) + scale(leftCEN_Z) + scale(Age.at.V2) + Sex + (1|id_participant),
 data = df_aim1_all_language,
 na.action=na.exclude)

summary(fit1)

## Linear mixed model fit by REML. t-tests use Satterthwaite's method [
## lmerModLmerTest]
## Formula: scale(i_day_prop) ~ 1 + scale(RADS_total) + scale(Age.at.V2) +
## Sex + (1 | id_participant)
## Data: df_aim1_all_language
##
## REML criterion at convergence: 6089.1
##
## Scaled residuals:
## Min 1Q Median 3Q Max
## -3.1275 -0.5758 -0.0125 0.5057 7.4829
##
## Random effects:
## Groups Name Variance Std.Dev.
## id_participant (Intercept) 0.1464 0.3826
## Residual 0.8430 0.9182
## Number of obs: 2251, groups: id_participant, 40
##
## Fixed effects:
## Estimate Std. Error df t value Pr(>|t|)
## (Intercept) -0.18796 0.25294 31.83071 -0.743 0.4629
## scale(RADS_total) 0.16888 0.06885 32.48517 2.453 0.0197 *
## scale(Age.at.V2) -0.01481 0.07313 32.40718 -0.202 0.8408
## Sex 0.12288 0.14862 32.79945 0.827 0.4143
## ---
## Signif. codes: 0 '***' 0.001 '**' 0.01 '*' 0.05 '.' 0.1 ' ' 1
##
## Correlation of Fixed Effects:
## (Intr) s(RADS s(A..V
## scl(RADS_t) 0.302
## scl(Ag..V2) -0.113 0.083
## Sex -0.960 -0.292 0.064

summary(fit2)

##
## Call:
## lm(formula = scale(leftCEN_Z) ~ scale(RADS_total) + scale(Age.at.V2) +
## Sex, data = df_px_means, na.action = na.exclude)
##
## Residuals:
## Min 1Q Median 3Q Max
## -2.06815 -0.41067 0.01125 0.50257 1.81677
##
## Coefficients:
## Estimate Std. Error t value Pr(>|t|)
## (Intercept) -0.4839 0.5876 -0.824 0.417
## scale(RADS_total) -0.4457 0.1666 -2.675 0.012 *
## scale(Age.at.V2) 0.1547 0.1608 0.962 0.344
## Sex 0.2992 0.3441 0.870 0.391
## ---
## Signif. codes: 0 '***' 0.001 '**' 0.01 '*' 0.05 '.' 0.1 ' ' 1
##
## Residual standard error: 0.9261 on 30 degrees of freedom
## (6 observations deleted due to missingness)
## Multiple R-squared: 0.2203, Adjusted R-squared: 0.1423
## F-statistic: 2.825 on 3 and 30 DF, p-value: 0.05541

summary(fit3)

## Linear mixed model fit by REML. t-tests use Satterthwaite's method [
## lmerModLmerTest]
## Formula: scale(i_day_prop) ~ 1 + scale(RADS_total) + scale(leftCEN_Z) +
## scale(Age.at.V2) + Sex + (1 | id_participant)
## Data: df_aim1_all_language
##
## REML criterion at convergence: 5576.6
##
## Scaled residuals:
## Min 1Q Median 3Q Max
## -3.0957 -0.5692 -0.0126 0.4975 7.5113
##
## Random effects:
## Groups Name Variance Std.Dev.
## id_participant (Intercept) 0.1361 0.3689
## Residual 0.8342 0.9134
## Number of obs: 2070, groups: id_participant, 34
##
## Fixed effects:
## Estimate Std. Error df t value Pr(>|t|)
## (Intercept) -0.02314 0.26941 26.15059 -0.086 0.93222
## scale(RADS_total) 0.30797 0.08562 31.61078 3.597 0.00108 **
## scale(leftCEN_Z) 0.19445 0.08243 31.98907 2.359 0.02459 *
## scale(Age.at.V2) -0.03028 0.07604 26.67876 -0.398 0.69366
## Sex 0.01827 0.15891 27.08035 0.115 0.90933
## ---
## Signif. codes: 0 '***' 0.001 '**' 0.01 '*' 0.05 '.' 0.1 ' ' 1
##
## Correlation of Fixed Effects:
## (Intr) s(RADS s(CEN_ s(A..V
## scl(RADS_t) 0.365
## scl(lCEN_Z) 0.197 0.532
## scl(Ag..V2) -0.165 0.004 -0.112
## Sex -0.961 -0.376 -0.203 0.092

standardize_parameters(fit1)

## # Standardization method: refit
##
## Parameter | Std. Coef. | 95% CI
## ----------------------------------------------
## (Intercept) | 3.64e-03 | [-0.14, 0.14]
## scale(RADS_total) | 0.17 | [ 0.03, 0.30]
## scale(Age.at.V2) | -0.01 | [-0.16, 0.13]
## Sex | 0.06 | [-0.08, 0.21]

standardize_parameters(fit2)

## # Standardization method: refit
##
## Parameter | Std. Coef. | 95% CI
## -----------------------------------------------
## (Intercept) | -8.75e-18 | [-0.32, 0.32]
## scale(RADS_total) | -0.45 | [-0.79, -0.11]
## scale(Age.at.V2) | 0.16 | [-0.17, 0.49]
## Sex | 0.15 | [-0.20, 0.49]

standardize_parameters(fit3)

## # Standardization method: refit
##
## Parameter | Std. Coef. | 95% CI
## ----------------------------------------------
## (Intercept) | 0.02 | [-0.13, 0.16]
## scale(RADS_total) | 0.31 | [ 0.14, 0.47]
## scale(leftCEN_Z) | 0.19 | [ 0.03, 0.36]
## scale(Age.at.V2) | -0.03 | [-0.18, 0.12]
## Sex | 9.08e-03 | [-0.15, 0.16]

fitb <- lmer(scale(i_day_prop) ~ 1 + scale(leftCEN_Z) + (1|id_participant),
 data = df_aim1_all_language,
 na.action=na.exclude)
summary(fitb)

## Linear mixed model fit by REML. t-tests use Satterthwaite's method [
## lmerModLmerTest]
## Formula: scale(i_day_prop) ~ 1 + scale(leftCEN_Z) + (1 | id_participant)
## Data: df_aim1_all_language
##
## REML criterion at convergence: 5582.2
##
## Scaled residuals:
## Min 1Q Median 3Q Max
## -3.1488 -0.5711 -0.0154 0.4973 7.4923
##
## Random effects:
## Groups Name Variance Std.Dev.
## id_participant (Intercept) 0.2007 0.4480
## Residual 0.8346 0.9136
## Number of obs: 2070, groups: id_participant, 34
##
## Fixed effects:
## Estimate Std. Error df t value Pr(>|t|)
## (Intercept) -0.01665 0.08450 29.10141 -0.197 0.845
## scale(leftCEN_Z) 0.04050 0.08171 29.23676 0.496 0.624
##
## Correlation of Fixed Effects:
## (Intr)
## scl(lCEN_Z) -0.038

standardize_parameters(fitb)

## # Standardization method: refit
##
## Parameter | Std. Coef. | 95% CI
## ---------------------------------------------
## (Intercept) | -0.02 | [-0.18, 0.15]
## scale(leftCEN_Z) | 0.04 | [-0.12, 0.20]

# CI for a*b:
c=summary(fit1)$coefficients[2,1]
a = summary(fit2)$coefficients[2,1]
se.a = summary(fit2)$coefficients[2,2]
b = summary(fit3)$coefficients[2,1]
se.b = summary(fit3)$coefficients[2,2]


medci(mu.x=a, mu.y=b, se.x=se.a, se.y=se.b, rho=0, alpha=.05, type="prodclin", plot=TRUE, plotCI=TRUE)

## Warning in arrows(MedCI[[1]][1], yci, MedCI[[1]][2], yci, length = smidge, :
## 'length', 'angle', or 'code' greater than length 1; values after the first are
## ignored


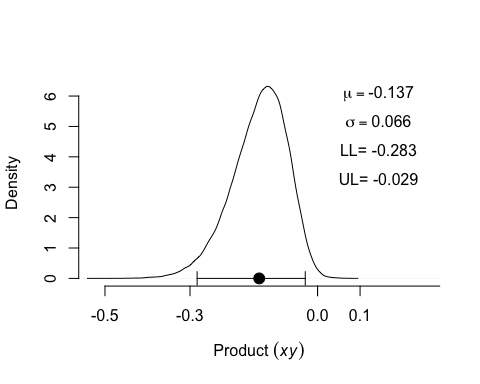


## $`95% CI`
## [1] -0.28326720 -0.02894307
##
## $Estimate
## [1] -0.1372595
##
## $SE
## [1] 0.06551041

## 3b. Dep Sx - aDMN - First-person Pronouns

fit1 <- lmer(scale(i_day_prop) ~ 1 + scale(RADS_total) + (1|id_participant),
 data = df_aim1_all_language,
 na.action=na.exclude)

fit2<- lm(scale(aDMN_Z) ~ scale(RADS_total), df_px_means, na.action=na.exclude)

fit3 <- lmer(scale(i_day_prop) ~ 1 + scale(RADS_total) + scale(aDMN_Z) + (1|id_participant),
 data = df_aim1_all_language,
 na.action=na.exclude)

summary(fit1)

## Linear mixed model fit by REML. t-tests use Satterthwaite's method [
## lmerModLmerTest]
## Formula: scale(i_day_prop) ~ 1 + scale(RADS_total) + (1 | id_participant)
## Data: df_aim1_all_language
##
## REML criterion at convergence: 6084.4
##
## Scaled residuals:
## Min 1Q Median 3Q Max
## -3.1166 -0.5743 -0.0162 0.5058 7.4826
##
## Random effects:
## Groups Name Variance Std.Dev.
## id_participant (Intercept) 0.1380 0.3715
## Residual 0.8432 0.9183
## Number of obs: 2251, groups: id_participant, 40
##
## Fixed effects:
## Estimate Std. Error df t value Pr(>|t|)
## (Intercept) 0.01033 0.06764 32.77133 0.153 0.87959
## scale(RADS_total) 0.18707 0.06387 32.15868 2.929 0.00621 **
## ---
## Signif. codes: 0 '***' 0.001 '**' 0.01 '*' 0.05 '.' 0.1 ' ' 1
##
## Correlation of Fixed Effects:
## (Intr)
## scl(RADS_t) 0.105

summary(fit2)

##
## Call:
## lm(formula = scale(aDMN_Z) ~ scale(RADS_total), data = df_px_means,
## na.action = na.exclude)
##
## Residuals:
## Min 1Q Median 3Q Max
## -2.2644 -0.5644 -0.1297 0.6473 2.5479
##
## Coefficients:
## Estimate Std. Error t value Pr(>|t|)
## (Intercept) 0.01266 0.17032 0.074 0.941
## scale(RADS_total) -0.21607 0.17209 -1.256 0.218
##
## Residual standard error: 0.9914 on 32 degrees of freedom
## (6 observations deleted due to missingness)
## Multiple R-squared: 0.04695, Adjusted R-squared: 0.01717
## F-statistic: 1.577 on 1 and 32 DF, p-value: 0.2183

summary(fit3)

## Linear mixed model fit by REML. t-tests use Satterthwaite's method [
## lmerModLmerTest]
## Formula: scale(i_day_prop) ~ 1 + scale(RADS_total) + scale(aDMN_Z) + (1 |
## id_participant)
## Data: df_aim1_all_language
##
## REML criterion at convergence: 5573.5
##
## Scaled residuals:
## Min 1Q Median 3Q Max
## -3.1295 -0.5621 -0.0136 0.5006 7.5236
##
## Random effects:
## Groups Name Variance Std.Dev.
## id_participant (Intercept) 0.1328 0.3644
## Residual 0.8342 0.9134
## Number of obs: 2070, groups: id_participant, 34
##
## Fixed effects:
## Estimate Std. Error df t value Pr(>|t|)
## (Intercept) 0.005414 0.070760 28.932145 0.077 0.93954
## scale(RADS_total) 0.243982 0.068793 28.898000 3.547 0.00135 **
## scale(aDMN_Z) 0.121800 0.056606 36.975811 2.152 0.03802 *
## ---
## Signif. codes: 0 '***' 0.001 '**' 0.01 '*' 0.05 '.' 0.1 ' ' 1
##
## Correlation of Fixed Effects:
## (Intr) s(RADS
## scl(RADS_t) 0.051
## scal(DMN_Z) 0.020 0.190

standardize_parameters(fit1)

## # Standardization method: refit
##
## Parameter | Std. Coef. | 95% CI
## ----------------------------------------------
## (Intercept) | 0.01 | [-0.12, 0.14]
## scale(RADS_total) | 0.19 | [ 0.06, 0.31]

standardize_parameters(fit2)

## # Standardization method: refit
##
## Parameter | Std. Coef. | 95% CI
## ----------------------------------------------
## (Intercept) | -1.70e-17 | [-0.35, 0.35]
## scale(RADS_total) | -0.22 | [-0.57, 0.13]

standardize_parameters(fit3)

## # Standardization method: refit
##
## Parameter | Std. Coef. | 95% CI
## ----------------------------------------------
## (Intercept) | 0.01 | [-0.12, 0.15]
## scale(RADS_total) | 0.24 | [ 0.11, 0.38]
## scale(aDMN_Z) | 0.12 | [ 0.01, 0.23]

# CI for a*b:
c=summary(fit1)$coefficients[2,1]
a = summary(fit2)$coefficients[2,1]
se.a = summary(fit2)$coefficients[2,2]
b = summary(fit3)$coefficients[2,1]
se.b = summary(fit3)$coefficients[2,2]


medci(mu.x=a, mu.y=b, se.x=se.a, se.y=se.b, rho=0, alpha=.05, type="prodclin", plot=TRUE, plotCI=TRUE)

## Warning in arrows(MedCI[[1]][1], yci, MedCI[[1]][2], yci, length = smidge, :
## 'length', 'angle', or 'code' greater than length 1; values after the first are
## ignored


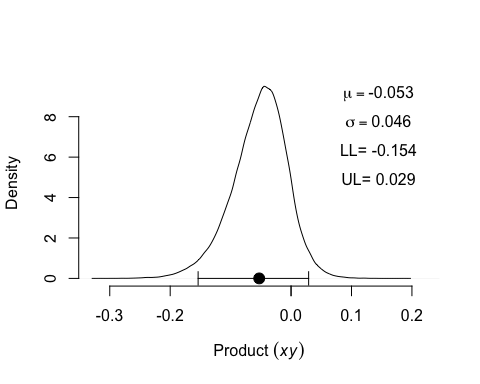


## $`95% CI`
## [1] -0.15379413 0.02907142
##
## $Estimate
## [1] -0.05271808
##
## $SE
## [1] 0.0460856

## 3c. MDD - SN - Negative Emotion Words

fit1 <- lmer(scale(negemo_day_prop) ~ 1 + Group + (1|id_participant),
 data = df_aim1_all_language,
 na.action=na.exclude)

fit2<- lm(scale(SN_CO_Z) ~ Group, df_px_means, na.action=na.exclude)

fit3 <- lmer(scale(negemo_day_prop) ~ 1 + Group + scale(SN_CO_Z) + (1|id_participant),
 data = df_aim1_all_language,
 na.action=na.exclude)

summary(fit1)

## Linear mixed model fit by REML. t-tests use Satterthwaite's method [
## lmerModLmerTest]
## Formula: scale(negemo_day_prop) ~ 1 + Group + (1 | id_participant)
## Data: df_aim1_all_language
##
## REML criterion at convergence: 6176.5
##
## Scaled residuals:
## Min 1Q Median 3Q Max
## -2.1937 -0.5804 -0.0889 0.3796 9.9977
##
## Random effects:
## Groups Name Variance Std.Dev.
## id_participant (Intercept) 0.08636 0.2939
## Residual 0.88466 0.9406
## Number of obs: 2251, groups: id_participant, 40
##
## Fixed effects:
## Estimate Std. Error df t value Pr(>|t|)
## (Intercept) -0.3946 0.1016 37.8636 -3.882 0.000401 ***
## GroupMDD 0.5808 0.1218 36.5820 4.771 2.93e-05 ***
## ---
## Signif. codes: 0 '***' 0.001 '**' 0.01 '*' 0.05 '.' 0.1 ' ' 1
##
## Correlation of Fixed Effects:
## (Intr)
## GroupMDD -0.835

summary(fit2)

##
## Call:
## lm(formula = scale(SN_CO_Z) ~ Group, data = df_px_means, na.action = na.exclude)
##
## Residuals:
## Min 1Q Median 3Q Max
## -1.4715 -0.6868 0.1419 0.4699 2.9623
##
## Coefficients:
## Estimate Std. Error t value Pr(>|t|)
## (Intercept) 0.2864 0.2999 0.955 0.347
## GroupMDD -0.4234 0.3647 -1.161 0.254
##
## Residual standard error: 0.9948 on 32 degrees of freedom
## (6 observations deleted due to missingness)
## Multiple R-squared: 0.04043, Adjusted R-squared: 0.01044
## F-statistic: 1.348 on 1 and 32 DF, p-value: 0.2542

summary(fit3)

## Linear mixed model fit by REML. t-tests use Satterthwaite's method [
## lmerModLmerTest]
## Formula:
## scale(negemo_day_prop) ~ 1 + Group + scale(SN_CO_Z) + (1 | id_participant)
## Data: df_aim1_all_language
##
## REML criterion at convergence: 5629.5
##
## Scaled residuals:
## Min 1Q Median 3Q Max
## -2.2065 -0.5776 -0.0863 0.3760 10.1242
##
## Random effects:
## Groups Name Variance Std.Dev.
## id_participant (Intercept) 0.0818 0.2860
## Residual 0.8628 0.9289
## Number of obs: 2070, groups: id_participant, 34
##
## Fixed effects:
## Estimate Std. Error df t value Pr(>|t|)
## (Intercept) -0.47736 0.11184 28.78852 -4.268 0.000195 ***
## GroupMDD 0.67234 0.13147 28.60636 5.114 1.92e-05 ***
## scale(SN_CO_Z) 0.10736 0.04779 31.96407 2.247 0.031696 *
## ---
## Signif. codes: 0 '***' 0.001 '**' 0.01 '*' 0.05 '.' 0.1 ' ' 1
##
## Correlation of Fixed Effects:
## (Intr) GrpMDD
## GroupMDD -0.853
## sc(SN_CO_Z) -0.187 0.174

standardize_parameters(fit1)

## # Standardization method: refit
##
## Parameter | Std. Coef. | 95% CI
## -----------------------------------------
## (Intercept) | -0.39 | [-0.59, -0.20]
## GroupMDD | 0.58 | [ 0.34, 0.82]

standardize_parameters(fit2)

## # Standardization method: refit
##
## Parameter | Std. Coef. | 95% CI
## ----------------------------------------
## (Intercept) | 0.29 | [-0.32, 0.90]
## GroupMDD | -0.42 | [-1.17, 0.32]

standardize_parameters(fit3)

## # Standardization method: refit
##
## Parameter | Std. Coef. | 95% CI
## --------------------------------------------
## (Intercept) | -0.49 | [-0.71, -0.26]
## GroupMDD | 0.68 | [ 0.42, 0.94]
## scale(SN_CO_Z) | 0.11 | [ 0.01, 0.20]

# CI for a*b:
c=summary(fit1)$coefficients[2,1]
a = summary(fit2)$coefficients[2,1]
se.a = summary(fit2)$coefficients[2,2]
b = summary(fit3)$coefficients[2,1]
se.b = summary(fit3)$coefficients[2,2]


medci(mu.x=a, mu.y=b, se.x=se.a, se.y=se.b, rho=0, alpha=.05, type="prodclin", plot=TRUE, plotCI=TRUE)

## Warning in arrows(MedCI[[1]][1], yci, MedCI[[1]][2], yci, length = smidge, :
## 'length', 'angle', or 'code' greater than length 1; values after the first are
## ignored


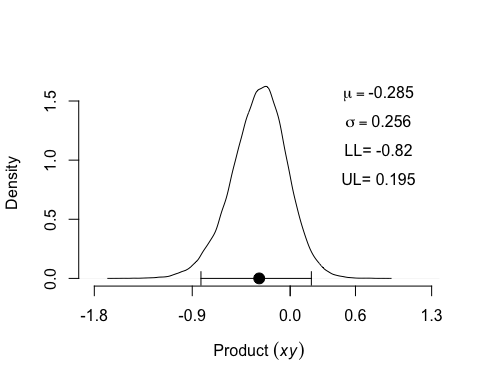


## $`95% CI`
## [1] -0.8199987 0.1946240
##
## $Estimate
## [1] -0.284693
##
## $SE
## [1] 0.2559546

## 3d. Dep Sx - SN - Negative Emotion Words

fit1 <- lmer(scale(negemo_day_prop) ~ 1 + scale(RADS_total) + (1|id_participant),
 data = df_aim1_all_language,
 na.action=na.exclude)

fit2<- lm(scale(SN_CO_Z) ~ scale(RADS_total), df_px_means, na.action=na.exclude)

fit3 <- lmer(scale(negemo_day_prop) ~ 1 + scale(RADS_total) + scale(SN_CO_Z) + (1|id_participant),
 data = df_aim1_all_language,
 na.action=na.exclude)

summary(fit1)

## Linear mixed model fit by REML. t-tests use Satterthwaite's method [
## lmerModLmerTest]
## Formula: scale(negemo_day_prop) ~ 1 + scale(RADS_total) + (1 | id_participant)
## Data: df_aim1_all_language
##
## REML criterion at convergence: 6182
##
## Scaled residuals:
## Min 1Q Median 3Q Max
## -2.2606 -0.5817 -0.0889 0.3822 9.9958
##
## Random effects:
## Groups Name Variance Std.Dev.
## id_participant (Intercept) 0.09523 0.3086
## Residual 0.88517 0.9408
## Number of obs: 2251, groups: id_participant, 40
##
## Fixed effects:
## Estimate Std. Error df t value Pr(>|t|)
## (Intercept) 0.03444 0.05844 33.76574 0.589 0.559581
## scale(RADS_total) 0.23058 0.05508 32.95346 4.186 0.000198 ***
## ---
## Signif. codes: 0 '***' 0.001 '**' 0.01 '*' 0.05 '.' 0.1 ' ' 1
##
## Correlation of Fixed Effects:
## (Intr)
## scl(RADS_t) 0.100

summary(fit2)

##
## Call:
## lm(formula = scale(SN_CO_Z) ~ scale(RADS_total), data = df_px_means,
## na.action = na.exclude)
##
## Residuals:
## Min 1Q Median 3Q Max
## -1.40560 -0.68083 0.01814 0.54676 2.77038
##
## Coefficients:
## Estimate Std. Error t value Pr(>|t|)
## (Intercept) 0.01496 0.16865 0.089 0.930
## scale(RADS_total) -0.25536 0.17039 -1.499 0.144
##
## Residual standard error: 0.9816 on 32 degrees of freedom
## (6 observations deleted due to missingness)
## Multiple R-squared: 0.06558, Adjusted R-squared: 0.03638
## F-statistic: 2.246 on 1 and 32 DF, p-value: 0.1438

summary(fit3)

## Linear mixed model fit by REML. t-tests use Satterthwaite's method [
## lmerModLmerTest]
## Formula: scale(negemo_day_prop) ~ 1 + scale(RADS_total) + scale(SN_CO_Z) +
## (1 | id_participant)
## Data: df_aim1_all_language
##
## REML criterion at convergence: 5633.5
##
## Scaled residuals:
## Min 1Q Median 3Q Max
## -2.2851 -0.5794 -0.0829 0.3822 10.1241
##
## Random effects:
## Groups Name Variance Std.Dev.
## id_participant (Intercept) 0.08297 0.2880
## Residual 0.86365 0.9293
## Number of obs: 2070, groups: id_participant, 34
##
## Fixed effects:
## Estimate Std. Error df t value Pr(>|t|)
## (Intercept) 0.01742 0.05864 25.35441 0.297 0.7689
## scale(RADS_total) 0.28266 0.05822 25.78639 4.855 5.03e-05 ***
## scale(SN_CO_Z) 0.13440 0.04943 30.65808 2.719 0.0107 *
## ---
## Signif. codes: 0 '***' 0.001 '**' 0.01 '*' 0.05 '.' 0.1 ' ' 1
##
## Correlation of Fixed Effects:
## (Intr) s(RADS
## scl(RADS_t) 0.024
## sc(SN_CO_Z) -0.065 0.290

standardize_parameters(fit1)

## # Standardization method: refit
##
## Parameter | Std. Coef. | 95% CI
## ----------------------------------------------
## (Intercept) | 0.03 | [-0.08, 0.15]
## scale(RADS_total) | 0.23 | [ 0.12, 0.34]

standardize_parameters(fit2)

## # Standardization method: refit
##
## Parameter | Std. Coef. | 95% CI
## ----------------------------------------------
## (Intercept) | 2.44e-18 | [-0.34, 0.34]
## scale(RADS_total) | -0.26 | [-0.60, 0.09]

standardize_parameters(fit3)

## # Standardization method: refit
##
## Parameter | Std. Coef. | 95% CI
## ----------------------------------------------
## (Intercept) | 0.02 | [-0.09, 0.14]
## scale(RADS_total) | 0.28 | [ 0.17, 0.40]
## scale(SN_CO_Z) | 0.14 | [ 0.04, 0.23]

# CI for a*b:
c=summary(fit1)$coefficients[2,1]
a = summary(fit2)$coefficients[2,1]
se.a = summary(fit2)$coefficients[2,2]
b = summary(fit3)$coefficients[2,1]
se.b = summary(fit3)$coefficients[2,2]


medci(mu.x=a, mu.y=b, se.x=se.a, se.y=se.b, rho=0, alpha=.05, type="prodclin", plot=TRUE, plotCI=TRUE)

## Warning in arrows(MedCI[[1]][1], yci, MedCI[[1]][2], yci, length = smidge, :
## 'length', 'angle', or 'code' greater than length 1; values after the first are
## ignored


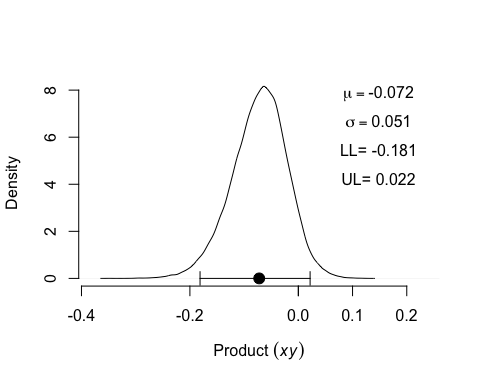


## $`95% CI`
## [1] -0.18129062 0.02171149
##
## $Estimate
## [1] -0.07218029
##
## $SE
## [1] 0.05137269

## 3e. MDD - aDMN - Future-focus Words

fit1 <- lmer(scale(focusfuture_day_prop) ~ 1 + Group + (1|id_participant),
 data = df_aim1_all_language,
 na.action=na.exclude)

fit2<- lm(scale(aDMN_Z) ~ Group, df_px_means, na.action=na.exclude)

fit3 <- lmer(scale(focusfuture_day_prop) ~ 1 + Group + scale(aDMN_Z) + (1|id_participant),
 data = df_aim1_all_language,
 na.action=na.exclude)

summary(fit1)

## Linear mixed model fit by REML. t-tests use Satterthwaite's method [
## lmerModLmerTest]
## Formula: scale(focusfuture_day_prop) ~ 1 + Group + (1 | id_participant)
## Data: df_aim1_all_language
##
## REML criterion at convergence: 6262.2
##
## Scaled residuals:
## Min 1Q Median 3Q Max
## -1.9359 -0.6197 -0.0888 0.4275 8.0243
##
## Random effects:
## Groups Name Variance Std.Dev.
## id_participant (Intercept) 0.09232 0.3038
## Residual 0.91871 0.9585
## Number of obs: 2251, groups: id_participant, 40
##
## Fixed effects:
## Estimate Std. Error df t value Pr(>|t|)
## (Intercept) 0.2595 0.1047 28.4992 2.478 0.0194 *
## GroupMDD -0.2739 0.1255 27.5340 -2.183 0.0377 *
## ---
## Signif. codes: 0 '***' 0.001 '**' 0.01 '*' 0.05 '.' 0.1 ' ' 1
##
## Correlation of Fixed Effects:
## (Intr)
## GroupMDD -0.835

summary(fit2)

##
## Call:
## lm(formula = scale(aDMN_Z) ~ Group, data = df_px_means, na.action = na.exclude)
##
## Residuals:
## Min 1Q Median 3Q Max
## -2.10460 -0.64853 -0.09011 0.71425 2.57399
##
## Coefficients:
## Estimate Std. Error t value Pr(>|t|)
## (Intercept) 0.2369 0.3019 0.785 0.438
## GroupMDD -0.3502 0.3671 -0.954 0.347
##
## Residual standard error: 1.001 on 32 degrees of freedom
## (6 observations deleted due to missingness)
## Multiple R-squared: 0.02765, Adjusted R-squared: -0.002733
## F-statistic: 0.9101 on 1 and 32 DF, p-value: 0.3473

summary(fit3)

## Linear mixed model fit by REML. t-tests use Satterthwaite's method [
## lmerModLmerTest]
## Formula: scale(focusfuture_day_prop) ~ 1 + Group + scale(aDMN_Z) + (1 |
## id_participant)
## Data: df_aim1_all_language
##
## REML criterion at convergence: 5710.3
##
## Scaled residuals:
## Min 1Q Median 3Q Max
## -1.9513 -0.6115 -0.0903 0.4230 8.0910
##
## Random effects:
## Groups Name Variance Std.Dev.
## id_participant (Intercept) 0.09598 0.3098
## Residual 0.89602 0.9466
## Number of obs: 2070, groups: id_participant, 34
##
## Fixed effects:
## Estimate Std. Error df t value Pr(>|t|)
## (Intercept) 0.27125 0.11776 25.88128 2.303 0.0295 *
## GroupMDD -0.26042 0.13913 25.48264 -1.872 0.0728 .
## scale(aDMN_Z) 0.10605 0.05005 31.58817 2.119 0.0421 *
## ---
## Signif. codes: 0 '***' 0.001 '**' 0.01 '*' 0.05 '.' 0.1 ' ' 1
##
## Correlation of Fixed Effects:
## (Intr) GrpMDD
## GroupMDD -0.849
## scal(DMN_Z) -0.077 0.100

standardize_parameters(fit1)

## # Standardization method: refit
##
## Parameter | Std. Coef. | 95% CI
## -----------------------------------------
## (Intercept) | 0.26 | [ 0.05, 0.46]
## GroupMDD | -0.27 | [-0.52, -0.03]

standardize_parameters(fit2)

## # Standardization method: refit
##
## Parameter | Std. Coef. | 95% CI
## ----------------------------------------
## (Intercept) | 0.24 | [-0.38, 0.85]
## GroupMDD | -0.35 | [-1.10, 0.40]

standardize_parameters(fit3)

## # Standardization method: refit
##
## Parameter | Std. Coef. | 95% CI
## ------------------------------------------
## (Intercept) | 0.27 | [ 0.04, 0.51]
## GroupMDD | -0.26 | [-0.54, 0.01]
## scale(aDMN_Z) | 0.11 | [ 0.01, 0.21]

# CI for a*b:
c=summary(fit1)$coefficients[2,1]
a = summary(fit2)$coefficients[2,1]
se.a = summary(fit2)$coefficients[2,2]
b = summary(fit3)$coefficients[2,1]
se.b = summary(fit3)$coefficients[2,2]


medci(mu.x=a, mu.y=b, se.x=se.a, se.y=se.b, rho=0, alpha=.05, type="prodclin", plot=TRUE, plotCI=TRUE)

## Warning in arrows(MedCI[[1]][1], yci, MedCI[[1]][2], yci, length = smidge, :
## 'length', 'angle', or 'code' greater than length 1; values after the first are
## ignored


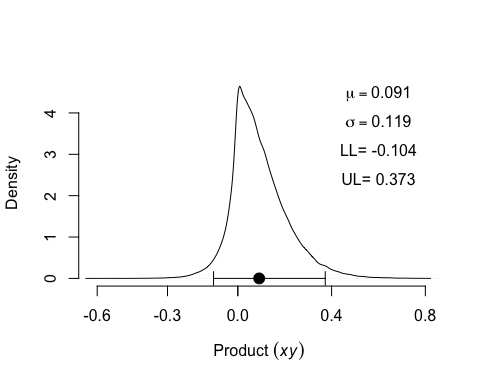


## $`95% CI`
## [1] -0.1036954 0.3729873
##
## $Estimate
## [1] 0.09119688
##
## $SE
## [1] 0.1188317

## 3f. Dep Sx - aDMN - Future-focus Words

fit1 <- lmer(scale(focusfuture_day_prop) ~ 1 + scale(RADS_total) + (1|id_participant),
 data = df_aim1_all_language,
 na.action=na.exclude)

fit2<- lm(scale(aDMN_Z) ~ scale(RADS_total), df_px_means, na.action=na.exclude)

fit3 <- lmer(scale(focusfuture_day_prop) ~ 1 + scale(RADS_total) + scale(aDMN_Z) + (1|id_participant),
 data = df_aim1_all_language,
 na.action=na.exclude)

summary(fit1)

## Linear mixed model fit by REML. t-tests use Satterthwaite's method [
## lmerModLmerTest]
## Formula:
## scale(focusfuture_day_prop) ~ 1 + scale(RADS_total) + (1 | id_participant)
## Data: df_aim1_all_language
##
## REML criterion at convergence: 6263.3
##
## Scaled residuals:
## Min 1Q Median 3Q Max
## -1.9146 -0.6200 -0.0865 0.4261 8.0308
##
## Random effects:
## Groups Name Variance Std.Dev.
## id_participant (Intercept) 0.09307 0.3051
## Residual 0.91839 0.9583
## Number of obs: 2251, groups: id_participant, 40
##
## Fixed effects:
## Estimate Std. Error df t value Pr(>|t|)
## (Intercept) 0.05538 0.05815 26.73857 0.953 0.3494
## scale(RADS_total) -0.12603 0.05479 26.08237 -2.300 0.0297 *
## ---
## Signif. codes: 0 '***' 0.001 '**' 0.01 '*' 0.05 '.' 0.1 ' ' 1
##
## Correlation of Fixed Effects:
## (Intr)
## scl(RADS_t) 0.100

summary(fit2)

##
## Call:
## lm(formula = scale(aDMN_Z) ~ scale(RADS_total), data = df_px_means,
## na.action = na.exclude)
##
## Residuals:
## Min 1Q Median 3Q Max
## -2.2644 -0.5644 -0.1297 0.6473 2.5479
##
## Coefficients:
## Estimate Std. Error t value Pr(>|t|)
## (Intercept) 0.01266 0.17032 0.074 0.941
## scale(RADS_total) -0.21607 0.17209 -1.256 0.218
##
## Residual standard error: 0.9914 on 32 degrees of freedom
## (6 observations deleted due to missingness)
## Multiple R-squared: 0.04695, Adjusted R-squared: 0.01717
## F-statistic: 1.577 on 1 and 32 DF, p-value: 0.2183

summary(fit3)

## Linear mixed model fit by REML. t-tests use Satterthwaite's method [
## lmerModLmerTest]
## Formula: scale(focusfuture_day_prop) ~ 1 + scale(RADS_total) + scale(aDMN_Z) +
## (1 | id_participant)
## Data: df_aim1_all_language
##
## REML criterion at convergence: 5713
##
## Scaled residuals:
## Min 1Q Median 3Q Max
## -1.9431 -0.6124 -0.0927 0.4208 8.1051
##
## Random effects:
## Groups Name Variance Std.Dev.
## id_participant (Intercept) 0.1015 0.3186
## Residual 0.8959 0.9465
## Number of obs: 2070, groups: id_participant, 34
##
## Fixed effects:
## Estimate Std. Error df t value Pr(>|t|)
## (Intercept) 0.07995 0.06370 24.82279 1.255 0.2211
## scale(RADS_total) -0.09575 0.06193 24.90158 -1.546 0.1347
## scale(aDMN_Z) 0.10078 0.05184 32.74061 1.944 0.0605 .
## ---
## Signif. codes: 0 '***' 0.001 '**' 0.01 '*' 0.05 '.' 0.1 ' ' 1
##
## Correlation of Fixed Effects:
## (Intr) s(RADS
## scl(RADS_t) 0.049
## scal(DMN_Z) 0.025 0.199

standardize_parameters(fit1)

## # Standardization method: refit
##
## Parameter | Std. Coef. | 95% CI
## -----------------------------------------------
## (Intercept) | 0.06 | [-0.06, 0.17]
## scale(RADS_total) | -0.13 | [-0.23, -0.02]

standardize_parameters(fit2)

## # Standardization method: refit
##
## Parameter | Std. Coef. | 95% CI
## ----------------------------------------------
## (Intercept) | -1.70e-17 | [-0.35, 0.35]
## scale(RADS_total) | -0.22 | [-0.57, 0.13]

standardize_parameters(fit3)

## # Standardization method: refit
##
## Parameter | Std. Coef. | 95% CI
## ----------------------------------------------
## (Intercept) | 0.08 | [-0.05, 0.20]
## scale(RADS_total) | -0.10 | [-0.22, 0.03]
## scale(aDMN_Z) | 0.10 | [ 0.00, 0.20]

# CI for a*b:
c=summary(fit1)$coefficients[2,1]
a = summary(fit2)$coefficients[2,1]
se.a = summary(fit2)$coefficients[2,2]
b = summary(fit3)$coefficients[2,1]
se.b = summary(fit3)$coefficients[2,2]


medci(mu.x=a, mu.y=b, se.x=se.a, se.y=se.b, rho=0, alpha=.05, type="prodclin", plot=TRUE, plotCI=TRUE)

## Warning in arrows(MedCI[[1]][1], yci, MedCI[[1]][2], yci, length = smidge, :
## 'length', 'angle', or 'code' greater than length 1; values after the first are
## ignored


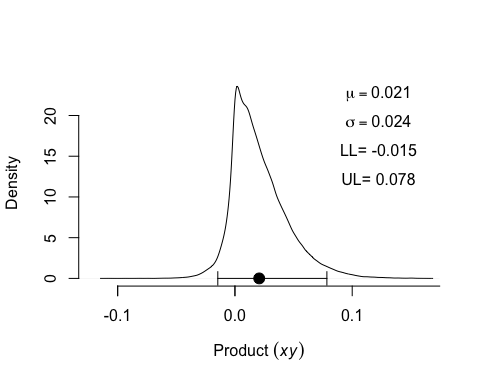


## $`95% CI`
## [1] -0.01461790 0.07840343
##
## $Estimate
## [1] 0.02068874
##
## $SE
## [1] 0.02375086

# 4. AIM 4 - Does intrinsic connectivity moderate the association between depression and depression-related linguistic features of smartphone use?

## S4a. Dep Sx - First-person pronouns

### S4a.1 Within-Network aDMN

modelS4a.1 <- lmer(formula = i_day_prop ~
 aDMN_Z*RADS_total +
 (1 |id_participant),
 data = df_aim1_all_language,
 na.action=na.exclude)
summary(modelS4a.1)

## Linear mixed model fit by REML. t-tests use Satterthwaite's method [
## lmerModLmerTest]
## Formula: i_day_prop ~ aDMN_Z * RADS_total + (1 | id_participant)
## Data: df_aim1_all_language
##
## REML criterion at convergence: 10988.4
##
## Scaled residuals:
## Min 1Q Median 3Q Max
## -3.1296 -0.5653 -0.0137 0.5010 7.5196
##
## Random effects:
## Groups Name Variance Std.Dev.
## id_participant (Intercept) 1.831 1.353
## Residual 11.415 3.379
## Number of obs: 2070, groups: id_participant, 34
##
## Fixed effects:
## Estimate Std. Error df t value Pr(>|t|)
## (Intercept) -1.38262 4.14626 30.16524 -0.333 0.7411
## aDMN_Z 3.91399 2.53509 31.44673 1.544 0.1326
## RADS_total 0.10001 0.05808 29.77035 1.722 0.0955 .
## aDMN_Z:RADS_total -0.03365 0.03654 31.43599 -0.921 0.3641
## ---
## Signif. codes: 0 '***' 0.001 '**' 0.01 '*' 0.05 '.' 0.1 ' ' 1
##
## Correlation of Fixed Effects:
## (Intr) aDMN_Z RADS_t
## aDMN_Z -0.970
## RADS_total -0.955 0.938
## aDMN_Z:RADS 0.911 -0.950 -0.972

standardize_parameters(modelS4a.1)

## # Standardization method: refit
##
## Parameter | Std. Coef. | 95% CI
## ----------------------------------------------
## (Intercept) | 3.37e-03 | [-0.14, 0.14]
## aDMN_Z | 0.10 | [-0.02, 0.22]
## RADS_total | 0.25 | [ 0.11, 0.38]
## aDMN_Z:RADS_total | -0.05 | [-0.14, 0.05]

### S4a.2 Within-Network leftCEN_Z

modelS4a.2 <- lmer(formula = i_day_prop ~
 leftCEN_Z*RADS_total +
 (1 |id_participant),
 data = df_aim1_all_language,
 na.action=na.exclude)
summary(modelS4a.2)

## Linear mixed model fit by REML. t-tests use Satterthwaite's method [
## lmerModLmerTest]
## Formula: i_day_prop ~ leftCEN_Z * RADS_total + (1 | id_participant)
## Data: df_aim1_all_language
##
## REML criterion at convergence: 10985.2
##
## Scaled residuals:
## Min 1Q Median 3Q Max
## -3.0861 -0.5698 -0.0093 0.5010 7.4955
##
## Random effects:
## Groups Name Variance Std.Dev.
## id_participant (Intercept) 1.651 1.285
## Residual 11.414 3.378
## Number of obs: 2070, groups: id_participant, 34
##
## Fixed effects:
## Estimate Std. Error df t value Pr(>|t|)
## (Intercept) -9.59342 6.34936 32.21670 -1.511 0.1406
## leftCEN_Z 6.48250 2.89661 30.25529 2.238 0.0327 *
## RADS_total 0.18475 0.08131 30.17823 2.272 0.0304 *
## leftCEN_Z:RADS_total -0.05940 0.03850 28.77038 -1.543 0.1338
## ---
## Signif. codes: 0 '***' 0.001 '**' 0.01 '*' 0.05 '.' 0.1 ' ' 1
##
## Correlation of Fixed Effects:
## (Intr) lfCEN_Z RADS_t
## leftCEN_Z -0.987
## RADS_total -0.963 0.964
## lCEN_Z:RADS 0.923 -0.951 -0.983

standardize_parameters(modelS4a.2)

## # Standardization method: refit
##
## Parameter | Std. Coef. | 95% CI
## -------------------------------------------------
## (Intercept) | -0.04 | [-0.18, 0.11]
## leftCEN_Z | 0.18 | [ 0.03, 0.33]
## RADS_total | 0.37 | [ 0.20, 0.53]
## leftCEN_Z:RADS_total | -0.10 | [-0.22, 0.03]

### S4a.3 Between-Network admn_lcen

modelS4a.3 <- lmer(formula = i_day_prop ~
 admn_lcen*RADS_total +
 (1 |id_participant),
 data = df_aim1_all_language,
 na.action=na.exclude)
summary(modelS4a.3)

## Linear mixed model fit by REML. t-tests use Satterthwaite's method [
## lmerModLmerTest]
## Formula: i_day_prop ~ admn_lcen * RADS_total + (1 | id_participant)
## Data: df_aim1_all_language
##
## REML criterion at convergence: 10882.9
##
## Scaled residuals:
## Min 1Q Median 3Q Max
## -3.1656 -0.5685 -0.0126 0.5039 7.5617
##
## Random effects:
## Groups Name Variance Std.Dev.
## id_participant (Intercept) 2.145 1.465
## Residual 11.293 3.360
## Number of obs: 2054, groups: id_participant, 32
##
## Fixed effects:
## Estimate Std. Error df t value Pr(>|t|)
## (Intercept) 6.21986 2.00329 34.12353 3.105 0.00382 **
## admn_lcen -3.33305 5.28567 34.16732 -0.631 0.53251
## RADS_total 0.02820 0.02591 31.68824 1.088 0.28468
## admn_lcen:RADS_total 0.04034 0.06309 32.66386 0.639 0.52702
## ---
## Signif. codes: 0 '***' 0.001 '**' 0.01 '*' 0.05 '.' 0.1 ' ' 1
##
## Correlation of Fixed Effects:
## (Intr) admn_l RADS_t
## admn_lcen -0.834
## RADS_total -0.973 0.784
## admn_:RADS_ 0.838 -0.980 -0.824

standardize_parameters(modelS4a.3)

## # Standardization method: refit
##
## Parameter | Std. Coef. | 95% CI
## -------------------------------------------------
## (Intercept) | -3.80e-04 | [-0.16, 0.16]
## admn_lcen | -0.02 | [-0.19, 0.14]
## RADS_total | 0.23 | [ 0.07, 0.38]
## admn_lcen:RADS_total | 0.06 | [-0.11, 0.22]

### S4a.4 Between-Network admn_rcen

modelS4a.4 <- lmer(formula = i_day_prop ~
 admn_rcen*RADS_total +
 (1 |id_participant),
 data = df_aim1_all_language,
 na.action=na.exclude)
summary(modelS4a.4)

## Linear mixed model fit by REML. t-tests use Satterthwaite's method [
## lmerModLmerTest]
## Formula: i_day_prop ~ admn_rcen * RADS_total + (1 | id_participant)
## Data: df_aim1_all_language
##
## REML criterion at convergence: 10023.9
##
## Scaled residuals:
## Min 1Q Median 3Q Max
## -3.3686 -0.5742 -0.0044 0.5225 4.6347
##
## Random effects:
## Groups Name Variance Std.Dev.
## id_participant (Intercept) 1.916 1.384
## Residual 9.941 3.153
## Number of obs: 1938, groups: id_participant, 31
##
## Fixed effects:
## Estimate Std. Error df t value Pr(>|t|)
## (Intercept) 7.17889 2.30289 25.00379 3.117 0.00455 **
## admn_rcen -5.64161 3.75127 24.10202 -1.504 0.14559
## RADS_total 0.02097 0.02808 23.99355 0.747 0.46232
## admn_rcen:RADS_total 0.06064 0.04215 23.54606 1.438 0.16346
## ---
## Signif. codes: 0 '***' 0.001 '**' 0.01 '*' 0.05 '.' 0.1 ' ' 1
##
## Correlation of Fixed Effects:
## (Intr) admn_r RADS_t
## admn_rcen -0.862
## RADS_total -0.961 0.779
## admn_:RADS_ 0.861 -0.970 -0.837

standardize_parameters(modelS4a.4)

## # Standardization method: refit
##
## Parameter | Std. Coef. | 95% CI
## -------------------------------------------------
## (Intercept) | 0.01 | [-0.15, 0.17]
## admn_rcen | -0.09 | [-0.27, 0.09]
## RADS_total | 0.27 | [ 0.13, 0.41]
## admn_rcen:RADS_total | 0.09 | [-0.03, 0.21]

### S4a.5 Between-Network admn_sn

modelS4a.5 <- lmer(formula = i_day_prop ~
 admn_sn*RADS_total +
 (1 |id_participant),
 data = df_aim1_all_language,
 na.action=na.exclude)
summary(modelS4a.5)

## Linear mixed model fit by REML. t-tests use Satterthwaite's method [
## lmerModLmerTest]
## Formula: i_day_prop ~ admn_sn * RADS_total + (1 | id_participant)
## Data: df_aim1_all_language
##
## REML criterion at convergence: 10024.7
##
## Scaled residuals:
## Min 1Q Median 3Q Max
## -3.3771 -0.5749 -0.0029 0.5261 4.6344
##
## Random effects:
## Groups Name Variance Std.Dev.
## id_participant (Intercept) 1.984 1.409
## Residual 9.944 3.153
## Number of obs: 1938, groups: id_participant, 31
##
## Fixed effects:
## Estimate Std. Error df t value Pr(>|t|)
## (Intercept) 4.77463 1.29058 24.45461 3.700 0.0011 **
## admn_sn -4.25456 4.18771 23.22906 -1.016 0.3201
## RADS_total 0.04582 0.01720 23.77282 2.664 0.0136 *
## admn_sn:RADS_total 0.05240 0.05252 22.97714 0.998 0.3288
## ---
## Signif. codes: 0 '***' 0.001 '**' 0.01 '*' 0.05 '.' 0.1 ' ' 1
##
## Correlation of Fixed Effects:
## (Intr) admn_s RADS_t
## admn_sn -0.428
## RADS_total -0.965 0.411
## admn_:RADS_ 0.439 -0.977 -0.455

standardize_parameters(modelS4a.5)

## # Standardization method: refit
##
## Parameter | Std. Coef. | 95% CI
## -----------------------------------------------
## (Intercept) | 0.03 | [-0.13, 0.19]
## admn_sn | -0.02 | [-0.16, 0.12]
## RADS_total | 0.26 | [ 0.12, 0.40]
## admn_sn:RADS_total | 0.07 | [-0.07, 0.20]

### S4a.6 Between-Network sn_lcen

modelS4a.6 <- lmer(formula = i_day_prop ~
 sn_lcen*RADS_total +
 (1 |id_participant),
 data = df_aim1_all_language,
 na.action=na.exclude)
summary(modelS4a.6)

## Linear mixed model fit by REML. t-tests use Satterthwaite's method [
## lmerModLmerTest]
## Formula: i_day_prop ~ sn_lcen * RADS_total + (1 | id_participant)
## Data: df_aim1_all_language
##
## REML criterion at convergence: 10991.6
##
## Scaled residuals:
## Min 1Q Median 3Q Max
## -3.1310 -0.5663 -0.0102 0.5017 7.5179
##
## Random effects:
## Groups Name Variance Std.Dev.
## id_participant (Intercept) 2.112 1.453
## Residual 11.422 3.380
## Number of obs: 2070, groups: id_participant, 34
##
## Fixed effects:
## Estimate Std. Error df t value Pr(>|t|)
## (Intercept) 4.11600 2.19508 29.23485 1.875 0.0708 .
## sn_lcen 1.86650 4.18752 28.26412 0.446 0.6592
## RADS_total 0.05067 0.02838 29.22848 1.785 0.0846 .
## sn_lcen:RADS_total -0.01373 0.05575 27.93592 -0.246 0.8073
## ---
## Signif. codes: 0 '***' 0.001 '**' 0.01 '*' 0.05 '.' 0.1 ' ' 1
##
## Correlation of Fixed Effects:
## (Intr) sn_lcn RADS_t
## sn_lcen -0.873
## RADS_total -0.973 0.853
## sn_lc:RADS_ 0.828 -0.962 -0.862

standardize_parameters(modelS4a.6)

## # Standardization method: refit
##
## Parameter | Std. Coef. | 95% CI
## -----------------------------------------------
## (Intercept) | 0.02 | [-0.13, 0.18]
## sn_lcen | 0.05 | [-0.08, 0.18]
## RADS_total | 0.23 | [ 0.08, 0.37]
## sn_lcen:RADS_total | -0.02 | [-0.14, 0.11]

## 4b. MDD - Negative Emotion Words

### 4b.1 Within-Network SN_CO_Z

model4b.1 <- lmer(formula = negemo_day_prop ~
 SN_CO_Z*Group +
 (1 |id_participant),
 data = df_aim1_all_language,
 na.action=na.exclude)
summary(model4b.1)

## Linear mixed model fit by REML. t-tests use Satterthwaite's method [
## lmerModLmerTest]
## Formula: negemo_day_prop ~ SN_CO_Z * Group + (1 | id_participant)
## Data: df_aim1_all_language
##
## REML criterion at convergence: 7487.5
##
## Scaled residuals:
## Min 1Q Median 3Q Max
## -2.2108 -0.5794 -0.0893 0.3731 10.1230
##
## Random effects:
## Groups Name Variance Std.Dev.
## id_participant (Intercept) 0.2051 0.4529
## Residual 2.1229 1.4570
## Number of obs: 2070, groups: id_participant, 34
##
## Fixed effects:
## Estimate Std. Error df t value Pr(>|t|)
## (Intercept) -0.3157 1.0715 30.5965 -0.295 0.7702
## SN_CO_Z 0.7011 0.3990 30.9416 1.757 0.0888 .
## GroupMDD 1.8360 1.2639 30.3714 1.453 0.1566
## SN_CO_Z:GroupMDD -0.3003 0.4795 30.8715 -0.626 0.5358
## ---
## Signif. codes: 0 '***' 0.001 '**' 0.01 '*' 0.05 '.' 0.1 ' ' 1
##
## Correlation of Fixed Effects:
## (Intr) SN_CO_Z GrpMDD
## SN_CO_Z -0.987
## GroupMDD -0.848 0.837
## SN_CO_Z:GMD 0.821 -0.832 -0.986

standardize_parameters(model4b.1)

## # Standardization method: refit
##
## Parameter | Std. Coef. | 95% CI
## ----------------------------------------------
## (Intercept) | -0.51 | [-0.74, -0.27]
## SN_CO_Z | 0.15 | [-0.02, 0.32]
## GroupMDD | 0.70 | [ 0.43, 0.96]
## SN_CO_Z:GroupMDD | -0.07 | [-0.27, 0.14]

### 4b.2 Between-Network admn_sn

model4b.2 <- lmer(formula = negemo_day_prop ~
 admn_sn*Group +
 (1 |id_participant),
 data = df_aim1_all_language,
 na.action=na.exclude)
summary(model4b.2)

## Linear mixed model fit by REML. t-tests use Satterthwaite's method [
## lmerModLmerTest]
## Formula: negemo_day_prop ~ admn_sn * Group + (1 | id_participant)
## Data: df_aim1_all_language
##
## REML criterion at convergence: 7009.5
##
## Scaled residuals:
## Min 1Q Median 3Q Max
## -2.2318 -0.5563 -0.0847 0.3712 10.1213
##
## Random effects:
## Groups Name Variance Std.Dev.
## id_participant (Intercept) 0.2353 0.4851
## Residual 2.1210 1.4564
## Number of obs: 1938, groups: id_participant, 31
##
## Fixed effects:
## Estimate Std. Error df t value Pr(>|t|)
## (Intercept) 1.5731 0.2208 27.7210 7.123 1e-07 ***
## admn_sn 0.8881 0.5695 24.3422 1.559 0.13183
## GroupMDD 0.9608 0.2666 26.4972 3.603 0.00128 **
## admn_sn:GroupMDD -0.9486 0.6884 24.2787 -1.378 0.18076
## ---
## Signif. codes: 0 '***' 0.001 '**' 0.01 '*' 0.05 '.' 0.1 ' ' 1
##
## Correlation of Fixed Effects:
## (Intr) admn_s GrpMDD
## admn_sn -0.316
## GroupMDD -0.828 0.262
## admn_s:GMDD 0.262 -0.827 -0.419

standardize_parameters(model4b.2)

## # Standardization method: refit
##
## Parameter | Std. Coef. | 95% CI
## ----------------------------------------------
## (Intercept) | -0.35 | [-0.62, -0.07]
## admn_sn | 0.16 | [-0.04, 0.37]
## GroupMDD | 0.50 | [ 0.19, 0.81]
## admn_sn:GroupMDD | -0.18 | [-0.43, 0.07]

### 4b.3 Between-Network sn_lcen

model4b.3 <- lmer(formula = negemo_day_prop ~
 sn_lcen*Group +
 (1 |id_participant),
 data = df_aim1_all_language,
 na.action=na.exclude)
summary(model4b.3)

## Linear mixed model fit by REML. t-tests use Satterthwaite's method [
## lmerModLmerTest]
## Formula: negemo_day_prop ~ sn_lcen * Group + (1 | id_participant)
## Data: df_aim1_all_language
##
## REML criterion at convergence: 7485.8
##
## Scaled residuals:
## Min 1Q Median 3Q Max
## -2.2121 -0.5773 -0.0897 0.3700 10.1159
##
## Random effects:
## Groups Name Variance Std.Dev.
## id_participant (Intercept) 0.2163 0.4651
## Residual 2.1224 1.4569
## Number of obs: 2070, groups: id_participant, 34
##
## Fixed effects:
## Estimate Std. Error df t value Pr(>|t|)
## (Intercept) 0.8543 0.3898 32.9573 2.191 0.035603 *
## sn_lcen 1.4750 0.7463 30.6850 1.976 0.057149 .
## GroupMDD 1.7855 0.4349 32.3227 4.106 0.000256 ***
## sn_lcen:GroupMDD -1.8281 0.8749 29.7908 -2.089 0.045314 *
## ---
## Signif. codes: 0 '***' 0.001 '**' 0.01 '*' 0.05 '.' 0.1 ' ' 1
##
## Correlation of Fixed Effects:
## (Intr) sn_lcn GrpMDD
## sn_lcen -0.891
## GroupMDD -0.896 0.798
## sn_lcn:GMDD 0.760 -0.853 -0.870

standardize_parameters(model4b.3)

## # Standardization method: refit
##
## Parameter | Std. Coef. | 95% CI
## ----------------------------------------------
## (Intercept) | -0.47 | [-0.69, -0.24]
## sn_lcen | 0.21 | [ 0.00, 0.42]
## GroupMDD | 0.63 | [ 0.36, 0.90]
## sn_lcen:GroupMDD | -0.26 | [-0.50, -0.02]

### 4b.4 Between-Network sn_rcen

model4b.4 <- lmer(formula = negemo_day_prop ~
 sn_rcen*Group +
 (1 |id_participant),
 data = df_aim1_all_language,
 na.action=na.exclude)
summary(model4b.4)

## Linear mixed model fit by REML. t-tests use Satterthwaite's method [
## lmerModLmerTest]
## Formula: negemo_day_prop ~ sn_rcen * Group + (1 | id_participant)
## Data: df_aim1_all_language
##
## REML criterion at convergence: 7485.4
##
## Scaled residuals:
## Min 1Q Median 3Q Max
## -2.2129 -0.5816 -0.0899 0.3740 10.1150
##
## Random effects:
## Groups Name Variance Std.Dev.
## id_participant (Intercept) 0.2116 0.460
## Residual 2.1225 1.457
## Number of obs: 2070, groups: id_participant, 34
##
## Fixed effects:
## Estimate Std. Error df t value Pr(>|t|)
## (Intercept) 0.7790 0.4132 25.5073 1.885 0.070818 .
## sn_rcen 1.7357 0.8515 24.1289 2.038 0.052608 .
## GroupMDD 1.8582 0.4469 25.8942 4.158 0.000311 ***
## sn_rcen:GroupMDD -2.0991 0.9388 24.4947 -2.236 0.034721 *
## ---
## Signif. codes: 0 '***' 0.001 '**' 0.01 '*' 0.05 '.' 0.1 ' ' 1
##
## Correlation of Fixed Effects:
## (Intr) sn_rcn GrpMDD
## sn_rcen -0.905
## GroupMDD -0.925 0.837
## sn_rcn:GMDD 0.821 -0.907 -0.882

standardize_parameters(model4b.4)

## # Standardization method: refit
##
## Parameter | Std. Coef. | 95% CI
## ----------------------------------------------
## (Intercept) | -0.49 | [-0.71, -0.26]
## sn_rcen | 0.26 | [ 0.01, 0.50]
## GroupMDD | 0.66 | [ 0.39, 0.93]
## sn_rcen:GroupMDD | -0.31 | [-0.58, -0.04]

## S4b. Dep Sx - Negative Emotion Words

### S4b.1 Within-Network SN_CO_Z

modelS4b.1 <- lmer(formula = negemo_day_prop ~
 SN_CO_Z*RADS_total +
 (1 |id_participant),
 data = df_aim1_all_language,
 na.action=na.exclude)
summary(modelS4b.1)

## Linear mixed model fit by REML. t-tests use Satterthwaite's method [
## lmerModLmerTest]
## Formula: negemo_day_prop ~ SN_CO_Z * RADS_total + (1 | id_participant)
## Data: df_aim1_all_language
##
## REML criterion at convergence: 7505
##
## Scaled residuals:
## Min 1Q Median 3Q Max
## -2.2877 -0.5788 -0.0825 0.3817 10.1223
##
## Random effects:
## Groups Name Variance Std.Dev.
## id_participant (Intercept) 0.2138 0.4624
## Residual 2.1248 1.4577
## Number of obs: 2070, groups: id_participant, 34
##
## Fixed effects:
## Estimate Std. Error df t value Pr(>|t|)
## (Intercept) -1.1665995 2.3141828 28.1545282 -0.504 0.618
## SN_CO_Z 0.6684393 0.8994224 28.3033366 0.743 0.463
## RADS_total 0.0254791 0.0315476 27.2325328 0.808 0.426
## SN_CO_Z:RADS_total -0.0007653 0.0125824 27.5844749 -0.061 0.952
##
## Correlation of Fixed Effects:
## (Intr) SN_CO_Z RADS_t
## SN_CO_Z -0.987
## RADS_total -0.966 0.966
## SN_CO_Z:RAD 0.942 -0.966 -0.988

standardize_parameters(modelS4b.1)

## # Standardization method: refit
##
## Parameter | Std. Coef. | 95% CI
## -----------------------------------------------
## (Intercept) | 0.02 | [-0.10, 0.14]
## SN_CO_Z | 0.13 | [ 0.03, 0.24]
## RADS_total | 0.28 | [ 0.17, 0.40]
## SN_CO_Z:RADS_total | -3.15e-03 | [-0.10, 0.10]

### S4b.2 Between-Network admn_sn

modelS4b.2 <- lmer(formula = negemo_day_prop ~
 admn_sn*RADS_total +
 (1 |id_participant),
 data = df_aim1_all_language,
 na.action=na.exclude)
summary(modelS4b.2)

## Linear mixed model fit by REML. t-tests use Satterthwaite's method [
## lmerModLmerTest]
## Formula: negemo_day_prop ~ admn_sn * RADS_total + (1 | id_participant)
## Data: df_aim1_all_language
##
## REML criterion at convergence: 7026.7
##
## Scaled residuals:
## Min 1Q Median 3Q Max
## -2.2884 -0.5606 -0.0822 0.3719 10.1257
##
## Random effects:
## Groups Name Variance Std.Dev.
## id_participant (Intercept) 0.2626 0.5124
## Residual 2.1213 1.4565
## Number of obs: 1938, groups: id_participant, 31
##
## Fixed effects:
## Estimate Std. Error df t value Pr(>|t|)
## (Intercept) 0.847479 0.487740 26.315913 1.738 0.09398 .
## admn_sn 0.970270 1.575120 25.253244 0.616 0.54341
## RADS_total 0.019102 0.006480 25.497601 2.948 0.00676 **
## admn_sn:RADS_total -0.009304 0.019728 24.898368 -0.472 0.64133
## ---
## Signif. codes: 0 '***' 0.001 '**' 0.01 '*' 0.05 '.' 0.1 ' ' 1
##
## Correlation of Fixed Effects:
## (Intr) admn_s RADS_t
## admn_sn -0.426
## RADS_total -0.965 0.408
## admn_:RADS_ 0.436 -0.977 -0.451

standardize_parameters(modelS4b.2)

## # Standardization method: refit
##
## Parameter | Std. Coef. | 95% CI
## -----------------------------------------------
## (Intercept) | 0.05 | [-0.08, 0.19]
## admn_sn | 0.05 | [-0.07, 0.17]
## RADS_total | 0.18 | [ 0.06, 0.30]
## admn_sn:RADS_total | -0.03 | [-0.14, 0.09]

### S4b.3 Between-Network sn_lcen

modelS4b.3 <- lmer(formula = negemo_day_prop ~
 sn_lcen*RADS_total +
 (1 |id_participant),
 data = df_aim1_all_language,
 na.action=na.exclude)
summary(modelS4b.3)

## Linear mixed model fit by REML. t-tests use Satterthwaite's method [
## lmerModLmerTest]
## Formula: negemo_day_prop ~ sn_lcen * RADS_total + (1 | id_participant)
## Data: df_aim1_all_language
##
## REML criterion at convergence: 7509.1
##
## Scaled residuals:
## Min 1Q Median 3Q Max
## -2.2973 -0.5792 -0.0836 0.3780 10.1139
##
## Random effects:
## Groups Name Variance Std.Dev.
## id_participant (Intercept) 0.2975 0.5454
## Residual 2.1234 1.4572
## Number of obs: 2070, groups: id_participant, 34
##
## Fixed effects:
## Estimate Std. Error df t value Pr(>|t|)
## (Intercept) 0.624350 0.846466 29.678246 0.738 0.4666
## sn_lcen 0.345136 1.611233 28.391069 0.214 0.8319
## RADS_total 0.022044 0.010946 29.875920 2.014 0.0531 .
## sn_lcen:RADS_total -0.004438 0.021443 28.314153 -0.207 0.8375
## ---
## Signif. codes: 0 '***' 0.001 '**' 0.01 '*' 0.05 '.' 0.1 ' ' 1
##
## Correlation of Fixed Effects:
## (Intr) sn_lcn RADS_t
## sn_lcen -0.874
## RADS_total -0.973 0.855
## sn_lc:RADS_ 0.830 -0.962 -0.864

standardize_parameters(modelS4b.3)

## # Standardization method: refit
##
## Parameter | Std. Coef. | 95% CI
## -----------------------------------------------
## (Intercept) | 0.03 | [-0.11, 0.17]
## sn_lcen | 2.14e-03 | [-0.12, 0.12]
## RADS_total | 0.24 | [ 0.11, 0.37]
## sn_lcen:RADS_total | -0.01 | [-0.12, 0.10]

### S4b.4 Between-Network sn_rcen

modelS4b.4 <- lmer(formula = negemo_day_prop ~
 sn_rcen*RADS_total +
 (1 |id_participant),
 data = df_aim1_all_language,
 na.action=na.exclude)
summary(modelS4b.4)

## Linear mixed model fit by REML. t-tests use Satterthwaite's method [
## lmerModLmerTest]
## Formula: negemo_day_prop ~ sn_rcen * RADS_total + (1 | id_participant)
## Data: df_aim1_all_language
##
## REML criterion at convergence: 7508.1
##
## Scaled residuals:
## Min 1Q Median 3Q Max
## -2.2950 -0.5801 -0.0832 0.3750 10.1099
##
## Random effects:
## Groups Name Variance Std.Dev.
## id_participant (Intercept) 0.293 0.5413
## Residual 2.123 1.4571
## Number of obs: 2070, groups: id_participant, 34
##
## Fixed effects:
## Estimate Std. Error df t value Pr(>|t|)
## (Intercept) 0.941061 0.939815 29.192496 1.001 0.325
## sn_rcen -0.428411 2.296036 28.722148 -0.187 0.853
## RADS_total 0.019369 0.012123 28.894497 1.598 0.121
## sn_rcen:RADS_total 0.001677 0.029422 28.232473 0.057 0.955
##
## Correlation of Fixed Effects:
## (Intr) sn_rcn RADS_t
## sn_rcen -0.901
## RADS_total -0.981 0.882
## sn_rc:RADS_ 0.888 -0.984 -0.896

standardize_parameters(modelS4b.4)

## # Standardization method: refit
##
## Parameter | Std. Coef. | 95% CI
## -----------------------------------------------
## (Intercept) | 0.02 | [-0.11, 0.16]
## sn_rcen | -0.04 | [-0.16, 0.07]
## RADS_total | 0.24 | [ 0.11, 0.37]
## sn_rcen:RADS_total | 4.64e-03 | [-0.15, 0.16]

## 4c. MDD - Future focus words

### 4c.1 Within-Network aDMN

model4c.1 <- lmer(formula = focusfuture_day_prop ~
 aDMN_Z*Group +
 (1 |id_participant),
 data = df_aim1_all_language,
 na.action=na.exclude)
summary(model4c.1)

## Linear mixed model fit by REML. t-tests use Satterthwaite's method [
## lmerModLmerTest]
## Formula: focusfuture_day_prop ~ aDMN_Z * Group + (1 | id_participant)
## Data: df_aim1_all_language
##
## REML criterion at convergence: 6303.5
##
## Scaled residuals:
## Min 1Q Median 3Q Max
## -2.0052 -0.6070 -0.0881 0.4232 8.1157
##
## Random effects:
## Groups Name Variance Std.Dev.
## id_participant (Intercept) 0.125 0.3535
## Residual 1.196 1.0938
## Number of obs: 2070, groups: id_participant, 34
##
## Fixed effects:
## Estimate Std. Error df t value Pr(>|t|)
## (Intercept) 1.5712 0.5093 28.7066 3.085 0.00447 **
## aDMN_Z 0.1090 0.3151 28.9901 0.346 0.73190
## GroupMDD -1.3030 0.6777 31.0269 -1.923 0.06373 .
## aDMN_Z:GroupMDD 0.6572 0.4321 31.8152 1.521 0.13815
## ---
## Signif. codes: 0 '***' 0.001 '**' 0.01 '*' 0.05 '.' 0.1 ' ' 1
##
## Correlation of Fixed Effects:
## (Intr) aDMN_Z GrpMDD
## aDMN_Z -0.965
## GroupMDD -0.752 0.725
## aDMN_Z:GMDD 0.703 -0.729 -0.972

standardize_parameters(model4c.1)

## # Standardization method: refit
##
## Parameter | Std. Coef. | 95% CI
## --------------------------------------------
## (Intercept) | 0.29 | [ 0.06, 0.52]
## aDMN_Z | 0.03 | [-0.12, 0.17]
## GroupMDD | -0.27 | [-0.54, 0.00]
## aDMN_Z:GroupMDD | 0.15 | [-0.04, 0.35]

### 4c.2 Between-Network admn_lcen

model4c.2 <- lmer(formula = focusfuture_day_prop ~
 admn_lcen*Group +
 (1 |id_participant),
 data = df_aim1_all_language,
 na.action=na.exclude)
summary(model4c.2)

## Linear mixed model fit by REML. t-tests use Satterthwaite's method [
## lmerModLmerTest]
## Formula: focusfuture_day_prop ~ admn_lcen * Group + (1 | id_participant)
## Data: df_aim1_all_language
##
## REML criterion at convergence: 6219.6
##
## Scaled residuals:
## Min 1Q Median 3Q Max
## -2.0416 -0.6089 -0.0905 0.4268 8.1951
##
## Random effects:
## Groups Name Variance Std.Dev.
## id_participant (Intercept) 0.1396 0.3736
## Residual 1.1757 1.0843
## Number of obs: 2054, groups: id_participant, 32
##
## Fixed effects:
## Estimate Std. Error df t value Pr(>|t|)
## (Intercept) 1.9710 0.2710 39.3810 7.272 8.59e-09 ***
## admn_lcen -0.5816 0.6967 36.3565 -0.835 0.409
## GroupMDD -0.4735 0.3029 34.4654 -1.564 0.127
## admn_lcen:GroupMDD 0.3234 0.7571 32.8858 0.427 0.672
## ---
## Signif. codes: 0 '***' 0.001 '**' 0.01 '*' 0.05 '.' 0.1 ' ' 1
##
## Correlation of Fixed Effects:
## (Intr) admn_l GrpMDD
## admn_lcen -0.838
## GroupMDD -0.895 0.750
## admn_l:GMDD 0.771 -0.920 -0.823

standardize_parameters(model4c.2)

## # Standardization method: refit
##
## Parameter | Std. Coef. | 95% CI
## -----------------------------------------------
## (Intercept) | 0.29 | [ 0.02, 0.56]
## admn_lcen | -0.14 | [-0.46, 0.19]
## GroupMDD | -0.30 | [-0.61, 0.01]
## admn_lcen:GroupMDD | 0.08 | [-0.27, 0.43]

### 4c.3 Between-Network admn_rcen

model4c.3 <- lmer(formula = focusfuture_day_prop ~
 admn_rcen*Group +
 (1 |id_participant),
 data = df_aim1_all_language,
 na.action=na.exclude)
summary(model4c.3)

## Linear mixed model fit by REML. t-tests use Satterthwaite's method [
## lmerModLmerTest]
## Formula: focusfuture_day_prop ~ admn_rcen * Group + (1 | id_participant)
## Data: df_aim1_all_language
##
## REML criterion at convergence: 5776.2
##
## Scaled residuals:
## Min 1Q Median 3Q Max
## -2.1492 -0.6146 -0.0870 0.4179 8.3989
##
## Random effects:
## Groups Name Variance Std.Dev.
## id_participant (Intercept) 0.1342 0.3663
## Residual 1.1202 1.0584
## Number of obs: 1938, groups: id_participant, 31
##
## Fixed effects:
## Estimate Std. Error df t value Pr(>|t|)
## (Intercept) 2.1877 0.3143 22.1889 6.961 5.23e-07 ***
## admn_rcen -0.7978 0.5200 19.9346 -1.534 0.141
## GroupMDD -0.5273 0.3664 20.9459 -1.439 0.165
## admn_rcen:GroupMDD 0.3833 0.5884 19.4584 0.652 0.522
## ---
## Signif. codes: 0 '***' 0.001 '**' 0.01 '*' 0.05 '.' 0.1 ' ' 1
##
## Correlation of Fixed Effects:
## (Intr) admn_r GrpMDD
## admn_rcen -0.866
## GroupMDD -0.858 0.743
## admn_r:GMDD 0.766 -0.884 -0.871

standardize_parameters(model4c.3)

## # Standardization method: refit
##
## Parameter | Std. Coef. | 95% CI
## -----------------------------------------------
## (Intercept) | 0.28 | [-0.01, 0.57]
## admn_rcen | -0.24 | [-0.54, 0.07]
## GroupMDD | -0.26 | [-0.59, 0.07]
## admn_rcen:GroupMDD | 0.11 | [-0.23, 0.46]

### 4c.4 Between-Network admn_sn

model4c.4 <- lmer(formula = focusfuture_day_prop ~
 admn_sn*Group +
 (1 |id_participant),
 data = df_aim1_all_language,
 na.action=na.exclude)
summary(model4c.4)

## Linear mixed model fit by REML. t-tests use Satterthwaite's method [
## lmerModLmerTest]
## Formula: focusfuture_day_prop ~ admn_sn * Group + (1 | id_participant)
## Data: df_aim1_all_language
##
## REML criterion at convergence: 5779.6
##
## Scaled residuals:
## Min 1Q Median 3Q Max
## -2.1200 -0.6138 -0.0896 0.4110 8.3933
##
## Random effects:
## Groups Name Variance Std.Dev.
## id_participant (Intercept) 0.1521 0.390
## Residual 1.1205 1.059
## Number of obs: 1938, groups: id_participant, 31
##
## Fixed effects:
## Estimate Std. Error df t value Pr(>|t|)
## (Intercept) 1.8332 0.1739 21.3935 10.543 6.22e-10 ***
## admn_sn -0.5001 0.4508 18.7793 -1.109 0.2813
## GroupMDD -0.4155 0.2103 20.5134 -1.976 0.0618 .
## admn_sn:GroupMDD 0.4685 0.5450 18.7553 0.860 0.4009
## ---
## Signif. codes: 0 '***' 0.001 '**' 0.01 '*' 0.05 '.' 0.1 ' ' 1
##
## Correlation of Fixed Effects:
## (Intr) admn_s GrpMDD
## admn_sn -0.312
## GroupMDD -0.827 0.258
## admn_s:GMDD 0.258 -0.827 -0.417

standardize_parameters(model4c.4)

## # Standardization method: refit
##
## Parameter | Std. Coef. | 95% CI
## ---------------------------------------------
## (Intercept) | 0.31 | [ 0.01, 0.61]
## admn_sn | -0.13 | [-0.36, 0.10]
## GroupMDD | -0.29 | [-0.63, 0.05]
## admn_sn:GroupMDD | 0.12 | [-0.15, 0.40]

## S4c. Dep Sx - Future focus words

### S4c.1 Within-Network aDMN

modelS4c.1 <- lmer(formula = focusfuture_day_prop ~
 aDMN_Z*RADS_total +
 (1 |id_participant),
 data = df_aim1_all_language,
 na.action=na.exclude)
summary(modelS4c.1)

## Linear mixed model fit by REML. t-tests use Satterthwaite's method [
## lmerModLmerTest]
## Formula: focusfuture_day_prop ~ aDMN_Z * RADS_total + (1 | id_participant)
## Data: df_aim1_all_language
##
## REML criterion at convergence: 6321.6
##
## Scaled residuals:
## Min 1Q Median 3Q Max
## -1.9441 -0.6120 -0.0921 0.4205 8.1115
##
## Random effects:
## Groups Name Variance Std.Dev.
## id_participant (Intercept) 0.1419 0.3767
## Residual 1.1965 1.0939
## Number of obs: 2070, groups: id_participant, 34
##
## Fixed effects:
## Estimate Std. Error df t value Pr(>|t|)
## (Intercept) 1.698969 1.189032 26.184255 1.429 0.165
## aDMN_Z 0.164538 0.728789 27.266314 0.226 0.823
## RADS_total -0.012407 0.016647 26.051557 -0.745 0.463
## aDMN_Z:RADS_total 0.004233 0.010509 27.689332 0.403 0.690
##
## Correlation of Fixed Effects:
## (Intr) aDMN_Z RADS_t
## aDMN_Z -0.970
## RADS_total -0.954 0.938
## aDMN_Z:RADS 0.910 -0.949 -0.973

standardize_parameters(modelS4c.1)

## # Standardization method: refit
##
## Parameter | Std. Coef. | 95% CI
## ----------------------------------------------
## (Intercept) | 0.08 | [-0.05, 0.21]
## aDMN_Z | 0.11 | [ 0.00, 0.22]
## RADS_total | -0.10 | [-0.22, 0.03]
## aDMN_Z:RADS_total | 0.02 | [-0.07, 0.11]

### S4c.2 Between-Network admn_lcen

modelS4c.2 <- lmer(formula = focusfuture_day_prop ~
 admn_lcen*RADS_total +
 (1 |id_participant),
 data = df_aim1_all_language,
 na.action=na.exclude)
summary(modelS4c.2)

## Linear mixed model fit by REML. t-tests use Satterthwaite's method [
## lmerModLmerTest]
## Formula: focusfuture_day_prop ~ admn_lcen * RADS_total + (1 | id_participant)
## Data: df_aim1_all_language
##
## REML criterion at convergence: 6235.9
##
## Scaled residuals:
## Min 1Q Median 3Q Max
## -1.9839 -0.6114 -0.0887 0.4216 8.2083
##
## Random effects:
## Groups Name Variance Std.Dev.
## id_participant (Intercept) 0.1504 0.3878
## Residual 1.1754 1.0842
## Number of obs: 2054, groups: id_participant, 32
##
## Fixed effects:
## Estimate Std. Error df t value Pr(>|t|)
## (Intercept) 1.728819 0.560400 29.351438 3.085 0.00441 **
## admn_lcen 0.812237 1.479013 29.560167 0.549 0.58701
## RADS_total -0.002311 0.007210 27.360675 -0.321 0.75096
## admn_lcen:RADS_total -0.012683 0.017599 28.418172 -0.721 0.47701
## ---
## Signif. codes: 0 '***' 0.001 '**' 0.01 '*' 0.05 '.' 0.1 ' ' 1
##
## Correlation of Fixed Effects:
## (Intr) admn_l RADS_t
## admn_lcen -0.843
## RADS_total -0.973 0.794
## admn_:RADS_ 0.847 -0.981 -0.832

standardize_parameters(modelS4c.2)

## # Standardization method: refit
##
## Parameter | Std. Coef. | 95% CI
## -------------------------------------------------
## (Intercept) | 0.09 | [-0.06, 0.23]
## admn_lcen | -0.03 | [-0.18, 0.12]
## RADS_total | -0.12 | [-0.26, 0.01]
## admn_lcen:RADS_total | -0.06 | [-0.21, 0.10]

### S4c.3 Between-Network admn_rcen

modelS4c.3 <- lmer(formula = focusfuture_day_prop ~
 admn_rcen*RADS_total +
 (1 |id_participant),
 data = df_aim1_all_language,
 na.action=na.exclude)
summary(modelS4c.3)

## Linear mixed model fit by REML. t-tests use Satterthwaite's method [
## lmerModLmerTest]
## Formula: focusfuture_day_prop ~ admn_rcen * RADS_total + (1 | id_participant)
## Data: df_aim1_all_language
##
## REML criterion at convergence: 5792.4
##
## Scaled residuals:
## Min 1Q Median 3Q Max
## -2.1288 -0.6200 -0.0896 0.4140 8.4159
##
## Random effects:
## Groups Name Variance Std.Dev.
## id_participant (Intercept) 0.1471 0.3836
## Residual 1.1197 1.0581
## Number of obs: 1938, groups: id_participant, 31
##
## Fixed effects:
## Estimate Std. Error df t value Pr(>|t|)
## (Intercept) 2.741091 0.658744 21.872580 4.161 0.000411 ***
## admn_rcen -1.539437 1.070152 21.217150 -1.439 0.164875
## RADS_total -0.012000 0.008002 20.803974 -1.500 0.148728
## admn_rcen:RADS_total 0.012234 0.012000 20.642417 1.019 0.319781
## ---
## Signif. codes: 0 '***' 0.001 '**' 0.01 '*' 0.05 '.' 0.1 ' ' 1
##
## Correlation of Fixed Effects:
## (Intr) admn_r RADS_t
## admn_rcen -0.862
## RADS_total -0.962 0.780
## admn_:RADS_ 0.861 -0.970 -0.837

standardize_parameters(modelS4c.3)

## # Standardization method: refit
##
## Parameter | Std. Coef. | 95% CI
## --------------------------------------------------
## (Intercept) | 0.06 | [-0.09, 0.20]
## admn_rcen | -0.18 | [-0.34, -0.01]
## RADS_total | -0.06 | [-0.19, 0.06]
## admn_rcen:RADS_total | 0.06 | [-0.05, 0.17]

### S4c.4 Between-Network admn_sn

modelS4c.4 <- lmer(formula = focusfuture_day_prop ~
 admn_sn*RADS_total +
 (1 |id_participant),
 data = df_aim1_all_language,
 na.action=na.exclude)
summary(modelS4c.4)

## Linear mixed model fit by REML. t-tests use Satterthwaite's method [
## lmerModLmerTest]
## Formula: focusfuture_day_prop ~ admn_sn * RADS_total + (1 | id_participant)
## Data: df_aim1_all_language
##
## REML criterion at convergence: 5795.8
##
## Scaled residuals:
## Min 1Q Median 3Q Max
## -2.0805 -0.6159 -0.0883 0.4107 8.4099
##
## Random effects:
## Groups Name Variance Std.Dev.
## id_participant (Intercept) 0.1708 0.4133
## Residual 1.1200 1.0583
## Number of obs: 1938, groups: id_participant, 31
##
## Fixed effects:
## Estimate Std. Error df t value Pr(>|t|)
## (Intercept) 2.060462 0.386364 21.216985 5.333 2.65e-05 ***
## admn_sn -0.397371 1.250132 20.251022 -0.318 0.754
## RADS_total -0.006990 0.005140 20.593878 -1.360 0.189
## admn_sn:RADS_total 0.002563 0.015667 19.997971 0.164 0.872
## ---
## Signif. codes: 0 '***' 0.001 '**' 0.01 '*' 0.05 '.' 0.1 ' ' 1
##
## Correlation of Fixed Effects:
## (Intr) admn_s RADS_t
## admn_sn -0.427
## RADS_total -0.965 0.409
## admn_:RADS_ 0.437 -0.977 -0.453

standardize_parameters(modelS4c.4)

## # Standardization method: refit
##
## Parameter | Std. Coef. | 95% CI
## -----------------------------------------------
## (Intercept) | 0.09 | [-0.06, 0.24]
## admn_sn | -0.05 | [-0.19, 0.08]
## RADS_total | -0.09 | [-0.23, 0.04]
## admn_sn:RADS_total | 0.01 | [-0.12, 0.14]
