# Supplementary material for: Neural correlates of depression-related smartphone language use in adolescents
Source: NPP Digit Psychiatry Neurosci. 2024 Jul 9;2:11. doi: 10.1038/s44277-024-00009-6 (PMC12592214; doi:10.1038/s44277-024-00009-6)
Supplement: Supplementary file 3 — Supplement 3 [file 44277_2024_9_MOESM3_ESM.docx]

Supplement 3: Sensitivity Analysis Results

Neural Correlates of Depression-Related Smartphone Language Use in Adolescents

Table of Contents

[1. AIM 1: What are the linguistic features of smartphone use that are associated with depression? 4](#_Toc164094100)

[S1a.1 Dep Sx - First-person pronouns 4](#_Toc164094101)

[S1a.2 Age 5](#_Toc164094102)

[S1a.3 Sex 6](#_Toc164094103)

[S1a.4 Gender 7](#_Toc164094104)

[S1a.5 COVID 8](#_Toc164094105)

[S1a.6 Number of Messages 10](#_Toc164094106)

[S1a.7 Days between V2 and EARS start 11](#_Toc164094107)

[S1a.8 Days since COVID stay-at-home order 12](#_Toc164094108)

[S1a.9 Medication Status 13](#_Toc164094109)

[1c.1 MDD - Negative Emotion Words 15](#_Toc164094110)

[1c.2 Age 16](#_Toc164094111)

[1c.3 Sex 17](#_Toc164094112)

[1c.4 Gender 18](#_Toc164094113)

[1c.5 COVID 19](#_Toc164094114)

[1c.6 Number of Messages 21](#_Toc164094115)

[1c.7 Days between V2 and EARS start 22](#_Toc164094116)

[1c.8 Days since COVID stay-at-home order 23](#_Toc164094117)

[1c.9 Medication Status 24](#_Toc164094118)

[S1c.1 Dep Sx - Negative Emotion Words 26](#_Toc164094119)

[S1c.2 Age 27](#_Toc164094120)

[S1c.3 Sex 28](#_Toc164094121)

[S1c.4 Gender 29](#_Toc164094122)

[S1c.5 COVID 30](#_Toc164094123)

[S1c.6 Number of Messages 32](#_Toc164094124)

[S1c.7 Days between fMRI and EARS start 33](#_Toc164094125)

[S1c.8 Days since COVID stay-at-home order 34](#_Toc164094126)

[S1c.9 Medication Status 35](#_Toc164094127)

[1f.1 MDD - Future Focus Words 37](#_Toc164094128)

[1f.2 Age 38](#_Toc164094129)

[1f.3 Sex 39](#_Toc164094130)

[1f.4 Gender 40](#_Toc164094131)

[1f.5 COVID 41](#_Toc164094132)

[1f.6 Number of Messages 43](#_Toc164094133)

[1f.7 Days between V2 and EARS start 44](#_Toc164094134)

[1f.8 Days since COVID stay-at-home order 45](#_Toc164094135)

[1f.9 Medication Status 46](#_Toc164094136)

[S1f.1 Dep Sx - Future Focus Words 48](#_Toc164094137)

[S1f.2 Age 49](#_Toc164094138)

[S1f.3 Sex 50](#_Toc164094139)

[S1f.4 Gender 51](#_Toc164094140)

[S1f.5 COVID 53](#_Toc164094141)

[S1f.6 Number of Messages 54](#_Toc164094142)

[S1f.7 Days between V2 and EARS start 55](#_Toc164094143)

[S1f.8 Days since COVID stay-at-home order 56](#_Toc164094144)

[S1f.9 Medication Status 58](#_Toc164094145)

[2. AIM 2 - Which intrinsic connectivity networks are associated with depression-related linguistic features of smartphone use? 59](#_Toc164094146)

[S2a. First-person pronouns (controlling for depressive symptoms) 59](#_Toc164094147)

[S2a.1a Within-Network aDMN 59](#_Toc164094148)

[S2a.1b Age 60](#_Toc164094149)

[S2a.1c Sex 61](#_Toc164094150)

[S2a.1d Motion 63](#_Toc164094151)

[S2a.1e Days between V2 and EARS start 64](#_Toc164094152)

[S2a.2a Within-Network leftCEN_Z 65](#_Toc164094153)

[S2a.2b Age 66](#_Toc164094154)

[S2a.2c Sex 67](#_Toc164094155)

[S2a.2d Motion 68](#_Toc164094156)

[S2a.2e Days between V2 and EARS start 69](#_Toc164094157)

[2b. Negative Emotion Words (controlling for Group) 70](#_Toc164094158)

[2b.4a Within-Network SN_CO_Z 70](#_Toc164094159)

[2b.4b Age 71](#_Toc164094160)

[2b.4c Sex 73](#_Toc164094161)

[2b.4d Motion 74](#_Toc164094162)

[2b.4e Days between V2 and EARS start 75](#_Toc164094163)

[S2b. Negative Emotion Words (controlling for depressive symptoms) 76](#_Toc164094164)

[S2b.4a Within-Network SN_CO_Z 76](#_Toc164094165)

[S2b.4b Age 77](#_Toc164094166)

[S2b.4c Sex 78](#_Toc164094167)

[S2b.4d Motion 80](#_Toc164094168)

[S2b.4e Days between V2 and EARS start 81](#_Toc164094169)

[2c. Future focus words (controlling for Group) 82](#_Toc164094170)

[2c.1a Within-Network aDMN 82](#_Toc164094171)

[2c.1b Age 83](#_Toc164094172)

[2c.1c Sex 84](#_Toc164094173)

[2c.1d Motion 85](#_Toc164094174)

[2c.1e Days between V2 and EARS start 86](#_Toc164094175)

[3. AIM 3: Mediation: Does intrinsic network connectivity mediate the association between depression and depression-related linguistic features of smartphone use? 88](#_Toc164094176)

[3a.1 Dep Sx - leftCEN - First-person Pronouns 88](#_Toc164094177)

[3a.2 Motion 93](#_Toc164094178)

[4. AIM 4 - Does intrinsic connectivity moderate the association between depression and depression-related linguistic features of smartphone use? 98](#_Toc164094179)

[4b. MDD - Negative Emotion Words 98](#_Toc164094180)

[4b.3a Between-Network sn_lcen 98](#_Toc164094181)

[4b.3b Age 99](#_Toc164094182)

[4b.3c Sex 100](#_Toc164094183)

[4b.3d Motion 101](#_Toc164094184)

[4b.4a Between-Network sn_rcen 103](#_Toc164094185)

[4b.4b Age 104](#_Toc164094186)

[4b.4c Sex 105](#_Toc164094187)

[4b.4d Motion 106](#_Toc164094188)

# 1. AIM 1: What are the linguistic features of smartphone use that are associated with depression?

## S1a.1 Dep Sx - First-person pronouns

#First-person pronouns (daily proportion of total words)
 model.S1a.1 <- lmer(formula = i_day_prop ~
 RADS_total
 + (1 |id_participant),
 data = df_aim1_all_language,
 na.action=na.exclude)
summary(model.S1a.1)

## Linear mixed model fit by REML. t-tests use Satterthwaite's method [
## lmerModLmerTest]
## Formula: i_day_prop ~ RADS_total + (1 | id_participant)
## Data: df_aim1_all_language
##
## REML criterion at convergence: 11974.2
##
## Scaled residuals:
## Min 1Q Median 3Q Max
## -3.1166 -0.5743 -0.0162 0.5058 7.4826
##
## Random effects:
## Groups Name Variance Std.Dev.
## id_participant (Intercept) 1.889 1.374
## Residual 11.538 3.397
## Number of obs: 2251, groups: id_participant, 40
##
## Fixed effects:
## Estimate Std. Error df t value Pr(>|t|)
## (Intercept) 5.50537 0.93401 32.77152 5.894 1.35e-06 ***
## RADS_total 0.03680 0.01256 32.15868 2.929 0.00621 **
## ---
## Signif. codes: 0 '***' 0.001 '**' 0.01 '*' 0.05 '.' 0.1 ' ' 1
##
## Correlation of Fixed Effects:
## (Intr)
## RADS_total -0.964

standardize_parameters(model.S1a.1)

## # Standardization method: refit
##
## Parameter | Std. Coef. | 95% CI
## ----------------------------------------
## (Intercept) | 0.01 | [-0.12, 0.14]
## RADS_total | 0.19 | [ 0.06, 0.31]

### S1a.2 Age

#First-person pronouns (daily proportion of total words)
 model.S1a.2 <- lmer(formula = i_day_prop ~
 RADS_total
 + Age.at.V2
 + Sex
 + (1 |id_participant),
 data = df_aim1_all_language,
 na.action=na.exclude)
summary(model.S1a.2)

## Linear mixed model fit by REML. t-tests use Satterthwaite's method [
## lmerModLmerTest]
## Formula: i_day_prop ~ RADS_total + Age.at.V2 + Sex + (1 | id_participant)
## Data: df_aim1_all_language
##
## REML criterion at convergence: 11974
##
## Scaled residuals:
## Min 1Q Median 3Q Max
## -3.1275 -0.5758 -0.0125 0.5057 7.4829
##
## Random effects:
## Groups Name Variance Std.Dev.
## id_participant (Intercept) 2.003 1.415
## Residual 11.536 3.397
## Number of obs: 2251, groups: id_participant, 40
##
## Fixed effects:
## Estimate Std. Error df t value Pr(>|t|)
## (Intercept) 5.75565 3.91029 32.89537 1.472 0.1505
## RADS_total 0.03322 0.01354 32.48517 2.453 0.0197 *
## Age.at.V2 -0.04505 0.22250 32.40717 -0.202 0.8408
## Sex 0.45454 0.54977 32.79945 0.827 0.4143
## ---
## Signif. codes: 0 '***' 0.001 '**' 0.01 '*' 0.05 '.' 0.1 ' ' 1
##
## Correlation of Fixed Effects:
## (Intr) RADS_t Ag..V2
## RADS_total -0.259
## Age.at.V2 -0.958 0.083
## Sex -0.213 -0.292 0.064

standardize_parameters(model.S1a.2)

## # Standardization method: refit
##
## Parameter | Std. Coef. | 95% CI
## ----------------------------------------
## (Intercept) | 3.64e-03 | [-0.14, 0.14]
## RADS_total | 0.17 | [ 0.03, 0.30]
## Age.at.V2 | -0.01 | [-0.16, 0.13]
## Sex | 0.06 | [-0.08, 0.21]

#Model comparison with LRT
anova(model.S1a.1, model.S1a.2)

## refitting model(s) with ML (instead of REML)

## Data: df_aim1_all_language
## Models:
## model.S1a.1: i_day_prop ~ RADS_total + (1 | id_participant)
## model.S1a.2: i_day_prop ~ RADS_total + Age.at.V2 + Sex + (1 | id_participant)
## npar AIC BIC logLik deviance Chisq Df Pr(>Chisq)
## model.S1a.1 4 11974 11997 -5983.1 11966
## model.S1a.2 6 11978 12012 -5982.7 11966 0.793 2 0.6727

### S1a.3 Sex

#First-person pronouns (daily proportion of total words)
 model.S1a.3 <- lmer(formula = i_day_prop ~
 RADS_total
 + Sex
 + (1 |id_participant),
 data = df_aim1_all_language,
 na.action=na.exclude)
summary(model.S1a.3)

## Linear mixed model fit by REML. t-tests use Satterthwaite's method [
## lmerModLmerTest]
## Formula: i_day_prop ~ RADS_total + Sex + (1 | id_participant)
## Data: df_aim1_all_language
##
## REML criterion at convergence: 11972.9
##
## Scaled residuals:
## Min 1Q Median 3Q Max
## -3.1263 -0.5764 -0.0147 0.5065 7.4847
##
## Random effects:
## Groups Name Variance Std.Dev.
## id_participant (Intercept) 1.942 1.394
## Residual 11.536 3.396
## Number of obs: 2251, groups: id_participant, 40
##
## Fixed effects:
## Estimate Std. Error df t value Pr(>|t|)
## (Intercept) 5.00581 1.11268 30.45691 4.499 9.28e-05 ***
## RADS_total 0.03344 0.01332 33.72019 2.510 0.017 *
## Sex 0.45783 0.54163 33.59229 0.845 0.404
## ---
## Signif. codes: 0 '***' 0.001 '**' 0.01 '*' 0.05 '.' 0.1 ' ' 1
##
## Correlation of Fixed Effects:
## (Intr) RADS_t
## RADS_total -0.623
## Sex -0.528 -0.300

standardize_parameters(model.S1a.3)

## # Standardization method: refit
##
## Parameter | Std. Coef. | 95% CI
## ----------------------------------------
## (Intercept) | 1.40e-03 | [-0.13, 0.14]
## RADS_total | 0.17 | [ 0.04, 0.30]
## Sex | 0.06 | [-0.08, 0.20]

#Model comparison with LRT
anova(model.S1a.1, model.S1a.3)

## refitting model(s) with ML (instead of REML)

## Data: df_aim1_all_language
## Models:
## model.S1a.1: i_day_prop ~ RADS_total + (1 | id_participant)
## model.S1a.3: i_day_prop ~ RADS_total + Sex + (1 | id_participant)
## npar AIC BIC logLik deviance Chisq Df Pr(>Chisq)
## model.S1a.1 4 11974 11997 -5983.1 11966
## model.S1a.3 5 11976 12004 -5982.8 11966 0.7324 1 0.3921

### S1a.4 Gender

#First-person pronouns (daily proportion of total words)
 model.S1a.4 <- lmer(formula = i_day_prop ~
 RADS_total
 + Gender
 + (1 |id_participant),
 data = df_aim1_all_language,
 na.action=na.exclude)
summary(model.S1a.4)

## Linear mixed model fit by REML. t-tests use Satterthwaite's method [
## lmerModLmerTest]
## Formula: i_day_prop ~ RADS_total + Gender + (1 | id_participant)
## Data: df_aim1_all_language
##
## REML criterion at convergence: 11974.1
##
## Scaled residuals:
## Min 1Q Median 3Q Max
## -3.1209 -0.5736 -0.0166 0.5057 7.4820
##
## Random effects:
## Groups Name Variance Std.Dev.
## id_participant (Intercept) 1.968 1.403
## Residual 11.537 3.397
## Number of obs: 2251, groups: id_participant, 40
##
## Fixed effects:
## Estimate Std. Error df t value Pr(>|t|)
## (Intercept) 5.45467 0.99884 30.43271 5.461 6.06e-06 ***
## RADS_total 0.03591 0.01414 33.10831 2.540 0.016 *
## Gender 0.06251 0.41374 30.54927 0.151 0.881
## ---
## Signif. codes: 0 '***' 0.001 '**' 0.01 '*' 0.05 '.' 0.1 ' ' 1
##
## Correlation of Fixed Effects:
## (Intr) RADS_t
## RADS_total -0.697
## Gender -0.308 -0.427

standardize_parameters(model.S1a.4)

## # Standardization method: refit
##
## Parameter | Std. Coef. | 95% CI
## ----------------------------------------
## (Intercept) | 0.01 | [-0.12, 0.15]
## RADS_total | 0.18 | [ 0.04, 0.32]
## Gender | 0.01 | [-0.16, 0.19]

#Model comparison with LRT
anova(model.S1a.1, model.S1a.4)

## refitting model(s) with ML (instead of REML)

## Data: df_aim1_all_language
## Models:
## model.S1a.1: i_day_prop ~ RADS_total + (1 | id_participant)
## model.S1a.4: i_day_prop ~ RADS_total + Gender + (1 | id_participant)
## npar AIC BIC logLik deviance Chisq Df Pr(>Chisq)
## model.S1a.1 4 11974 11997 -5983.1 11966
## model.S1a.4 5 11976 12005 -5983.1 11966 0.0144 1 0.9046

### S1a.5 COVID

#First-person pronouns (daily proportion of total words)
 model.S1a.5 <- lmer(formula = i_day_prop ~
 RADS_total
 + Collected_During_Covid
 + (1 |id_participant),
 data = df_aim1_all_language,
 na.action=na.exclude)
summary(model.S1a.5)

## Linear mixed model fit by REML. t-tests use Satterthwaite's method [
## lmerModLmerTest]
## Formula:
## i_day_prop ~ RADS_total + Collected_During_Covid + (1 | id_participant)
## Data: df_aim1_all_language
##
## REML criterion at convergence: 11973
##
## Scaled residuals:
## Min 1Q Median 3Q Max
## -3.1100 -0.5745 -0.0154 0.5050 7.4825
##
## Random effects:
## Groups Name Variance Std.Dev.
## id_participant (Intercept) 1.948 1.396
## Residual 11.537 3.397
## Number of obs: 2251, groups: id_participant, 40
##
## Fixed effects:
## Estimate Std. Error df t value Pr(>|t|)
## (Intercept) 5.37333 0.97153 32.37386 5.531 4.09e-06 ***
## RADS_total 0.04010 0.01391 31.96257 2.882 0.00701 **
## Collected_During_Covid -0.35000 0.59943 32.57844 -0.584 0.56332
## ---
## Signif. codes: 0 '***' 0.001 '**' 0.01 '*' 0.05 '.' 0.1 ' ' 1
##
## Correlation of Fixed Effects:
## (Intr) RADS_t
## RADS_total -0.950
## Cllctd_Dr_C 0.227 -0.404

standardize_parameters(model.S1a.5)

## # Standardization method: refit
##
## Parameter | Std. Coef. | 95% CI
## ---------------------------------------------------
## (Intercept) | 0.01 | [-0.12, 0.15]
## RADS_total | 0.20 | [ 0.07, 0.34]
## Collected_During_Covid | -0.04 | [-0.19, 0.10]

#Model comparison with LRT
anova(model.S1a.1, model.S1a.5)

## refitting model(s) with ML (instead of REML)

## Data: df_aim1_all_language
## Models:
## model.S1a.1: i_day_prop ~ RADS_total + (1 | id_participant)
## model.S1a.5: i_day_prop ~ RADS_total + Collected_During_Covid + (1 | id_participant)
## npar AIC BIC logLik deviance Chisq Df Pr(>Chisq)
## model.S1a.1 4 11974 11997 -5983.1 11966
## model.S1a.5 5 11976 12004 -5983.0 11966 0.3545 1 0.5516

### S1a.6 Number of Messages

#First-person pronouns (daily proportion of total words)
 model.S1a.6 <- lmer(formula = i_day_prop ~
 RADS_total
 + n_msg_day
 + (1 |id_participant),
 data = df_aim1_all_language,
 na.action=na.exclude)
summary(model.S1a.6)

## Linear mixed model fit by REML. t-tests use Satterthwaite's method [
## lmerModLmerTest]
## Formula: i_day_prop ~ RADS_total + n_msg_day + (1 | id_participant)
## Data: df_aim1_all_language
##
## REML criterion at convergence: 11977.1
##
## Scaled residuals:
## Min 1Q Median 3Q Max
## -3.0773 -0.5644 -0.0309 0.5044 7.5059
##
## Random effects:
## Groups Name Variance Std.Dev.
## id_participant (Intercept) 1.765 1.328
## Residual 11.499 3.391
## Number of obs: 2251, groups: id_participant, 40
##
## Fixed effects:
## Estimate Std. Error df t value Pr(>|t|)
## (Intercept) 5.550e+00 9.078e-01 3.220e+01 6.114 7.62e-07 ***
## RADS_total 3.367e-02 1.224e-02 3.199e+01 2.750 0.00972 **
## n_msg_day 1.544e-03 4.738e-04 1.912e+03 3.259 0.00114 **
## ---
## Signif. codes: 0 '***' 0.001 '**' 0.01 '*' 0.05 '.' 0.1 ' ' 1
##
## Correlation of Fixed Effects:
## (Intr) RADS_t
## RADS_total -0.962
## n_msg_day 0.013 -0.077

standardize_parameters(model.S1a.6)

## # Standardization method: refit
##
## Parameter | Std. Coef. | 95% CI
## ----------------------------------------
## (Intercept) | 0.02 | [-0.11, 0.15]
## RADS_total | 0.17 | [ 0.05, 0.29]
## n_msg_day | 0.09 | [ 0.03, 0.14]

#Model comparison with LRT
anova(model.S1a.1, model.S1a.6)

## refitting model(s) with ML (instead of REML)

## Data: df_aim1_all_language
## Models:
## model.S1a.1: i_day_prop ~ RADS_total + (1 | id_participant)
## model.S1a.6: i_day_prop ~ RADS_total + n_msg_day + (1 | id_participant)
## npar AIC BIC logLik deviance Chisq Df Pr(>Chisq)
## model.S1a.1 4 11974 11997 -5983.1 11966
## model.S1a.6 5 11966 11994 -5977.8 11956 10.671 1 0.001088 **
## ---
## Signif. codes: 0 '***' 0.001 '**' 0.01 '*' 0.05 '.' 0.1 ' ' 1

### S1a.7 Days between V2 and EARS start

#First-person pronouns (daily proportion of total words)
 model.S1a.7 <- lmer(formula = i_day_prop ~
 RADS_total
 + days_since_V2
 + (1 |id_participant),
 data = df_aim1_all_language,
 na.action=na.exclude)
summary(model.S1a.7)

## Linear mixed model fit by REML. t-tests use Satterthwaite's method [
## lmerModLmerTest]
## Formula: i_day_prop ~ RADS_total + days_since_V2 + (1 | id_participant)
## Data: df_aim1_all_language
##
## REML criterion at convergence: 11985.7
##
## Scaled residuals:
## Min 1Q Median 3Q Max
## -3.1105 -0.5747 -0.0148 0.5043 7.4833
##
## Random effects:
## Groups Name Variance Std.Dev.
## id_participant (Intercept) 1.95 1.396
## Residual 11.54 3.397
## Number of obs: 2251, groups: id_participant, 40
##
## Fixed effects:
## Estimate Std. Error df t value Pr(>|t|)
## (Intercept) 5.293e+00 9.968e-01 3.261e+01 5.310 7.64e-06 ***
## RADS_total 3.681e-02 1.273e-02 3.172e+01 2.891 0.00688 **
## days_since_V2 6.628e-04 9.923e-04 3.165e+01 0.668 0.50897
## ---
## Signif. codes: 0 '***' 0.001 '**' 0.01 '*' 0.05 '.' 0.1 ' ' 1
##
## Correlation of Fixed Effects:
## (Intr) RADS_t
## RADS_total -0.915
## days_snc_V2 -0.314 -0.001

standardize_parameters(model.S1a.7)

## # Standardization method: refit
##
## Parameter | Std. Coef. | 95% CI
## ------------------------------------------
## (Intercept) | 0.01 | [-0.12, 0.15]
## RADS_total | 0.19 | [ 0.06, 0.31]
## days_since_V2 | 0.05 | [-0.09, 0.18]

#Model comparison with LRT
anova(model.S1a.1, model.S1a.7)

## refitting model(s) with ML (instead of REML)

## Data: df_aim1_all_language
## Models:
## model.S1a.1: i_day_prop ~ RADS_total + (1 | id_participant)
## model.S1a.7: i_day_prop ~ RADS_total + days_since_V2 + (1 | id_participant)
## npar AIC BIC logLik deviance Chisq Df Pr(>Chisq)
## model.S1a.1 4 11974 11997 -5983.1 11966
## model.S1a.7 5 11976 12004 -5982.9 11966 0.4582 1 0.4985

### S1a.8 Days since COVID stay-at-home order

#First-person pronouns (daily proportion of total words)
 model.S1a.8 <- lmer(formula = i_day_prop ~
 RADS_total
 + days_since_stay_at_home
 + (1 |id_participant),
 data = df_aim1_all_language,
 na.action=na.exclude)
summary(model.S1a.8)

## Linear mixed model fit by REML. t-tests use Satterthwaite's method [
## lmerModLmerTest]
## Formula:
## i_day_prop ~ RADS_total + days_since_stay_at_home + (1 | id_participant)
## Data: df_aim1_all_language
##
## REML criterion at convergence: 11985
##
## Scaled residuals:
## Min 1Q Median 3Q Max
## -3.1135 -0.5749 -0.0175 0.5044 7.4819
##
## Random effects:
## Groups Name Variance Std.Dev.
## id_participant (Intercept) 1.949 1.396
## Residual 11.538 3.397
## Number of obs: 2251, groups: id_participant, 40
##
## Fixed effects:
## Estimate Std. Error df t value Pr(>|t|)
## (Intercept) 5.4542933 0.9573387 31.8765372 5.697 2.65e-06 ***
## RADS_total 0.0387892 0.0140652 31.4013182 2.758 0.00961 **
## days_since_stay_at_home -0.0005531 0.0016779 36.3136250 -0.330 0.74355
## ---
## Signif. codes: 0 '***' 0.001 '**' 0.01 '*' 0.05 '.' 0.1 ' ' 1
##
## Correlation of Fixed Effects:
## (Intr) RADS_t
## RADS_total -0.927
## dys_snc_s__ 0.152 -0.425

standardize_parameters(model.S1a.8)

## # Standardization method: refit
##
## Parameter | Std. Coef. | 95% CI
## ----------------------------------------------------
## (Intercept) | 0.01 | [-0.12, 0.15]
## RADS_total | 0.20 | [ 0.06, 0.34]
## days_since_stay_at_home | -0.02 | [-0.16, 0.11]

#Model comparison with LRT
anova(model.S1a.1, model.S1a.8)

## refitting model(s) with ML (instead of REML)

## Data: df_aim1_all_language
## Models:
## model.S1a.1: i_day_prop ~ RADS_total + (1 | id_participant)
## model.S1a.8: i_day_prop ~ RADS_total + days_since_stay_at_home + (1 | id_participant)
## npar AIC BIC logLik deviance Chisq Df Pr(>Chisq)
## model.S1a.1 4 11974 11997 -5983.1 11966
## model.S1a.8 5 11976 12005 -5983.1 11966 0.1172 1 0.7321

### S1a.9 Medication Status

#First-person pronouns (daily proportion of total words)
 model.S1a.9 <- lmer(formula = i_day_prop ~
 RADS_total
 + Current_Med_Depression
 + (1 |id_participant),
 data = df_aim1_all_language,
 na.action=na.exclude)
summary(model.S1a.9)

## Linear mixed model fit by REML. t-tests use Satterthwaite's method [
## lmerModLmerTest]
## Formula:
## i_day_prop ~ RADS_total + Current_Med_Depression + (1 | id_participant)
## Data: df_aim1_all_language
##
## REML criterion at convergence: 11972.9
##
## Scaled residuals:
## Min 1Q Median 3Q Max
## -3.1221 -0.5764 -0.0160 0.5051 7.4818
##
## Random effects:
## Groups Name Variance Std.Dev.
## id_participant (Intercept) 1.909 1.381
## Residual 11.540 3.397
## Number of obs: 2251, groups: id_participant, 40
##
## Fixed effects:
## Estimate Std. Error df t value Pr(>|t|)
## (Intercept) 5.44057 0.94355 31.38957 5.766 2.29e-06 ***
## RADS_total 0.03889 0.01305 31.36997 2.980 0.00552 **
## Current_Med_Depression -0.39188 0.62433 29.78850 -0.628 0.53499
## ---
## Signif. codes: 0 '***' 0.001 '**' 0.01 '*' 0.05 '.' 0.1 ' ' 1
##
## Correlation of Fixed Effects:
## (Intr) RADS_t
## RADS_total -0.954
## Crrnt_Md_Dp 0.108 -0.255

standardize_parameters(model.S1a.9)

## # Standardization method: refit
##
## Parameter | Std. Coef. | 95% CI
## ---------------------------------------------------
## (Intercept) | -4.04e-03 | [-0.14, 0.14]
## RADS_total | 0.20 | [ 0.07, 0.33]
## Current_Med_Depression | -0.05 | [-0.21, 0.11]

#Model comparison with LRT
anova(model.S1a.1, model.S1a.9)

## refitting model(s) with ML (instead of REML)

## Data: df_aim1_all_language
## Models:
## model.S1a.1: i_day_prop ~ RADS_total + (1 | id_participant)
## model.S1a.9: i_day_prop ~ RADS_total + Current_Med_Depression + (1 | id_participant)
## npar AIC BIC logLik deviance Chisq Df Pr(>Chisq)
## model.S1a.1 4 11974 11997 -5983.1 11966
## model.S1a.9 5 11976 12004 -5982.9 11966 0.459 1 0.4981

## 1c.1 MDD - Negative Emotion Words

#Negative Emotion Words (daily proportion of total words)
 model1c.1 <- lmer(formula = negemo_day_prop ~
 Group
 + (1 |id_participant),
 data = df_aim1_all_language,
 na.action=na.exclude)
summary(model1c.1)

## Linear mixed model fit by REML. t-tests use Satterthwaite's method [
## lmerModLmerTest]
## Formula: negemo_day_prop ~ Group + (1 | id_participant)
## Data: df_aim1_all_language
##
## REML criterion at convergence: 8201.2
##
## Scaled residuals:
## Min 1Q Median 3Q Max
## -2.1937 -0.5804 -0.0889 0.3796 9.9977
##
## Random effects:
## Groups Name Variance Std.Dev.
## id_participant (Intercept) 0.2125 0.4609
## Residual 2.1765 1.4753
## Number of obs: 2251, groups: id_participant, 40
##
## Fixed effects:
## Estimate Std. Error df t value Pr(>|t|)
## (Intercept) 1.5991 0.1594 37.8636 10.031 3.26e-12 ***
## GroupMDD 0.9111 0.1910 36.5820 4.771 2.93e-05 ***
## ---
## Signif. codes: 0 '***' 0.001 '**' 0.01 '*' 0.05 '.' 0.1 ' ' 1
##
## Correlation of Fixed Effects:
## (Intr)
## GroupMDD -0.835

standardize_parameters(model1c.1)

## # Standardization method: refit
##
## Parameter | Std. Coef. | 95% CI
## -----------------------------------------
## (Intercept) | -0.39 | [-0.59, -0.20]
## GroupMDD | 0.58 | [ 0.34, 0.82]

### 1c.2 Age

#Negative Emotion Words (daily proportion of total words)
 model1c.2 <- lmer(formula = negemo_day_prop ~
 Group
 + Age.at.V2
 + (1 |id_participant),
 data = df_aim1_all_language,
 na.action=na.exclude)
summary(model1c.2)

## Linear mixed model fit by REML. t-tests use Satterthwaite's method [
## lmerModLmerTest]
## Formula: negemo_day_prop ~ Group + Age.at.V2 + (1 | id_participant)
## Data: df_aim1_all_language
##
## REML criterion at convergence: 8204.5
##
## Scaled residuals:
## Min 1Q Median 3Q Max
## -2.2017 -0.5790 -0.0892 0.3801 9.9972
##
## Random effects:
## Groups Name Variance Std.Dev.
## id_participant (Intercept) 0.2196 0.4687
## Residual 2.1766 1.4753
## Number of obs: 2251, groups: id_participant, 40
##
## Fixed effects:
## Estimate Std. Error df t value Pr(>|t|)
## (Intercept) 1.89496 1.25402 34.42756 1.511 0.140
## GroupMDD 0.91455 0.19365 35.58758 4.723 3.58e-05 ***
## Age.at.V2 -0.01839 0.07710 34.08743 -0.238 0.813
## ---
## Signif. codes: 0 '***' 0.001 '**' 0.01 '*' 0.05 '.' 0.1 ' ' 1
##
## Correlation of Fixed Effects:
## (Intr) GrpMDD
## GroupMDD -0.058
## Age.at.V2 -0.992 -0.050

standardize_parameters(model1c.2)

## # Standardization method: refit
##
## Parameter | Std. Coef. | 95% CI
## -----------------------------------------
## (Intercept) | -0.39 | [-0.60, -0.19]
## GroupMDD | 0.58 | [ 0.34, 0.83]
## Age.at.V2 | -0.01 | [-0.13, 0.10]

#Model comparison with LRT
anova(model1c.1, model1c.2)

## refitting model(s) with ML (instead of REML)

## Data: df_aim1_all_language
## Models:
## model1c.1: negemo_day_prop ~ Group + (1 | id_participant)
## model1c.2: negemo_day_prop ~ Group + Age.at.V2 + (1 | id_participant)
## npar AIC BIC logLik deviance Chisq Df Pr(>Chisq)
## model1c.1 4 8204.7 8227.6 -4098.3 8196.7
## model1c.2 5 8206.6 8235.2 -4098.3 8196.6 0.0657 1 0.7977

### 1c.3 Sex

#Negative Emotion Words (daily proportion of total words)
 model1c.3 <- lmer(formula = negemo_day_prop ~
 Group
 + Sex
 + (1 |id_participant),
 data = df_aim1_all_language,
 na.action=na.exclude)
summary(model1c.3)

## Linear mixed model fit by REML. t-tests use Satterthwaite's method [
## lmerModLmerTest]
## Formula: negemo_day_prop ~ Group + Sex + (1 | id_participant)
## Data: df_aim1_all_language
##
## REML criterion at convergence: 8202.7
##
## Scaled residuals:
## Min 1Q Median 3Q Max
## -2.1999 -0.5786 -0.0884 0.3783 9.9984
##
## Random effects:
## Groups Name Variance Std.Dev.
## id_participant (Intercept) 0.2211 0.4703
## Residual 2.1764 1.4753
## Number of obs: 2251, groups: id_participant, 40
##
## Fixed effects:
## Estimate Std. Error df t value Pr(>|t|)
## (Intercept) 1.51800 0.32544 32.84152 4.664 4.99e-05 ***
## GroupMDD 0.90763 0.19467 35.89588 4.662 4.22e-05 ***
## Sex 0.05206 0.18319 33.37381 0.284 0.778
## ---
## Signif. codes: 0 '***' 0.001 '**' 0.01 '*' 0.05 '.' 0.1 ' ' 1
##
## Correlation of Fixed Effects:
## (Intr) GrpMDD
## GroupMDD -0.337
## Sex -0.868 -0.088

standardize_parameters(model1c.3)

## # Standardization method: refit
##
## Parameter | Std. Coef. | 95% CI
## -----------------------------------------
## (Intercept) | -0.39 | [-0.60, -0.19]
## GroupMDD | 0.58 | [ 0.34, 0.82]
## Sex | 0.02 | [-0.10, 0.13]

#Model comparison with LRT
anova(model1c.1, model1c.3)

## refitting model(s) with ML (instead of REML)

## Data: df_aim1_all_language
## Models:
## model1c.1: negemo_day_prop ~ Group + (1 | id_participant)
## model1c.3: negemo_day_prop ~ Group + Sex + (1 | id_participant)
## npar AIC BIC logLik deviance Chisq Df Pr(>Chisq)
## model1c.1 4 8204.7 8227.6 -4098.3 8196.7
## model1c.3 5 8206.6 8235.2 -4098.3 8196.6 0.0744 1 0.7851

### 1c.4 Gender

#Negative Emotion Words (daily proportion of total words)
 model1c.4 <- lmer(formula = negemo_day_prop ~
 Group
 + Gender
 + (1 |id_participant),
 data = df_aim1_all_language,
 na.action=na.exclude)
summary(model1c.4)

## Linear mixed model fit by REML. t-tests use Satterthwaite's method [
## lmerModLmerTest]
## Formula: negemo_day_prop ~ Group + Gender + (1 | id_participant)
## Data: df_aim1_all_language
##
## REML criterion at convergence: 8203.3
##
## Scaled residuals:
## Min 1Q Median 3Q Max
## -2.1979 -0.5795 -0.0870 0.3793 9.9933
##
## Random effects:
## Groups Name Variance Std.Dev.
## id_participant (Intercept) 0.2206 0.4697
## Residual 2.1764 1.4753
## Number of obs: 2251, groups: id_participant, 40
##
## Fixed effects:
## Estimate Std. Error df t value Pr(>|t|)
## (Intercept) 1.52157 0.26230 31.89865 5.801 1.96e-06 ***
## GroupMDD 0.89456 0.19961 36.26799 4.482 7.15e-05 ***
## Gender 0.04978 0.13401 30.80794 0.371 0.713
## ---
## Signif. codes: 0 '***' 0.001 '**' 0.01 '*' 0.05 '.' 0.1 ' ' 1
##
## Correlation of Fixed Effects:
## (Intr) GrpMDD
## GroupMDD -0.310
## Gender -0.787 -0.241

standardize_parameters(model1c.4)

## # Standardization method: refit
##
## Parameter | Std. Coef. | 95% CI
## -----------------------------------------
## (Intercept) | -0.38 | [-0.59, -0.17]
## GroupMDD | 0.57 | [ 0.32, 0.82]
## Gender | 0.03 | [-0.11, 0.16]

#Model comparison with LRT
anova(model1c.1, model1c.4)

## refitting model(s) with ML (instead of REML)

## Data: df_aim1_all_language
## Models:
## model1c.1: negemo_day_prop ~ Group + (1 | id_participant)
## model1c.4: negemo_day_prop ~ Group + Gender + (1 | id_participant)
## npar AIC BIC logLik deviance Chisq Df Pr(>Chisq)
## model1c.1 4 8204.7 8227.6 -4098.3 8196.7
## model1c.4 5 8206.5 8235.1 -4098.3 8196.5 0.1406 1 0.7077

### 1c.5 COVID

#Negative Emotion Words (daily proportion of total words)
 model1c.5 <- lmer(formula = negemo_day_prop ~
 Group
 + Collected_During_Covid
 + (1 |id_participant),
 data = df_aim1_all_language,
 na.action=na.exclude)
summary(model1c.5)

## Linear mixed model fit by REML. t-tests use Satterthwaite's method [
## lmerModLmerTest]
## Formula:
## negemo_day_prop ~ Group + Collected_During_Covid + (1 | id_participant)
## Data: df_aim1_all_language
##
## REML criterion at convergence: 8202
##
## Scaled residuals:
## Min 1Q Median 3Q Max
## -2.1861 -0.5825 -0.0911 0.3805 9.9916
##
## Random effects:
## Groups Name Variance Std.Dev.
## id_participant (Intercept) 0.2135 0.4621
## Residual 2.1769 1.4754
## Number of obs: 2251, groups: id_participant, 40
##
## Fixed effects:
## Estimate Std. Error df t value Pr(>|t|)
## (Intercept) 1.5974 0.1597 35.9681 10.000 6.27e-12 ***
## GroupMDD 0.8510 0.2109 34.8604 4.036 0.000283 ***
## Collected_During_Covid 0.1433 0.2108 33.3902 0.680 0.501400
## ---
## Signif. codes: 0 '***' 0.001 '**' 0.01 '*' 0.05 '.' 0.1 ' ' 1
##
## Correlation of Fixed Effects:
## (Intr) GrpMDD
## GroupMDD -0.751
## Cllctd_Dr_C -0.014 -0.420

standardize_parameters(model1c.5)

## # Standardization method: refit
##
## Parameter | Std. Coef. | 95% CI
## ----------------------------------------------------
## (Intercept) | -0.37 | [-0.58, -0.15]
## GroupMDD | 0.54 | [ 0.28, 0.81]
## Collected_During_Covid | 0.04 | [-0.08, 0.16]

#Model comparison with LRT
anova(model1c.1, model1c.5)

## refitting model(s) with ML (instead of REML)

## Data: df_aim1_all_language
## Models:
## model1c.1: negemo_day_prop ~ Group + (1 | id_participant)
## model1c.5: negemo_day_prop ~ Group + Collected_During_Covid + (1 | id_participant)
## npar AIC BIC logLik deviance Chisq Df Pr(>Chisq)
## model1c.1 4 8204.7 8227.6 -4098.3 8196.7
## model1c.5 5 8206.1 8234.7 -4098.1 8196.1 0.5289 1 0.4671

### 1c.6 Number of Messages

#Negative Emotion Words (daily proportion of total words)
 model1c.6 <- lmer(formula = negemo_day_prop ~
 Group
 + n_msg_day
 + (1 |id_participant),
 data = df_aim1_all_language,
 na.action=na.exclude)
summary(model1c.6)

## Linear mixed model fit by REML. t-tests use Satterthwaite's method [
## lmerModLmerTest]
## Formula: negemo_day_prop ~ Group + n_msg_day + (1 | id_participant)
## Data: df_aim1_all_language
##
## REML criterion at convergence: 8215.2
##
## Scaled residuals:
## Min 1Q Median 3Q Max
## -2.1703 -0.5810 -0.0944 0.3778 10.0015
##
## Random effects:
## Groups Name Variance Std.Dev.
## id_participant (Intercept) 0.2159 0.4646
## Residual 2.1759 1.4751
## Number of obs: 2251, groups: id_participant, 40
##
## Fixed effects:
## Estimate Std. Error df t value Pr(>|t|)
## (Intercept) 1.586e+00 1.608e-01 3.847e+01 9.864 4.37e-12 ***
## GroupMDD 8.915e-01 1.930e-01 3.749e+01 4.619 4.44e-05 ***
## n_msg_day 2.217e-04 2.034e-04 1.625e+03 1.090 0.276
## ---
## Signif. codes: 0 '***' 0.001 '**' 0.01 '*' 0.05 '.' 0.1 ' ' 1
##
## Correlation of Fixed Effects:
## (Intr) GrpMDD
## GroupMDD -0.822
## n_msg_day -0.073 -0.096

standardize_parameters(model1c.6)

## # Standardization method: refit
##
## Parameter | Std. Coef. | 95% CI
## -----------------------------------------
## (Intercept) | -0.38 | [-0.58, -0.18]
## GroupMDD | 0.57 | [ 0.33, 0.81]
## n_msg_day | 0.03 | [-0.02, 0.08]

#Model comparison with LRT
anova(model1c.1, model1c.6)

## refitting model(s) with ML (instead of REML)

## Data: df_aim1_all_language
## Models:
## model1c.1: negemo_day_prop ~ Group + (1 | id_participant)
## model1c.6: negemo_day_prop ~ Group + n_msg_day + (1 | id_participant)
## npar AIC BIC logLik deviance Chisq Df Pr(>Chisq)
## model1c.1 4 8204.7 8227.6 -4098.3 8196.7
## model1c.6 5 8205.5 8234.1 -4097.8 8195.5 1.1661 1 0.2802

### 1c.7 Days between V2 and EARS start

#Negative Emotion Words (daily proportion of total words)
 model1c.7 <- lmer(formula = negemo_day_prop ~
 Group
 + days_since_V2
 + (1 |id_participant),
 data = df_aim1_all_language,
 na.action=na.exclude)
summary(model1c.7)

## Linear mixed model fit by REML. t-tests use Satterthwaite's method [
## lmerModLmerTest]
## Formula: negemo_day_prop ~ Group + days_since_V2 + (1 | id_participant)
## Data: df_aim1_all_language
##
## REML criterion at convergence: 8215.2
##
## Scaled residuals:
## Min 1Q Median 3Q Max
## -2.1911 -0.5813 -0.0894 0.3786 9.9946
##
## Random effects:
## Groups Name Variance Std.Dev.
## id_participant (Intercept) 0.2189 0.4679
## Residual 2.1767 1.4754
## Number of obs: 2251, groups: id_participant, 40
##
## Fixed effects:
## Estimate Std. Error df t value Pr(>|t|)
## (Intercept) 1.631e+00 2.053e-01 3.522e+01 7.946 2.30e-09 ***
## GroupMDD 9.063e-01 1.945e-01 3.516e+01 4.660 4.43e-05 ***
## days_since_V2 -9.015e-05 3.510e-04 3.296e+01 -0.257 0.799
## ---
## Signif. codes: 0 '***' 0.001 '**' 0.01 '*' 0.05 '.' 0.1 ' ' 1
##
## Correlation of Fixed Effects:
## (Intr) GrpMDD
## GroupMDD -0.723
## days_snc_V2 -0.619 0.117

standardize_parameters(model1c.7)

## # Standardization method: refit
##
## Parameter | Std. Coef. | 95% CI
## -------------------------------------------
## (Intercept) | -0.39 | [-0.60, -0.19]
## GroupMDD | 0.58 | [ 0.33, 0.82]
## days_since_V2 | -0.01 | [-0.13, 0.10]

#Model comparison with LRT
anova(model1c.1, model1c.7)

## refitting model(s) with ML (instead of REML)

## Data: df_aim1_all_language
## Models:
## model1c.1: negemo_day_prop ~ Group + (1 | id_participant)
## model1c.7: negemo_day_prop ~ Group + days_since_V2 + (1 | id_participant)
## npar AIC BIC logLik deviance Chisq Df Pr(>Chisq)
## model1c.1 4 8204.7 8227.6 -4098.3 8196.7
## model1c.7 5 8206.6 8235.2 -4098.3 8196.6 0.0844 1 0.7714

### 1c.8 Days since COVID stay-at-home order

#Negative Emotion Words (daily proportion of total words)
 model1c.8 <- lmer(formula = negemo_day_prop ~
 Group
 + days_since_stay_at_home
 + (1 |id_participant),
 data = df_aim1_all_language,
 na.action=na.exclude)
summary(model1c.8)

## Linear mixed model fit by REML. t-tests use Satterthwaite's method [
## lmerModLmerTest]
## Formula:
## negemo_day_prop ~ Group + days_since_stay_at_home + (1 | id_participant)
## Data: df_aim1_all_language
##
## REML criterion at convergence: 8213
##
## Scaled residuals:
## Min 1Q Median 3Q Max
## -2.1778 -0.5843 -0.0900 0.3821 9.9917
##
## Random effects:
## Groups Name Variance Std.Dev.
## id_participant (Intercept) 0.2072 0.4551
## Residual 2.1769 1.4754
## Number of obs: 2251, groups: id_participant, 40
##
## Fixed effects:
## Estimate Std. Error df t value Pr(>|t|)
## (Intercept) 1.558e+00 1.622e-01 3.596e+01 9.604 1.83e-11 ***
## GroupMDD 8.116e-01 2.087e-01 3.518e+01 3.888 0.000428 ***
## days_since_stay_at_home 6.548e-04 5.865e-04 3.904e+01 1.116 0.271063
## ---
## Signif. codes: 0 '***' 0.001 '**' 0.01 '*' 0.05 '.' 0.1 ' ' 1
##
## Correlation of Fixed Effects:
## (Intr) GrpMDD
## GroupMDD -0.640
## dys_snc_s__ -0.229 -0.423

standardize_parameters(model1c.8)

## # Standardization method: refit
##
## Parameter | Std. Coef. | 95% CI
## -----------------------------------------------------
## (Intercept) | -0.36 | [-0.56, -0.15]
## GroupMDD | 0.52 | [ 0.26, 0.78]
## days_since_stay_at_home | 0.06 | [-0.05, 0.17]

#Model comparison with LRT
anova(model1c.1, model1c.8)

## refitting model(s) with ML (instead of REML)

## Data: df_aim1_all_language
## Models:
## model1c.1: negemo_day_prop ~ Group + (1 | id_participant)
## model1c.8: negemo_day_prop ~ Group + days_since_stay_at_home + (1 | id_participant)
## npar AIC BIC logLik deviance Chisq Df Pr(>Chisq)
## model1c.1 4 8204.7 8227.6 -4098.3 8196.7
## model1c.8 5 8205.3 8233.9 -4097.7 8195.3 1.3468 1 0.2458

### 1c.9 Medication Status

#Negative Emotion Words (daily proportion of total words)
 model1c.9 <- lmer(formula = negemo_day_prop ~
 Group
 + Current_Med_Depression
 + (1 |id_participant),
 data = df_aim1_all_language,
 na.action=na.exclude)
summary(model1c.9)

## Linear mixed model fit by REML. t-tests use Satterthwaite's method [
## lmerModLmerTest]
## Formula:
## negemo_day_prop ~ Group + Current_Med_Depression + (1 | id_participant)
## Data: df_aim1_all_language
##
## REML criterion at convergence: 8200.2
##
## Scaled residuals:
## Min 1Q Median 3Q Max
## -2.2030 -0.5815 -0.0886 0.3771 9.9926
##
## Random effects:
## Groups Name Variance Std.Dev.
## id_participant (Intercept) 0.2017 0.4491
## Residual 2.1766 1.4753
## Number of obs: 2251, groups: id_participant, 40
##
## Fixed effects:
## Estimate Std. Error df t value Pr(>|t|)
## (Intercept) 1.6001 0.1564 36.4368 10.234 2.91e-12 ***
## GroupMDD 1.0170 0.2004 35.5928 5.075 1.23e-05 ***
## Current_Med_Depression -0.3323 0.2200 30.7864 -1.510 0.141
## ---
## Signif. codes: 0 '***' 0.001 '**' 0.01 '*' 0.05 '.' 0.1 ' ' 1
##
## Correlation of Fixed Effects:
## (Intr) GrpMDD
## GroupMDD -0.780
## Crrnt_Md_Dp 0.000 -0.356

standardize_parameters(model1c.9)

## # Standardization method: refit
##
## Parameter | Std. Coef. | 95% CI
## ----------------------------------------------------
## (Intercept) | -0.47 | [-0.69, -0.25]
## GroupMDD | 0.65 | [ 0.40, 0.90]
## Current_Med_Depression | -0.10 | [-0.23, 0.03]

#Model comparison with LRT
anova(model1c.1, model1c.9)

## refitting model(s) with ML (instead of REML)

## Data: df_aim1_all_language
## Models:
## model1c.1: negemo_day_prop ~ Group + (1 | id_participant)
## model1c.9: negemo_day_prop ~ Group + Current_Med_Depression + (1 | id_participant)
## npar AIC BIC logLik deviance Chisq Df Pr(>Chisq)
## model1c.1 4 8204.7 8227.6 -4098.3 8196.7
## model1c.9 5 8204.3 8232.9 -4097.1 8194.3 2.409 1 0.1206

## S1c.1 Dep Sx - Negative Emotion Words

#Negative Emotion Words (daily proportion of total words)
 model.S1c.1 <- lmer(formula = negemo_day_prop ~
 RADS_total
 + (1 |id_participant),
 data = df_aim1_all_language,
 na.action=na.exclude)
summary(model.S1c.1)

## Linear mixed model fit by REML. t-tests use Satterthwaite's method [
## lmerModLmerTest]
## Formula: negemo_day_prop ~ RADS_total + (1 | id_participant)
## Data: df_aim1_all_language
##
## REML criterion at convergence: 8212.6
##
## Scaled residuals:
## Min 1Q Median 3Q Max
## -2.2606 -0.5817 -0.0889 0.3822 9.9958
##
## Random effects:
## Groups Name Variance Std.Dev.
## id_participant (Intercept) 0.2343 0.484
## Residual 2.1778 1.476
## Number of obs: 2251, groups: id_participant, 40
##
## Fixed effects:
## Estimate Std. Error df t value Pr(>|t|)
## (Intercept) 0.853749 0.342013 33.425330 2.496 0.017653 *
## RADS_total 0.019230 0.004594 32.953465 4.186 0.000198 ***
## ---
## Signif. codes: 0 '***' 0.001 '**' 0.01 '*' 0.05 '.' 0.1 ' ' 1
##
## Correlation of Fixed Effects:
## (Intr)
## RADS_total -0.964

standardize_parameters(model.S1c.1)

## # Standardization method: refit
##
## Parameter | Std. Coef. | 95% CI
## ----------------------------------------
## (Intercept) | 0.03 | [-0.08, 0.15]
## RADS_total | 0.23 | [ 0.12, 0.34]

### S1c.2 Age

#Negative Emotion Words (daily proportion of total words)
 model.S1c.2 <- lmer(formula = negemo_day_prop ~
 RADS_total
 + Age.at.V2
 + (1 |id_participant),
 data = df_aim1_all_language,
 na.action=na.exclude)
summary(model.S1c.2)

## Linear mixed model fit by REML. t-tests use Satterthwaite's method [
## lmerModLmerTest]
## Formula: negemo_day_prop ~ RADS_total + Age.at.V2 + (1 | id_participant)
## Data: df_aim1_all_language
##
## REML criterion at convergence: 8215.6
##
## Scaled residuals:
## Min 1Q Median 3Q Max
## -2.2545 -0.5820 -0.0870 0.3822 9.9930
##
## Random effects:
## Groups Name Variance Std.Dev.
## id_participant (Intercept) 0.2432 0.4932
## Residual 2.1776 1.4757
## Number of obs: 2251, groups: id_participant, 40
##
## Fixed effects:
## Estimate Std. Error df t value Pr(>|t|)
## (Intercept) 0.24380 1.38712 33.08286 0.176 0.861552
## RADS_total 0.01948 0.00469 32.15737 4.154 0.000225 ***
## Age.at.V2 0.03650 0.08062 33.15590 0.453 0.653694
## ---
## Signif. codes: 0 '***' 0.001 '**' 0.01 '*' 0.05 '.' 0.1 ' ' 1
##
## Correlation of Fixed Effects:
## (Intr) RADS_t
## RADS_total -0.343
## Age.at.V2 -0.968 0.106

standardize_parameters(model.S1c.2)

## # Standardization method: refit
##
## Parameter | Std. Coef. | 95% CI
## ----------------------------------------
## (Intercept) | 0.03 | [-0.09, 0.15]
## RADS_total | 0.23 | [ 0.12, 0.34]
## Age.at.V2 | 0.03 | [-0.09, 0.15]

#Model comparison with LRT
anova(model.S1c.1, model.S1c.2)

## refitting model(s) with ML (instead of REML)

## Data: df_aim1_all_language
## Models:
## model.S1c.1: negemo_day_prop ~ RADS_total + (1 | id_participant)
## model.S1c.2: negemo_day_prop ~ RADS_total + Age.at.V2 + (1 | id_participant)
## npar AIC BIC logLik deviance Chisq Df Pr(>Chisq)
## model.S1c.1 4 8208.7 8231.6 -4100.3 8200.7
## model.S1c.2 5 8210.5 8239.1 -4100.2 8200.5 0.2057 1 0.6502

### S1c.3 Sex

#Negative Emotion Words (daily proportion of total words)
 model.S1c.3 <- lmer(formula = negemo_day_prop ~
 RADS_total
 + Sex
 + (1 |id_participant),
 data = df_aim1_all_language,
 na.action=na.exclude)
summary(model.S1c.3)

## Linear mixed model fit by REML. t-tests use Satterthwaite's method [
## lmerModLmerTest]
## Formula: negemo_day_prop ~ RADS_total + Sex + (1 | id_participant)
## Data: df_aim1_all_language
##
## REML criterion at convergence: 8213.6
##
## Scaled residuals:
## Min 1Q Median 3Q Max
## -2.2594 -0.5801 -0.0881 0.3844 9.9899
##
## Random effects:
## Groups Name Variance Std.Dev.
## id_participant (Intercept) 0.2358 0.4855
## Residual 2.1782 1.4759
## Number of obs: 2251, groups: id_participant, 40
##
## Fixed effects:
## Estimate Std. Error df t value Pr(>|t|)
## (Intercept) 0.986722 0.401245 29.617619 2.459 0.019995 *
## RADS_total 0.020198 0.004846 32.283430 4.168 0.000215 ***
## Sex -0.125963 0.196982 32.286631 -0.639 0.527031
## ---
## Signif. codes: 0 '***' 0.001 '**' 0.01 '*' 0.05 '.' 0.1 ' ' 1
##
## Correlation of Fixed Effects:
## (Intr) RADS_t
## RADS_total -0.621
## Sex -0.519 -0.311

standardize_parameters(model.S1c.3)

## # Standardization method: refit
##
## Parameter | Std. Coef. | 95% CI
## ----------------------------------------
## (Intercept) | 0.04 | [-0.08, 0.16]
## RADS_total | 0.24 | [ 0.13, 0.36]
## Sex | -0.04 | [-0.16, 0.08]

#Model comparison with LRT
anova(model.S1c.1, model.S1c.3)

## refitting model(s) with ML (instead of REML)

## Data: df_aim1_all_language
## Models:
## model.S1c.1: negemo_day_prop ~ RADS_total + (1 | id_participant)
## model.S1c.3: negemo_day_prop ~ RADS_total + Sex + (1 | id_participant)
## npar AIC BIC logLik deviance Chisq Df Pr(>Chisq)
## model.S1c.1 4 8208.7 8231.6 -4100.3 8200.7
## model.S1c.3 5 8210.2 8238.8 -4100.1 8200.2 0.4783 1 0.4892

### S1c.4 Gender

#Negative Emotion Words (daily proportion of total words)
 model.S1c.4 <- lmer(formula = negemo_day_prop ~
 RADS_total
 + Gender
 + (1 |id_participant),
 data = df_aim1_all_language,
 na.action=na.exclude)
summary(model.S1c.4)

## Linear mixed model fit by REML. t-tests use Satterthwaite's method [
## lmerModLmerTest]
## Formula: negemo_day_prop ~ RADS_total + Gender + (1 | id_participant)
## Data: df_aim1_all_language
##
## REML criterion at convergence: 8214.4
##
## Scaled residuals:
## Min 1Q Median 3Q Max
## -2.2644 -0.5810 -0.0875 0.3835 9.9979
##
## Random effects:
## Groups Name Variance Std.Dev.
## id_participant (Intercept) 0.2409 0.4908
## Residual 2.1779 1.4758
## Number of obs: 2251, groups: id_participant, 40
##
## Fixed effects:
## Estimate Std. Error df t value Pr(>|t|)
## (Intercept) 0.894397 0.362017 30.877015 2.471 0.019216 *
## RADS_total 0.020135 0.005162 33.406897 3.901 0.000439 ***
## Gender -0.058963 0.149931 30.572624 -0.393 0.696850
## ---
## Signif. codes: 0 '***' 0.001 '**' 0.01 '*' 0.05 '.' 0.1 ' ' 1
##
## Correlation of Fixed Effects:
## (Intr) RADS_t
## RADS_total -0.700
## Gender -0.296 -0.436

standardize_parameters(model.S1c.4)

## # Standardization method: refit
##
## Parameter | Std. Coef. | 95% CI
## ----------------------------------------
## (Intercept) | 0.03 | [-0.08, 0.15]
## RADS_total | 0.24 | [ 0.12, 0.36]
## Gender | -0.03 | [-0.18, 0.12]

#Model comparison with LRT
anova(model.S1c.1, model.S1c.4)

## refitting model(s) with ML (instead of REML)

## Data: df_aim1_all_language
## Models:
## model.S1c.1: negemo_day_prop ~ RADS_total + (1 | id_participant)
## model.S1c.4: negemo_day_prop ~ RADS_total + Gender + (1 | id_participant)
## npar AIC BIC logLik deviance Chisq Df Pr(>Chisq)
## model.S1c.1 4 8208.7 8231.6 -4100.3 8200.7
## model.S1c.4 5 8210.5 8239.1 -4100.2 8200.5 0.182 1 0.6697

### S1c.5 COVID

#Negative Emotion Words (daily proportion of total words)
 model.S1c.5 <- lmer(formula = negemo_day_prop ~
 RADS_total
 + Collected_During_Covid
 + (1 |id_participant),
 data = df_aim1_all_language,
 na.action=na.exclude)
summary(model.S1c.5)

## Linear mixed model fit by REML. t-tests use Satterthwaite's method [
## lmerModLmerTest]
## Formula: negemo_day_prop ~ RADS_total + Collected_During_Covid + (1 |
## id_participant)
## Data: df_aim1_all_language
##
## REML criterion at convergence: 8213
##
## Scaled residuals:
## Min 1Q Median 3Q Max
## -2.2444 -0.5829 -0.0880 0.3863 9.9887
##
## Random effects:
## Groups Name Variance Std.Dev.
## id_participant (Intercept) 0.2326 0.4823
## Residual 2.1782 1.4759
## Number of obs: 2251, groups: id_participant, 40
##
## Fixed effects:
## Estimate Std. Error df t value Pr(>|t|)
## (Intercept) 0.926006 0.350311 31.894567 2.643 0.01262 *
## RADS_total 0.017402 0.005011 31.560953 3.473 0.00152 **
## Collected_During_Covid 0.194096 0.216395 32.486986 0.897 0.37634
## ---
## Signif. codes: 0 '***' 0.001 '**' 0.01 '*' 0.05 '.' 0.1 ' ' 1
##
## Correlation of Fixed Effects:
## (Intr) RADS_t
## RADS_total -0.950
## Cllctd_Dr_C 0.229 -0.406

standardize_parameters(model.S1c.5)

## # Standardization method: refit
##
## Parameter | Std. Coef. | 95% CI
## ---------------------------------------------------
## (Intercept) | 0.03 | [-0.08, 0.15]
## RADS_total | 0.21 | [ 0.09, 0.33]
## Collected_During_Covid | 0.06 | [-0.07, 0.18]

#Model comparison with LRT
anova(model.S1c.1, model.S1c.5)

## refitting model(s) with ML (instead of REML)

## Data: df_aim1_all_language
## Models:
## model.S1c.1: negemo_day_prop ~ RADS_total + (1 | id_participant)
## model.S1c.5: negemo_day_prop ~ RADS_total + Collected_During_Covid + (1 | id_participant)
## npar AIC BIC logLik deviance Chisq Df Pr(>Chisq)
## model.S1c.1 4 8208.7 8231.6 -4100.3 8200.7
## model.S1c.5 5 8209.8 8238.4 -4099.9 8199.8 0.8939 1 0.3444

### S1c.6 Number of Messages

#Negative Emotion Words (daily proportion of total words)
 model.S1c.6 <- lmer(formula = negemo_day_prop ~
 RADS_total
 + n_msg_day
 + (1 |id_participant),
 data = df_aim1_all_language,
 na.action=na.exclude)
summary(model.S1c.6)

## Linear mixed model fit by REML. t-tests use Satterthwaite's method [
## lmerModLmerTest]
## Formula: negemo_day_prop ~ RADS_total + n_msg_day + (1 | id_participant)
## Data: df_aim1_all_language
##
## REML criterion at convergence: 8226.4
##
## Scaled residuals:
## Min 1Q Median 3Q Max
## -2.2331 -0.5801 -0.0971 0.3850 9.9999
##
## Random effects:
## Groups Name Variance Std.Dev.
## id_participant (Intercept) 0.2371 0.4869
## Residual 2.1771 1.4755
## Number of obs: 2251, groups: id_participant, 40
##
## Fixed effects:
## Estimate Std. Error df t value Pr(>|t|)
## (Intercept) 8.589e-01 3.437e-01 3.376e+01 2.499 0.017476 *
## RADS_total 1.876e-02 4.634e-03 3.380e+01 4.049 0.000284 ***
## n_msg_day 2.354e-04 2.040e-04 1.684e+03 1.154 0.248679
## ---
## Signif. codes: 0 '***' 0.001 '**' 0.01 '*' 0.05 '.' 0.1 ' ' 1
##
## Correlation of Fixed Effects:
## (Intr) RADS_t
## RADS_total -0.961
## n_msg_day 0.015 -0.089

standardize_parameters(model.S1c.6)

## # Standardization method: refit
##
## Parameter | Std. Coef. | 95% CI
## ----------------------------------------
## (Intercept) | 0.04 | [-0.08, 0.15]
## RADS_total | 0.22 | [ 0.12, 0.33]
## n_msg_day | 0.03 | [-0.02, 0.08]

#Model comparison with LRT
anova(model.S1c.1, model.S1c.6)

## refitting model(s) with ML (instead of REML)

## Data: df_aim1_all_language
## Models:
## model.S1c.1: negemo_day_prop ~ RADS_total + (1 | id_participant)
## model.S1c.6: negemo_day_prop ~ RADS_total + n_msg_day + (1 | id_participant)
## npar AIC BIC logLik deviance Chisq Df Pr(>Chisq)
## model.S1c.1 4 8208.7 8231.6 -4100.3 8200.7
## model.S1c.6 5 8209.4 8238.0 -4099.7 8199.4 1.3159 1 0.2513

### S1c.7 Days between fMRI and EARS start

#Negative Emotion Words (daily proportion of total words)
 model.S1c.7 <- lmer(formula = negemo_day_prop ~
 RADS_total
 + days_since_V2
 + (1 |id_participant),
 data = df_aim1_all_language,
 na.action=na.exclude)
summary(model.S1c.7)

## Linear mixed model fit by REML. t-tests use Satterthwaite's method [
## lmerModLmerTest]
## Formula: negemo_day_prop ~ RADS_total + days_since_V2 + (1 | id_participant)
## Data: df_aim1_all_language
##
## REML criterion at convergence: 8226
##
## Scaled residuals:
## Min 1Q Median 3Q Max
## -2.2457 -0.5820 -0.0880 0.3864 9.9902
##
## Random effects:
## Groups Name Variance Std.Dev.
## id_participant (Intercept) 0.2354 0.4851
## Residual 2.1780 1.4758
## Number of obs: 2251, groups: id_participant, 40
##
## Fixed effects:
## Estimate Std. Error df t value Pr(>|t|)
## (Intercept) 0.9414070 0.3610057 32.0225350 2.608 0.013730 *
## RADS_total 0.0192332 0.0046024 31.5161604 4.179 0.000216 ***
## days_since_V2 -0.0002773 0.0003587 31.7485802 -0.773 0.445235
## ---
## Signif. codes: 0 '***' 0.001 '**' 0.01 '*' 0.05 '.' 0.1 ' ' 1
##
## Correlation of Fixed Effects:
## (Intr) RADS_t
## RADS_total -0.915
## days_snc_V2 -0.315 0.000

standardize_parameters(model.S1c.7)

## # Standardization method: refit
##
## Parameter | Std. Coef. | 95% CI
## ------------------------------------------
## (Intercept) | 0.03 | [-0.08, 0.15]
## RADS_total | 0.23 | [ 0.12, 0.34]
## days_since_V2 | -0.05 | [-0.16, 0.07]

#Model comparison with LRT
anova(model.S1c.1, model.S1c.7)

## refitting model(s) with ML (instead of REML)

## Data: df_aim1_all_language
## Models:
## model.S1c.1: negemo_day_prop ~ RADS_total + (1 | id_participant)
## model.S1c.7: negemo_day_prop ~ RADS_total + days_since_V2 + (1 | id_participant)
## npar AIC BIC logLik deviance Chisq Df Pr(>Chisq)
## model.S1c.1 4 8208.7 8231.6 -4100.3 8200.7
## model.S1c.7 5 8210.0 8238.6 -4100.0 8200.0 0.6689 1 0.4134

### S1c.8 Days since COVID stay-at-home order

#Negative Emotion Words (daily proportion of total words)
 model.S1c.8 <- lmer(formula = negemo_day_prop ~
 RADS_total
 + days_since_stay_at_home
 + (1 |id_participant),
 data = df_aim1_all_language,
 na.action=na.exclude)
summary(model.S1c.8)

## Linear mixed model fit by REML. t-tests use Satterthwaite's method [
## lmerModLmerTest]
## Formula: negemo_day_prop ~ RADS_total + days_since_stay_at_home + (1 |
## id_participant)
## Data: df_aim1_all_language
##
## REML criterion at convergence: 8224
##
## Scaled residuals:
## Min 1Q Median 3Q Max
## -2.2354 -0.5838 -0.0875 0.3860 9.9894
##
## Random effects:
## Groups Name Variance Std.Dev.
## id_participant (Intercept) 0.2253 0.4747
## Residual 2.1782 1.4759
## Number of obs: 2251, groups: id_participant, 40
##
## Fixed effects:
## Estimate Std. Error df t value Pr(>|t|)
## (Intercept) 9.190e-01 3.405e-01 3.150e+01 2.699 0.01108 *
## RADS_total 1.653e-02 4.994e-03 3.094e+01 3.309 0.00238 **
## days_since_stay_at_home 7.653e-04 6.050e-04 3.674e+01 1.265 0.21386
## ---
## Signif. codes: 0 '***' 0.001 '**' 0.01 '*' 0.05 '.' 0.1 ' ' 1
##
## Correlation of Fixed Effects:
## (Intr) RADS_t
## RADS_total -0.926
## dys_snc_s__ 0.147 -0.424

standardize_parameters(model.S1c.8)

## # Standardization method: refit
##
## Parameter | Std. Coef. | 95% CI
## ----------------------------------------------------
## (Intercept) | 0.03 | [-0.09, 0.14]
## RADS_total | 0.20 | [ 0.08, 0.32]
## days_since_stay_at_home | 0.07 | [-0.04, 0.19]

#Model comparison with LRT
anova(model.S1c.1, model.S1c.8)

## refitting model(s) with ML (instead of REML)

## Data: df_aim1_all_language
## Models:
## model.S1c.1: negemo_day_prop ~ RADS_total + (1 | id_participant)
## model.S1c.8: negemo_day_prop ~ RADS_total + days_since_stay_at_home + (1 | id_participant)
## npar AIC BIC logLik deviance Chisq Df Pr(>Chisq)
## model.S1c.1 4 8208.7 8231.6 -4100.3 8200.7
## model.S1c.8 5 8209.0 8237.6 -4099.5 8199.0 1.7187 1 0.1899

### S1c.9 Medication Status

#Negative Emotion Words (daily proportion of total words)
 model.S1c.9 <- lmer(formula = negemo_day_prop ~
 RADS_total
 + Current_Med_Depression
 + (1 |id_participant),
 data = df_aim1_all_language,
 na.action=na.exclude)
summary(model.S1c.9)

## Linear mixed model fit by REML. t-tests use Satterthwaite's method [
## lmerModLmerTest]
## Formula: negemo_day_prop ~ RADS_total + Current_Med_Depression + (1 |
## id_participant)
## Data: df_aim1_all_language
##
## REML criterion at convergence: 8213.1
##
## Scaled residuals:
## Min 1Q Median 3Q Max
## -2.2703 -0.5807 -0.0873 0.3828 9.9922
##
## Random effects:
## Groups Name Variance Std.Dev.
## id_participant (Intercept) 0.2341 0.4839
## Residual 2.1781 1.4758
## Number of obs: 2251, groups: id_participant, 40
##
## Fixed effects:
## Estimate Std. Error df t value Pr(>|t|)
## (Intercept) 0.821197 0.344209 32.306897 2.386 0.023072 *
## RADS_total 0.020267 0.004763 32.584446 4.255 0.000165 ***
## Current_Med_Depression -0.186446 0.226672 30.525797 -0.823 0.417151
## ---
## Signif. codes: 0 '***' 0.001 '**' 0.01 '*' 0.05 '.' 0.1 ' ' 1
##
## Correlation of Fixed Effects:
## (Intr) RADS_t
## RADS_total -0.954
## Crrnt_Md_Dp 0.115 -0.265

standardize_parameters(model.S1c.9)

## # Standardization method: refit
##
## Parameter | Std. Coef. | 95% CI
## ---------------------------------------------------
## (Intercept) | 0.02 | [-0.10, 0.14]
## RADS_total | 0.24 | [ 0.13, 0.35]
## Current_Med_Depression | -0.06 | [-0.19, 0.08]

#Model comparison with LRT
anova(model.S1c.1, model.S1c.9)

## refitting model(s) with ML (instead of REML)

## Data: df_aim1_all_language
## Models:
## model.S1c.1: negemo_day_prop ~ RADS_total + (1 | id_participant)
## model.S1c.9: negemo_day_prop ~ RADS_total + Current_Med_Depression + (1 | id_participant)
## npar AIC BIC logLik deviance Chisq Df Pr(>Chisq)
## model.S1c.1 4 8208.7 8231.6 -4100.3 8200.7
## model.S1c.9 5 8209.9 8238.5 -4100.0 8199.9 0.7584 1 0.3838

## 1f.1 MDD - Future Focus Words

#Future focus words (daily proportion of total words)
 model1f.1 <- lmer(formula = focusfuture_day_prop ~
 Group
 + (1 |id_participant),
 data = df_aim1_all_language,
 na.action=na.exclude)
summary(model1f.1)

## Linear mixed model fit by REML. t-tests use Satterthwaite's method [
## lmerModLmerTest]
## Formula: focusfuture_day_prop ~ Group + (1 | id_participant)
## Data: df_aim1_all_language
##
## REML criterion at convergence: 6913.1
##
## Scaled residuals:
## Min 1Q Median 3Q Max
## -1.9359 -0.6197 -0.0888 0.4275 8.0243
##
## Random effects:
## Groups Name Variance Std.Dev.
## id_participant (Intercept) 0.1233 0.3512
## Residual 1.2271 1.1077
## Number of obs: 2251, groups: id_participant, 40
##
## Fixed effects:
## Estimate Std. Error df t value Pr(>|t|)
## (Intercept) 1.7058 0.1210 28.4992 14.096 2.24e-14 ***
## GroupMDD -0.3166 0.1450 27.5340 -2.183 0.0377 *
## ---
## Signif. codes: 0 '***' 0.001 '**' 0.01 '*' 0.05 '.' 0.1 ' ' 1
##
## Correlation of Fixed Effects:
## (Intr)
## GroupMDD -0.835

standardize_parameters(model1f.1)

## # Standardization method: refit
##
## Parameter | Std. Coef. | 95% CI
## -----------------------------------------
## (Intercept) | 0.26 | [ 0.05, 0.46]
## GroupMDD | -0.27 | [-0.52, -0.03]

### 1f.2 Age

#Future focus words (daily proportion of total words)
 model1f.2 <- lmer(formula = focusfuture_day_prop ~
 Group
 + Age.at.V2
 + (1 |id_participant),
 data = df_aim1_all_language,
 na.action=na.exclude)
summary(model1f.2)

## Linear mixed model fit by REML. t-tests use Satterthwaite's method [
## lmerModLmerTest]
## Formula: focusfuture_day_prop ~ Group + Age.at.V2 + (1 | id_participant)
## Data: df_aim1_all_language
##
## REML criterion at convergence: 6911.4
##
## Scaled residuals:
## Min 1Q Median 3Q Max
## -1.9457 -0.6249 -0.0874 0.4252 8.0284
##
## Random effects:
## Groups Name Variance Std.Dev.
## id_participant (Intercept) 0.1106 0.3326
## Residual 1.2261 1.1073
## Number of obs: 2251, groups: id_participant, 40
##
## Fixed effects:
## Estimate Std. Error df t value Pr(>|t|)
## (Intercept) -0.43603 0.90141 26.50122 -0.484 0.6326
## GroupMDD -0.33585 0.13930 27.47173 -2.411 0.0229 *
## Age.at.V2 0.13286 0.05541 26.25145 2.398 0.0239 *
## ---
## Signif. codes: 0 '***' 0.001 '**' 0.01 '*' 0.05 '.' 0.1 ' ' 1
##
## Correlation of Fixed Effects:
## (Intr) GrpMDD
## GroupMDD -0.059
## Age.at.V2 -0.992 -0.049

standardize_parameters(model1f.2)

## # Standardization method: refit
##
## Parameter | Std. Coef. | 95% CI
## -----------------------------------------
## (Intercept) | 0.24 | [ 0.05, 0.44]
## GroupMDD | -0.29 | [-0.53, -0.05]
## Age.at.V2 | 0.14 | [ 0.03, 0.25]

#Model comparison with LRT
anova(model1f.1, model1f.2)

## refitting model(s) with ML (instead of REML)

## Data: df_aim1_all_language
## Models:
## model1f.1: focusfuture_day_prop ~ Group + (1 | id_participant)
## model1f.2: focusfuture_day_prop ~ Group + Age.at.V2 + (1 | id_participant)
## npar AIC BIC logLik deviance Chisq Df Pr(>Chisq)
## model1f.1 4 6915.5 6938.3 -3453.7 6907.5
## model1f.2 5 6911.6 6940.1 -3450.8 6901.6 5.9112 1 0.01504 *
## ---
## Signif. codes: 0 '***' 0.001 '**' 0.01 '*' 0.05 '.' 0.1 ' ' 1

### 1f.3 Sex

#Future focus words (daily proportion of total words)
 model1f.3 <- lmer(formula = focusfuture_day_prop ~
 Group
 + Sex
 + (1 |id_participant),
 data = df_aim1_all_language,
 na.action=na.exclude)
summary(model1f.3)

## Linear mixed model fit by REML. t-tests use Satterthwaite's method [
## lmerModLmerTest]
## Formula: focusfuture_day_prop ~ Group + Sex + (1 | id_participant)
## Data: df_aim1_all_language
##
## REML criterion at convergence: 6914.9
##
## Scaled residuals:
## Min 1Q Median 3Q Max
## -1.9309 -0.6249 -0.0883 0.4248 8.0228
##
## Random effects:
## Groups Name Variance Std.Dev.
## id_participant (Intercept) 0.1305 0.3612
## Residual 1.2266 1.1075
## Number of obs: 2251, groups: id_participant, 40
##
## Fixed effects:
## Estimate Std. Error df t value Pr(>|t|)
## (Intercept) 1.57124 0.24883 24.92117 6.314 1.33e-06 ***
## GroupMDD -0.32312 0.14872 27.28533 -2.173 0.0387 *
## Sex 0.08675 0.14005 25.36953 0.619 0.5412
## ---
## Signif. codes: 0 '***' 0.001 '**' 0.01 '*' 0.05 '.' 0.1 ' ' 1
##
## Correlation of Fixed Effects:
## (Intr) GrpMDD
## GroupMDD -0.337
## Sex -0.868 -0.088

standardize_parameters(model1f.3)

## # Standardization method: refit
##
## Parameter | Std. Coef. | 95% CI
## -----------------------------------------
## (Intercept) | 0.26 | [ 0.05, 0.47]
## GroupMDD | -0.28 | [-0.53, -0.03]
## Sex | 0.04 | [-0.08, 0.16]

#Model comparison with LRT
anova(model1f.1, model1f.3)

## refitting model(s) with ML (instead of REML)

## Data: df_aim1_all_language
## Models:
## model1f.1: focusfuture_day_prop ~ Group + (1 | id_participant)
## model1f.3: focusfuture_day_prop ~ Group + Sex + (1 | id_participant)
## npar AIC BIC logLik deviance Chisq Df Pr(>Chisq)
## model1f.1 4 6915.5 6938.3 -3453.7 6907.5
## model1f.3 5 6917.1 6945.7 -3453.5 6907.1 0.3658 1 0.5453

### 1f.4 Gender

#Future focus words (daily proportion of total words)
 model1f.4 <- lmer(formula = focusfuture_day_prop ~
 Group
 + Gender
 + (1 |id_participant),
 data = df_aim1_all_language,
 na.action=na.exclude)
summary(model1f.4)

## Linear mixed model fit by REML. t-tests use Satterthwaite's method [
## lmerModLmerTest]
## Formula: focusfuture_day_prop ~ Group + Gender + (1 | id_participant)
## Data: df_aim1_all_language
##
## REML criterion at convergence: 6915.7
##
## Scaled residuals:
## Min 1Q Median 3Q Max
## -1.9445 -0.6221 -0.0881 0.4268 8.0276
##
## Random effects:
## Groups Name Variance Std.Dev.
## id_participant (Intercept) 0.1265 0.3556
## Residual 1.2272 1.1078
## Number of obs: 2251, groups: id_participant, 40
##
## Fixed effects:
## Estimate Std. Error df t value Pr(>|t|)
## (Intercept) 1.77471 0.19828 23.02440 8.950 5.88e-09 ***
## GroupMDD -0.29981 0.15083 26.19681 -1.988 0.0574 .
## Gender -0.04493 0.10131 22.25294 -0.444 0.6617
## ---
## Signif. codes: 0 '***' 0.001 '**' 0.01 '*' 0.05 '.' 0.1 ' ' 1
##
## Correlation of Fixed Effects:
## (Intr) GrpMDD
## GroupMDD -0.310
## Gender -0.788 -0.240

standardize_parameters(model1f.4)

## # Standardization method: refit
##
## Parameter | Std. Coef. | 95% CI
## -----------------------------------------
## (Intercept) | 0.25 | [ 0.03, 0.46]
## GroupMDD | -0.26 | [-0.52, 0.00]
## Gender | -0.03 | [-0.17, 0.11]

#Model comparison with LRT
anova(model1f.1, model1f.4)

## refitting model(s) with ML (instead of REML)

## Data: df_aim1_all_language
## Models:
## model1f.1: focusfuture_day_prop ~ Group + (1 | id_participant)
## model1f.4: focusfuture_day_prop ~ Group + Gender + (1 | id_participant)
## npar AIC BIC logLik deviance Chisq Df Pr(>Chisq)
## model1f.1 4 6915.5 6938.3 -3453.7 6907.5
## model1f.4 5 6917.2 6945.8 -3453.6 6907.2 0.2615 1 0.6091

### 1f.5 COVID

#Future focus words (daily proportion of total words)
 model1f.5 <- lmer(formula = focusfuture_day_prop ~
 Group
 + Collected_During_Covid
 + (1 |id_participant),
 data = df_aim1_all_language,
 na.action=na.exclude)
summary(model1f.5)

## Linear mixed model fit by REML. t-tests use Satterthwaite's method [
## lmerModLmerTest]
## Formula: focusfuture_day_prop ~ Group + Collected_During_Covid + (1 |
## id_participant)
## Data: df_aim1_all_language
##
## REML criterion at convergence: 6914.6
##
## Scaled residuals:
## Min 1Q Median 3Q Max
## -1.9414 -0.6215 -0.0887 0.4257 8.0269
##
## Random effects:
## Groups Name Variance Std.Dev.
## id_participant (Intercept) 0.1293 0.3596
## Residual 1.2268 1.1076
## Number of obs: 2251, groups: id_participant, 40
##
## Fixed effects:
## Estimate Std. Error df t value Pr(>|t|)
## (Intercept) 1.70623 0.12320 28.09038 13.849 4.46e-14 ***
## GroupMDD -0.27398 0.16269 27.28803 -1.684 0.104
## Collected_During_Covid -0.09836 0.16274 26.11569 -0.604 0.551
## ---
## Signif. codes: 0 '***' 0.001 '**' 0.01 '*' 0.05 '.' 0.1 ' ' 1
##
## Correlation of Fixed Effects:
## (Intr) GrpMDD
## GroupMDD -0.751
## Cllctd_Dr_C -0.015 -0.420

standardize_parameters(model1f.5)

## # Standardization method: refit
##
## Parameter | Std. Coef. | 95% CI
## ---------------------------------------------------
## (Intercept) | 0.23 | [ 0.01, 0.46]
## GroupMDD | -0.24 | [-0.51, 0.04]
## Collected_During_Covid | -0.04 | [-0.17, 0.09]

#Model comparison with LRT
anova(model1f.1, model1f.5)

## refitting model(s) with ML (instead of REML)

## Data: df_aim1_all_language
## Models:
## model1f.1: focusfuture_day_prop ~ Group + (1 | id_participant)
## model1f.5: focusfuture_day_prop ~ Group + Collected_During_Covid + (1 | id_participant)
## npar AIC BIC logLik deviance Chisq Df Pr(>Chisq)
## model1f.1 4 6915.5 6938.3 -3453.7 6907.5
## model1f.5 5 6917.1 6945.7 -3453.5 6907.1 0.3675 1 0.5444

### 1f.6 Number of Messages

#Future focus words (daily proportion of total words)
 model1f.6 <- lmer(formula = focusfuture_day_prop ~
 Group
 + n_msg_day
 + (1 |id_participant),
 data = df_aim1_all_language,
 na.action=na.exclude)
summary(model1f.6)

## Linear mixed model fit by REML. t-tests use Satterthwaite's method [
## lmerModLmerTest]
## Formula: focusfuture_day_prop ~ Group + n_msg_day + (1 | id_participant)
## Data: df_aim1_all_language
##
## REML criterion at convergence: 6927.9
##
## Scaled residuals:
## Min 1Q Median 3Q Max
## -1.9341 -0.6207 -0.0943 0.4230 8.0326
##
## Random effects:
## Groups Name Variance Std.Dev.
## id_participant (Intercept) 0.1221 0.3494
## Residual 1.2272 1.1078
## Number of obs: 2251, groups: id_participant, 40
##
## Fixed effects:
## Estimate Std. Error df t value Pr(>|t|)
## (Intercept) 1.697e+00 1.209e-01 2.849e+01 14.041 2.49e-14 ***
## GroupMDD -3.307e-01 1.451e-01 2.775e+01 -2.279 0.0305 *
## n_msg_day 1.528e-04 1.528e-04 1.481e+03 1.000 0.3174
## ---
## Signif. codes: 0 '***' 0.001 '**' 0.01 '*' 0.05 '.' 0.1 ' ' 1
##
## Correlation of Fixed Effects:
## (Intr) GrpMDD
## GroupMDD -0.822
## n_msg_day -0.072 -0.096

standardize_parameters(model1f.6)

## # Standardization method: refit
##
## Parameter | Std. Coef. | 95% CI
## -----------------------------------------
## (Intercept) | 0.27 | [ 0.06, 0.48]
## GroupMDD | -0.29 | [-0.53, -0.04]
## n_msg_day | 0.03 | [-0.03, 0.08]

#Model comparison with LRT
anova(model1f.1, model1f.6)

## refitting model(s) with ML (instead of REML)

## Data: df_aim1_all_language
## Models:
## model1f.1: focusfuture_day_prop ~ Group + (1 | id_participant)
## model1f.6: focusfuture_day_prop ~ Group + n_msg_day + (1 | id_participant)
## npar AIC BIC logLik deviance Chisq Df Pr(>Chisq)
## model1f.1 4 6915.5 6938.3 -3453.7 6907.5
## model1f.6 5 6916.4 6945.0 -3453.2 6906.4 1.0232 1 0.3118

### 1f.7 Days between V2 and EARS start

#Future focus words (daily proportion of total words)
 model1f.7 <- lmer(formula = focusfuture_day_prop ~
 Group
 + days_since_V2
 + (1 |id_participant),
 data = df_aim1_all_language,
 na.action=na.exclude)
summary(model1f.7)

## Linear mixed model fit by REML. t-tests use Satterthwaite's method [
## lmerModLmerTest]
## Formula: focusfuture_day_prop ~ Group + days_since_V2 + (1 | id_participant)
## Data: df_aim1_all_language
##
## REML criterion at convergence: 6927.5
##
## Scaled residuals:
## Min 1Q Median 3Q Max
## -1.9368 -0.6192 -0.0894 0.4268 8.0248
##
## Random effects:
## Groups Name Variance Std.Dev.
## id_participant (Intercept) 0.1286 0.3585
## Residual 1.2269 1.1077
## Number of obs: 2251, groups: id_participant, 40
##
## Fixed effects:
## Estimate Std. Error df t value Pr(>|t|)
## (Intercept) 1.6576725 0.1565546 26.9348789 10.588 4.22e-11 ***
## GroupMDD -0.3069370 0.1483392 26.8547712 -2.069 0.0483 *
## days_since_V2 0.0001313 0.0002678 25.1628874 0.490 0.6281
## ---
## Signif. codes: 0 '***' 0.001 '**' 0.01 '*' 0.05 '.' 0.1 ' ' 1
##
## Correlation of Fixed Effects:
## (Intr) GrpMDD
## GroupMDD -0.723
## days_snc_V2 -0.619 0.117

standardize_parameters(model1f.7)

## # Standardization method: refit
##
## Parameter | Std. Coef. | 95% CI
## -------------------------------------------
## (Intercept) | 0.26 | [ 0.05, 0.46]
## GroupMDD | -0.27 | [-0.52, -0.01]
## days_since_V2 | 0.03 | [-0.09, 0.15]

#Model comparison with LRT
anova(model1f.1, model1f.7)

## refitting model(s) with ML (instead of REML)

## Data: df_aim1_all_language
## Models:
## model1f.1: focusfuture_day_prop ~ Group + (1 | id_participant)
## model1f.7: focusfuture_day_prop ~ Group + days_since_V2 + (1 | id_participant)
## npar AIC BIC logLik deviance Chisq Df Pr(>Chisq)
## model1f.1 4 6915.5 6938.3 -3453.7 6907.5
## model1f.7 5 6917.2 6945.8 -3453.6 6907.2 0.2585 1 0.6112

### 1f.8 Days since COVID stay-at-home order

#Future focus words (daily proportion of total words)
 model1f.8 <- lmer(formula = focusfuture_day_prop ~
 Group
 + days_since_stay_at_home
 + (1 |id_participant),
 data = df_aim1_all_language,
 na.action=na.exclude)
summary(model1f.8)

## Linear mixed model fit by REML. t-tests use Satterthwaite's method [
## lmerModLmerTest]
## Formula: focusfuture_day_prop ~ Group + days_since_stay_at_home + (1 |
## id_participant)
## Data: df_aim1_all_language
##
## REML criterion at convergence: 6926.4
##
## Scaled residuals:
## Min 1Q Median 3Q Max
## -1.9419 -0.6218 -0.0887 0.4243 8.0269
##
## Random effects:
## Groups Name Variance Std.Dev.
## id_participant (Intercept) 0.1292 0.3594
## Residual 1.2268 1.1076
## Number of obs: 2251, groups: id_participant, 40
##
## Fixed effects:
## Estimate Std. Error df t value Pr(>|t|)
## (Intercept) 1.7215338 0.1265125 28.0576143 13.608 7.02e-14 ***
## GroupMDD -0.2764645 0.1628301 27.5608456 -1.698 0.101
## days_since_stay_at_home -0.0002579 0.0004566 30.4724286 -0.565 0.576
## ---
## Signif. codes: 0 '***' 0.001 '**' 0.01 '*' 0.05 '.' 0.1 ' ' 1
##
## Correlation of Fixed Effects:
## (Intr) GrpMDD
## GroupMDD -0.639
## dys_snc_s__ -0.229 -0.422

standardize_parameters(model1f.8)

## # Standardization method: refit
##
## Parameter | Std. Coef. | 95% CI
## ----------------------------------------------------
## (Intercept) | 0.24 | [ 0.02, 0.46]
## GroupMDD | -0.24 | [-0.52, 0.04]
## days_since_stay_at_home | -0.03 | [-0.15, 0.08]

#Model comparison with LRT
anova(model1f.1, model1f.8)

## refitting model(s) with ML (instead of REML)

## Data: df_aim1_all_language
## Models:
## model1f.1: focusfuture_day_prop ~ Group + (1 | id_participant)
## model1f.8: focusfuture_day_prop ~ Group + days_since_stay_at_home + (1 | id_participant)
## npar AIC BIC logLik deviance Chisq Df Pr(>Chisq)
## model1f.1 4 6915.5 6938.3 -3453.7 6907.5
## model1f.8 5 6917.2 6945.7 -3453.6 6907.2 0.3151 1 0.5746

### 1f.9 Medication Status

#Future focus words (daily proportion of total words)
 model1f.9 <- lmer(formula = focusfuture_day_prop ~
 Group
 + Current_Med_Depression
 + (1 |id_participant),
 data = df_aim1_all_language,
 na.action=na.exclude)
summary(model1f.9)

## Linear mixed model fit by REML. t-tests use Satterthwaite's method [
## lmerModLmerTest]
## Formula: focusfuture_day_prop ~ Group + Current_Med_Depression + (1 |
## id_participant)
## Data: df_aim1_all_language
##
## REML criterion at convergence: 6909.9
##
## Scaled residuals:
## Min 1Q Median 3Q Max
## -1.9260 -0.6249 -0.0820 0.4274 8.0236
##
## Random effects:
## Groups Name Variance Std.Dev.
## id_participant (Intercept) 0.1118 0.3343
## Residual 1.2263 1.1074
## Number of obs: 2251, groups: id_participant, 40
##
## Fixed effects:
## Estimate Std. Error df t value Pr(>|t|)
## (Intercept) 1.7071 0.1166 29.5349 14.635 4.51e-15 ***
## GroupMDD -0.4400 0.1495 28.8510 -2.944 0.00634 **
## Current_Med_Depression 0.3722 0.1641 24.9216 2.268 0.03223 *
## ---
## Signif. codes: 0 '***' 0.001 '**' 0.01 '*' 0.05 '.' 0.1 ' ' 1
##
## Correlation of Fixed Effects:
## (Intr) GrpMDD
## GroupMDD -0.780
## Crrnt_Md_Dp 0.000 -0.356

standardize_parameters(model1f.9)

## # Standardization method: refit
##
## Parameter | Std. Coef. | 95% CI
## ----------------------------------------------------
## (Intercept) | 0.38 | [ 0.16, 0.60]
## GroupMDD | -0.38 | [-0.63, -0.13]
## Current_Med_Depression | 0.16 | [ 0.02, 0.29]

#Model comparison with LRT
anova(model1f.1, model1f.9)

## refitting model(s) with ML (instead of REML)

## Data: df_aim1_all_language
## Models:
## model1f.1: focusfuture_day_prop ~ Group + (1 | id_participant)
## model1f.9: focusfuture_day_prop ~ Group + Current_Med_Depression + (1 | id_participant)
## npar AIC BIC logLik deviance Chisq Df Pr(>Chisq)
## model1f.1 4 6915.5 6938.3 -3453.7 6907.5
## model1f.9 5 6912.2 6940.8 -3451.1 6902.2 5.3077 1 0.02123 *
## ---
## Signif. codes: 0 '***' 0.001 '**' 0.01 '*' 0.05 '.' 0.1 ' ' 1

## S1f.1 Dep Sx - Future Focus Words

#Future focus words (daily proportion of total words)
 model.S1f.1 <- lmer(formula = focusfuture_day_prop ~
 RADS_total
 + (1 |id_participant),
 data = df_aim1_all_language,
 na.action=na.exclude)
summary(model.S1f.1)

## Linear mixed model fit by REML. t-tests use Satterthwaite's method [
## lmerModLmerTest]
## Formula: focusfuture_day_prop ~ RADS_total + (1 | id_participant)
## Data: df_aim1_all_language
##
## REML criterion at convergence: 6920.1
##
## Scaled residuals:
## Min 1Q Median 3Q Max
## -1.9146 -0.6200 -0.0865 0.4261 8.0308
##
## Random effects:
## Groups Name Variance Std.Dev.
## id_participant (Intercept) 0.1243 0.3526
## Residual 1.2267 1.1076
## Number of obs: 2251, groups: id_participant, 40
##
## Fixed effects:
## Estimate Std. Error df t value Pr(>|t|)
## (Intercept) 2.041092 0.250683 26.441813 8.142 1.12e-08 ***
## RADS_total -0.007745 0.003367 26.082358 -2.300 0.0297 *
## ---
## Signif. codes: 0 '***' 0.001 '**' 0.01 '*' 0.05 '.' 0.1 ' ' 1
##
## Correlation of Fixed Effects:
## (Intr)
## RADS_total -0.964

standardize_parameters(model.S1f.1)

## # Standardization method: refit
##
## Parameter | Std. Coef. | 95% CI
## -----------------------------------------
## (Intercept) | 0.06 | [-0.06, 0.17]
## RADS_total | -0.13 | [-0.23, -0.02]

### S1f.2 Age

#Future focus words (daily proportion of total words)
 model.S1f.2 <- lmer(formula = focusfuture_day_prop ~
 RADS_total
 + Age.at.V2
 + (1 |id_participant),
 data = df_aim1_all_language,
 na.action=na.exclude)
summary(model.S1f.2)

## Linear mixed model fit by REML. t-tests use Satterthwaite's method [
## lmerModLmerTest]
## Formula: focusfuture_day_prop ~ RADS_total + Age.at.V2 + (1 | id_participant)
## Data: df_aim1_all_language
##
## REML criterion at convergence: 6920
##
## Scaled residuals:
## Min 1Q Median 3Q Max
## -1.9201 -0.6229 -0.0883 0.4221 8.0397
##
## Random effects:
## Groups Name Variance Std.Dev.
## id_participant (Intercept) 0.1178 0.3433
## Residual 1.2258 1.1071
## Number of obs: 2251, groups: id_participant, 40
##
## Fixed effects:
## Estimate Std. Error df t value Pr(>|t|)
## (Intercept) 0.127678 0.981126 26.570387 0.130 0.8974
## RADS_total -0.007046 0.003315 25.918568 -2.126 0.0432 *
## Age.at.V2 0.114874 0.057028 26.698748 2.014 0.0542 .
## ---
## Signif. codes: 0 '***' 0.001 '**' 0.01 '*' 0.05 '.' 0.1 ' ' 1
##
## Correlation of Fixed Effects:
## (Intr) RADS_t
## RADS_total -0.342
## Age.at.V2 -0.968 0.106

standardize_parameters(model.S1f.2)

## # Standardization method: refit
##
## Parameter | Std. Coef. | 95% CI
## -----------------------------------------
## (Intercept) | 0.03 | [-0.08, 0.15]
## RADS_total | -0.11 | [-0.22, -0.01]
## Age.at.V2 | 0.12 | [ 0.00, 0.24]

#Model comparison with LRT
anova(model.S1f.1, model.S1f.2)

## refitting model(s) with ML (instead of REML)

## Data: df_aim1_all_language
## Models:
## model.S1f.1: focusfuture_day_prop ~ RADS_total + (1 | id_participant)
## model.S1f.2: focusfuture_day_prop ~ RADS_total + Age.at.V2 + (1 | id_participant)
## npar AIC BIC logLik deviance Chisq Df Pr(>Chisq)
## model.S1f.1 4 6914.9 6937.8 -3453.5 6906.9
## model.S1f.2 5 6912.7 6941.3 -3451.4 6902.7 4.2092 1 0.0402 *
## ---
## Signif. codes: 0 '***' 0.001 '**' 0.01 '*' 0.05 '.' 0.1 ' ' 1

### S1f.3 Sex

#Future focus words (daily proportion of total words)
 model.S1f.3 <- lmer(formula = focusfuture_day_prop ~
 RADS_total
 + Sex
 + (1 |id_participant),
 data = df_aim1_all_language,
 na.action=na.exclude)
summary(model.S1f.3)

## Linear mixed model fit by REML. t-tests use Satterthwaite's method [
## lmerModLmerTest]
## Formula: focusfuture_day_prop ~ RADS_total + Sex + (1 | id_participant)
## Data: df_aim1_all_language
##
## REML criterion at convergence: 6920.7
##
## Scaled residuals:
## Min 1Q Median 3Q Max
## -1.8976 -0.6254 -0.0870 0.4266 8.0204
##
## Random effects:
## Groups Name Variance Std.Dev.
## id_participant (Intercept) 0.1271 0.3566
## Residual 1.2261 1.1073
## Number of obs: 2251, groups: id_participant, 40
##
## Fixed effects:
## Estimate Std. Error df t value Pr(>|t|)
## (Intercept) 1.857425 0.295809 24.849591 6.279 1.47e-06 ***
## RADS_total -0.009077 0.003575 27.038368 -2.539 0.0172 *
## Sex 0.173949 0.145327 27.044400 1.197 0.2417
## ---
## Signif. codes: 0 '***' 0.001 '**' 0.01 '*' 0.05 '.' 0.1 ' ' 1
##
## Correlation of Fixed Effects:
## (Intr) RADS_t
## RADS_total -0.621
## Sex -0.519 -0.312

standardize_parameters(model.S1f.3)

## # Standardization method: refit
##
## Parameter | Std. Coef. | 95% CI
## -----------------------------------------
## (Intercept) | 0.05 | [-0.07, 0.16]
## RADS_total | -0.15 | [-0.26, -0.03]
## Sex | 0.07 | [-0.05, 0.20]

#Model comparison with LRT
anova(model.S1f.1, model.S1f.3)

## refitting model(s) with ML (instead of REML)

## Data: df_aim1_all_language
## Models:
## model.S1f.1: focusfuture_day_prop ~ RADS_total + (1 | id_participant)
## model.S1f.3: focusfuture_day_prop ~ RADS_total + Sex + (1 | id_participant)
## npar AIC BIC logLik deviance Chisq Df Pr(>Chisq)
## model.S1f.1 4 6914.9 6937.8 -3453.5 6906.9
## model.S1f.3 5 6915.4 6944.0 -3452.7 6905.4 1.4841 1 0.2231

### S1f.4 Gender

#Future focus words (daily proportion of total words)
 model.S1f.4 <- lmer(formula = focusfuture_day_prop ~
 RADS_total
 + Gender
 + (1 |id_participant),
 data = df_aim1_all_language,
 na.action=na.exclude)
summary(model.S1f.4)

## Linear mixed model fit by REML. t-tests use Satterthwaite's method [
## lmerModLmerTest]
## Formula: focusfuture_day_prop ~ RADS_total + Gender + (1 | id_participant)
## Data: df_aim1_all_language
##
## REML criterion at convergence: 6922.7
##
## Scaled residuals:
## Min 1Q Median 3Q Max
## -1.9204 -0.6198 -0.0869 0.4264 8.0323
##
## Random effects:
## Groups Name Variance Std.Dev.
## id_participant (Intercept) 0.131 0.3619
## Residual 1.226 1.1075
## Number of obs: 2251, groups: id_participant, 40
##
## Fixed effects:
## Estimate Std. Error df t value Pr(>|t|)
## (Intercept) 2.035945 0.267789 24.557430 7.603 6.6e-08 ***
## RADS_total -0.007848 0.003821 26.556773 -2.054 0.0499 *
## Gender 0.007228 0.110897 24.272271 0.065 0.9486
## ---
## Signif. codes: 0 '***' 0.001 '**' 0.01 '*' 0.05 '.' 0.1 ' ' 1
##
## Correlation of Fixed Effects:
## (Intr) RADS_t
## RADS_total -0.700
## Gender -0.295 -0.437

standardize_parameters(model.S1f.4)

## # Standardization method: refit
##
## Parameter | Std. Coef. | 95% CI
## -----------------------------------------
## (Intercept) | 0.06 | [-0.06, 0.17]
## RADS_total | -0.13 | [-0.25, -0.01]
## Gender | 5.05e-03 | [-0.15, 0.16]

#Model comparison with LRT
anova(model.S1f.1, model.S1f.4)

## refitting model(s) with ML (instead of REML)

## Data: df_aim1_all_language
## Models:
## model.S1f.1: focusfuture_day_prop ~ RADS_total + (1 | id_participant)
## model.S1f.4: focusfuture_day_prop ~ RADS_total + Gender + (1 | id_participant)
## npar AIC BIC logLik deviance Chisq Df Pr(>Chisq)
## model.S1f.1 4 6914.9 6937.8 -3453.5 6906.9
## model.S1f.4 5 6916.9 6945.5 -3453.5 6906.9 4e-04 1 0.9846

### S1f.5 COVID

#Future focus words (daily proportion of total words)
 model.S1f.5 <- lmer(formula = focusfuture_day_prop ~
 RADS_total
 + Collected_During_Covid
 + (1 |id_participant),
 data = df_aim1_all_language,
 na.action=na.exclude)
summary(model.S1f.5)

## Linear mixed model fit by REML. t-tests use Satterthwaite's method [
## lmerModLmerTest]
## Formula: focusfuture_day_prop ~ RADS_total + Collected_During_Covid +
## (1 | id_participant)
## Data: df_aim1_all_language
##
## REML criterion at convergence: 6921.6
##
## Scaled residuals:
## Min 1Q Median 3Q Max
## -1.9235 -0.6205 -0.0894 0.4263 8.0318
##
## Random effects:
## Groups Name Variance Std.Dev.
## id_participant (Intercept) 0.1298 0.3602
## Residual 1.2264 1.1074
## Number of obs: 2251, groups: id_participant, 40
##
## Fixed effects:
## Estimate Std. Error df t value Pr(>|t|)
## (Intercept) 2.006519 0.261918 26.147817 7.661 3.8e-08 ***
## RADS_total -0.006864 0.003747 25.874058 -1.832 0.0785 .
## Collected_During_Covid -0.093350 0.161798 26.639894 -0.577 0.5688
## ---
## Signif. codes: 0 '***' 0.001 '**' 0.01 '*' 0.05 '.' 0.1 ' ' 1
##
## Correlation of Fixed Effects:
## (Intr) RADS_t
## RADS_total -0.950
## Cllctd_Dr_C 0.229 -0.406

standardize_parameters(model.S1f.5)

## # Standardization method: refit
##
## Parameter | Std. Coef. | 95% CI
## ---------------------------------------------------
## (Intercept) | 0.06 | [-0.06, 0.17]
## RADS_total | -0.11 | [-0.23, 0.01]
## Collected_During_Covid | -0.04 | [-0.16, 0.09]

#Model comparison with LRT
anova(model.S1f.1, model.S1f.5)

## refitting model(s) with ML (instead of REML)

## Data: df_aim1_all_language
## Models:
## model.S1f.1: focusfuture_day_prop ~ RADS_total + (1 | id_participant)
## model.S1f.5: focusfuture_day_prop ~ RADS_total + Collected_During_Covid + (1 | id_participant)
## npar AIC BIC logLik deviance Chisq Df Pr(>Chisq)
## model.S1f.1 4 6914.9 6937.8 -3453.5 6906.9
## model.S1f.5 5 6916.6 6945.2 -3453.3 6906.6 0.3429 1 0.5582

### S1f.6 Number of Messages

#Future focus words (daily proportion of total words)
 model.S1f.6 <- lmer(formula = focusfuture_day_prop ~
 RADS_total
 + n_msg_day
 + (1 |id_participant),
 data = df_aim1_all_language,
 na.action=na.exclude)
summary(model.S1f.6)

## Linear mixed model fit by REML. t-tests use Satterthwaite's method [
## lmerModLmerTest]
## Formula: focusfuture_day_prop ~ RADS_total + n_msg_day + (1 | id_participant)
## Data: df_aim1_all_language
##
## REML criterion at convergence: 6934.8
##
## Scaled residuals:
## Min 1Q Median 3Q Max
## -1.9117 -0.6185 -0.0949 0.4257 8.0395
##
## Random effects:
## Groups Name Variance Std.Dev.
## id_participant (Intercept) 0.1232 0.351
## Residual 1.2268 1.108
## Number of obs: 2251, groups: id_participant, 40
##
## Fixed effects:
## Estimate Std. Error df t value Pr(>|t|)
## (Intercept) 2.045e+00 2.498e-01 2.616e+01 8.185 1.1e-08 ***
## RADS_total -8.056e-03 3.369e-03 2.625e+01 -2.391 0.0242 *
## n_msg_day 1.532e-04 1.527e-04 1.495e+03 1.003 0.3161
## ---
## Signif. codes: 0 '***' 0.001 '**' 0.01 '*' 0.05 '.' 0.1 ' ' 1
##
## Correlation of Fixed Effects:
## (Intr) RADS_t
## RADS_total -0.961
## n_msg_day 0.015 -0.092

standardize_parameters(model.S1f.6)

## # Standardization method: refit
##
## Parameter | Std. Coef. | 95% CI
## -----------------------------------------
## (Intercept) | 0.06 | [-0.06, 0.17]
## RADS_total | -0.13 | [-0.24, -0.02]
## n_msg_day | 0.03 | [-0.03, 0.08]

#Model comparison with LRT
anova(model.S1f.1, model.S1f.6)

## refitting model(s) with ML (instead of REML)

## Data: df_aim1_all_language
## Models:
## model.S1f.1: focusfuture_day_prop ~ RADS_total + (1 | id_participant)
## model.S1f.6: focusfuture_day_prop ~ RADS_total + n_msg_day + (1 | id_participant)
## npar AIC BIC logLik deviance Chisq Df Pr(>Chisq)
## model.S1f.1 4 6914.9 6937.8 -3453.5 6906.9
## model.S1f.6 5 6915.9 6944.5 -3452.9 6905.9 1.0271 1 0.3108

### S1f.7 Days between V2 and EARS start

#Future focus words (daily proportion of total words)
 model.S1f.7 <- lmer(formula = focusfuture_day_prop ~
 RADS_total
 + days_since_V2
 + (1 |id_participant),
 data = df_aim1_all_language,
 na.action=na.exclude)
summary(model.S1f.7)

## Linear mixed model fit by REML. t-tests use Satterthwaite's method [
## lmerModLmerTest]
## Formula:
## focusfuture_day_prop ~ RADS_total + days_since_V2 + (1 | id_participant)
## Data: df_aim1_all_language
##
## REML criterion at convergence: 6934.2
##
## Scaled residuals:
## Min 1Q Median 3Q Max
## -1.9149 -0.6181 -0.0891 0.4251 8.0285
##
## Random effects:
## Groups Name Variance Std.Dev.
## id_participant (Intercept) 0.1277 0.3574
## Residual 1.2265 1.1075
## Number of obs: 2251, groups: id_participant, 40
##
## Fixed effects:
## Estimate Std. Error df t value Pr(>|t|)
## (Intercept) 1.9789526 0.2669490 25.8576021 7.413 7.41e-08 ***
## RADS_total -0.0077418 0.0034029 25.4741232 -2.275 0.0316 *
## days_since_V2 0.0001960 0.0002653 25.6777246 0.739 0.4666
## ---
## Signif. codes: 0 '***' 0.001 '**' 0.01 '*' 0.05 '.' 0.1 ' ' 1
##
## Correlation of Fixed Effects:
## (Intr) RADS_t
## RADS_total -0.915
## days_snc_V2 -0.315 0.000

standardize_parameters(model.S1f.7)

## # Standardization method: refit
##
## Parameter | Std. Coef. | 95% CI
## -------------------------------------------
## (Intercept) | 0.06 | [-0.06, 0.17]
## RADS_total | -0.13 | [-0.23, -0.02]
## days_since_V2 | 0.04 | [-0.07, 0.16]

#Model comparison with LRT
anova(model.S1f.1, model.S1f.7)

## refitting model(s) with ML (instead of REML)

## Data: df_aim1_all_language
## Models:
## model.S1f.1: focusfuture_day_prop ~ RADS_total + (1 | id_participant)
## model.S1f.7: focusfuture_day_prop ~ RADS_total + days_since_V2 + (1 | id_participant)
## npar AIC BIC logLik deviance Chisq Df Pr(>Chisq)
## model.S1f.1 4 6914.9 6937.8 -3453.5 6906.9
## model.S1f.7 5 6916.3 6944.9 -3453.2 6906.3 0.5937 1 0.441

### S1f.8 Days since COVID stay-at-home order

#Future focus words (daily proportion of total words)
 model.S1f.8 <- lmer(formula = focusfuture_day_prop ~
 RADS_total
 + days_since_stay_at_home
 + (1 |id_participant),
 data = df_aim1_all_language,
 na.action=na.exclude)
summary(model.S1f.8)

## Linear mixed model fit by REML. t-tests use Satterthwaite's method [
## lmerModLmerTest]
## Formula: focusfuture_day_prop ~ RADS_total + days_since_stay_at_home +
## (1 | id_participant)
## Data: df_aim1_all_language
##
## REML criterion at convergence: 6933.4
##
## Scaled residuals:
## Min 1Q Median 3Q Max
## -1.9235 -0.6196 -0.0890 0.4260 8.0320
##
## Random effects:
## Groups Name Variance Std.Dev.
## id_participant (Intercept) 0.1297 0.3601
## Residual 1.2265 1.1075
## Number of obs: 2251, groups: id_participant, 40
##
## Fixed effects:
## Estimate Std. Error df t value Pr(>|t|)
## (Intercept) 2.0220542 0.2577308 25.8633986 7.846 2.64e-08 ***
## RADS_total -0.0069363 0.0037807 25.4026280 -1.835 0.0783 .
## days_since_stay_at_home -0.0002296 0.0004577 30.1508701 -0.502 0.6195
## ---
## Signif. codes: 0 '***' 0.001 '**' 0.01 '*' 0.05 '.' 0.1 ' ' 1
##
## Correlation of Fixed Effects:
## (Intr) RADS_t
## RADS_total -0.926
## dys_snc_s__ 0.147 -0.424

standardize_parameters(model.S1f.8)

## # Standardization method: refit
##
## Parameter | Std. Coef. | 95% CI
## ----------------------------------------------------
## (Intercept) | 0.06 | [-0.06, 0.18]
## RADS_total | -0.11 | [-0.23, 0.01]
## days_since_stay_at_home | -0.03 | [-0.15, 0.09]

#Model comparison with LRT
anova(model.S1f.1, model.S1f.8)

## refitting model(s) with ML (instead of REML)

## Data: df_aim1_all_language
## Models:
## model.S1f.1: focusfuture_day_prop ~ RADS_total + (1 | id_participant)
## model.S1f.8: focusfuture_day_prop ~ RADS_total + days_since_stay_at_home + (1 | id_participant)
## npar AIC BIC logLik deviance Chisq Df Pr(>Chisq)
## model.S1f.1 4 6914.9 6937.8 -3453.5 6906.9
## model.S1f.8 5 6916.7 6945.3 -3453.3 6906.7 0.2562 1 0.6127

### S1f.9 Medication Status

#Future focus words (daily proportion of total words)
 model.S1f.9 <- lmer(formula = focusfuture_day_prop ~
 RADS_total
 + Current_Med_Depression
 + (1 |id_participant),
 data = df_aim1_all_language,
 na.action=na.exclude)
summary(model.S1f.9)

## Linear mixed model fit by REML. t-tests use Satterthwaite's method [
## lmerModLmerTest]
## Formula: focusfuture_day_prop ~ RADS_total + Current_Med_Depression +
## (1 | id_participant)
## Data: df_aim1_all_language
##
## REML criterion at convergence: 6917.9
##
## Scaled residuals:
## Min 1Q Median 3Q Max
## -1.9433 -0.6239 -0.0842 0.4253 8.0356
##
## Random effects:
## Groups Name Variance Std.Dev.
## id_participant (Intercept) 0.1168 0.3417
## Residual 1.2259 1.1072
## Number of obs: 2251, groups: id_participant, 40
##
## Fixed effects:
## Estimate Std. Error df t value Pr(>|t|)
## (Intercept) 2.099087 0.246305 27.355111 8.522 3.5e-09 ***
## RADS_total -0.009580 0.003409 27.652653 -2.810 0.00898 **
## Current_Med_Depression 0.324797 0.161974 25.771952 2.005 0.05555 .
## ---
## Signif. codes: 0 '***' 0.001 '**' 0.01 '*' 0.05 '.' 0.1 ' ' 1
##
## Correlation of Fixed Effects:
## (Intr) RADS_t
## RADS_total -0.954
## Crrnt_Md_Dp 0.117 -0.268

standardize_parameters(model.S1f.9)

## # Standardization method: refit
##
## Parameter | Std. Coef. | 95% CI
## ----------------------------------------------------
## (Intercept) | 0.09 | [-0.03, 0.21]
## RADS_total | -0.16 | [-0.26, -0.05]
## Current_Med_Depression | 0.14 | [ 0.00, 0.27]

#Model comparison with LRT
anova(model.S1f.1, model.S1f.9)

## refitting model(s) with ML (instead of REML)

## Data: df_aim1_all_language
## Models:
## model.S1f.1: focusfuture_day_prop ~ RADS_total + (1 | id_participant)
## model.S1f.9: focusfuture_day_prop ~ RADS_total + Current_Med_Depression + (1 | id_participant)
## npar AIC BIC logLik deviance Chisq Df Pr(>Chisq)
## model.S1f.1 4 6914.9 6937.8 -3453.5 6906.9
## model.S1f.9 5 6912.7 6941.3 -3451.4 6902.7 4.1766 1 0.04099 *
## ---
## Signif. codes: 0 '***' 0.001 '**' 0.01 '*' 0.05 '.' 0.1 ' ' 1

# 2. AIM 2 - Which intrinsic connectivity networks are associated with depression-related linguistic features of smartphone use?

## S2a. First-person pronouns (controlling for depressive symptoms)

### S2a.1a Within-Network aDMN

modelS2a.1a <- lmer(formula = i_day_prop ~
 aDMN_Z + RADS_total
 + (1 |id_participant) ,
 data = df_aim1_all_language,
 na.action=na.exclude)
summary(modelS2a.1a)

## Linear mixed model fit by REML. t-tests use Satterthwaite's method [
## lmerModLmerTest]
## Formula: i_day_prop ~ aDMN_Z + RADS_total + (1 | id_participant)
## Data: df_aim1_all_language
##
## REML criterion at convergence: 10984.5
##
## Scaled residuals:
## Min 1Q Median 3Q Max
## -3.1295 -0.5621 -0.0136 0.5006 7.5236
##
## Random effects:
## Groups Name Variance Std.Dev.
## id_participant (Intercept) 1.817 1.348
## Residual 11.416 3.379
## Number of obs: 2070, groups: id_participant, 34
##
## Fixed effects:
## Estimate Std. Error df t value Pr(>|t|)
## (Intercept) 2.10012 1.70117 34.41917 1.235 0.22537
## aDMN_Z 1.69478 0.78764 36.97581 2.152 0.03802 *
## RADS_total 0.04799 0.01353 28.89800 3.547 0.00135 **
## ---
## Signif. codes: 0 '***' 0.001 '**' 0.01 '*' 0.05 '.' 0.1 ' ' 1
##
## Correlation of Fixed Effects:
## (Intr) aDMN_Z
## aDMN_Z -0.808
## RADS_total -0.712 0.190

standardize_parameters(modelS2a.1a)

## # Standardization method: refit
##
## Parameter | Std. Coef. | 95% CI
## ----------------------------------------
## (Intercept) | 0.01 | [-0.12, 0.15]
## aDMN_Z | 0.12 | [ 0.01, 0.23]
## RADS_total | 0.24 | [ 0.11, 0.38]

### S2a.1b Age

modelS2a.1b <- lmer(formula = i_day_prop ~
 aDMN_Z + RADS_total
 + Age.at.V2
 + (1 |id_participant) ,
 data = df_aim1_all_language,
 na.action=na.exclude)
summary(modelS2a.1b)

## Linear mixed model fit by REML. t-tests use Satterthwaite's method [
## lmerModLmerTest]
## Formula: i_day_prop ~ aDMN_Z + RADS_total + Age.at.V2 + (1 | id_participant)
## Data: df_aim1_all_language
##
## REML criterion at convergence: 10985.6
##
## Scaled residuals:
## Min 1Q Median 3Q Max
## -3.1333 -0.5625 -0.0129 0.5003 7.5223
##
## Random effects:
## Groups Name Variance Std.Dev.
## id_participant (Intercept) 1.893 1.376
## Residual 11.416 3.379
## Number of obs: 2070, groups: id_participant, 34
##
## Fixed effects:
## Estimate Std. Error df t value Pr(>|t|)
## (Intercept) 2.18377 4.33793 31.80557 0.503 0.61814
## aDMN_Z 1.69758 0.80173 36.39587 2.117 0.04113 *
## RADS_total 0.04802 0.01386 28.06132 3.465 0.00172 **
## Age.at.V2 -0.00567 0.23147 28.38702 -0.024 0.98063
## ---
## Signif. codes: 0 '***' 0.001 '**' 0.01 '*' 0.05 '.' 0.1 ' ' 1
##
## Correlation of Fixed Effects:
## (Intr) aDMN_Z RADS_t
## aDMN_Z -0.385
## RADS_total -0.388 0.195
## Age.at.V2 -0.917 0.070 0.116

standardize_parameters(modelS2a.1b)

## # Standardization method: refit
##
## Parameter | Std. Coef. | 95% CI
## ----------------------------------------
## (Intercept) | 0.01 | [-0.13, 0.16]
## aDMN_Z | 0.12 | [ 0.01, 0.23]
## RADS_total | 0.24 | [ 0.11, 0.38]
## Age.at.V2 | -1.90e-03 | [-0.15, 0.15]

#Model comparison with LRT
anova(modelS2a.1a, modelS2a.1b)

## refitting model(s) with ML (instead of REML)

## Data: df_aim1_all_language
## Models:
## modelS2a.1a: i_day_prop ~ aDMN_Z + RADS_total + (1 | id_participant)
## modelS2a.1b: i_day_prop ~ aDMN_Z + RADS_total + Age.at.V2 + (1 | id_participant)
## npar AIC BIC logLik deviance Chisq Df Pr(>Chisq)
## modelS2a.1a 5 10988 11016 -5489 10978
## modelS2a.1b 6 10990 11024 -5489 10978 0.0025 1 0.96

### S2a.1c Sex

modelS2a.1c <- lmer(formula = i_day_prop ~
 aDMN_Z + RADS_total
 + Sex
 + (1 |id_participant) ,
 data = df_aim1_all_language,
 na.action=na.exclude)
summary(modelS2a.1c)

## Linear mixed model fit by REML. t-tests use Satterthwaite's method [
## lmerModLmerTest]
## Formula: i_day_prop ~ aDMN_Z + RADS_total + Sex + (1 | id_participant)
## Data: df_aim1_all_language
##
## REML criterion at convergence: 10983.6
##
## Scaled residuals:
## Min 1Q Median 3Q Max
## -3.1371 -0.5648 -0.0125 0.5015 7.5237
##
## Random effects:
## Groups Name Variance Std.Dev.
## id_participant (Intercept) 1.887 1.374
## Residual 11.415 3.379
## Number of obs: 2070, groups: id_participant, 34
##
## Fixed effects:
## Estimate Std. Error df t value Pr(>|t|)
## (Intercept) 1.88251 1.78892 34.45418 1.052 0.29998
## aDMN_Z 1.66854 0.80186 35.03876 2.081 0.04482 *
## RADS_total 0.04589 0.01462 28.04665 3.140 0.00396 **
## Sex 0.25302 0.57949 27.23571 0.437 0.66583
## ---
## Signif. codes: 0 '***' 0.001 '**' 0.01 '*' 0.05 '.' 0.1 ' ' 1
##
## Correlation of Fixed Effects:
## (Intr) aDMN_Z RADS_t
## aDMN_Z -0.754
## RADS_total -0.557 0.206
## Sex -0.262 -0.086 -0.339

standardize_parameters(modelS2a.1c)

## # Standardization method: refit
##
## Parameter | Std. Coef. | 95% CI
## ----------------------------------------
## (Intercept) | 8.73e-03 | [-0.13, 0.15]
## aDMN_Z | 0.12 | [ 0.01, 0.23]
## RADS_total | 0.23 | [ 0.09, 0.38]
## Sex | 0.03 | [-0.12, 0.19]

#Model comparison with LRT
anova(modelS2a.1a, modelS2a.1c)

## refitting model(s) with ML (instead of REML)

## Data: df_aim1_all_language
## Models:
## modelS2a.1a: i_day_prop ~ aDMN_Z + RADS_total + (1 | id_participant)
## modelS2a.1c: i_day_prop ~ aDMN_Z + RADS_total + Sex + (1 | id_participant)
## npar AIC BIC logLik deviance Chisq Df Pr(>Chisq)
## modelS2a.1a 5 10988 11016 -5489.0 10978
## modelS2a.1c 6 10990 11024 -5488.9 10978 0.2027 1 0.6525

### S2a.1d Motion

modelS2a.1d <- lmer(formula = i_day_prop ~
 aDMN_Z + RADS_total
 + motion
 + (1 |id_participant) ,
 data = df_aim1_all_language,
 na.action=na.exclude)
summary(modelS2a.1d)

## Linear mixed model fit by REML. t-tests use Satterthwaite's method [
## lmerModLmerTest]
## Formula: i_day_prop ~ aDMN_Z + RADS_total + motion + (1 | id_participant)
## Data: df_aim1_all_language
##
## REML criterion at convergence: 10977.5
##
## Scaled residuals:
## Min 1Q Median 3Q Max
## -3.1320 -0.5635 -0.0158 0.4999 7.5149
##
## Random effects:
## Groups Name Variance Std.Dev.
## id_participant (Intercept) 1.832 1.354
## Residual 11.416 3.379
## Number of obs: 2070, groups: id_participant, 34
##
## Fixed effects:
## Estimate Std. Error df t value Pr(>|t|)
## (Intercept) 1.03512 2.16103 30.98006 0.479 0.63531
## aDMN_Z 1.80128 0.80105 35.03562 2.249 0.03093 *
## RADS_total 0.05086 0.01404 28.21477 3.623 0.00114 **
## motion 7.70574 9.61662 27.04790 0.801 0.42994
## ---
## Signif. codes: 0 '***' 0.001 '**' 0.01 '*' 0.05 '.' 0.1 ' ' 1
##
## Correlation of Fixed Effects:
## (Intr) aDMN_Z RADS_t
## aDMN_Z -0.730
## RADS_total -0.700 0.223
## motion -0.613 0.165 0.254

standardize_parameters(modelS2a.1d)

## # Standardization method: refit
##
## Parameter | Std. Coef. | 95% CI
## ----------------------------------------
## (Intercept) | 0.03 | [-0.12, 0.17]
## aDMN_Z | 0.13 | [ 0.02, 0.24]
## RADS_total | 0.26 | [ 0.12, 0.40]
## motion | 0.06 | [-0.09, 0.22]

### S2a.1e Days between V2 and EARS start

modelS2a.1e <- lmer(formula = i_day_prop ~
 aDMN_Z + RADS_total
 + days_since_V2
 + (1 |id_participant) ,
 data = df_aim1_all_language,
 na.action=na.exclude)
summary(modelS2a.1e)

## Linear mixed model fit by REML. t-tests use Satterthwaite's method [
## lmerModLmerTest]
## Formula:
## i_day_prop ~ aDMN_Z + RADS_total + days_since_V2 + (1 | id_participant)
## Data: df_aim1_all_language
##
## REML criterion at convergence: 10996.4
##
## Scaled residuals:
## Min 1Q Median 3Q Max
## -3.1346 -0.5624 -0.0135 0.5002 7.5223
##
## Random effects:
## Groups Name Variance Std.Dev.
## id_participant (Intercept) 1.895 1.377
## Residual 11.416 3.379
## Number of obs: 2070, groups: id_participant, 34
##
## Fixed effects:
## Estimate Std. Error df t value Pr(>|t|)
## (Intercept) 2.081e+00 1.731e+00 3.308e+01 1.202 0.23779
## aDMN_Z 1.713e+00 8.270e-01 3.492e+01 2.071 0.04577 *
## RADS_total 4.813e-02 1.381e-02 2.796e+01 3.485 0.00164 **
## days_since_V2 -7.128e-05 1.058e-03 2.659e+01 -0.067 0.94679
## ---
## Signif. codes: 0 '***' 0.001 '**' 0.01 '*' 0.05 '.' 0.1 ' ' 1
##
## Correlation of Fixed Effects:
## (Intr) aDMN_Z RADS_t
## aDMN_Z -0.791
## RADS_total -0.712 0.201
## days_snc_V2 0.044 -0.253 -0.074

standardize_parameters(modelS2a.1e)

## # Standardization method: refit
##
## Parameter | Std. Coef. | 95% CI
## ------------------------------------------
## (Intercept) | 0.01 | [-0.13, 0.16]
## aDMN_Z | 0.12 | [ 0.01, 0.24]
## RADS_total | 0.24 | [ 0.11, 0.38]
## days_since_V2 | -5.11e-03 | [-0.15, 0.14]

### S2a.2a Within-Network leftCEN_Z

modelS2a.2a <- lmer(formula = i_day_prop ~
 leftCEN_Z + RADS_total
 + (1 |id_participant) ,
 data = df_aim1_all_language,
 na.action=na.exclude)
summary(modelS2a.2a)

## Linear mixed model fit by REML. t-tests use Satterthwaite's method [
## lmerModLmerTest]
## Formula: i_day_prop ~ leftCEN_Z + RADS_total + (1 | id_participant)
## Data: df_aim1_all_language
##
## REML criterion at convergence: 10982.9
##
## Scaled residuals:
## Min 1Q Median 3Q Max
## -3.0881 -0.5685 -0.0138 0.5009 7.5146
##
## Random effects:
## Groups Name Variance Std.Dev.
## id_participant (Intercept) 1.731 1.316
## Residual 11.415 3.379
## Number of obs: 2070, groups: id_participant, 34
##
## Fixed effects:
## Estimate Std. Error df t value Pr(>|t|)
## (Intercept) -0.57178 2.49014 37.69776 -0.230 0.819631
## leftCEN_Z 2.23640 0.90764 35.05626 2.464 0.018789 *
## RADS_total 0.06149 0.01515 33.46429 4.059 0.000279 ***
## ---
## Signif. codes: 0 '***' 0.001 '**' 0.01 '*' 0.05 '.' 0.1 ' ' 1
##
## Correlation of Fixed Effects:
## (Intr) lCEN_Z
## leftCEN_Z -0.919
## RADS_total -0.797 0.512

standardize_parameters(modelS2a.2a)

## # Standardization method: refit
##
## Parameter | Std. Coef. | 95% CI
## ----------------------------------------
## (Intercept) | 0.01 | [-0.13, 0.15]
## leftCEN_Z | 0.19 | [ 0.04, 0.34]
## RADS_total | 0.31 | [ 0.16, 0.46]

### S2a.2b Age

modelS2a.2b <- lmer(formula = i_day_prop ~
 leftCEN_Z + RADS_total
 + Age.at.V2
 + (1 |id_participant) ,
 data = df_aim1_all_language,
 na.action=na.exclude)
summary(modelS2a.2b)

## Linear mixed model fit by REML. t-tests use Satterthwaite's method [
## lmerModLmerTest]
## Formula: i_day_prop ~ leftCEN_Z + RADS_total + Age.at.V2 + (1 | id_participant)
## Data: df_aim1_all_language
##
## REML criterion at convergence: 10983.9
##
## Scaled residuals:
## Min 1Q Median 3Q Max
## -3.0883 -0.5693 -0.0134 0.4997 7.5116
##
## Random effects:
## Groups Name Variance Std.Dev.
## id_participant (Intercept) 1.78 1.334
## Residual 11.42 3.379
## Number of obs: 2070, groups: id_participant, 34
##
## Fixed effects:
## Estimate Std. Error df t value Pr(>|t|)
## (Intercept) 0.93632 4.36798 31.90070 0.214 0.831630
## leftCEN_Z 2.27671 0.92155 33.01610 2.471 0.018828 *
## RADS_total 0.06124 0.01533 32.19926 3.995 0.000353 ***
## Age.at.V2 -0.09609 0.22599 27.36955 -0.425 0.674023
## ---
## Signif. codes: 0 '***' 0.001 '**' 0.01 '*' 0.05 '.' 0.1 ' ' 1
##
## Correlation of Fixed Effects:
## (Intr) lCEN_Z RADS_t
## leftCEN_Z -0.450
## RADS_total -0.493 0.504
## Age.at.V2 -0.817 -0.094 0.042

standardize_parameters(modelS2a.2b)

## # Standardization method: refit
##
## Parameter | Std. Coef. | 95% CI
## ----------------------------------------
## (Intercept) | 0.02 | [-0.12, 0.16]
## leftCEN_Z | 0.20 | [ 0.04, 0.35]
## RADS_total | 0.31 | [ 0.16, 0.46]
## Age.at.V2 | -0.03 | [-0.18, 0.12]

#Model comparison with LRT
anova(modelS2a.2a, modelS2a.2b)

## refitting model(s) with ML (instead of REML)

## Data: df_aim1_all_language
## Models:
## modelS2a.2a: i_day_prop ~ leftCEN_Z + RADS_total + (1 | id_participant)
## modelS2a.2b: i_day_prop ~ leftCEN_Z + RADS_total + Age.at.V2 + (1 | id_participant)
## npar AIC BIC logLik deviance Chisq Df Pr(>Chisq)
## modelS2a.2a 5 10987 11015 -5488.3 10977
## modelS2a.2b 6 10988 11022 -5488.2 10976 0.2264 1 0.6342

### S2a.2c Sex

modelS2a.2c <- lmer(formula = i_day_prop ~
 leftCEN_Z + RADS_total
 + Sex
 + (1 |id_participant) ,
 data = df_aim1_all_language,
 na.action=na.exclude)
summary(modelS2a.2c)

## Linear mixed model fit by REML. t-tests use Satterthwaite's method [
## lmerModLmerTest]
## Formula: i_day_prop ~ leftCEN_Z + RADS_total + Sex + (1 | id_participant)
## Data: df_aim1_all_language
##
## REML criterion at convergence: 10982.2
##
## Scaled residuals:
## Min 1Q Median 3Q Max
## -3.0956 -0.5689 -0.0141 0.5012 7.5143
##
## Random effects:
## Groups Name Variance Std.Dev.
## id_participant (Intercept) 1.809 1.345
## Residual 11.415 3.379
## Number of obs: 2070, groups: id_participant, 34
##
## Fixed effects:
## Estimate Std. Error df t value Pr(>|t|)
## (Intercept) -0.60582 2.53398 36.08873 -0.239 0.812397
## leftCEN_Z 2.21460 0.94119 34.06758 2.353 0.024536 *
## RADS_total 0.06061 0.01665 32.96773 3.641 0.000922 ***
## Sex 0.08548 0.57813 27.97362 0.148 0.883519
## ---
## Signif. codes: 0 '***' 0.001 '**' 0.01 '*' 0.05 '.' 0.1 ' ' 1
##
## Correlation of Fixed Effects:
## (Intr) lCEN_Z RADS_t
## leftCEN_Z -0.890
## RADS_total -0.718 0.537
## Sex -0.049 -0.195 -0.378

standardize_parameters(modelS2a.2c)

## # Standardization method: refit
##
## Parameter | Std. Coef. | 95% CI
## ----------------------------------------
## (Intercept) | 8.13e-03 | [-0.13, 0.15]
## leftCEN_Z | 0.19 | [ 0.03, 0.35]
## RADS_total | 0.31 | [ 0.14, 0.47]
## Sex | 0.01 | [-0.14, 0.16]

#Model comparison with LRT
anova(modelS2a.2a, modelS2a.2c)

## refitting model(s) with ML (instead of REML)

## Data: df_aim1_all_language
## Models:
## modelS2a.2a: i_day_prop ~ leftCEN_Z + RADS_total + (1 | id_participant)
## modelS2a.2c: i_day_prop ~ leftCEN_Z + RADS_total + Sex + (1 | id_participant)
## npar AIC BIC logLik deviance Chisq Df Pr(>Chisq)
## modelS2a.2a 5 10987 11015 -5488.3 10977
## modelS2a.2c 6 10989 11022 -5488.3 10977 0.0145 1 0.9041

### S2a.2d Motion

modelS2a.2d <- lmer(formula = i_day_prop ~
 leftCEN_Z + RADS_total
 + motion
 + (1 |id_participant) ,
 data = df_aim1_all_language,
 na.action=na.exclude)
summary(modelS2a.2d)

## Linear mixed model fit by REML. t-tests use Satterthwaite's method [
## lmerModLmerTest]
## Formula: i_day_prop ~ leftCEN_Z + RADS_total + motion + (1 | id_participant)
## Data: df_aim1_all_language
##
## REML criterion at convergence: 10976.1
##
## Scaled residuals:
## Min 1Q Median 3Q Max
## -3.0900 -0.5682 -0.0136 0.4999 7.5064
##
## Random effects:
## Groups Name Variance Std.Dev.
## id_participant (Intercept) 1.75 1.323
## Residual 11.42 3.379
## Number of obs: 2070, groups: id_participant, 34
##
## Fixed effects:
## Estimate Std. Error df t value Pr(>|t|)
## (Intercept) -1.49360 2.81826 34.89811 -0.530 0.599488
## leftCEN_Z 2.30340 0.91623 33.51240 2.514 0.016913 *
## RADS_total 0.06422 0.01570 32.48469 4.091 0.000265 ***
## motion 6.60059 9.35066 27.33060 0.706 0.486227
## ---
## Signif. codes: 0 '***' 0.001 '**' 0.01 '*' 0.05 '.' 0.1 ' ' 1
##
## Correlation of Fixed Effects:
## (Intr) lCEN_Z RADS_t
## leftCEN_Z -0.858
## RADS_total -0.799 0.518
## motion -0.461 0.101 0.246

standardize_parameters(modelS2a.2d)

## # Standardization method: refit
##
## Parameter | Std. Coef. | 95% CI
## ----------------------------------------
## (Intercept) | 0.02 | [-0.12, 0.16]
## leftCEN_Z | 0.20 | [ 0.04, 0.35]
## RADS_total | 0.33 | [ 0.17, 0.48]
## motion | 0.06 | [-0.10, 0.21]

### S2a.2e Days between V2 and EARS start

modelS2a.2e <- lmer(formula = i_day_prop ~
 leftCEN_Z + RADS_total
 + days_since_V2
 + (1 |id_participant) ,
 data = df_aim1_all_language,
 na.action=na.exclude)
summary(modelS2a.2e)

## Linear mixed model fit by REML. t-tests use Satterthwaite's method [
## lmerModLmerTest]
## Formula:
## i_day_prop ~ leftCEN_Z + RADS_total + days_since_V2 + (1 | id_participant)
## Data: df_aim1_all_language
##
## REML criterion at convergence: 10994.1
##
## Scaled residuals:
## Min 1Q Median 3Q Max
## -3.0957 -0.5686 -0.0106 0.5018 7.5108
##
## Random effects:
## Groups Name Variance Std.Dev.
## id_participant (Intercept) 1.752 1.324
## Residual 11.416 3.379
## Number of obs: 2070, groups: id_participant, 34
##
## Fixed effects:
## Estimate Std. Error df t value Pr(>|t|)
## (Intercept) -1.2249833 2.6525415 37.4205676 -0.462 0.646888
## leftCEN_Z 2.5843318 1.0265648 34.4593667 2.517 0.016630 *
## RADS_total 0.0647260 0.0158410 33.4866776 4.086 0.000258 ***
## days_since_V2 -0.0008184 0.0011141 27.6162953 -0.735 0.468825
## ---
## Signif. codes: 0 '***' 0.001 '**' 0.01 '*' 0.05 '.' 0.1 ' ' 1
##
## Correlation of Fixed Effects:
## (Intr) lCEN_Z RADS_t
## leftCEN_Z -0.923
## RADS_total -0.815 0.564
## days_snc_V2 0.333 -0.460 -0.277

standardize_parameters(modelS2a.2e)

## # Standardization method: refit
##
## Parameter | Std. Coef. | 95% CI
## ------------------------------------------
## (Intercept) | 4.54e-03 | [-0.13, 0.14]
## leftCEN_Z | 0.22 | [ 0.05, 0.39]
## RADS_total | 0.33 | [ 0.17, 0.49]
## days_since_V2 | -0.06 | [-0.22, 0.10]

## 2b. Negative Emotion Words (controlling for Group)

### 2b.4a Within-Network SN_CO_Z

model2b.4a <- lmer(formula = negemo_day_prop ~
 SN_CO_Z + Group +
 (1 |id_participant),
 data = df_aim1_all_language,
 na.action=na.exclude)
summary(model2b.4a)

## Linear mixed model fit by REML. t-tests use Satterthwaite's method [
## lmerModLmerTest]
## Formula: negemo_day_prop ~ SN_CO_Z + Group + (1 | id_participant)
## Data: df_aim1_all_language
##
## REML criterion at convergence: 7488.2
##
## Scaled residuals:
## Min 1Q Median 3Q Max
## -2.2065 -0.5776 -0.0863 0.3760 10.1242
##
## Random effects:
## Groups Name Variance Std.Dev.
## id_participant (Intercept) 0.2013 0.4486
## Residual 2.1227 1.4570
## Number of obs: 2070, groups: id_participant, 34
##
## Fixed effects:
## Estimate Std. Error df t value Pr(>|t|)
## (Intercept) 0.2343 0.6074 31.4548 0.386 0.7023
## SN_CO_Z 0.4938 0.2198 31.9641 2.247 0.0317 *
## GroupMDD 1.0546 0.2062 28.6064 5.114 1.92e-05 ***
## ---
## Signif. codes: 0 '***' 0.001 '**' 0.01 '*' 0.05 '.' 0.1 ' ' 1
##
## Correlation of Fixed Effects:
## (Intr) SN_CO_
## SN_CO_Z -0.959
## GroupMDD -0.404 0.174

standardize_parameters(model2b.4a)

## # Standardization method: refit
##
## Parameter | Std. Coef. | 95% CI
## -----------------------------------------
## (Intercept) | -0.49 | [-0.71, -0.26]
## SN_CO_Z | 0.11 | [ 0.01, 0.20]
## GroupMDD | 0.68 | [ 0.42, 0.94]

### 2b.4b Age

model2b.4b <- lmer(formula = negemo_day_prop ~
 SN_CO_Z + Group
 + Age.at.V2
 + (1 |id_participant),
 data = df_aim1_all_language,
 na.action=na.exclude)
summary(model2b.4b)

## Linear mixed model fit by REML. t-tests use Satterthwaite's method [
## lmerModLmerTest]
## Formula: negemo_day_prop ~ SN_CO_Z + Group + Age.at.V2 + (1 | id_participant)
## Data: df_aim1_all_language
##
## REML criterion at convergence: 7491.4
##
## Scaled residuals:
## Min 1Q Median 3Q Max
## -2.2168 -0.5795 -0.0861 0.3768 10.1235
##
## Random effects:
## Groups Name Variance Std.Dev.
## id_participant (Intercept) 0.2098 0.458
## Residual 2.1228 1.457
## Number of obs: 2070, groups: id_participant, 34
##
## Fixed effects:
## Estimate Std. Error df t value Pr(>|t|)
## (Intercept) 0.56832 1.43357 28.03112 0.396 0.6948
## SN_CO_Z 0.49287 0.22315 30.84315 2.209 0.0348 *
## GroupMDD 1.05933 0.20996 27.67426 5.045 2.53e-05 ***
## Age.at.V2 -0.02055 0.08011 26.25877 -0.257 0.7995
## ---
## Signif. codes: 0 '***' 0.001 '**' 0.01 '*' 0.05 '.' 0.1 ' ' 1
##
## Correlation of Fixed Effects:
## (Intr) SN_CO_ GrpMDD
## SN_CO_Z -0.406
## GroupMDD -0.118 0.173
## Age.at.V2 -0.903 -0.007 -0.062

standardize_parameters(model2b.4b)

## # Standardization method: refit
##
## Parameter | Std. Coef. | 95% CI
## -----------------------------------------
## (Intercept) | -0.48 | [-0.71, -0.26]
## SN_CO_Z | 0.11 | [ 0.01, 0.20]
## GroupMDD | 0.68 | [ 0.42, 0.94]
## Age.at.V2 | -0.02 | [-0.14, 0.11]

#Model comparison with LRT
anova(model2b.4a, model2b.4b)

## refitting model(s) with ML (instead of REML)

## Data: df_aim1_all_language
## Models:
## model2b.4a: negemo_day_prop ~ SN_CO_Z + Group + (1 | id_participant)
## model2b.4b: negemo_day_prop ~ SN_CO_Z + Group + Age.at.V2 + (1 | id_participant)
## npar AIC BIC logLik deviance Chisq Df Pr(>Chisq)
## model2b.4a 5 7492.6 7520.8 -3741.3 7482.6
## model2b.4b 6 7494.5 7528.3 -3741.3 7482.5 0.0826 1 0.7738

### 2b.4c Sex

model2b.4c <- lmer(formula = negemo_day_prop ~
 SN_CO_Z + Group
 + Sex
 + (1 |id_participant),
 data = df_aim1_all_language,
 na.action=na.exclude)
summary(model2b.4c)

## Linear mixed model fit by REML. t-tests use Satterthwaite's method [
## lmerModLmerTest]
## Formula: negemo_day_prop ~ SN_CO_Z + Group + Sex + (1 | id_participant)
## Data: df_aim1_all_language
##
## REML criterion at convergence: 7489.5
##
## Scaled residuals:
## Min 1Q Median 3Q Max
## -2.2159 -0.5811 -0.0863 0.3800 10.1266
##
## Random effects:
## Groups Name Variance Std.Dev.
## id_participant (Intercept) 0.2108 0.4591
## Residual 2.1225 1.4569
## Number of obs: 2070, groups: id_participant, 34
##
## Fixed effects:
## Estimate Std. Error df t value Pr(>|t|)
## (Intercept) 0.08281 0.69381 30.60688 0.119 0.906
## SN_CO_Z 0.49580 0.22367 31.25978 2.217 0.034 *
## GroupMDD 1.05050 0.21030 27.84296 4.995 2.85e-05 ***
## Sex 0.09283 0.19047 26.07046 0.487 0.630
## ---
## Signif. codes: 0 '***' 0.001 '**' 0.01 '*' 0.05 '.' 0.1 ' ' 1
##
## Correlation of Fixed Effects:
## (Intr) SN_CO_ GrpMDD
## SN_CO_Z -0.868
## GroupMDD -0.333 0.171
## Sex -0.455 0.032 -0.056

standardize_parameters(model2b.4c)

## # Standardization method: refit
##
## Parameter | Std. Coef. | 95% CI
## -----------------------------------------
## (Intercept) | -0.49 | [-0.71, -0.26]
## SN_CO_Z | 0.11 | [ 0.01, 0.20]
## GroupMDD | 0.67 | [ 0.41, 0.94]
## Sex | 0.03 | [-0.09, 0.15]

#Model comparison with LRT
anova(model2b.4a, model2b.4c)

## refitting model(s) with ML (instead of REML)

## Data: df_aim1_all_language
## Models:
## model2b.4a: negemo_day_prop ~ SN_CO_Z + Group + (1 | id_participant)
## model2b.4c: negemo_day_prop ~ SN_CO_Z + Group + Sex + (1 | id_participant)
## npar AIC BIC logLik deviance Chisq Df Pr(>Chisq)
## model2b.4a 5 7492.6 7520.8 -3741.3 7482.6
## model2b.4c 6 7494.3 7528.2 -3741.2 7482.3 0.2377 1 0.6259

### 2b.4d Motion

model2b.4d <- lmer(formula = negemo_day_prop ~
 SN_CO_Z + Group
 + motion
 + (1 |id_participant),
 data = df_aim1_all_language,
 na.action=na.exclude)
summary(model2b.4d)

## Linear mixed model fit by REML. t-tests use Satterthwaite's method [
## lmerModLmerTest]
## Formula: negemo_day_prop ~ SN_CO_Z + Group + motion + (1 | id_participant)
## Data: df_aim1_all_language
##
## REML criterion at convergence: 7482
##
## Scaled residuals:
## Min 1Q Median 3Q Max
## -2.2140 -0.5768 -0.0864 0.3741 10.1161
##
## Random effects:
## Groups Name Variance Std.Dev.
## id_participant (Intercept) 0.193 0.4393
## Residual 2.123 1.4569
## Number of obs: 2070, groups: id_participant, 34
##
## Fixed effects:
## Estimate Std. Error df t value Pr(>|t|)
## (Intercept) 0.5239 0.6321 28.6262 0.829 0.4140
## SN_CO_Z 0.5674 0.2223 30.9155 2.552 0.0159 *
## GroupMDD 0.9889 0.2079 27.1206 4.758 5.78e-05 ***
## motion -4.7495 3.3301 24.3412 -1.426 0.1665
## ---
## Signif. codes: 0 '***' 0.001 '**' 0.01 '*' 0.05 '.' 0.1 ' ' 1
##
## Correlation of Fixed Effects:
## (Intr) SN_CO_ GrpMDD
## SN_CO_Z -0.810
## GroupMDD -0.443 0.116
## motion -0.324 -0.228 0.217

standardize_parameters(model2b.4d)

## # Standardization method: refit
##
## Parameter | Std. Coef. | 95% CI
## -----------------------------------------
## (Intercept) | -0.47 | [-0.69, -0.25]
## SN_CO_Z | 0.12 | [ 0.03, 0.22]
## GroupMDD | 0.63 | [ 0.37, 0.90]
## motion | -0.09 | [-0.22, 0.04]

### 2b.4e Days between V2 and EARS start

model2b.4e <- lmer(formula = negemo_day_prop ~
 SN_CO_Z + Group
 + days_since_V2
 + (1 |id_participant),
 data = df_aim1_all_language,
 na.action=na.exclude)
summary(model2b.4e)

## Linear mixed model fit by REML. t-tests use Satterthwaite's method [
## lmerModLmerTest]
## Formula:
## negemo_day_prop ~ SN_CO_Z + Group + days_since_V2 + (1 | id_participant)
## Data: df_aim1_all_language
##
## REML criterion at convergence: 7501.8
##
## Scaled residuals:
## Min 1Q Median 3Q Max
## -2.2078 -0.5785 -0.0879 0.3771 10.1189
##
## Random effects:
## Groups Name Variance Std.Dev.
## id_participant (Intercept) 0.205 0.4528
## Residual 2.123 1.4570
## Number of obs: 2070, groups: id_participant, 34
##
## Fixed effects:
## Estimate Std. Error df t value Pr(>|t|)
## (Intercept) 0.2844685 0.6158219 30.4075195 0.462 0.6474
## SN_CO_Z 0.5051348 0.2219646 30.2010260 2.276 0.0301 *
## GroupMDD 1.0454086 0.2082084 27.3051728 5.021 2.8e-05 ***
## days_since_V2 -0.0002413 0.0003542 25.0644468 -0.681 0.5020
## ---
## Signif. codes: 0 '***' 0.001 '**' 0.01 '*' 0.05 '.' 0.1 ' ' 1
##
## Correlation of Fixed Effects:
## (Intr) SN_CO_ GrpMDD
## SN_CO_Z -0.940
## GroupMDD -0.408 0.167
## days_snc_V2 -0.117 -0.079 0.069

standardize_parameters(model2b.4e)

## # Standardization method: refit
##
## Parameter | Std. Coef. | 95% CI
## -------------------------------------------
## (Intercept) | -0.48 | [-0.71, -0.26]
## SN_CO_Z | 0.11 | [ 0.02, 0.21]
## GroupMDD | 0.67 | [ 0.41, 0.93]
## days_since_V2 | -0.04 | [-0.16, 0.08]

## S2b. Negative Emotion Words (controlling for depressive symptoms)

### S2b.4a Within-Network SN_CO_Z

modelS2b.4a <- lmer(formula = negemo_day_prop ~
 SN_CO_Z + RADS_total
 + (1 |id_participant) ,
 data = df_aim1_all_language,
 na.action=na.exclude)
summary(modelS2b.4a)

## Linear mixed model fit by REML. t-tests use Satterthwaite's method [
## lmerModLmerTest]
## Formula: negemo_day_prop ~ SN_CO_Z + RADS_total + (1 | id_participant)
## Data: df_aim1_all_language
##
## REML criterion at convergence: 7498.1
##
## Scaled residuals:
## Min 1Q Median 3Q Max
## -2.2851 -0.5794 -0.0829 0.3822 10.1241
##
## Random effects:
## Groups Name Variance Std.Dev.
## id_participant (Intercept) 0.2041 0.4518
## Residual 2.1248 1.4577
## Number of obs: 2070, groups: id_participant, 34
##
## Fixed effects:
## Estimate Std. Error df t value Pr(>|t|)
## (Intercept) -1.039218 0.763732 29.846689 -1.361 0.1838
## SN_CO_Z 0.618096 0.227345 30.658083 2.719 0.0107 *
## RADS_total 0.023574 0.004856 25.786385 4.855 5.03e-05 ***
## ---
## Signif. codes: 0 '***' 0.001 '**' 0.01 '*' 0.05 '.' 0.1 ' ' 1
##
## Correlation of Fixed Effects:
## (Intr) SN_CO_
## SN_CO_Z -0.888
## RADS_total -0.682 0.290

standardize_parameters(modelS2b.4a)

## # Standardization method: refit
##
## Parameter | Std. Coef. | 95% CI
## ----------------------------------------
## (Intercept) | 0.02 | [-0.09, 0.14]
## SN_CO_Z | 0.14 | [ 0.04, 0.23]
## RADS_total | 0.28 | [ 0.17, 0.40]

### S2b.4b Age

modelS2b.4b <- lmer(formula = negemo_day_prop ~
 SN_CO_Z + RADS_total
 + Age.at.V2
 + (1 |id_participant) ,
 data = df_aim1_all_language,
 na.action=na.exclude)
summary(modelS2b.4b)

## Linear mixed model fit by REML. t-tests use Satterthwaite's method [
## lmerModLmerTest]
## Formula:
## negemo_day_prop ~ SN_CO_Z + RADS_total + Age.at.V2 + (1 | id_participant)
## Data: df_aim1_all_language
##
## REML criterion at convergence: 7500.9
##
## Scaled residuals:
## Min 1Q Median 3Q Max
## -2.2755 -0.5804 -0.0823 0.3828 10.1206
##
## Random effects:
## Groups Name Variance Std.Dev.
## id_participant (Intercept) 0.2131 0.4617
## Residual 2.1245 1.4576
## Number of obs: 2070, groups: id_participant, 34
##
## Fixed effects:
## Estimate Std. Error df t value Pr(>|t|)
## (Intercept) -1.823955 1.583274 26.412692 -1.152 0.2596
## SN_CO_Z 0.620320 0.231070 29.710730 2.685 0.0118 *
## RADS_total 0.023889 0.004968 24.965379 4.809 6.14e-05 ***
## Age.at.V2 0.046311 0.080947 24.471955 0.572 0.5725
## ---
## Signif. codes: 0 '***' 0.001 '**' 0.01 '*' 0.05 '.' 0.1 ' ' 1
##
## Correlation of Fixed Effects:
## (Intr) SN_CO_ RADS_t
## SN_CO_Z -0.465
## RADS_total -0.426 0.291
## Age.at.V2 -0.872 0.035 0.108

standardize_parameters(modelS2b.4b)

## # Standardization method: refit
##
## Parameter | Std. Coef. | 95% CI
## ----------------------------------------
## (Intercept) | 0.01 | [-0.11, 0.13]
## SN_CO_Z | 0.14 | [ 0.04, 0.23]
## RADS_total | 0.29 | [ 0.17, 0.41]
## Age.at.V2 | 0.04 | [-0.09, 0.16]

#Model comparison with LRT
anova(modelS2b.4a, modelS2b.4b)

## refitting model(s) with ML (instead of REML)

## Data: df_aim1_all_language
## Models:
## modelS2b.4a: negemo_day_prop ~ SN_CO_Z + RADS_total + (1 | id_participant)
## modelS2b.4b: negemo_day_prop ~ SN_CO_Z + RADS_total + Age.at.V2 + (1 | id_participant)
## npar AIC BIC logLik deviance Chisq Df Pr(>Chisq)
## modelS2b.4a 5 7494.9 7523.1 -3742.5 7484.9
## modelS2b.4b 6 7496.6 7530.4 -3742.3 7484.6 0.3498 1 0.5542

### S2b.4c Sex

modelS2b.4c <- lmer(formula = negemo_day_prop ~
 SN_CO_Z + RADS_total
 + Sex
 + (1 |id_participant) ,
 data = df_aim1_all_language,
 na.action=na.exclude)
summary(modelS2b.4c)

## Linear mixed model fit by REML. t-tests use Satterthwaite's method [
## lmerModLmerTest]
## Formula: negemo_day_prop ~ SN_CO_Z + RADS_total + Sex + (1 | id_participant)
## Data: df_aim1_all_language
##
## REML criterion at convergence: 7498.6
##
## Scaled residuals:
## Min 1Q Median 3Q Max
## -2.2819 -0.5781 -0.0826 0.3800 10.1155
##
## Random effects:
## Groups Name Variance Std.Dev.
## id_participant (Intercept) 0.1994 0.4466
## Residual 2.1255 1.4579
## Number of obs: 2070, groups: id_participant, 34
##
## Fixed effects:
## Estimate Std. Error df t value Pr(>|t|)
## (Intercept) -0.895788 0.773782 26.860491 -1.158 0.25719
## SN_CO_Z 0.631606 0.225841 27.784248 2.797 0.00926 **
## RADS_total 0.025131 0.005103 23.104103 4.925 5.55e-05 ***
## Sex -0.181254 0.197277 22.325155 -0.919 0.36803
## ---
## Signif. codes: 0 '***' 0.001 '**' 0.01 '*' 0.05 '.' 0.1 ' ' 1
##
## Correlation of Fixed Effects:
## (Intr) SN_CO_ RADS_t
## SN_CO_Z -0.856
## RADS_total -0.561 0.293
## Sex -0.205 -0.059 -0.333

standardize_parameters(modelS2b.4c)

## # Standardization method: refit
##
## Parameter | Std. Coef. | 95% CI
## ----------------------------------------
## (Intercept) | 0.03 | [-0.09, 0.15]
## SN_CO_Z | 0.14 | [ 0.04, 0.24]
## RADS_total | 0.30 | [ 0.18, 0.42]
## Sex | -0.06 | [-0.18, 0.07]

#Model comparison with LRT
anova(modelS2b.4a, modelS2b.4c)

## refitting model(s) with ML (instead of REML)

## Data: df_aim1_all_language
## Models:
## modelS2b.4a: negemo_day_prop ~ SN_CO_Z + RADS_total + (1 | id_participant)
## modelS2b.4c: negemo_day_prop ~ SN_CO_Z + RADS_total + Sex + (1 | id_participant)
## npar AIC BIC logLik deviance Chisq Df Pr(>Chisq)
## modelS2b.4a 5 7494.9 7523.1 -3742.5 7484.9
## modelS2b.4c 6 7495.9 7529.7 -3741.9 7483.9 1.055 1 0.3044

### S2b.4d Motion

modelS2b.4d <- lmer(formula = negemo_day_prop ~
 SN_CO_Z + RADS_total
 + motion
 + (1 |id_participant) ,
 data = df_aim1_all_language,
 na.action=na.exclude)
summary(modelS2b.4d)

## Linear mixed model fit by REML. t-tests use Satterthwaite's method [
## lmerModLmerTest]
## Formula: negemo_day_prop ~ SN_CO_Z + RADS_total + motion + (1 | id_participant)
## Data: df_aim1_all_language
##
## REML criterion at convergence: 7491
##
## Scaled residuals:
## Min 1Q Median 3Q Max
## -2.2925 -0.5790 -0.0812 0.3807 10.1157
##
## Random effects:
## Groups Name Variance Std.Dev.
## id_participant (Intercept) 0.1929 0.4392
## Residual 2.1242 1.4575
## Number of obs: 2070, groups: id_participant, 34
##
## Fixed effects:
## Estimate Std. Error df t value Pr(>|t|)
## (Intercept) -0.642714 0.785467 28.155906 -0.818 0.420082
## SN_CO_Z 0.700367 0.227668 29.599027 3.076 0.004483 **
## RADS_total 0.022154 0.004822 25.039848 4.595 0.000106 ***
## motion -5.549861 3.300313 23.217279 -1.682 0.106048
## ---
## Signif. codes: 0 '***' 0.001 '**' 0.01 '*' 0.05 '.' 0.1 ' ' 1
##
## Correlation of Fixed Effects:
## (Intr) SN_CO_ RADS_t
## SN_CO_Z -0.765
## RADS_total -0.693 0.245
## motion -0.305 -0.207 0.174

standardize_parameters(modelS2b.4d)

## # Standardization method: refit
##
## Parameter | Std. Coef. | 95% CI
## ----------------------------------------
## (Intercept) | 3.15e-03 | [-0.11, 0.12]
## SN_CO_Z | 0.15 | [ 0.06, 0.25]
## RADS_total | 0.27 | [ 0.15, 0.38]
## motion | -0.11 | [-0.24, 0.02]

### S2b.4e Days between V2 and EARS start

modelS2b.4e <- lmer(formula = negemo_day_prop ~
 SN_CO_Z + RADS_total
 + days_since_V2
 + (1 |id_participant) ,
 data = df_aim1_all_language,
 na.action=na.exclude)
summary(modelS2b.4e)

## Linear mixed model fit by REML. t-tests use Satterthwaite's method [
## lmerModLmerTest]
## Formula: negemo_day_prop ~ SN_CO_Z + RADS_total + days_since_V2 + (1 |
## id_participant)
## Data: df_aim1_all_language
##
## REML criterion at convergence: 7510.4
##
## Scaled residuals:
## Min 1Q Median 3Q Max
## -2.2538 -0.5776 -0.0824 0.3822 10.1162
##
## Random effects:
## Groups Name Variance Std.Dev.
## id_participant (Intercept) 0.1947 0.4412
## Residual 2.1251 1.4578
## Number of obs: 2070, groups: id_participant, 34
##
## Fixed effects:
## Estimate Std. Error df t value Pr(>|t|)
## (Intercept) -1.0102238 0.7510739 28.4042372 -1.345 0.18925
## SN_CO_Z 0.6517291 0.2247021 28.7281783 2.900 0.00708 **
## RADS_total 0.0239274 0.0047738 24.4180538 5.012 3.85e-05 ***
## days_since_V2 -0.0004639 0.0003467 23.1351536 -1.338 0.19385
## ---
## Signif. codes: 0 '***' 0.001 '**' 0.01 '*' 0.05 '.' 0.1 ' ' 1
##
## Correlation of Fixed Effects:
## (Intr) SN_CO_ RADS_t
## SN_CO_Z -0.880
## RADS_total -0.679 0.295
## days_snc_V2 -0.034 -0.103 -0.057

standardize_parameters(modelS2b.4e)

## # Standardization method: refit
##
## Parameter | Std. Coef. | 95% CI
## ------------------------------------------
## (Intercept) | 0.01 | [-0.10, 0.13]
## SN_CO_Z | 0.14 | [ 0.05, 0.24]
## RADS_total | 0.29 | [ 0.18, 0.40]
## days_since_V2 | -0.08 | [-0.20, 0.04]

## 2c. Future focus words (controlling for Group)

### 2c.1a Within-Network aDMN

model2c.1a <- lmer(formula = focusfuture_day_prop ~
 aDMN_Z + Group +
 (1 |id_participant),
 data = df_aim1_all_language,
 na.action=na.exclude)
summary(model2c.1a)

## Linear mixed model fit by REML. t-tests use Satterthwaite's method [
## lmerModLmerTest]
## Formula: focusfuture_day_prop ~ aDMN_Z + Group + (1 | id_participant)
## Data: df_aim1_all_language
##
## REML criterion at convergence: 6305.9
##
## Scaled residuals:
## Min 1Q Median 3Q Max
## -1.9513 -0.6115 -0.0903 0.4230 8.0910
##
## Random effects:
## Groups Name Variance Std.Dev.
## id_participant (Intercept) 0.1282 0.358
## Residual 1.1968 1.094
## Number of obs: 2070, groups: id_participant, 34
##
## Fixed effects:
## Estimate Std. Error df t value Pr(>|t|)
## (Intercept) 1.0226 0.3654 30.5717 2.798 0.00881 **
## aDMN_Z 0.4610 0.2176 31.5882 2.119 0.04205 *
## GroupMDD -0.3010 0.1608 25.4826 -1.872 0.07275 .
## ---
## Signif. codes: 0 '***' 0.001 '**' 0.01 '*' 0.05 '.' 0.1 ' ' 1
##
## Correlation of Fixed Effects:
## (Intr) aDMN_Z
## aDMN_Z -0.928
## GroupMDD -0.407 0.100

standardize_parameters(model2c.1a)

## # Standardization method: refit
##
## Parameter | Std. Coef. | 95% CI
## ----------------------------------------
## (Intercept) | 0.27 | [ 0.04, 0.51]
## aDMN_Z | 0.11 | [ 0.01, 0.21]
## GroupMDD | -0.26 | [-0.54, 0.01]

### 2c.1b Age

model2c.1b <- lmer(formula = focusfuture_day_prop ~
 aDMN_Z + Group
 + Age.at.V2
 + (1 |id_participant),
 data = df_aim1_all_language,
 na.action=na.exclude)
summary(model2c.1b)

## Linear mixed model fit by REML. t-tests use Satterthwaite's method [
## lmerModLmerTest]
## Formula:
## focusfuture_day_prop ~ aDMN_Z + Group + Age.at.V2 + (1 | id_participant)
## Data: df_aim1_all_language
##
## REML criterion at convergence: 6305.8
##
## Scaled residuals:
## Min 1Q Median 3Q Max
## -1.9432 -0.6063 -0.0875 0.4193 8.0886
##
## Random effects:
## Groups Name Variance Std.Dev.
## id_participant (Intercept) 0.1147 0.3387
## Residual 1.1965 1.0939
## Number of obs: 2070, groups: id_participant, 34
##
## Fixed effects:
## Estimate Std. Error df t value Pr(>|t|)
## (Intercept) -0.94480 1.05033 27.10618 -0.900 0.3763
## aDMN_Z 0.48062 0.20981 31.69638 2.291 0.0288 *
## GroupMDD -0.31824 0.15415 24.72349 -2.064 0.0496 *
## Age.at.V2 0.11943 0.05959 24.11483 2.004 0.0564 .
## ---
## Signif. codes: 0 '***' 0.001 '**' 0.01 '*' 0.05 '.' 0.1 ' ' 1
##
## Correlation of Fixed Effects:
## (Intr) aDMN_Z GrpMDD
## aDMN_Z -0.380
## GroupMDD -0.085 0.097
## Age.at.V2 -0.942 0.074 -0.054

standardize_parameters(model2c.1b)

## # Standardization method: refit
##
## Parameter | Std. Coef. | 95% CI
## -----------------------------------------
## (Intercept) | 0.25 | [ 0.03, 0.48]
## aDMN_Z | 0.11 | [ 0.02, 0.21]
## GroupMDD | -0.28 | [-0.54, -0.01]
## Age.at.V2 | 0.13 | [ 0.00, 0.26]

#Model comparison with LRT
anova(model2c.1a, model2c.1b)

## refitting model(s) with ML (instead of REML)

## Data: df_aim1_all_language
## Models:
## model2c.1a: focusfuture_day_prop ~ aDMN_Z + Group + (1 | id_participant)
## model2c.1b: focusfuture_day_prop ~ aDMN_Z + Group + Age.at.V2 + (1 | id_participant)
## npar AIC BIC logLik deviance Chisq Df Pr(>Chisq)
## model2c.1a 5 6309.3 6337.5 -3149.7 6299.3
## model2c.1b 6 6307.0 6340.8 -3147.5 6295.0 4.3104 1 0.03788 *
## ---
## Signif. codes: 0 '***' 0.001 '**' 0.01 '*' 0.05 '.' 0.1 ' ' 1

### 2c.1c Sex

model2c.1c <- lmer(formula = focusfuture_day_prop ~
 aDMN_Z + Group
 + Sex
 + (1 |id_participant),
 data = df_aim1_all_language,
 na.action=na.exclude)
summary(model2c.1c)

## Linear mixed model fit by REML. t-tests use Satterthwaite's method [
## lmerModLmerTest]
## Formula: focusfuture_day_prop ~ aDMN_Z + Group + Sex + (1 | id_participant)
## Data: df_aim1_all_language
##
## REML criterion at convergence: 6307.2
##
## Scaled residuals:
## Min 1Q Median 3Q Max
## -1.9484 -0.6021 -0.0862 0.4198 8.0869
##
## Random effects:
## Groups Name Variance Std.Dev.
## id_participant (Intercept) 0.1328 0.3645
## Residual 1.1965 1.0938
## Number of obs: 2070, groups: id_participant, 34
##
## Fixed effects:
## Estimate Std. Error df t value Pr(>|t|)
## (Intercept) 0.8152 0.4378 32.7949 1.862 0.0716 .
## aDMN_Z 0.4653 0.2204 31.0900 2.111 0.0429 *
## GroupMDD -0.3097 0.1634 24.9374 -1.895 0.0697 .
## Sex 0.1293 0.1496 23.5198 0.864 0.3962
## ---
## Signif. codes: 0 '***' 0.001 '**' 0.01 '*' 0.05 '.' 0.1 ' ' 1
##
## Correlation of Fixed Effects:
## (Intr) aDMN_Z GrpMDD
## aDMN_Z -0.786
## GroupMDD -0.309 0.100
## Sex -0.534 0.002 -0.063

standardize_parameters(model2c.1c)

## # Standardization method: refit
##
## Parameter | Std. Coef. | 95% CI
## ----------------------------------------
## (Intercept) | 0.27 | [ 0.04, 0.51]
## aDMN_Z | 0.11 | [ 0.01, 0.21]
## GroupMDD | -0.27 | [-0.55, 0.01]
## Sex | 0.06 | [-0.07, 0.18]

#Model comparison with LRT
anova(model2c.1a, model2c.1c)

## refitting model(s) with ML (instead of REML)

## Data: df_aim1_all_language
## Models:
## model2c.1a: focusfuture_day_prop ~ aDMN_Z + Group + (1 | id_participant)
## model2c.1c: focusfuture_day_prop ~ aDMN_Z + Group + Sex + (1 | id_participant)
## npar AIC BIC logLik deviance Chisq Df Pr(>Chisq)
## model2c.1a 5 6309.3 6337.5 -3149.7 6299.3
## model2c.1c 6 6310.5 6344.3 -3149.3 6298.5 0.791 1 0.3738

### 2c.1d Motion

model2c.1d <- lmer(formula = focusfuture_day_prop ~
 aDMN_Z + Group
 + motion
 + (1 |id_participant),
 data = df_aim1_all_language,
 na.action=na.exclude)
summary(model2c.1d)

## Linear mixed model fit by REML. t-tests use Satterthwaite's method [
## lmerModLmerTest]
## Formula: focusfuture_day_prop ~ aDMN_Z + Group + motion + (1 | id_participant)
## Data: df_aim1_all_language
##
## REML criterion at convergence: 6302.1
##
## Scaled residuals:
## Min 1Q Median 3Q Max
## -1.9536 -0.6106 -0.0903 0.4209 8.0905
##
## Random effects:
## Groups Name Variance Std.Dev.
## id_participant (Intercept) 0.1359 0.3686
## Residual 1.1966 1.0939
## Number of obs: 2070, groups: id_participant, 34
##
## Fixed effects:
## Estimate Std. Error df t value Pr(>|t|)
## (Intercept) 1.0905 0.4925 24.8161 2.214 0.0362 *
## aDMN_Z 0.4599 0.2242 29.9628 2.051 0.0491 *
## GroupMDD -0.3114 0.1706 24.5878 -1.825 0.0802 .
## motion -0.6422 2.6939 22.0342 -0.238 0.8138
## ---
## Signif. codes: 0 '***' 0.001 '**' 0.01 '*' 0.05 '.' 0.1 ' ' 1
##
## Correlation of Fixed Effects:
## (Intr) aDMN_Z GrpMDD
## aDMN_Z -0.785
## GroupMDD -0.470 0.131
## motion -0.652 0.136 0.265

standardize_parameters(model2c.1d)

## # Standardization method: refit
##
## Parameter | Std. Coef. | 95% CI
## ----------------------------------------
## (Intercept) | 0.28 | [ 0.04, 0.52]
## aDMN_Z | 0.11 | [ 0.00, 0.21]
## GroupMDD | -0.27 | [-0.56, 0.02]
## motion | -0.02 | [-0.16, 0.13]

### 2c.1e Days between V2 and EARS start

model2c.1e <- lmer(formula = focusfuture_day_prop ~
 aDMN_Z + Group
 + days_since_V2
 + (1 |id_participant),
 data = df_aim1_all_language,
 na.action=na.exclude)
summary(model2c.1e)

## Linear mixed model fit by REML. t-tests use Satterthwaite's method [
## lmerModLmerTest]
## Formula: focusfuture_day_prop ~ aDMN_Z + Group + days_since_V2 + (1 |
## id_participant)
## Data: df_aim1_all_language
##
## REML criterion at convergence: 6320.3
##
## Scaled residuals:
## Min 1Q Median 3Q Max
## -1.9539 -0.6110 -0.0901 0.4218 8.0936
##
## Random effects:
## Groups Name Variance Std.Dev.
## id_participant (Intercept) 0.1345 0.3668
## Residual 1.1967 1.0939
## Number of obs: 2070, groups: id_participant, 34
##
## Fixed effects:
## Estimate Std. Error df t value Pr(>|t|)
## (Intercept) 1.011e+00 3.722e-01 2.962e+01 2.716 0.0109 *
## aDMN_Z 4.513e-01 2.274e-01 2.964e+01 1.984 0.0565 .
## GroupMDD -2.977e-01 1.642e-01 2.469e+01 -1.813 0.0820 .
## days_since_V2 8.183e-05 2.898e-04 2.245e+01 0.282 0.7803
## ---
## Signif. codes: 0 '***' 0.001 '**' 0.01 '*' 0.05 '.' 0.1 ' ' 1
##
## Correlation of Fixed Effects:
## (Intr) aDMN_Z GrpMDD
## aDMN_Z -0.893
## GroupMDD -0.408 0.082
## days_snc_V2 -0.042 -0.230 0.064

standardize_parameters(model2c.1e)

## # Standardization method: refit
##
## Parameter | Std. Coef. | 95% CI
## ------------------------------------------
## (Intercept) | 0.27 | [ 0.04, 0.51]
## aDMN_Z | 0.10 | [ 0.00, 0.21]
## GroupMDD | -0.26 | [-0.54, 0.02]
## days_since_V2 | 0.02 | [-0.11, 0.15]

# 3. AIM 3: Mediation: Does intrinsic network connectivity mediate the association between depression and depression-related linguistic features of smartphone use?

## 3a.1 Dep Sx - leftCEN - First-person Pronouns

#install.packages("RMediation")
library(RMediation)

## Loading required package: lavaan

## This is lavaan 0.6-16
## lavaan is FREE software! Please report any bugs.

##
## Attaching package: 'lavaan'

## The following object is masked from 'package:psych':
##
## cor2cov

## Loading required package: e1071

## Loading required package: OpenMx

## OpenMx may run faster if it is compiled to take advantage of multiple cores.

##
## Attaching package: 'OpenMx'

## The following objects are masked from 'package:Matrix':
##
## %&%, expm

## The following object is masked from 'package:psych':
##
## tr

fit1 <- lmer(scale(i_day_prop) ~ 1 + scale(RADS_total) + scale(Age.at.V2) + Sex + (1|id_participant),
 data = df_aim1_all_language,
 na.action=na.exclude)

fit2<- lm(scale(leftCEN_Z) ~ scale(RADS_total) + scale(Age.at.V2) + Sex, df_px_means, na.action=na.exclude)

fit3 <- lmer(scale(i_day_prop) ~ 1 + scale(RADS_total) + scale(leftCEN_Z) + scale(Age.at.V2) + Sex + (1|id_participant),
 data = df_aim1_all_language,
 na.action=na.exclude)

summary(fit1)

## Linear mixed model fit by REML. t-tests use Satterthwaite's method [
## lmerModLmerTest]
## Formula: scale(i_day_prop) ~ 1 + scale(RADS_total) + scale(Age.at.V2) +
## Sex + (1 | id_participant)
## Data: df_aim1_all_language
##
## REML criterion at convergence: 6089.1
##
## Scaled residuals:
## Min 1Q Median 3Q Max
## -3.1275 -0.5758 -0.0125 0.5057 7.4829
##
## Random effects:
## Groups Name Variance Std.Dev.
## id_participant (Intercept) 0.1464 0.3826
## Residual 0.8430 0.9182
## Number of obs: 2251, groups: id_participant, 40
##
## Fixed effects:
## Estimate Std. Error df t value Pr(>|t|)
## (Intercept) -0.18796 0.25294 31.83071 -0.743 0.4629
## scale(RADS_total) 0.16888 0.06885 32.48517 2.453 0.0197 *
## scale(Age.at.V2) -0.01481 0.07313 32.40718 -0.202 0.8408
## Sex 0.12288 0.14862 32.79945 0.827 0.4143
## ---
## Signif. codes: 0 '***' 0.001 '**' 0.01 '*' 0.05 '.' 0.1 ' ' 1
##
## Correlation of Fixed Effects:
## (Intr) s(RADS s(A..V
## scl(RADS_t) 0.302
## scl(Ag..V2) -0.113 0.083
## Sex -0.960 -0.292 0.064

summary(fit2)

##
## Call:
## lm(formula = scale(leftCEN_Z) ~ scale(RADS_total) + scale(Age.at.V2) +
## Sex, data = df_px_means, na.action = na.exclude)
##
## Residuals:
## Min 1Q Median 3Q Max
## -2.06815 -0.41067 0.01125 0.50257 1.81677
##
## Coefficients:
## Estimate Std. Error t value Pr(>|t|)
## (Intercept) -0.4839 0.5876 -0.824 0.417
## scale(RADS_total) -0.4457 0.1666 -2.675 0.012 *
## scale(Age.at.V2) 0.1547 0.1608 0.962 0.344
## Sex 0.2992 0.3441 0.870 0.391
## ---
## Signif. codes: 0 '***' 0.001 '**' 0.01 '*' 0.05 '.' 0.1 ' ' 1
##
## Residual standard error: 0.9261 on 30 degrees of freedom
## (6 observations deleted due to missingness)
## Multiple R-squared: 0.2203, Adjusted R-squared: 0.1423
## F-statistic: 2.825 on 3 and 30 DF, p-value: 0.05541

summary(fit3)

## Linear mixed model fit by REML. t-tests use Satterthwaite's method [
## lmerModLmerTest]
## Formula: scale(i_day_prop) ~ 1 + scale(RADS_total) + scale(leftCEN_Z) +
## scale(Age.at.V2) + Sex + (1 | id_participant)
## Data: df_aim1_all_language
##
## REML criterion at convergence: 5576.6
##
## Scaled residuals:
## Min 1Q Median 3Q Max
## -3.0957 -0.5692 -0.0126 0.4975 7.5113
##
## Random effects:
## Groups Name Variance Std.Dev.
## id_participant (Intercept) 0.1361 0.3689
## Residual 0.8342 0.9134
## Number of obs: 2070, groups: id_participant, 34
##
## Fixed effects:
## Estimate Std. Error df t value Pr(>|t|)
## (Intercept) -0.02314 0.26941 26.15059 -0.086 0.93222
## scale(RADS_total) 0.30797 0.08562 31.61078 3.597 0.00108 **
## scale(leftCEN_Z) 0.19445 0.08243 31.98907 2.359 0.02459 *
## scale(Age.at.V2) -0.03028 0.07604 26.67876 -0.398 0.69366
## Sex 0.01827 0.15891 27.08035 0.115 0.90933
## ---
## Signif. codes: 0 '***' 0.001 '**' 0.01 '*' 0.05 '.' 0.1 ' ' 1
##
## Correlation of Fixed Effects:
## (Intr) s(RADS s(CEN_ s(A..V
## scl(RADS_t) 0.365
## scl(lCEN_Z) 0.197 0.532
## scl(Ag..V2) -0.165 0.004 -0.112
## Sex -0.961 -0.376 -0.203 0.092

standardize_parameters(fit1)

## # Standardization method: refit
##
## Parameter | Std. Coef. | 95% CI
## ----------------------------------------------
## (Intercept) | 3.64e-03 | [-0.14, 0.14]
## scale(RADS_total) | 0.17 | [ 0.03, 0.30]
## scale(Age.at.V2) | -0.01 | [-0.16, 0.13]
## Sex | 0.06 | [-0.08, 0.21]

standardize_parameters(fit2)

## # Standardization method: refit
##
## Parameter | Std. Coef. | 95% CI
## -----------------------------------------------
## (Intercept) | -8.75e-18 | [-0.32, 0.32]
## scale(RADS_total) | -0.45 | [-0.79, -0.11]
## scale(Age.at.V2) | 0.16 | [-0.17, 0.49]
## Sex | 0.15 | [-0.20, 0.49]

standardize_parameters(fit3)

## # Standardization method: refit
##
## Parameter | Std. Coef. | 95% CI
## ----------------------------------------------
## (Intercept) | 0.02 | [-0.13, 0.16]
## scale(RADS_total) | 0.31 | [ 0.14, 0.47]
## scale(leftCEN_Z) | 0.19 | [ 0.03, 0.36]
## scale(Age.at.V2) | -0.03 | [-0.18, 0.12]
## Sex | 9.08e-03 | [-0.15, 0.16]

fitb <- lmer(scale(i_day_prop) ~ 1 + scale(leftCEN_Z) + (1|id_participant),
 data = df_aim1_all_language,
 na.action=na.exclude)
summary(fitb)

## Linear mixed model fit by REML. t-tests use Satterthwaite's method [
## lmerModLmerTest]
## Formula: scale(i_day_prop) ~ 1 + scale(leftCEN_Z) + (1 | id_participant)
## Data: df_aim1_all_language
##
## REML criterion at convergence: 5582.2
##
## Scaled residuals:
## Min 1Q Median 3Q Max
## -3.1488 -0.5711 -0.0154 0.4973 7.4923
##
## Random effects:
## Groups Name Variance Std.Dev.
## id_participant (Intercept) 0.2007 0.4480
## Residual 0.8346 0.9136
## Number of obs: 2070, groups: id_participant, 34
##
## Fixed effects:
## Estimate Std. Error df t value Pr(>|t|)
## (Intercept) -0.01665 0.08450 29.10141 -0.197 0.845
## scale(leftCEN_Z) 0.04050 0.08171 29.23676 0.496 0.624
##
## Correlation of Fixed Effects:
## (Intr)
## scl(lCEN_Z) -0.038

standardize_parameters(fitb)

## # Standardization method: refit
##
## Parameter | Std. Coef. | 95% CI
## ---------------------------------------------
## (Intercept) | -0.02 | [-0.18, 0.15]
## scale(leftCEN_Z) | 0.04 | [-0.12, 0.20]

# CI for a*b:
c=summary(fit1)$coefficients[2,1]
a = summary(fit2)$coefficients[2,1]
se.a = summary(fit2)$coefficients[2,2]
b = summary(fit3)$coefficients[2,1]
se.b = summary(fit3)$coefficients[2,2]


medci(mu.x=a, mu.y=b, se.x=se.a, se.y=se.b, rho=0, alpha=.05, type="prodclin", plot=TRUE, plotCI=TRUE)

## Warning in arrows(MedCI[[1]][1], yci, MedCI[[1]][2], yci, length = smidge, :
## 'length', 'angle', or 'code' greater than length 1; values after the first are
## ignored


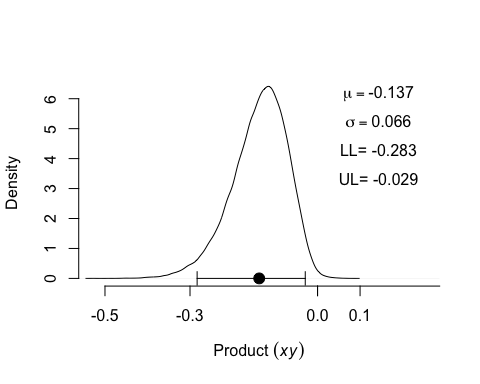


## $`95% CI`
## [1] -0.28326720 -0.02894307
##
## $Estimate
## [1] -0.1372595
##
## $SE
## [1] 0.06551041

### 3a.2 Motion

#install.packages("RMediation")
library(RMediation)

fit1 <- lmer(scale(i_day_prop) ~ 1 + scale(RADS_total) + scale(Age.at.V2) + Sex + (1|id_participant),
 data = df_aim1_all_language,
 na.action=na.exclude)

fit2<- lm(scale(leftCEN_Z) ~ scale(RADS_total) + scale(Age.at.V2) + Sex + scale(motion), df_px_means, na.action=na.exclude)

fit3 <- lmer(scale(i_day_prop) ~ 1 + scale(RADS_total) + scale(leftCEN_Z) + scale(Age.at.V2) + Sex + scale(motion) + (1|id_participant),
 data = df_aim1_all_language,
 na.action=na.exclude)

summary(fit1)

## Linear mixed model fit by REML. t-tests use Satterthwaite's method [
## lmerModLmerTest]
## Formula: scale(i_day_prop) ~ 1 + scale(RADS_total) + scale(Age.at.V2) +
## Sex + (1 | id_participant)
## Data: df_aim1_all_language
##
## REML criterion at convergence: 6089.1
##
## Scaled residuals:
## Min 1Q Median 3Q Max
## -3.1275 -0.5758 -0.0125 0.5057 7.4829
##
## Random effects:
## Groups Name Variance Std.Dev.
## id_participant (Intercept) 0.1464 0.3826
## Residual 0.8430 0.9182
## Number of obs: 2251, groups: id_participant, 40
##
## Fixed effects:
## Estimate Std. Error df t value Pr(>|t|)
## (Intercept) -0.18796 0.25294 31.83071 -0.743 0.4629
## scale(RADS_total) 0.16888 0.06885 32.48517 2.453 0.0197 *
## scale(Age.at.V2) -0.01481 0.07313 32.40718 -0.202 0.8408
## Sex 0.12288 0.14862 32.79945 0.827 0.4143
## ---
## Signif. codes: 0 '***' 0.001 '**' 0.01 '*' 0.05 '.' 0.1 ' ' 1
##
## Correlation of Fixed Effects:
## (Intr) s(RADS s(A..V
## scl(RADS_t) 0.302
## scl(Ag..V2) -0.113 0.083
## Sex -0.960 -0.292 0.064

summary(fit2)

##
## Call:
## lm(formula = scale(leftCEN_Z) ~ scale(RADS_total) + scale(Age.at.V2) +
## Sex + scale(motion), data = df_px_means, na.action = na.exclude)
##
## Residuals:
## Min 1Q Median 3Q Max
## -2.07530 -0.41132 0.00705 0.47821 1.86797
##
## Coefficients:
## Estimate Std. Error t value Pr(>|t|)
## (Intercept) -0.47847 0.59844 -0.800 0.4305
## scale(RADS_total) -0.45328 0.17663 -2.566 0.0157 *
## scale(Age.at.V2) 0.14369 0.17896 0.803 0.4286
## Sex 0.29696 0.35020 0.848 0.4034
## scale(motion) -0.02808 0.18528 -0.152 0.8806
## ---
## Signif. codes: 0 '***' 0.001 '**' 0.01 '*' 0.05 '.' 0.1 ' ' 1
##
## Residual standard error: 0.9416 on 29 degrees of freedom
## (6 observations deleted due to missingness)
## Multiple R-squared: 0.2209, Adjusted R-squared: 0.1134
## F-statistic: 2.056 on 4 and 29 DF, p-value: 0.1126

summary(fit3)

## Linear mixed model fit by REML. t-tests use Satterthwaite's method [
## lmerModLmerTest]
## Formula: scale(i_day_prop) ~ 1 + scale(RADS_total) + scale(leftCEN_Z) +
## scale(Age.at.V2) + Sex + scale(motion) + (1 | id_participant)
## Data: df_aim1_all_language
##
## REML criterion at convergence: 5579.4
##
## Scaled residuals:
## Min 1Q Median 3Q Max
## -3.1017 -0.5697 -0.0135 0.4996 7.5056
##
## Random effects:
## Groups Name Variance Std.Dev.
## id_participant (Intercept) 0.1397 0.3737
## Residual 0.8343 0.9134
## Number of obs: 2070, groups: id_participant, 34
##
## Fixed effects:
## Estimate Std. Error df t value Pr(>|t|)
## (Intercept) -0.03212 0.27273 25.11935 -0.118 0.90719
## scale(RADS_total) 0.31981 0.08896 30.72520 3.595 0.00112 **
## scale(leftCEN_Z) 0.19726 0.08339 30.75966 2.365 0.02450 *
## scale(Age.at.V2) -0.01260 0.08270 25.10146 -0.152 0.88014
## Sex 0.02547 0.16104 26.01476 0.158 0.87557
## scale(motion) 0.04975 0.08736 25.10286 0.569 0.57411
## ---
## Signif. codes: 0 '***' 0.001 '**' 0.01 '*' 0.05 '.' 0.1 ' ' 1
##
## Correlation of Fixed Effects:
## (Intr) s(RADS s(CEN_ s(A..V Sex
## scl(RADS_t) 0.344
## scl(lCEN_Z) 0.194 0.528
## scl(Ag..V2) -0.170 0.090 -0.084
## Sex -0.961 -0.348 -0.199 0.110
## scale(motn) -0.046 0.234 0.056 0.368 0.068

standardize_parameters(fit1)

## # Standardization method: refit
##
## Parameter | Std. Coef. | 95% CI
## ----------------------------------------------
## (Intercept) | 3.64e-03 | [-0.14, 0.14]
## scale(RADS_total) | 0.17 | [ 0.03, 0.30]
## scale(Age.at.V2) | -0.01 | [-0.16, 0.13]
## Sex | 0.06 | [-0.08, 0.21]

standardize_parameters(fit2)

## # Standardization method: refit
##
## Parameter | Std. Coef. | 95% CI
## -----------------------------------------------
## (Intercept) | -1.09e-17 | [-0.33, 0.33]
## scale(RADS_total) | -0.45 | [-0.82, -0.09]
## scale(Age.at.V2) | 0.14 | [-0.22, 0.51]
## Sex | 0.14 | [-0.20, 0.49]
## scale(motion) | -0.03 | [-0.41, 0.35]

standardize_parameters(fit3)

## # Standardization method: refit
##
## Parameter | Std. Coef. | 95% CI
## ----------------------------------------------
## (Intercept) | 0.02 | [-0.13, 0.17]
## scale(RADS_total) | 0.32 | [ 0.14, 0.49]
## scale(leftCEN_Z) | 0.20 | [ 0.03, 0.36]
## scale(Age.at.V2) | -0.01 | [-0.18, 0.15]
## Sex | 0.01 | [-0.14, 0.17]
## scale(motion) | 0.05 | [-0.12, 0.22]

# CI for a*b:
c=summary(fit1)$coefficients[2,1]
a = summary(fit2)$coefficients[2,1]
se.a = summary(fit2)$coefficients[2,2]
b = summary(fit3)$coefficients[2,1]
se.b = summary(fit3)$coefficients[2,2]


medci(mu.x=a, mu.y=b, se.x=se.a, se.y=se.b, rho=0, alpha=.05, type="prodclin", plot=TRUE, plotCI=TRUE)

## Warning in arrows(MedCI[[1]][1], yci, MedCI[[1]][2], yci, length = smidge, :
## 'length', 'angle', or 'code' greater than length 1; values after the first are
## ignored


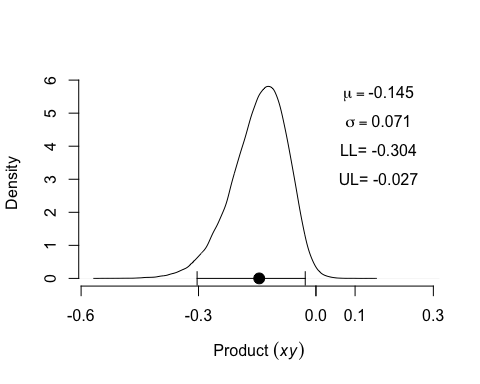


## $`95% CI`
## [1] -0.30362670 -0.02734489
##
## $Estimate
## [1] -0.1449644
##
## $SE
## [1] 0.07116003

# 4. AIM 4 - Does intrinsic connectivity moderate the association between depression and depression-related linguistic features of smartphone use?

## 4b. MDD - Negative Emotion Words

### 4b.3a Between-Network sn_lcen

model4b.3a <- lmer(formula = negemo_day_prop ~
 sn_lcen*Group +
 (1 |id_participant),
 data = df_aim1_all_language,
 na.action=na.exclude)
summary(model4b.3a)

## Linear mixed model fit by REML. t-tests use Satterthwaite's method [
## lmerModLmerTest]
## Formula: negemo_day_prop ~ sn_lcen * Group + (1 | id_participant)
## Data: df_aim1_all_language
##
## REML criterion at convergence: 7485.8
##
## Scaled residuals:
## Min 1Q Median 3Q Max
## -2.2121 -0.5773 -0.0897 0.3700 10.1159
##
## Random effects:
## Groups Name Variance Std.Dev.
## id_participant (Intercept) 0.2163 0.4651
## Residual 2.1224 1.4569
## Number of obs: 2070, groups: id_participant, 34
##
## Fixed effects:
## Estimate Std. Error df t value Pr(>|t|)
## (Intercept) 0.8543 0.3898 32.9573 2.191 0.035603 *
## sn_lcen 1.4750 0.7463 30.6850 1.976 0.057149 .
## GroupMDD 1.7855 0.4349 32.3227 4.106 0.000256 ***
## sn_lcen:GroupMDD -1.8281 0.8749 29.7908 -2.089 0.045314 *
## ---
## Signif. codes: 0 '***' 0.001 '**' 0.01 '*' 0.05 '.' 0.1 ' ' 1
##
## Correlation of Fixed Effects:
## (Intr) sn_lcn GrpMDD
## sn_lcen -0.891
## GroupMDD -0.896 0.798
## sn_lcn:GMDD 0.760 -0.853 -0.870

standardize_parameters(model4b.3a)

## # Standardization method: refit
##
## Parameter | Std. Coef. | 95% CI
## ----------------------------------------------
## (Intercept) | -0.47 | [-0.69, -0.24]
## sn_lcen | 0.21 | [ 0.00, 0.42]
## GroupMDD | 0.63 | [ 0.36, 0.90]
## sn_lcen:GroupMDD | -0.26 | [-0.50, -0.02]

### 4b.3b Age

model4b.3b <- lmer(formula = negemo_day_prop ~
 sn_lcen*Group
 + Age.at.V2
 + (1 |id_participant),
 data = df_aim1_all_language,
 na.action=na.exclude)
summary(model4b.3b)

## Linear mixed model fit by REML. t-tests use Satterthwaite's method [
## lmerModLmerTest]
## Formula: negemo_day_prop ~ sn_lcen * Group + Age.at.V2 + (1 | id_participant)
## Data: df_aim1_all_language
##
## REML criterion at convergence: 7488.3
##
## Scaled residuals:
## Min 1Q Median 3Q Max
## -2.2235 -0.5784 -0.0911 0.3684 10.1145
##
## Random effects:
## Groups Name Variance Std.Dev.
## id_participant (Intercept) 0.2184 0.4673
## Residual 2.1228 1.4570
## Number of obs: 2070, groups: id_participant, 34
##
## Fixed effects:
## Estimate Std. Error df t value Pr(>|t|)
## (Intercept) 1.84108 1.54135 26.78971 1.194 0.242769
## sn_lcen 1.47343 0.74904 29.22948 1.967 0.058728 .
## GroupMDD 1.84968 0.44706 31.33218 4.137 0.000245 ***
## Age.at.V2 -0.06086 0.09195 27.00669 -0.662 0.513652
## sn_lcen:GroupMDD -1.98570 0.91013 29.02716 -2.182 0.037367 *
## ---
## Signif. codes: 0 '***' 0.001 '**' 0.01 '*' 0.05 '.' 0.1 ' ' 1
##
## Correlation of Fixed Effects:
## (Intr) sn_lcn GrpMDD Ag..V2
## sn_lcen -0.228
## GroupMDD -0.013 0.779
## Age.at.V2 -0.967 0.002 -0.217
## sn_lcn:GMDD -0.068 -0.822 -0.877 0.262

standardize_parameters(model4b.3b)

## # Standardization method: refit
##
## Parameter | Std. Coef. | 95% CI
## ----------------------------------------------
## (Intercept) | -0.46 | [-0.68, -0.23]
## sn_lcen | 0.21 | [ 0.00, 0.42]
## GroupMDD | 0.63 | [ 0.36, 0.90]
## Age.at.V2 | -0.05 | [-0.19, 0.10]
## sn_lcen:GroupMDD | -0.28 | [-0.53, -0.03]

#Model comparison with LRT
anova(model4b.3a, model4b.3b)

## refitting model(s) with ML (instead of REML)

## Data: df_aim1_all_language
## Models:
## model4b.3a: negemo_day_prop ~ sn_lcen * Group + (1 | id_participant)
## model4b.3b: negemo_day_prop ~ sn_lcen * Group + Age.at.V2 + (1 | id_participant)
## npar AIC BIC logLik deviance Chisq Df Pr(>Chisq)
## model4b.3a 6 7494.9 7528.7 -3741.5 7482.9
## model4b.3b 7 7496.3 7535.8 -3741.2 7482.3 0.574 1 0.4487

### 4b.3c Sex

model4b.3c <- lmer(formula = negemo_day_prop ~
 sn_lcen*Group
 + Sex
 + (1 |id_participant),
 data = df_aim1_all_language,
 na.action=na.exclude)
summary(model4b.3c)

## Linear mixed model fit by REML. t-tests use Satterthwaite's method [
## lmerModLmerTest]
## Formula: negemo_day_prop ~ sn_lcen * Group + Sex + (1 | id_participant)
## Data: df_aim1_all_language
##
## REML criterion at convergence: 7487
##
## Scaled residuals:
## Min 1Q Median 3Q Max
## -2.2197 -0.5653 -0.0884 0.3704 10.1181
##
## Random effects:
## Groups Name Variance Std.Dev.
## id_participant (Intercept) 0.2254 0.4747
## Residual 2.1223 1.4568
## Number of obs: 2070, groups: id_participant, 34
##
## Fixed effects:
## Estimate Std. Error df t value Pr(>|t|)
## (Intercept) 0.6917 0.5003 31.3551 1.383 0.176542
## sn_lcen 1.4704 0.7581 30.0131 1.940 0.061861 .
## GroupMDD 1.7907 0.4417 31.6661 4.054 0.000305 ***
## Sex 0.1053 0.1981 26.8360 0.532 0.599296
## sn_lcen:GroupMDD -1.8622 0.8916 29.4955 -2.089 0.045472 *
## ---
## Signif. codes: 0 '***' 0.001 '**' 0.01 '*' 0.05 '.' 0.1 ' ' 1
##
## Correlation of Fixed Effects:
## (Intr) sn_lcn GrpMDD Sex
## sn_lcen -0.700
## GroupMDD -0.722 0.798
## Sex -0.612 -0.007 0.021
## sn_lcn:GMDD 0.646 -0.850 -0.869 -0.077

standardize_parameters(model4b.3c)

## # Standardization method: refit
##
## Parameter | Std. Coef. | 95% CI
## ----------------------------------------------
## (Intercept) | -0.47 | [-0.69, -0.24]
## sn_lcen | 0.21 | [ 0.00, 0.42]
## GroupMDD | 0.63 | [ 0.35, 0.90]
## Sex | 0.03 | [-0.09, 0.16]
## sn_lcen:GroupMDD | -0.26 | [-0.51, -0.02]

#Model comparison with LRT
anova(model4b.3a, model4b.3c)

## refitting model(s) with ML (instead of REML)

## Data: df_aim1_all_language
## Models:
## model4b.3a: negemo_day_prop ~ sn_lcen * Group + (1 | id_participant)
## model4b.3c: negemo_day_prop ~ sn_lcen * Group + Sex + (1 | id_participant)
## npar AIC BIC logLik deviance Chisq Df Pr(>Chisq)
## model4b.3a 6 7494.9 7528.7 -3741.5 7482.9
## model4b.3c 7 7496.6 7536.1 -3741.3 7482.6 0.2974 1 0.5855

### 4b.3d Motion

model4b.3d <- lmer(formula = negemo_day_prop ~
 sn_lcen*Group
 + motion
 + (1 |id_participant),
 data = df_aim1_all_language,
 na.action=na.exclude)
summary(model4b.3d)

## Linear mixed model fit by REML. t-tests use Satterthwaite's method [
## lmerModLmerTest]
## Formula: negemo_day_prop ~ sn_lcen * Group + motion + (1 | id_participant)
## Data: df_aim1_all_language
##
## REML criterion at convergence: 7479.6
##
## Scaled residuals:
## Min 1Q Median 3Q Max
## -2.2239 -0.5653 -0.0856 0.3699 10.1088
##
## Random effects:
## Groups Name Variance Std.Dev.
## id_participant (Intercept) 0.2084 0.4565
## Residual 2.1223 1.4568
## Number of obs: 2070, groups: id_participant, 34
##
## Fixed effects:
## Estimate Std. Error df t value Pr(>|t|)
## (Intercept) 1.2122 0.4625 27.9378 2.621 0.014033 *
## sn_lcen 1.7707 0.7656 30.3904 2.313 0.027678 *
## GroupMDD 1.8264 0.4300 31.5014 4.248 0.000178 ***
## motion -4.8653 3.4927 25.4616 -1.393 0.175673
## sn_lcen:GroupMDD -2.0717 0.8798 29.2846 -2.355 0.025449 *
## ---
## Signif. codes: 0 '***' 0.001 '**' 0.01 '*' 0.05 '.' 0.1 ' ' 1
##
## Correlation of Fixed Effects:
## (Intr) sn_lcn GrpMDD motion
## sn_lcen -0.559
## GroupMDD -0.706 0.785
## motion -0.556 -0.276 -0.068
## sn_lcn:GMDD 0.510 -0.858 -0.865 0.197

standardize_parameters(model4b.3d)

## # Standardization method: refit
##
## Parameter | Std. Coef. | 95% CI
## ----------------------------------------------
## (Intercept) | -0.45 | [-0.67, -0.23]
## sn_lcen | 0.25 | [ 0.04, 0.46]
## GroupMDD | 0.59 | [ 0.32, 0.86]
## motion | -0.10 | [-0.23, 0.04]
## sn_lcen:GroupMDD | -0.29 | [-0.54, -0.05]

### 4b.4a Between-Network sn_rcen

model4b.4a <- lmer(formula = negemo_day_prop ~
 sn_rcen*Group +
 (1 |id_participant),
 data = df_aim1_all_language,
 na.action=na.exclude)
summary(model4b.4a)

## Linear mixed model fit by REML. t-tests use Satterthwaite's method [
## lmerModLmerTest]
## Formula: negemo_day_prop ~ sn_rcen * Group + (1 | id_participant)
## Data: df_aim1_all_language
##
## REML criterion at convergence: 7485.4
##
## Scaled residuals:
## Min 1Q Median 3Q Max
## -2.2129 -0.5816 -0.0899 0.3740 10.1150
##
## Random effects:
## Groups Name Variance Std.Dev.
## id_participant (Intercept) 0.2116 0.460
## Residual 2.1225 1.457
## Number of obs: 2070, groups: id_participant, 34
##
## Fixed effects:
## Estimate Std. Error df t value Pr(>|t|)
## (Intercept) 0.7790 0.4132 25.5073 1.885 0.070818 .
## sn_rcen 1.7357 0.8515 24.1289 2.038 0.052608 .
## GroupMDD 1.8582 0.4469 25.8942 4.158 0.000311 ***
## sn_rcen:GroupMDD -2.0991 0.9388 24.4947 -2.236 0.034721 *
## ---
## Signif. codes: 0 '***' 0.001 '**' 0.01 '*' 0.05 '.' 0.1 ' ' 1
##
## Correlation of Fixed Effects:
## (Intr) sn_rcn GrpMDD
## sn_rcen -0.905
## GroupMDD -0.925 0.837
## sn_rcn:GMDD 0.821 -0.907 -0.882

standardize_parameters(model4b.4a)

## # Standardization method: refit
##
## Parameter | Std. Coef. | 95% CI
## ----------------------------------------------
## (Intercept) | -0.49 | [-0.71, -0.26]
## sn_rcen | 0.26 | [ 0.01, 0.50]
## GroupMDD | 0.66 | [ 0.39, 0.93]
## sn_rcen:GroupMDD | -0.31 | [-0.58, -0.04]

### 4b.4b Age

model4b.4b <- lmer(formula = negemo_day_prop ~
 sn_rcen*Group
 + Age.at.V2
 + (1 |id_participant),
 data = df_aim1_all_language,
 na.action=na.exclude)
summary(model4b.4b)

## Linear mixed model fit by REML. t-tests use Satterthwaite's method [
## lmerModLmerTest]
## Formula: negemo_day_prop ~ sn_rcen * Group + Age.at.V2 + (1 | id_participant)
## Data: df_aim1_all_language
##
## REML criterion at convergence: 7488.4
##
## Scaled residuals:
## Min 1Q Median 3Q Max
## -2.2243 -0.5797 -0.0910 0.3746 10.1142
##
## Random effects:
## Groups Name Variance Std.Dev.
## id_participant (Intercept) 0.2189 0.4678
## Residual 2.1226 1.4569
## Number of obs: 2070, groups: id_participant, 34
##
## Fixed effects:
## Estimate Std. Error df t value Pr(>|t|)
## (Intercept) 1.34151 1.46959 24.30087 0.913 0.370290
## sn_rcen 1.71446 0.86526 23.14158 1.981 0.059545 .
## GroupMDD 1.87167 0.45407 24.95297 4.122 0.000363 ***
## Age.at.V2 -0.03419 0.08548 24.66692 -0.400 0.692608
## sn_rcen:GroupMDD -2.12699 0.95471 23.59655 -2.228 0.035690 *
## ---
## Signif. codes: 0 '***' 0.001 '**' 0.01 '*' 0.05 '.' 0.1 ' ' 1
##
## Correlation of Fixed Effects:
## (Intr) sn_rcn GrpMDD Ag..V2
## sn_rcen -0.317
## GroupMDD -0.197 0.829
## Age.at.V2 -0.959 0.062 -0.068
## sn_rcn:GMDD 0.162 -0.898 -0.882 0.074

standardize_parameters(model4b.4b)

## # Standardization method: refit
##
## Parameter | Std. Coef. | 95% CI
## ----------------------------------------------
## (Intercept) | -0.48 | [-0.71, -0.25]
## sn_rcen | 0.25 | [ 0.00, 0.50]
## GroupMDD | 0.66 | [ 0.39, 0.93]
## Age.at.V2 | -0.03 | [-0.16, 0.11]
## sn_rcen:GroupMDD | -0.31 | [-0.59, -0.04]

#Model comparison with LRT
anova(model4b.4a, model4b.4b)

## refitting model(s) with ML (instead of REML)

## Data: df_aim1_all_language
## Models:
## model4b.4a: negemo_day_prop ~ sn_rcen * Group + (1 | id_participant)
## model4b.4b: negemo_day_prop ~ sn_rcen * Group + Age.at.V2 + (1 | id_participant)
## npar AIC BIC logLik deviance Chisq Df Pr(>Chisq)
## model4b.4a 6 7494.4 7528.2 -3741.2 7482.4
## model4b.4b 7 7496.2 7535.6 -3741.1 7482.2 0.222 1 0.6375

### 4b.4c Sex

model4b.4c <- lmer(formula = negemo_day_prop ~
 sn_rcen*Group
 + Sex
 + (1 |id_participant),
 data = df_aim1_all_language,
 na.action=na.exclude)
summary(model4b.4c)

## Linear mixed model fit by REML. t-tests use Satterthwaite's method [
## lmerModLmerTest]
## Formula: negemo_day_prop ~ sn_rcen * Group + Sex + (1 | id_participant)
## Data: df_aim1_all_language
##
## REML criterion at convergence: 7486.8
##
## Scaled residuals:
## Min 1Q Median 3Q Max
## -2.2194 -0.5777 -0.0901 0.3740 10.1149
##
## Random effects:
## Groups Name Variance Std.Dev.
## id_participant (Intercept) 0.2225 0.4717
## Residual 2.1224 1.4568
## Number of obs: 2070, groups: id_participant, 34
##
## Fixed effects:
## Estimate Std. Error df t value Pr(>|t|)
## (Intercept) 0.73341 0.48348 24.90931 1.517 0.141869
## sn_rcen 1.70298 0.88728 23.56503 1.919 0.067132 .
## GroupMDD 1.84709 0.46108 24.88537 4.006 0.000491 ***
## Sex 0.03768 0.20145 25.86162 0.187 0.853086
## sn_rcen:GroupMDD -2.07864 0.96444 23.68084 -2.155 0.041527 *
## ---
## Signif. codes: 0 '***' 0.001 '**' 0.01 '*' 0.05 '.' 0.1 ' ' 1
##
## Correlation of Fixed Effects:
## (Intr) sn_rcn GrpMDD Sex
## sn_rcen -0.677
## GroupMDD -0.726 0.841
## Sex -0.489 -0.198 -0.147
## sn_rcn:GMDD 0.659 -0.905 -0.883 0.109

standardize_parameters(model4b.4c)

## # Standardization method: refit
##
## Parameter | Std. Coef. | 95% CI
## ----------------------------------------------
## (Intercept) | -0.49 | [-0.72, -0.26]
## sn_rcen | 0.25 | [-0.01, 0.51]
## GroupMDD | 0.66 | [ 0.38, 0.93]
## Sex | 0.01 | [-0.11, 0.14]
## sn_rcen:GroupMDD | -0.31 | [-0.58, -0.03]

#Model comparison with LRT
anova(model4b.4a, model4b.4c)

## refitting model(s) with ML (instead of REML)

## Data: df_aim1_all_language
## Models:
## model4b.4a: negemo_day_prop ~ sn_rcen * Group + (1 | id_participant)
## model4b.4c: negemo_day_prop ~ sn_rcen * Group + Sex + (1 | id_participant)
## npar AIC BIC logLik deviance Chisq Df Pr(>Chisq)
## model4b.4a 6 7494.4 7528.2 -3741.2 7482.4
## model4b.4c 7 7496.4 7535.8 -3741.2 7482.4 0.024 1 0.8768

### 4b.4d Motion

model4b.4d <- lmer(formula = negemo_day_prop ~
 sn_rcen*Group
 + motion
 + (1 |id_participant),
 data = df_aim1_all_language,
 na.action=na.exclude)
summary(model4b.4d)

## Linear mixed model fit by REML. t-tests use Satterthwaite's method [
## lmerModLmerTest]
## Formula: negemo_day_prop ~ sn_rcen * Group + motion + (1 | id_participant)
## Data: df_aim1_all_language
##
## REML criterion at convergence: 7480.3
##
## Scaled residuals:
## Min 1Q Median 3Q Max
## -2.2247 -0.5820 -0.0887 0.3735 10.1100
##
## Random effects:
## Groups Name Variance Std.Dev.
## id_participant (Intercept) 0.2158 0.4646
## Residual 2.1222 1.4568
## Number of obs: 2070, groups: id_participant, 34
##
## Fixed effects:
## Estimate Std. Error df t value Pr(>|t|)
## (Intercept) 1.0669 0.5236 25.5266 2.038 0.052081 .
## sn_rcen 1.8010 0.8615 23.3876 2.090 0.047633 *
## GroupMDD 1.8239 0.4521 25.3541 4.035 0.000444 ***
## motion -3.1171 3.4291 23.8921 -0.909 0.372429
## sn_rcen:GroupMDD -2.1223 0.9469 23.8546 -2.241 0.034575 *
## ---
## Signif. codes: 0 '***' 0.001 '**' 0.01 '*' 0.05 '.' 0.1 ' ' 1
##
## Correlation of Fixed Effects:
## (Intr) sn_rcn GrpMDD motion
## sn_rcen -0.667
## GroupMDD -0.784 0.824
## motion -0.606 -0.083 0.085
## sn_rcn:GMDD 0.636 -0.906 -0.876 0.027

standardize_parameters(model4b.4d)

## # Standardization method: refit
##
## Parameter | Std. Coef. | 95% CI
## ----------------------------------------------
## (Intercept) | -0.47 | [-0.70, -0.25]
## sn_rcen | 0.26 | [ 0.02, 0.51]
## GroupMDD | 0.63 | [ 0.35, 0.91]
## motion | -0.06 | [-0.20, 0.07]
## sn_rcen:GroupMDD | -0.31 | [-0.59, -0.04]
